# Incidence, mortality, risk factors, and trends for Hodgkin lymphoma: a global data analysis

**DOI:** 10.1186/s13045-022-01281-9

**Published:** 2022-05-11

**Authors:** Junjie Huang, Wing Sze Pang, Veeleah Lok, Lin Zhang, Don Eliseo Lucero-Prisno, Wanghong Xu, Zhi-Jie Zheng, Edmar Elcarte, Mellissa Withers, Martin C. S. Wong, NCD Global Health Research Group, Association of Pacific Rim Universities (APRU)

**Affiliations:** 1 The Jockey Club School of Public Health and Primary Care, Faculty of Medicine, Chinese University of Hong Kong, Room 407, 4/F, Postgraduate Education Centre, Prince of Wales Hospital, 30-32 Ngan Shing Street, Shatin, NT, Hong Kong SAR China; 2 Department of Global Public Health, Karolinska Institute, Karolinska University Hospital, Stockholm, Sweden; 3 School of Population and Global Health, The University of Melbourne, Melbourne, VIC Australia; 4 School of Public Health, The Chinese Academy of Medical Sciences and Peking Union Medical College, Beijing, China; 5 Department of Global Health and Development, London School of Hygiene and Tropical Medicine, London, UK; 6 School of Public Health, Fudan University, Shanghai, China; 7 Department of Global Health, School of Public Health, Peking University, Beijing, China; 8 University of the Philippines, Manila, The Philippines; 9 Department of Population and Health Sciences, USC Institute On Inequalities in Global Health, APRU Global Health Program, Keck School of Medicine of USC, University of Southern California, Soto Street Building, SSB318G, 2001 North Soto Street, MC 9239, Los Angeles, CA 90089-9239 USA

**Keywords:** Hodgkin lymphoma, Incidence, Mortality, Risk factors, Temporal trend

## Abstract

**Background:**

Hodgkin lymphoma is a lymphatic malignancy commonly found in cervical lymph nodes. This study evaluated the worldwide incidence, mortality, associated risk factors, and temporal trends of Hodgkin lymphoma by sex, age, and country.

**Methods:**

The age-standardised Hodgkin lymphoma incidence and mortality were retrieved from the *GLOBOCAN*, *CI5* volumes I-XI, *WHO mortality database*, the *NORDCAN and SEER Program*. The age-standardised prevalence of smoking, alcohol drinking, obesity, and hypertension was also extracted for each country. Trends were tested using Average Annual Percentage Change (AAPC) from Joinpoint regression analysis.

**Results:**

The Hodgkin lymphoma incidence and mortality were 0.98 and 0.26 per 100,000 in 2020. A higher incidence was observed in high-income countries, while higher mortality was found in low-income countries. Incidence and mortality were associated with GDP per capita, prevalence of smoking, obesity, and hypertension at the population level. Despite the decreasing mortality trend, there was an increasing incidence, especially among females, younger population, and subjects from Asian countries.

**Conclusions:**

There was an increasing trend in Hodgkin lymphoma incidence, especially among subjects who were female, younger population, and from Asian countries. Further studies are needed to investigate the reasons for these epidemiologic trends.

**Supplementary Information:**

The online version contains supplementary material available at 10.1186/s13045-022-01281-9.

## Introduction

Hodgkin lymphoma is a lymphatic malignancy commonly found in cervical lymph nodes and can be classified into classical Hodgkin lymphoma and nodular lymphocyte-predominant Hodgkin lymphoma [1]. Globally, 0.4% and 0.2% of all newly reported cancer-related cases and deaths were due to Hodgkin lymphoma in 2020 [2]. Although it was relatively rare, it is the most common cancer among youngsters aged 15–19 years [3]. Recently, the survival rate of Hodgkin lymphoma has been improved due to the advances in antibody therapy. Other promising therapies included vaccine therapies, checkpoint inhibitors, and cytotoxic T lymphocytes [4, 5].

The burden of Hodgkin lymphoma varies with gender sex, age, and geographical location. People with a higher risk of Hodgkin lymphoma include males [1], adolescents and young adults [4], those with past history of Epstein-Barr virus infection [6], HIV/AIDS [7], autoimmune diseases [8], exposure to pollution [9], cigarette smoking [10], and family history. It was also found that the incidence of Hodgkin lymphoma varied by family size and socio-economic status [8]. Since the epidemiology of Hodgkin lymphoma is different across regions and may have changed over time, its global distribution pattern, risk factors, and temporal trends need to be assessed for developing tailored preventive measures for individual countries.

There are limited studies discussing the recent global trends of Hodgkin lymphoma, the prior studies are constrained to specific countries or age groups [8 9]. Therefore, a more comprehensive and worldwide analysis is needed by reporting the most updated data. This study aims to fill this research gap by 1) examining the most recent global incidence and mortality of Hodgkin lymphoma by location, sex, and socio-economic level; 2) investigating the relationship between common lifestyle and metabolic risk factors and the burden of Hodgkin lymphoma by sex and age; and 3) examining the recent temporal trends of Hodgkin lymphoma incidence and mortality among different age groups, sexes, and regions.

## Methods

### Data sources

Comprehensive and updated information of incidence and mortality rates of Hodgkin lymphoma was extracted from the Global Cancer Observatory (*GLOBOCAN)* database [11]. The United Nations and the World Bank were accessed for Human Development Index (HDI) and gross domestic products (GDP) per capita, respectively, for each country [12]. The prevalence of lifestyle and metabolic risk factors was examined for each country using data from the *Global Health Data Exchange* (*GHDx*), including the sex and age-specific prevalence of current smoking, alcohol drinking, overweight, obesity, and hypertension [13].

For cancer incidence trend analysis, the *Cancer Incidence in Five Continents* (*CI5*) volumes I–XI was used as the data source. The *CI5* is a collection of high-quality global cancer registries, which covers a large world population of cancer incidence-related data on regional and country levels [14]. As for the mortality trends, the number was collected from the *WHO cancer mortality database* [15]. Another two data sources, the *Nordic Cancer Registries* (*NORDCAN*) [16, 17] and the *Surveillance, Epidemiology, and End Results* (*SEER*) [18] Program, were accessed for the latest cancer incidence and mortality cases of Northern European countries and the USA, respectively. There was a total of 48 and 46 countries included for the trend analysis of incidence and mortality, respectively. The Additional file 2: Table S1 provides a detailed description of the data source used in the trend analysis. In order to present and compare the data easier across countries, the cancer incidence and mortality numbers were standardised by age using the Segi–Doll world reference population to obtain age-standardised rates (ASRs) for each country. The histological groups of Hodgkin lymphoma in the registries included nodular lymphocytic predominance, classical lymphocyte-rich, nodular sclerosis, mixed cellularity, lymphocytic depletion, and unspecified.

### Statistical analysis

Two choropleth maps were generated to show the worldwide incidence and mortality of Hodgkin lymphoma. The associations between HDI, GDP per capita, lifestyle and metabolic risk factors, and Hodgkin lymphoma incidence and mortality for each country were examined by multivariable linear regression analysis by sex (males and females) and age (50 or above and below 50), respectively. Beta coefficients (*β*) and the corresponding 95% confidence intervals (CI) were generated from the regression. The *β* estimates refer to the degree of change in ASR of Hodgkin lymphoma incidence or mortality per unit increase in the prevalence of risk factors.

For trend analysis, Joinpoint regression analysis software was used, which is developed by the SEER Program under the United States National Cancer Institute. The corresponding Average Annual Percentage Change (AAPC) for different regions and countries was calculated for the temporal trend of Hodgkin lymphoma incidence and mortality [19]. Data of the latest period of 10 years were used as a normal practice in epidemiology research for cancer. The incidence and mortality data had undergone a logarithmic transformation, and related standard errors had been calculated. They were subsequently used to calculate the AAPC and the 95% CI for different population groups. The temporal trends of Hodgkin lymphoma incidence and mortality were indicated by the AAPC, with a positive AAPC indicating an increasing trend and vice versa. The 95% CI was an indicator to assess the reliability of the trend estimates: an interval overlapping with 0 indicates a stable trend without significant increase or decrease trends.

In the current study, the trends Hodgkin lymphoma incidence and mortality of the different population groups were examined, including those of different age groups (0–85+, 50 or above, below 50, and below 40), different sexes (male and female), and different geographical locations (Asia, Oceania, America, Europe, and Africa).

## Results

### Global incidence of Hodgkin lymphoma in 2020

**Fig. 1:**
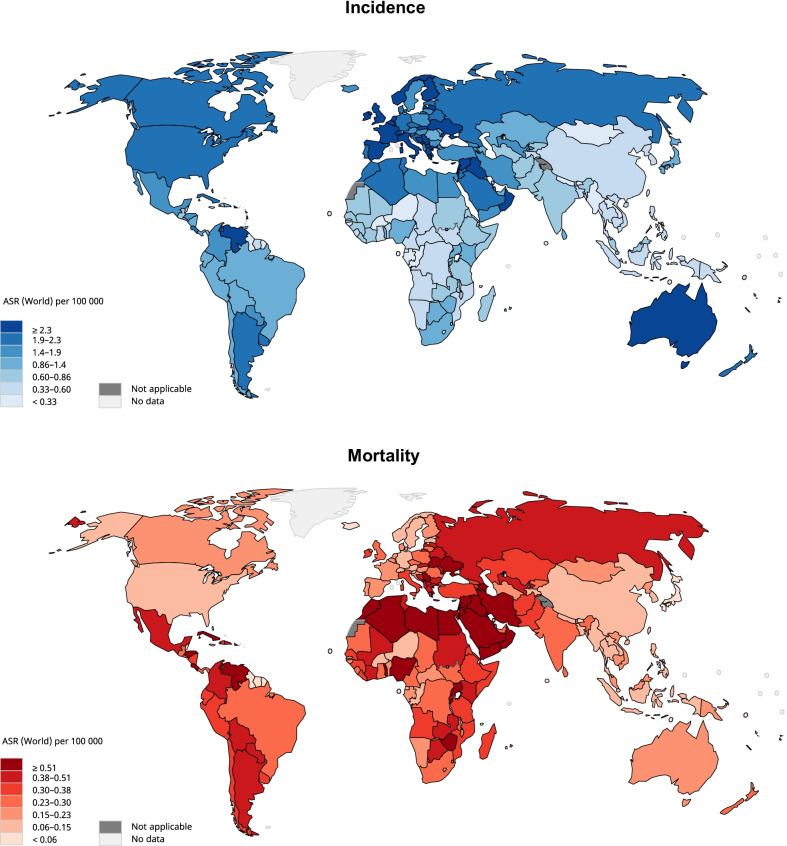
Global incidence and mortality of Hodgkin lymphoma, both sexes, all ages, in 2020

In 2020, a total of 83,087 new cases of Hodgkin lymphoma were reported (Fig. 1). The global age-standardised incidence rate was 0.98 per 100,000 people. There was a sixfold variation in the geographical distribution of incidence. The highest incidence was reported in Southern Europe (ASR = 2.8), followed by Northern Europe (ASR = 2.6), Australia and New Zealand (ASR = 2.6), and Western Europe (ASR = 2.5). Meanwhile, the lowest incidence was found in Eastern Asia (ASR = 0.44), followed by South-Eastern Asia (ASR = 0.45), Middle Africa (ASR = 0.46), and Melanesia (ASR = 0.59). The global incidence of male (ASR = 1.2) was 50% higher than female (ASR = 0.8). The incidence in countries with very high HDI (ASR = 2.0) was higher than that in low-HDI (ASR = 0.83), high-HDI (ASR = 0.79), and medium-HDI (ASR = 0.69) countries (Additional file 2: Table S2).

### Global mortality of Hodgkin lymphoma in 2020

In 2020, a total of 23,376 deaths of Hodgkin lymphoma were recorded worldwide. The ASR of mortality was 0.26 per 100,000 people. Unlike incidence, a smaller difference in mortality rate was observed among geographical regions. High mortality rates were found in Western Asia (ASR = 0.59), Northern Africa (ASR = 0.53), Western Africa (ASR = 0.45), and Central America (ASR = 0.42), while low mortality rates were found in Eastern Asia (ASR = 0.13), South-Eastern Asia (ASR = 0.14), North America (ASR = 0.15), and Western Europe (ASR = 0.17). The mortality rate of males (ASR = 0.33) was 70% higher than that of females (ASR = 0.19). The mortality in low-HDI countries (ASR = 0.43) was considerably higher than that in countries with very high (ASR = 0.23), high (ASR = 0.23), and medium (ASR = 0.27) HDI.

### Associations between risk factors and Hodgkin lymphoma incidence

**Fig. 2:**
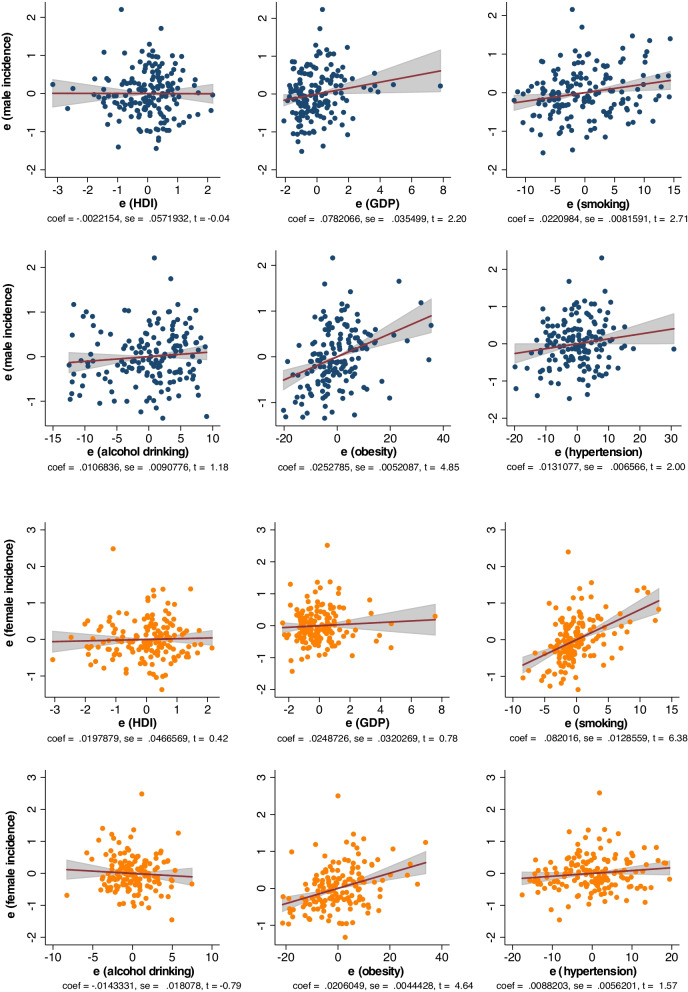
Associations between risk factors and Hodgkin lymphoma incidence

Among males, Hodgkin lymphoma incidence was associated with a higher GDP per capita (*β* = 0.078, CI 0.01 to 0.008, *p* = 0.029), prevalence of smoking (*β* = 0.022, CI 0.006 to 0.038, *p* = 0.008), obesity (*β* = 0.025, CI 0.015 to 0.036, *p* < 0.001), and hypertension (*β* = 0.013, CI 0.001 to 0.026, *p* = 0.048) (Fig. 2). Among females, incidence was associated with a higher prevalence of smoking (*β* = 0.082, CI 0.057 to 0.107, *p* < 0.001) and obesity (*β* = 0.021, CI 0.012 to 0.029, *p* < 0.001). Among subjects aged 50 or above, incidence was associated with a higher prevalence of smoking (*β* = 0.057, CI 0.027 to 0.088, *p* < 0.001) and obesity (*β* = 0.023, CI 0.010 to 0.035, *p* < 0.001) (Additional file 2: Fig. S1). Among subjects aged below 50, incidence was associated with a higher GDP per capita (*β* = 0.148, CI 0.053 to 0.243, *p* = 0.003), prevalence of smoking (*β* = 0.099, CI 0.063 to 0.136, *p* < 0.001), alcohol drinking (*β* = 0.033, CI 0.001 to 0.065, *p* = 0.049), and obesity (*β* = 0.023, CI 0.009 to 0.037, *p* = 0.001).

### Associations between risk factors and Hodgkin lymphoma mortality

**Fig. 3:**
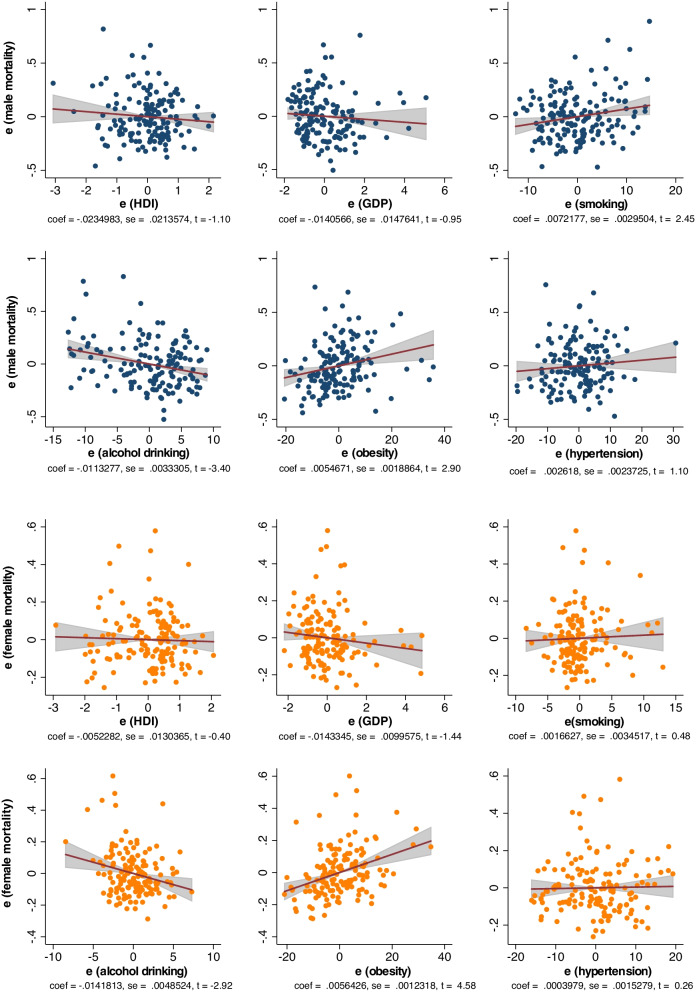
Associations between risk factors and Hodgkin lymphoma mortality

Among males, Hodgkin lymphoma mortality was associated with a higher prevalence of smoking (*β* = 0.007, CI 0.001 to 0.013, *p* = 0.016) and obesity (*β* = 0.005, CI 0.002 to 0.009, *p* = 0.004), but a lower alcohol drinking (*β* = −0.011, CI − 0.018 to − 0.005, *p* = 0.001) (Fig. 3). Among females, mortality was associated with a higher obesity (*β* = 0.006, CI 0.003 to 0.008, *p* < 0.001) but a lower alcohol drinking (*β* = −0.014, CI − 0.024 to − 0.005, *p* = 0.004). In the older subjects, mortality was associated with lower HDI (*β* = −0.106, CI −0.211 to −0.001, *p* = 0.046) and alcohol drinking (*β* = −0.027, CI −0.049 to −0.004, *p* = 0.022) but higher smoking (*β* = 0.021, CI 0.003 to 0.039, *p* = 0.025) and obesity (*β* = 0.013, CI 0.005 to 0.020, *p* = 0.001) (Additional file 1: Fig. 2). In the younger people, mortality was associated with lower alcohol drinking (*β* = −0.006, CI −0.011 to −0.001, *p* = 0.034), but higher smoking (*β* = 0.008, CI 0.002 to 0.014, *p* = 0.012), obesity (*β* = 0.003, CI 0.001 to 0.006, *p* = 0.006), and hypertension (*β* = 0.007, CI 0.003 to 0.011, *p* = 0.001).

### Temporal trends of Hodgkin lymphoma

The Hodgkin lymphoma incidence and mortality trends for each country are shown in Additional file 1: Fig. S3, and the results of Joinpoint regression are presented in Additional file 1: Fig. S4. Overall, there was an increasing trend of Hodgkin lymphoma incidence, especially among subjects who were female, younger population, and from Asian countries. As for Hodgkin lymphoma mortality, there was an overall decreasing trend for the past decade.

### Incidence trends of individuals aged 0–85+

**Fig. 4:**
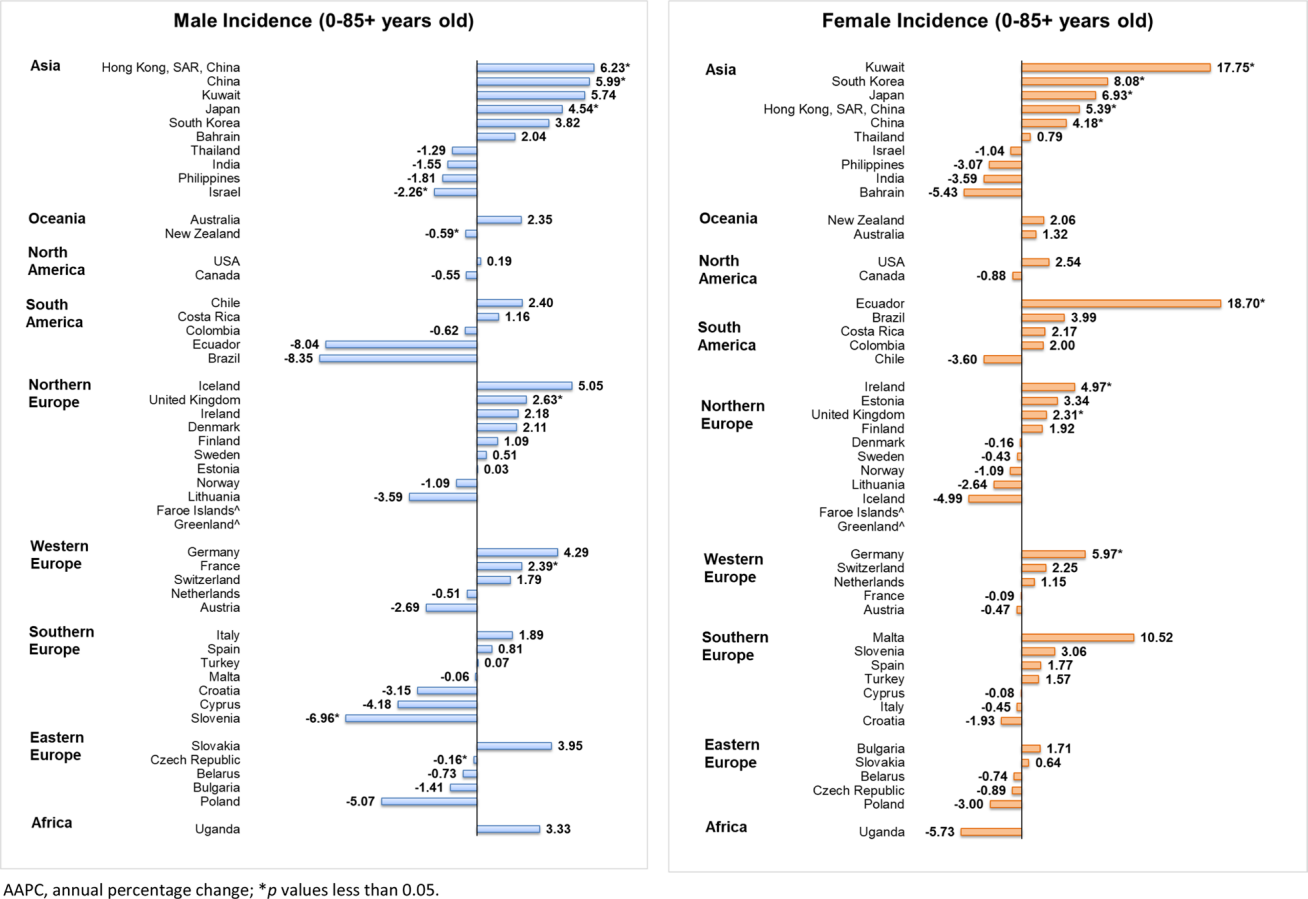
AAPC of Hodgkin lymphoma incidence in individuals aged 0–85+ years old

For males, 7 economies showed a significant increase in Hodgkin lymphoma incidence reported, of which 3 were in Asia (Fig. 4). The most remarkable increases were found in Asian economies, including Hong Kong (AAPC = 6.23, 95% CI 0.96 to 11.78, *p* = 0.025), China (AAPC = 5.99, 95% CI 0.87 to 11.37, *p* = 0.027), and Japan (AAPC = 4.54, 95% CI 0.26 to 9.01, *p* = 0.037). In contrast, only 2 countries reported decreasing trends of Hodgkin lymphoma incidence, which were Slovenia (AAPC = −6.96, 95% CI − 13.44 to 0.02, *p* = 0.050) and Israel (AAPC = −2.26, 95% CI − 4.44 to − 0.03, *p* = 0.047). For females, 9 economies had reported significant increases in Hodgkin lymphoma incidence, of which 5 were Asian countries. The highest increases were reported in Ecuador (AAPC = 18.7, 95% CI 2.41 to 37.59, *p* = 0.028), Kuwait (AAPC = 17.75, 95% CI 5.98 to 30.82, *p* = 0.002), and South Korea (AAPC = 8.08, 95% CI 2.55 to 13.9, *p* = 0.009). However, no significant decrease in Hodgkin lymphoma was found in females.

### Mortality trends of individuals aged 0–85+

**Fig. 5:**
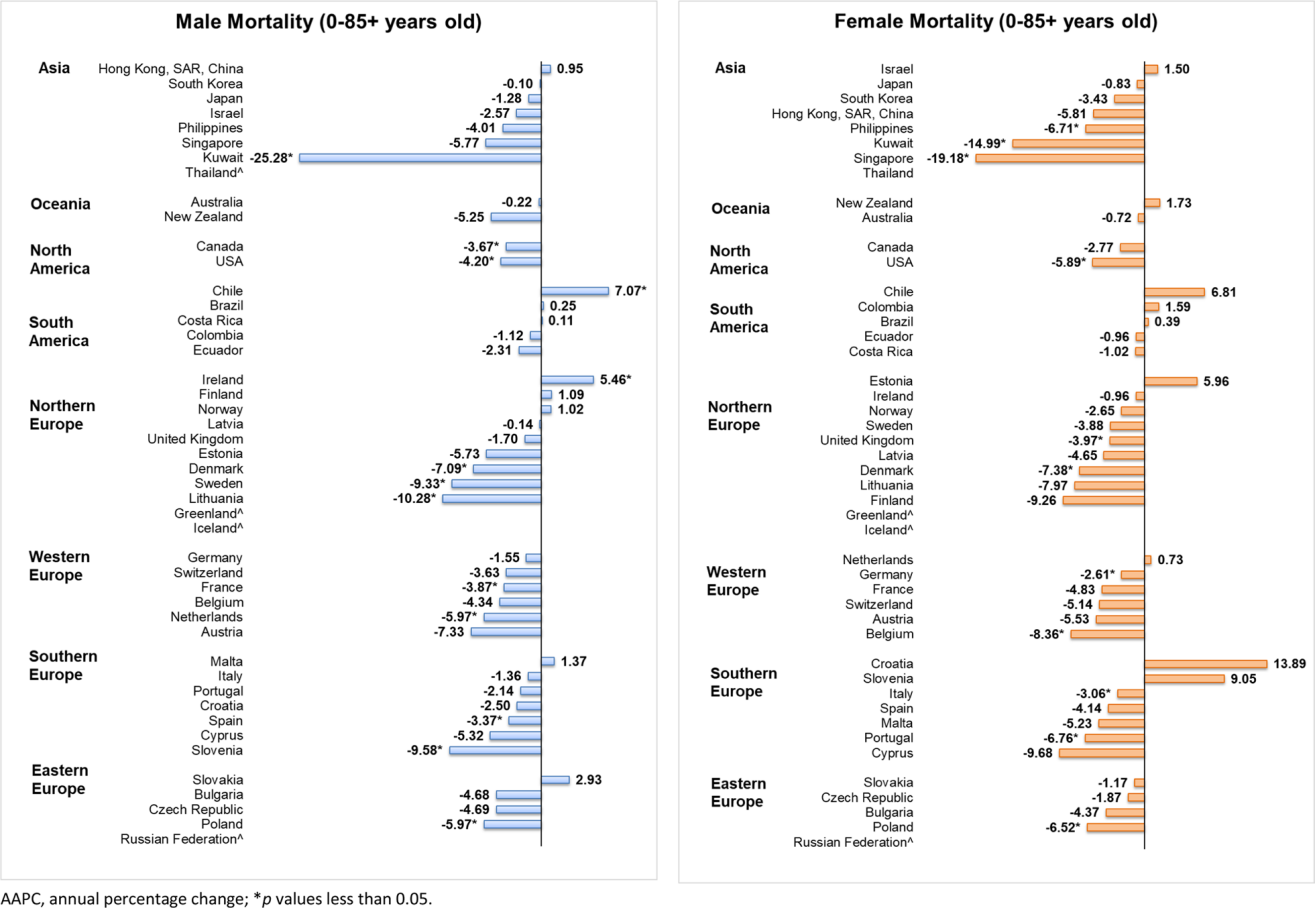
AAPC of Hodgkin lymphoma mortality in individuals aged 0–85+ years old

For males, 11 countries showed significant decreasing trends, with 8 of them from Europe (Fig. 5). Countries with the largest decline were Kuwait (AAPC = −25.28, 95% CI − 41.3 to − 4.9, *p* = 0.024), Lithuania (AAPC = −10.28, 95% CI − 15.51 to − 4.74, *p* = 0.003), and Slovenia (AAPC = −9.58, 95% CI − 16.38 to − 2.21, *p* = 0.018). By contrast, only Chile (AAPC = 7.07, 95% CI 2.38 to 11.98, *p* = 0.008) and Ireland (AAPC = 5.46, 95% CI 0.06 to 11.15, *p* = 0.048) showed significant increases in Hodgkin lymphoma mortality. The sensitivity analysis showed the mortality was consistently increasing in Chile for all single years removed, while the increasing was not statistically significant in Ireland after excluding some potential outliers (Additional file 2: Table S3). For females, a similar pattern was found that 11 countries reported significant decreasing trends, with 9 of them in Europe. The most notable decrease in Hodgkin lymphoma was in Singapore (AAPC = −19.18, 95% CI − 31.53 to − 4.59, *p* = 0.018), followed by Kuwait (AAPC = −14.99, 95% CI − 21.59 to − 7.58, *p* = 0.002) and Belgium (AAPC = −8.36, 95% CI − 13.41 to − 3.01, *p* = 0.008). However, no country had a significant increase in mortality in females.

### Incidence trends of individuals in specific age groups

For men aged 50 or above, 6 economies showed significant increases, of which Hong Kong had the largest increase (AAPC = 6.95, 95% CI 1.24 to 12.97, *p* = 0.022) (Additional file 1: Fig. S5). The significant decreasing trends were reported in 3 countries, with Croatia showing the largest decrease (AAPC = −8.60, 95% CI − 14.5 to − 2.3, *p* = 0.014). For women, 3 countries had significant increases in Hodgkin lymphoma, with most significant increase reported in Ireland (AAPC = 7.34, 95% CI 0.36 to 14.8, *p* = 0.041). However, only Philippines had a significant decrease (AAPC = −9.83, 95% CI − 14.93 to − 4.42, *p* = 0.003).

As for the younger age group in men below age 50, 4 countries showed significant increasing trends, with the most dramatic increase found in South Korea (AAPC = 9.64, 95% CI 1.87 to 18, *p* = 0.020) (Additional file 1: Fig. S6). A decreasing trend was only found in Slovenia (AAPC = −8.44, 95% CI − 16.12 to − 0.06, *p* = 0.049). For women, 4 countries reported significant increases, with South Korea having the largest increase (AAPC = 9.02, 95% CI 2.55 to 15.91, *p* = 0.012), while no country reported a significant decrease.

For men aged below 40, 4 countries showed significant increases, with South Korea reporting the most significant increase (AAPC = 12.22, 95% CI 3.48 to 21.7, *p* = 0.011) (Additional file 1: Fig. S7). However, no country showed a significant decrease in Hodgkin lymphoma. For women, 5 countries showed significant increases, with Malta having the largest increase (AAPC = 13.95, 95% CI 0.5 to 29.21, *p* = 0.043). Only Chile reported a drastic decline (AAPC = −17.16, 95% CI − 25.43 to − 7.98, *p* = 0.003).

## Discussion

### Summary of major findings

Using cancer databases and registries, this analysis presents a thorough, up-to-date evaluation of the global burden, risk factors, and epidemiologic temporal trends of Hodgkin lymphoma by age, sex, and country/region. There are several major findings: 1) There was a wide variation in the Hodgkin lymphoma burden, with higher incidence observed in high-income countries, while higher mortality was found in low-income countries; 2) higher Hodgkin lymphoma burden was associated with GDP, prevalence of smoking, obesity, and hypertension; 3) there was an increasing trend of Hodgkin lymphoma incidence, especially among subjects who were female, younger population, and from Asian countries although its mortality was decreasing.

### Explanations and comparison with the existing literature

The results are generally in line with previous research that found that the Hodgkin lymphoma incidence was higher in North America and Europe and lower in Africa, while its mortality was higher in Africa and lower in East Asia and Australia [20]. The lower Hodgkin lymphoma incidence in low-income countries could be due to the lack of diagnostic resources compared with developed regions. A study found that Asians have been shown to have a dramatically lower Hodgkin lymphoma incidence than other ethnicities, but there are significant incidence differences between US-born Asians (2.9 per 100,000, 95% CI 1.6 to 4.6) and native Asians (1.3 per 100,000, 95% CI 1.2 to 1.4) [21]. Other possible contributors include a higher rate of environmental risk factors and metabolic diseases for Hodgkin lymphoma in high-income countries. Previous studies found that higher levels of living during early childhood had been associated with an increased risk of young-onset Hodgkin lymphoma, while the opposite is true for children living in less favourable living conditions [22–24].

We found that higher Hodgkin lymphoma burden was associated with prevalence of smoking, obesity, and hypertension at the population level, but not alcohol drinking. This is generally in line with previous individual-level research examining the associations between common risk factors and the risk of Hodgkin lymphoma. A study of 3,335 Hodgkin lymphoma patients and 14,278 healthy controls found that smokers had an odds ratio (OR) of Hodgkin lymphoma of 1.10 (95% CI 1.01 to 1.21 compared with never smokers) [25]. Another study found the risk of Hodgkin lymphoma increased by 10% for every 5 kg/m^2^ increase in body mass index (BMI) according to an analysis of 927 Hodgkin lymphoma patients developed from a follow-up of a cohort of 5.82 million subjects [26]. However, this positive association was not observed for alcohol drinking. From a study of patients with Hodgkin lymphoma diagnosed between age 16 and 69 years in England, no associations between alcohol consumption and Hodgkin lymphoma were observed [27].

The increasing Hodgkin lymphoma incidence could be attributable to a better diagnostic capacity as well as an increasing rate of relevant risk factors. The more marked increase in Hodgkin lymphoma incidence among female subjects, younger population, and Asian countries could be due to the difference in the increasing trend of obesity and metabolic diseases. For example, there was a global increase in female overweight/obesity from 30% in 1980 to 38% in 2013 [28]. The prevalence of metabolic syndrome increased more rapidly in females (from 7.9 to 30.7%) than in men (9.4–27.2%) in the past three decades [29]. From 1985 to 2014, younger subjects aged 15–40 years showed a more evident increase in prevalence of central obesity (16.3–33.9%) than the older population (43.6–57.9%) [30]. The proportion of obese females increased faster in Asian countries than the global average [31]. By contrast, there was an overall decreasing Hodgkin lymphoma mortality for the past decade, which could be driven by the better advances, availability, and accessibility of treatment. Nevertheless, we observed increasing Hodgkin lymphoma mortality in Chile and Ireland although the trend was not significant after excluding some single years for Ireland. The reasons remained unexplored and may require further investigation. 

### Limitations

There are some limitations to this study. First, because of the poor infrastructure and cancer reporting mechanism in developing countries, there could be underestimations of the incidence and mortality of Hodgkin lymphoma in those countries. Also, overestimation could be a problem for some countries as the numbers were represented by the cancer registries of the major cities. Secondly, the risk factors association analysis was conducted at a population level. The findings may not apply to individuals. Besides, other important risk factors for Hodgkin lymphoma had not been considered in the analysis, including prevalence of Epstein-Barr virus infection, HIV/AIDS, and autoimmune diseases. Although comparisons of Hodgkin lymphoma incidence and mortality were made within the same regions according to age and sex group in this study, it could be challenging to directly compare different countries from the cancer registry that might change over time. Fourthly, some countries did not provide publicly available data on mortality although incidence data were reported, which have limited the analysis on relationship between incidence and mortality. In addition, trend analysis on subtypes of Hodgkin lymphoma was not performed due to data constraints. The disease distribution, risk factors, and temporal trends could be different among different subtypes of Hodgkin lymphoma, which could provide further insight into preventive measures of the disease.

## Conclusions

For the past decade, the overall Hodgkin lymphoma incidence rate has been increasing, especially among subjects who were female, younger population, and from Asian countries. The drivers for this trend remain unknown, but might be attributable to the increasing prevalence of lifestyle and metabolic risk factors as well as improvements in early diagnosis. The Hodgkin lymphoma mortality has been decreasing probably due to the early cancer diagnosis and advances in treatment. However, increasing Hodgkin lymphoma mortality rates were noted in Chile and Ireland. Further studies are needed to investigate the reasons for these epidemiologic trends and have a better understanding of the particular aetiology and prognosis of Hodgkin lymphoma.

## Supplementary Information

**Additional file 1. Fig. S1**: Associations between risk factors and Hodgkin lymphoma incidence by age. **Fig. S2**: Associations between risk factors and Hodgkin lymphoma mortality by age. **Fig. S3**: Incidence and mortality trends for individual countries. **Fig. S4**: Results of joinpoint regression for individual countries. **Fig. S5**: AAPC of Hodgkin lymphoma incidence aged 50 years and older. **Fig. S6**: AAPC of Hodgkin lymphoma incidence aged < 50 years old. **Fig. S7**: AAPC of Hodgkin lymphoma incidence aged < 40 years old.

**Additional file 2. Table S1**: Data sources for trend analysis. **Table S2**: Global incidence and mortality of Hodgkin lymphoma in 2020 by region, sex, and HDI. **Table S3**: Sensitivity analysis for mortality trend in Chile and Ireland.

## Data Availability

The data used for the analyses are publicly available from the WHO websites (https://gco.iarc.fr/; https://apps.who.int/gho/data/node.main).
