# Supplementary material for: Incidence, mortality, risk factors, and trends for Hodgkin lymphoma: a global data analysis
Source: J Hematol Oncol. 2022 May 11;15:57. doi: 10.1186/s13045-022-01281-9 (PMC9097358; doi:10.1186/s13045-022-01281-9)
Supplement: Supplementary file 1 — Additional file 1. Fig. S1: Associations between risk factors and Hodgkin lymphoma incidence by age. Fig. S2: Associations between risk factors and Hodgkin lymphoma mortality by age. Fig. S3: Incidence and mortality trends for individual countries. Fig. S4: Results of joinpoint regression for individual countries. Fig. S5: AAPC of Hodgkin lymphoma incidence aged 50 years and older. Fig. S6: AAPC of Hodgkin lymphoma incidence aged < 50 years old. Fig. S7: AAPC of Hodgkin lymphoma incidence aged < 40 years old. [file 13045_2022_1281_MOESM1_ESM.pdf]

Supplementary Figures Legends

Supplementary Figure 1. Associations between risk factors and Hodgkin lymphoma incidence by age

Supplementary Figure 2. Associations between risk factors and Hodgkin lymphoma mortality by age

Supplementary Figure 3. Incidence and mortality trends for individual countries

Supplementary Figure 4. Results of joinpoint regression for individual countries

Supplementary Figure 5. AAPC of Hodgkin lymphoma incidence aged 50 years and older

Supplementary Figure 6. AAPC of Hodgkin lymphoma incidence aged < 50 years old

Supplementary Figure 7. AAPC of Hodgkin lymphoma incidence aged < 40 years old

Supplementary Figure 1. Associations between risk factors and Hodgkin lymphoma incidence by age

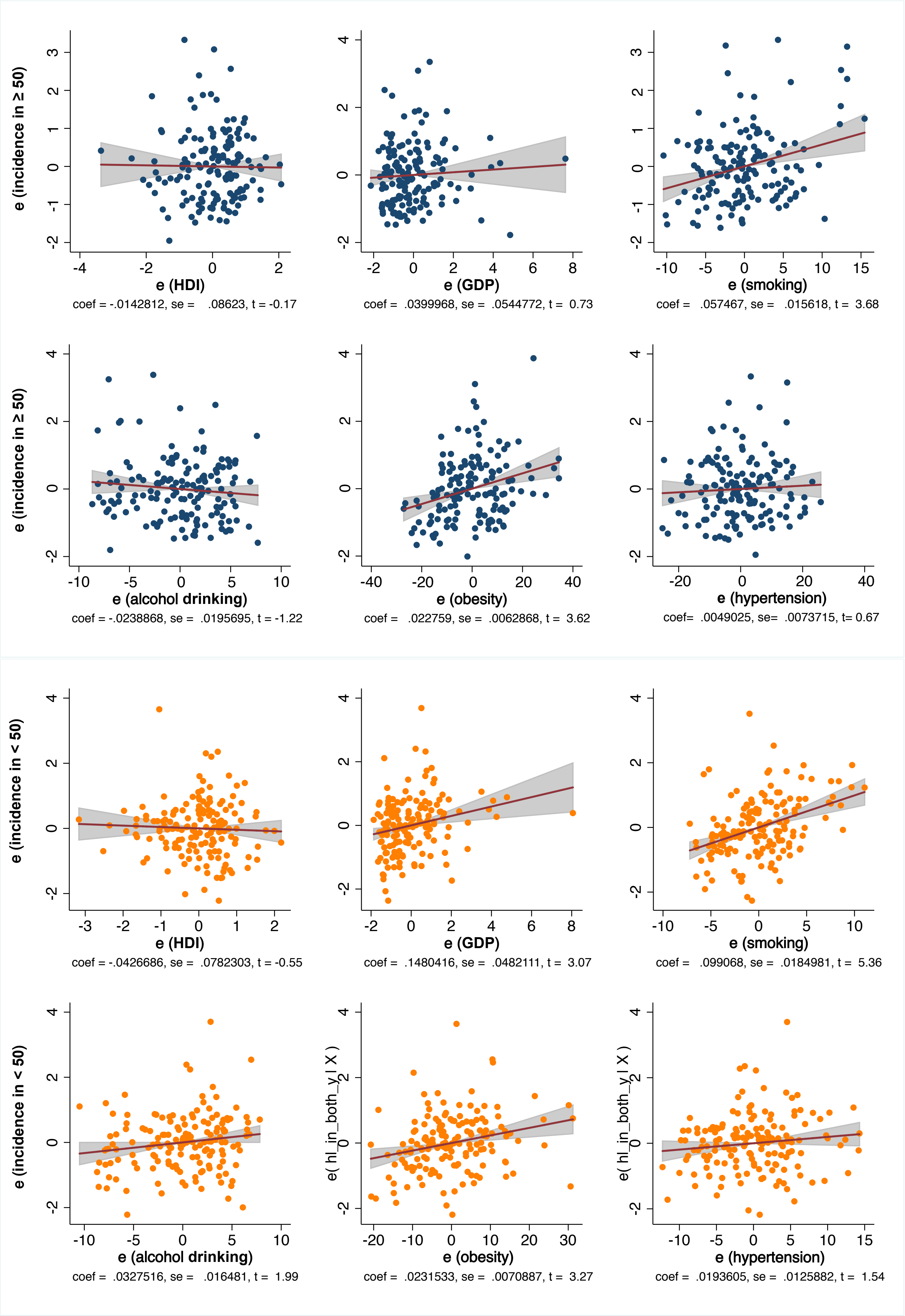

The added-variable plots were generated using multi-variable linear regression models. e, residuals; HDI, human development index; GDP, gross domestic products

Supplementary Figure 2. Associations between risk factors and Hodgkin lymphoma mortality by age

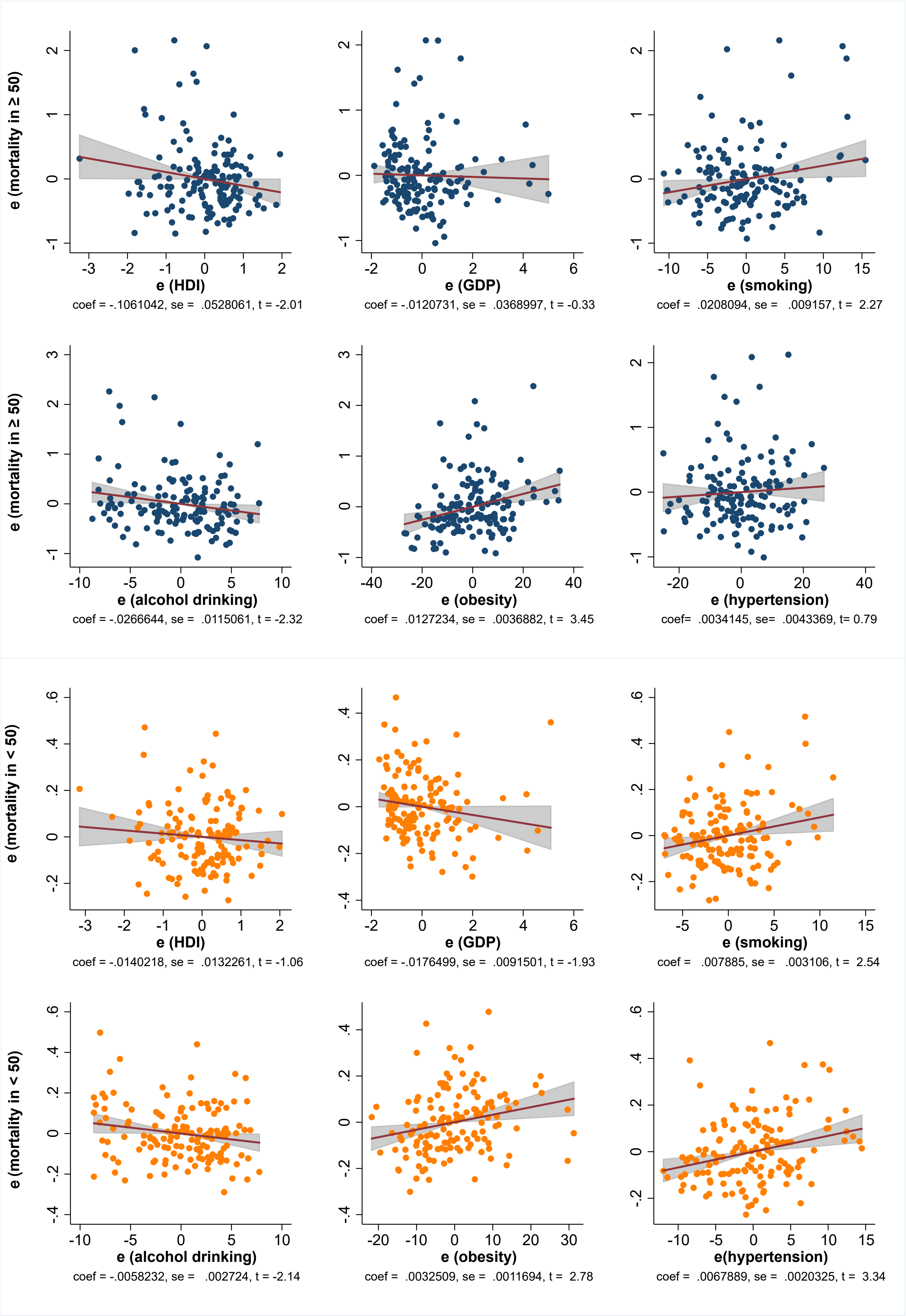

The added-variable plots were generated using multi-variable linear regression models. e, residuals; HDI, human development index; GDP, gross domestic products

Supplementary Figure 3. Incidence and mortality trends for individual countries

Male

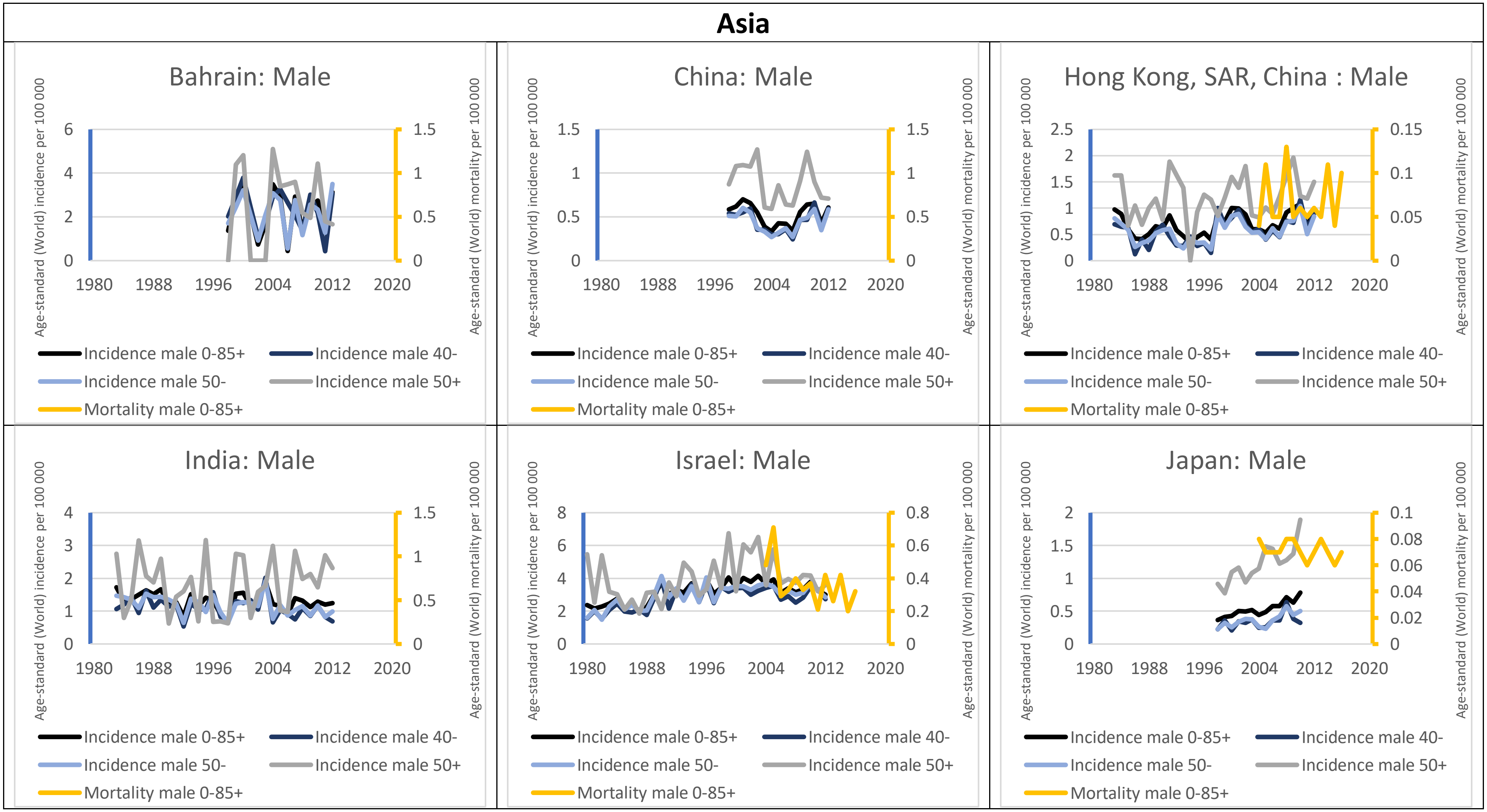

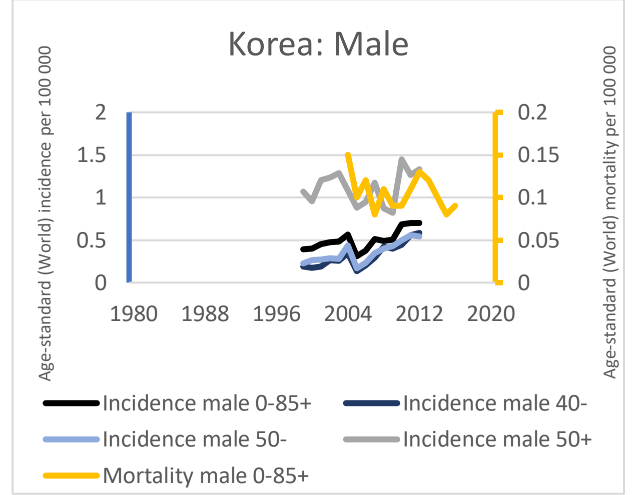
Korea: Male
Age-standard (World) incidence per 100 000
Age-standard (World) mortality per 100 000
2
1.5
1
0.5
0
0.2
0.15
0.1
0.05
0
1980
1988
1996
2004
2012
2020
Incidence male 0-85+
Incidence male 40-
Incidence male 50-
Incidence male 50+
Mortality male 0-85+

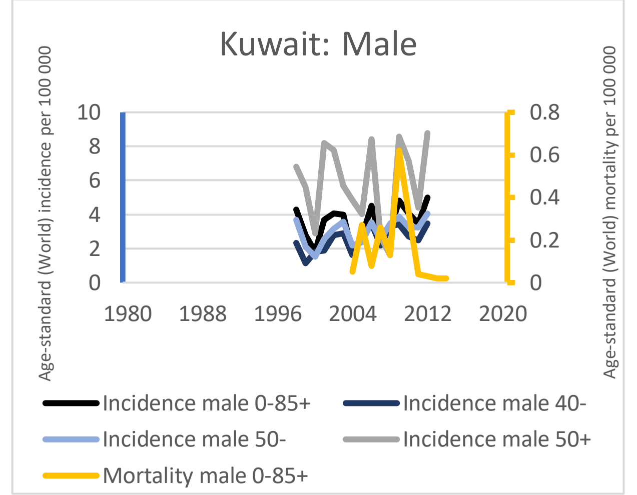
Kuwait: Male
Age-standard (World) incidence per 100 000
Age-standard (World) mortality per 100 000
10
8
6
4
2
0
0.8
0.6
0.4
0.2
0
1980
1988
1996
2004
2012
2020
Incidence male 0-85+
Incidence male 40-
Incidence male 50-
Incidence male 50+
Mortality male 0-85+

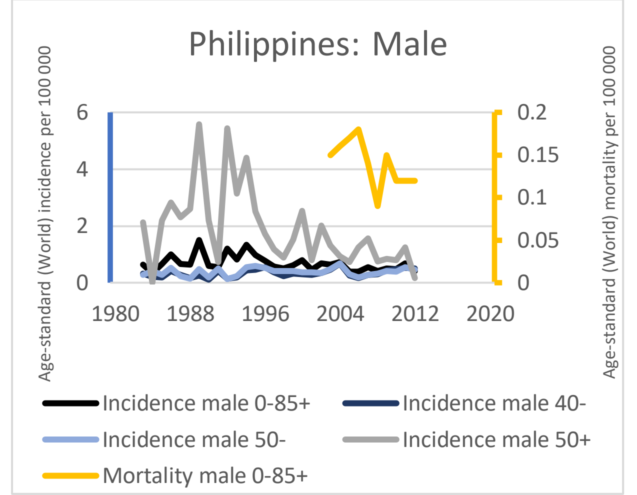
Philippines: Male
Age-standard (World) incidence per 100 000
Age-standard (World) mortality per 100 000
6
4
2
0
0.2
0.15
0.1
0.05
0
1980
1988
1996
2004
2012
2020
Incidence male 0-85+
Incidence male 40-
Incidence male 50-
Incidence male 50+
Mortality male 0-85+

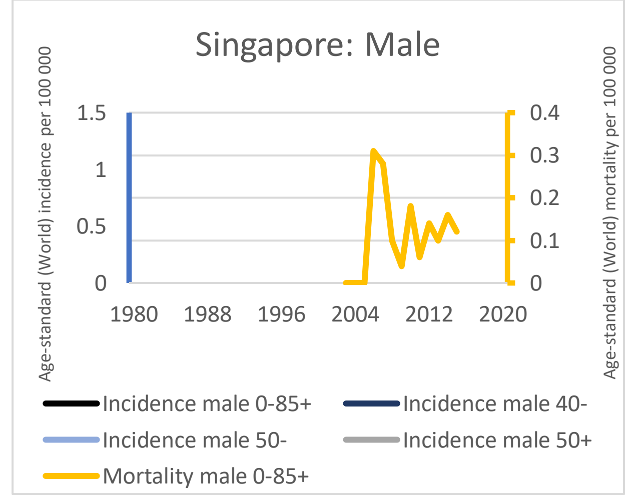
Singapore: Male
Age-standard (World) incidence per 100 000
Age-standard (World) mortality per 100 000
1.5
1
0.5
0
0.4
0.3
0.2
0.1
0
1980
1988
1996
2004
2012
2020
Incidence male 0-85+
Incidence male 40-
Incidence male 50-
Incidence male 50+
Mortality male 0-85+

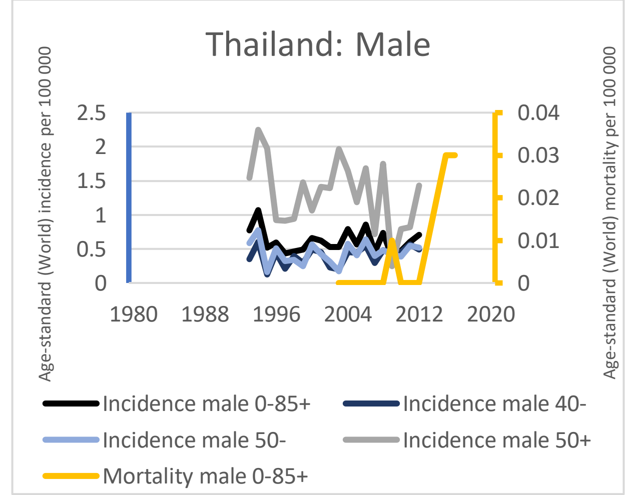
Thailand: Male
Age-standard (World) incidence per 100 000
Age-standard (World) mortality per 100 000
2.5
2
1.5
1
0.5
0
0.04
0.03
0.02
0.01
0
1980
1988
1996
2004
2012
2020
Incidence male 0-85+
Incidence male 40-
Incidence male 50-
Incidence male 50+
Mortality male 0-85+

Oceania

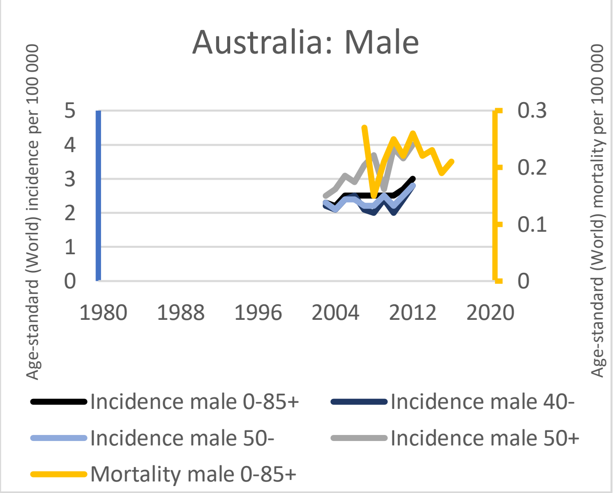

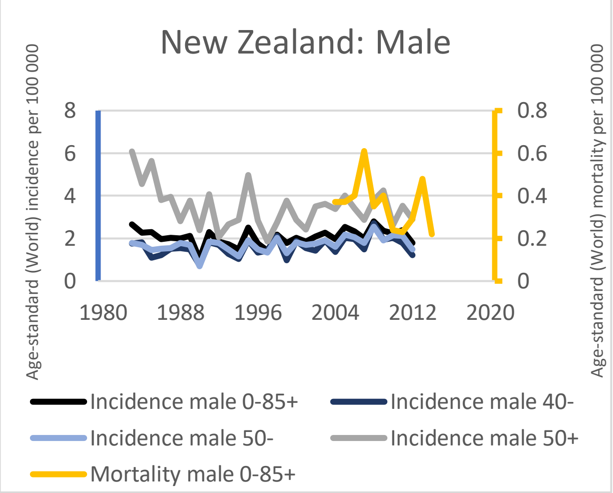

North America

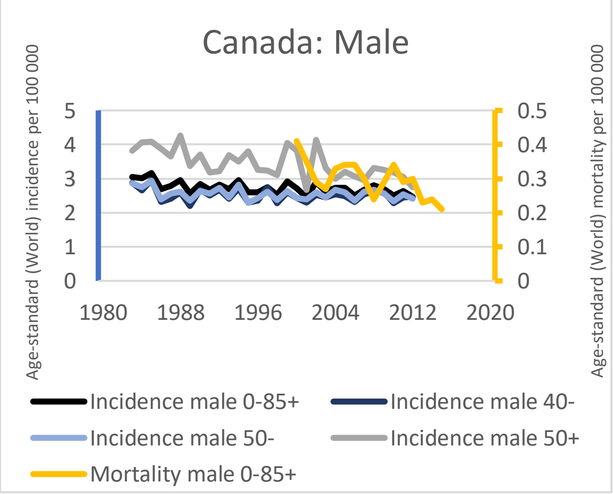

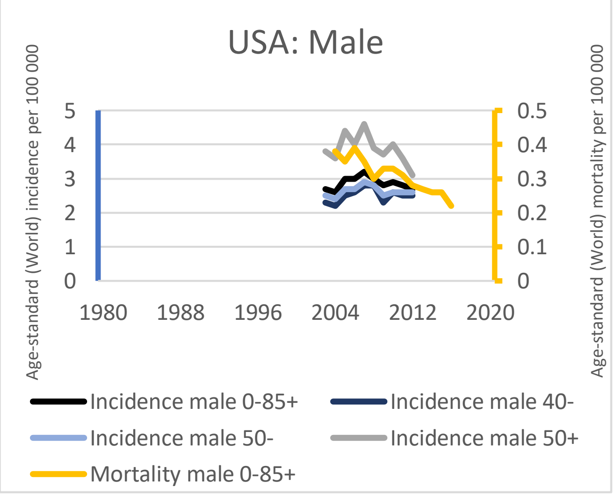

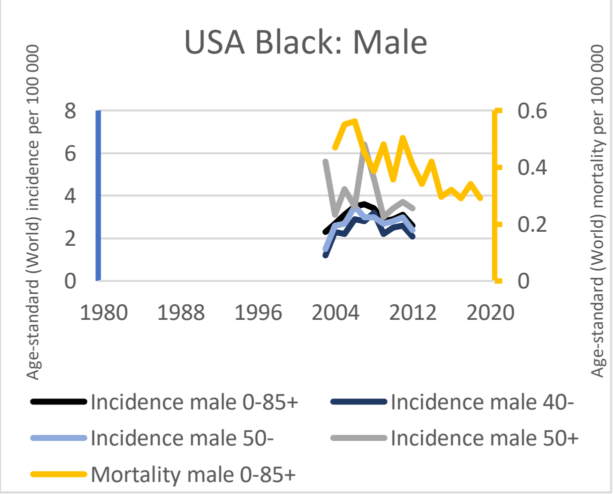

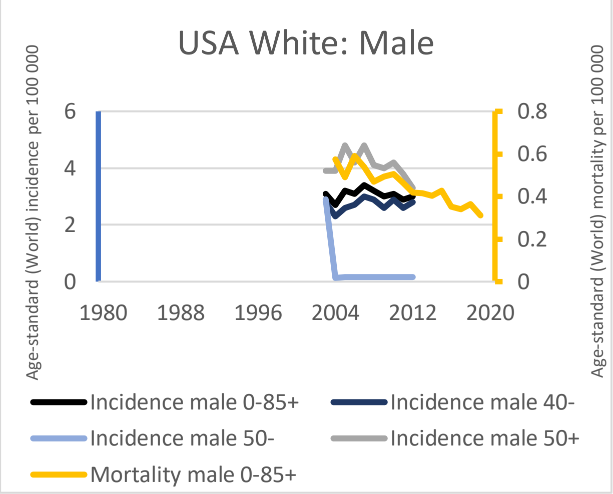

South America

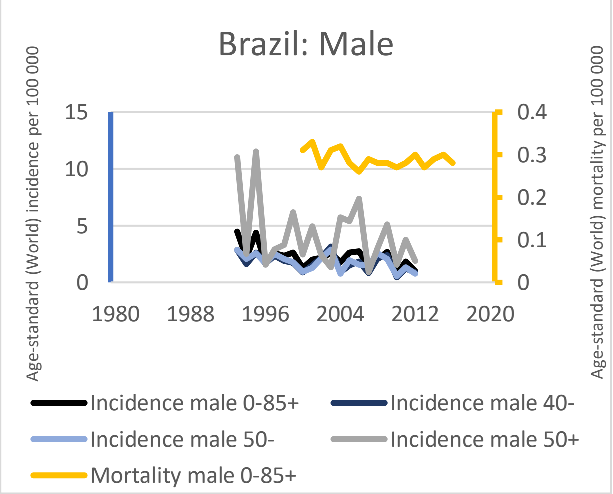

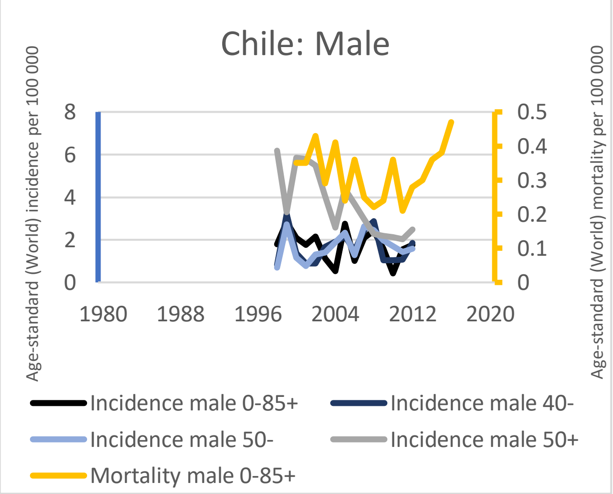

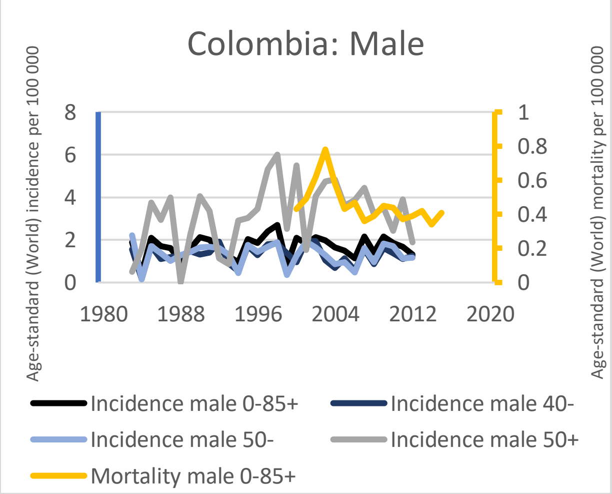

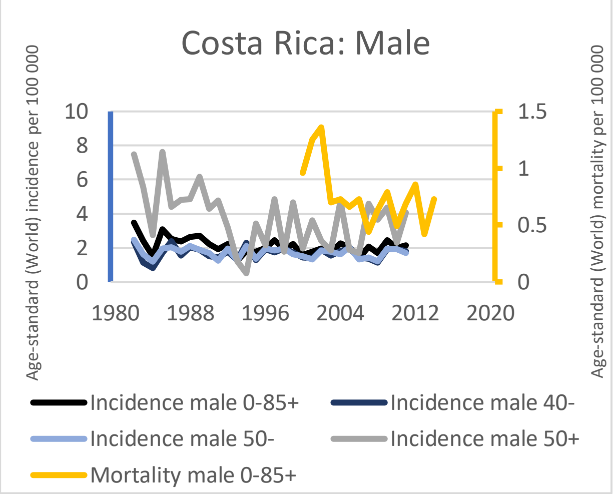

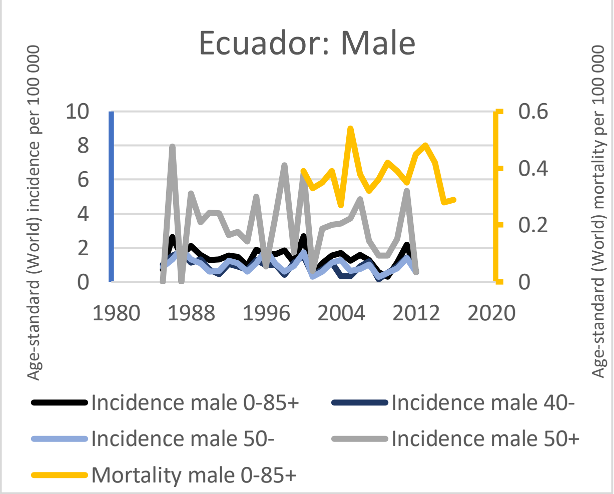

Northern Europe

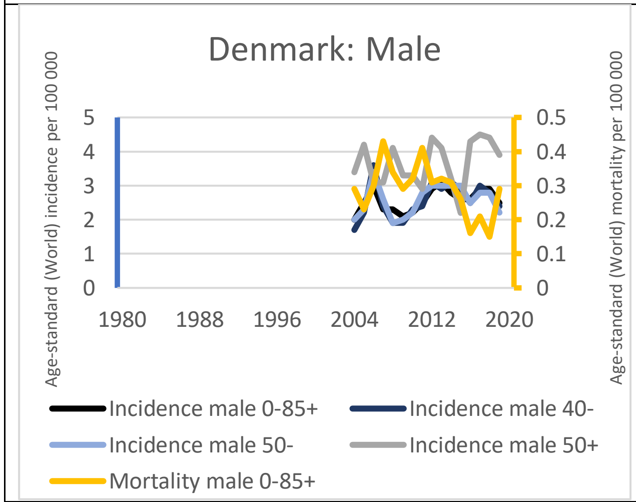

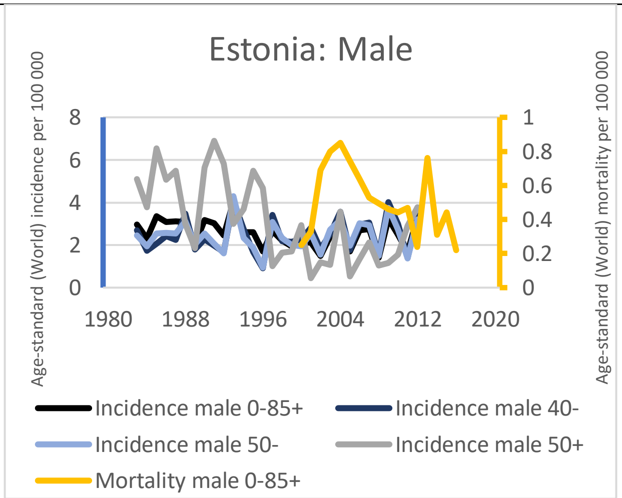

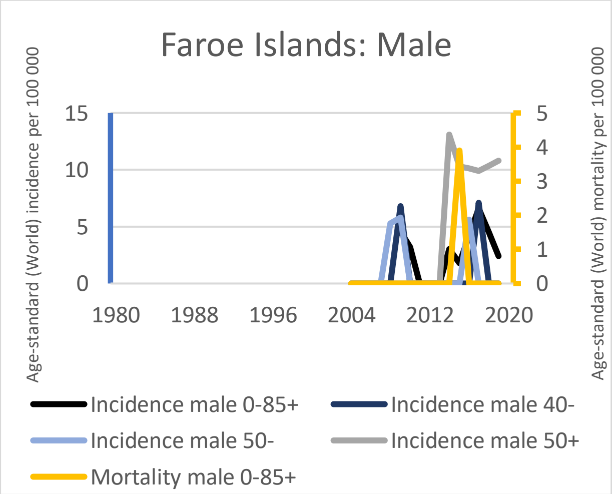

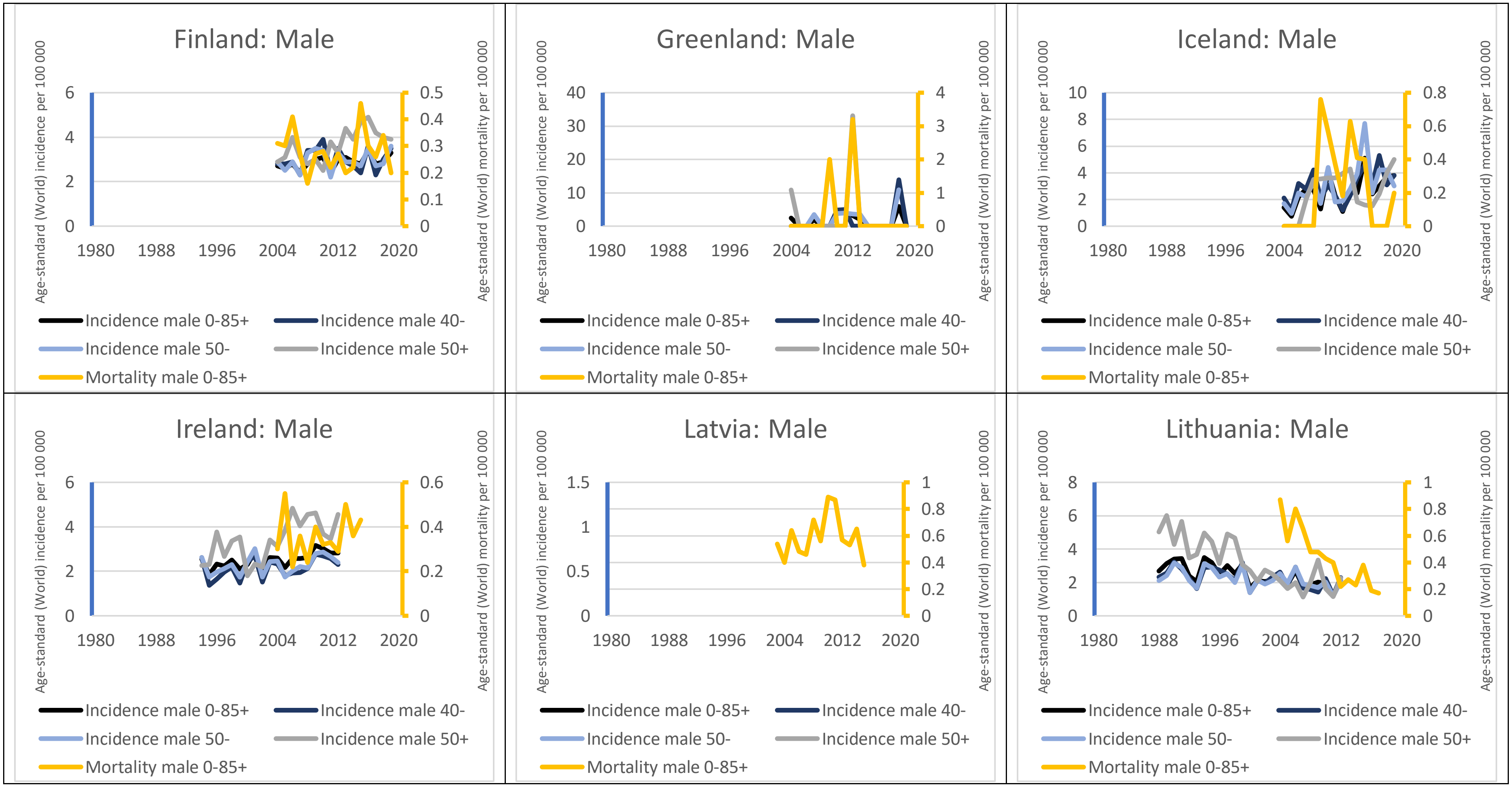
Finland: Male
Greenland: Male
Iceland: Male
Ireland: Male
Latvia: Male
Lithuania: Male
Age-standard (World) incidence per 100 000
Age-standard (World) mortality per 100 000
1980
1988
1996
2004
2012
2020
Incidence male 0-85+
Incidence male 40-
Incidence male 50-
Incidence male 50+
Mortality male 0-85+

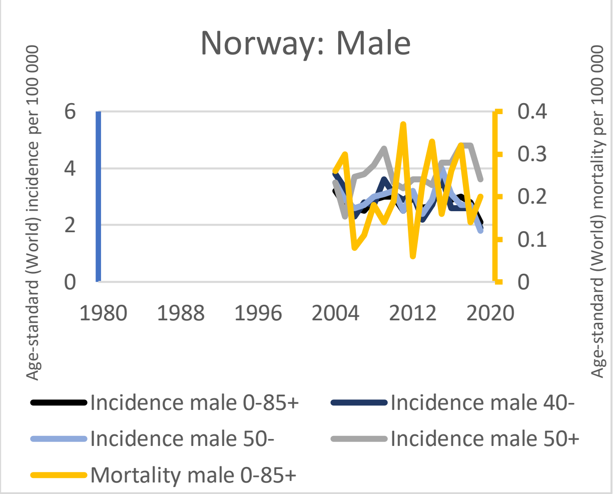

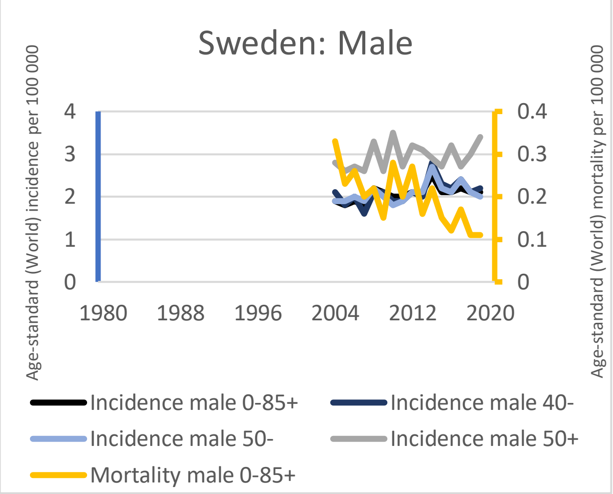

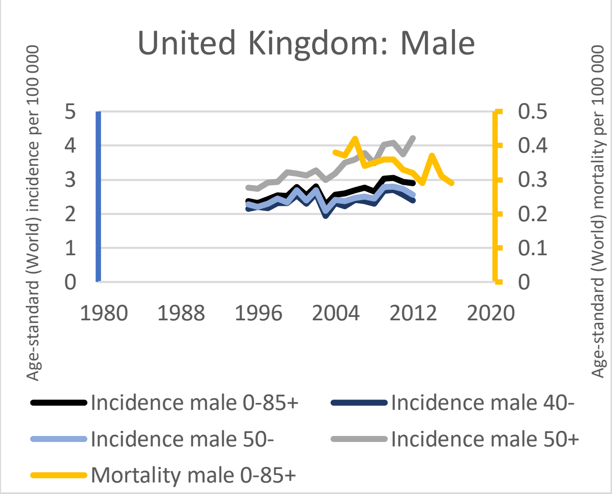

Western Europe

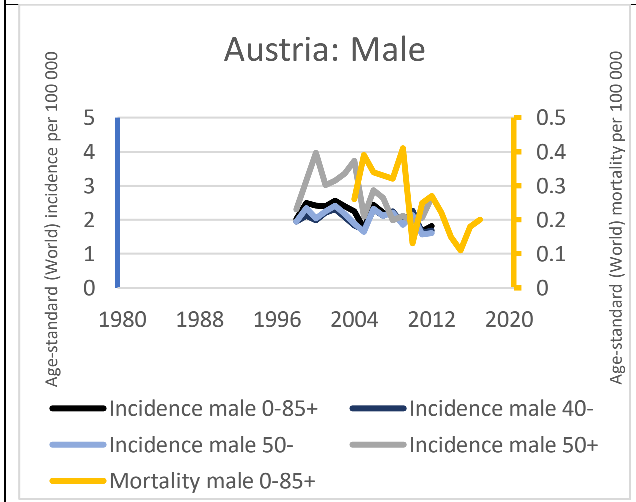

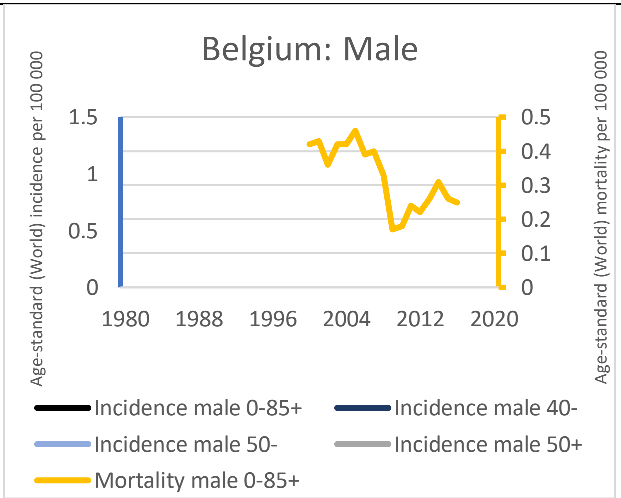

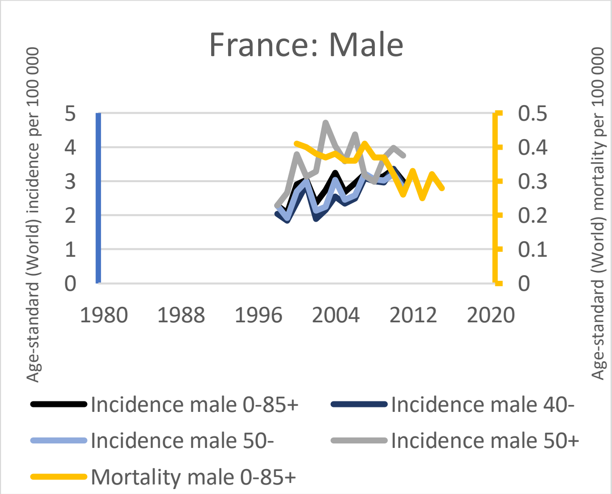

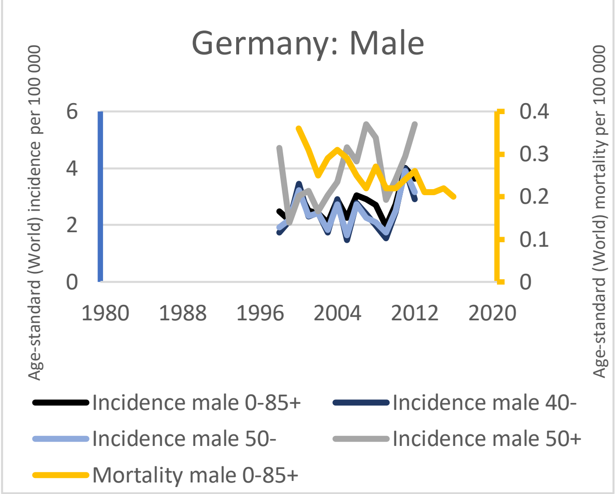

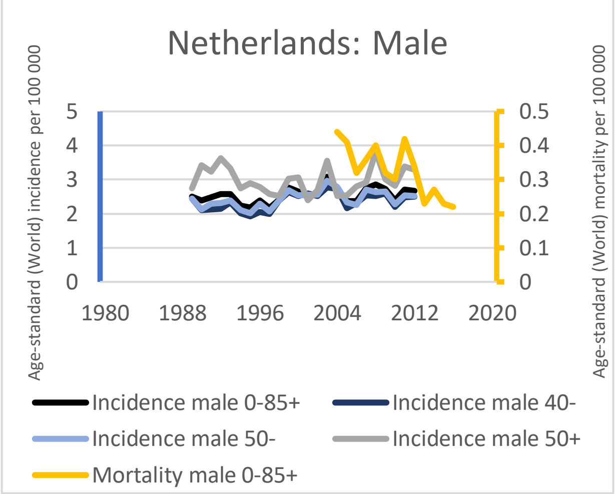

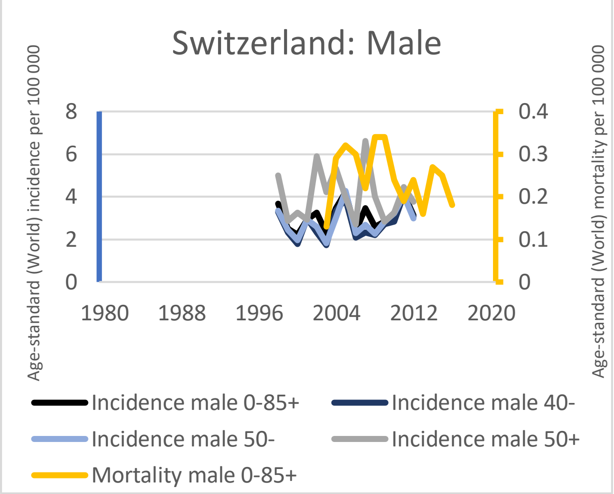

Southern Europe

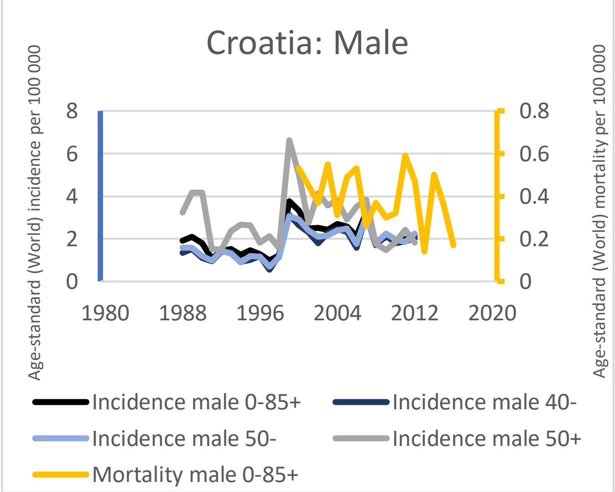

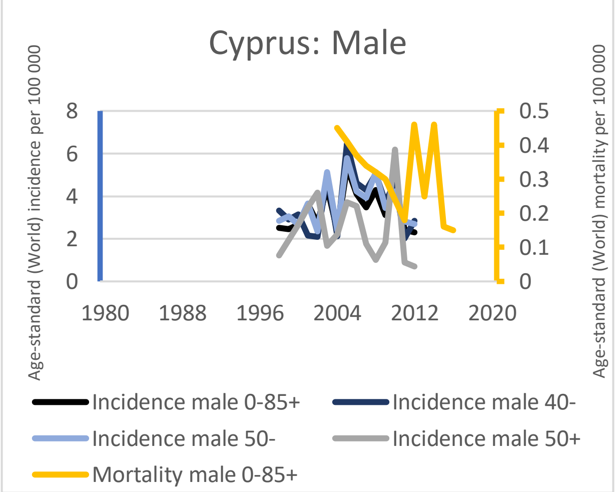

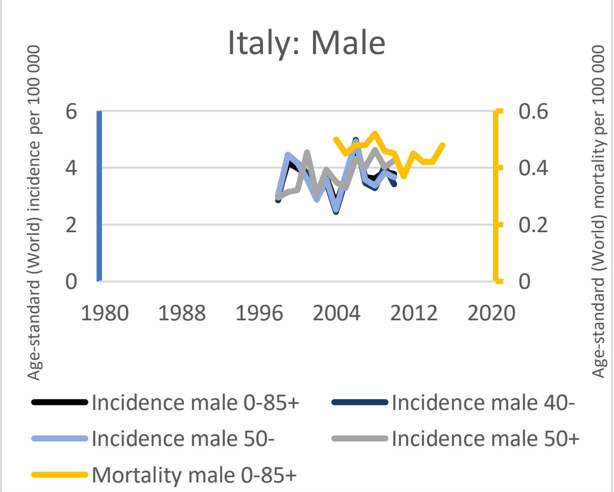

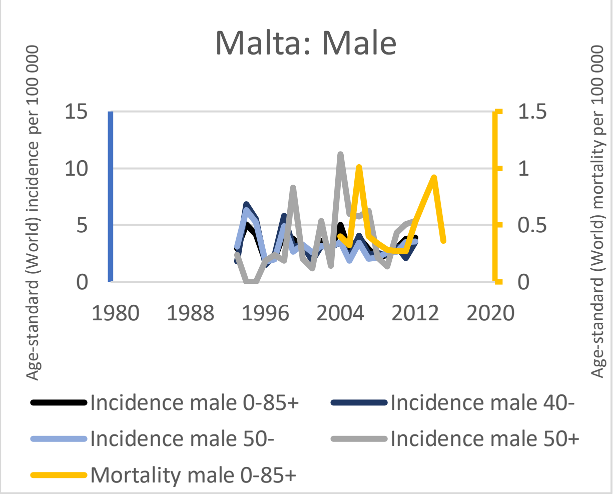
Malta: Male
Age-standard (World) incidence per 100 000
Age-standard (World) mortality per 100 000
15
10
5
0
1.5
1
0.5
0
1980
1988
1996
2004
2012
2020
Incidence male 0-85+
Incidence male 40-
Incidence male 50-
Incidence male 50+
Mortality male 0-85+

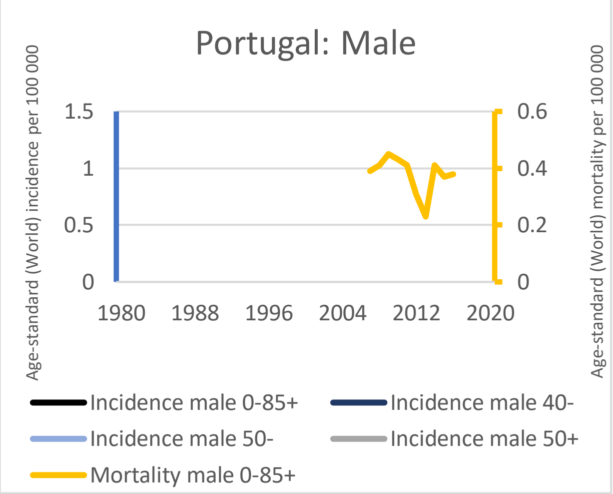
Portugal: Male
Age-standard (World) incidence per 100 000
Age-standard (World) mortality per 100 000
1.5
1
0.5
0
0.6
0.4
0.2
0
1980
1988
1996
2004
2012
2020
Incidence male 0-85+
Incidence male 40-
Incidence male 50-
Incidence male 50+
Mortality male 0-85+

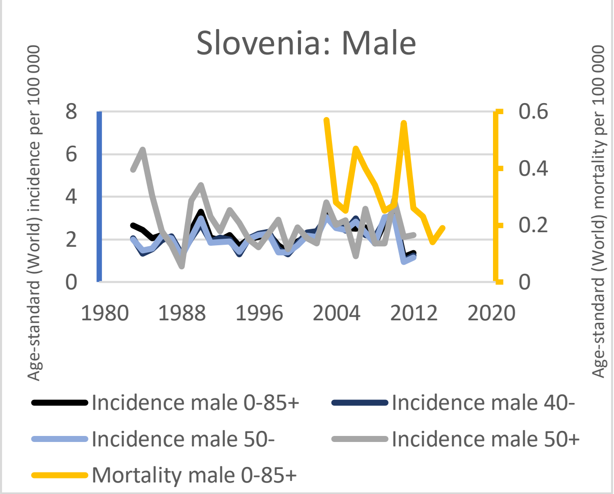
Slovenia: Male
Age-standard (World) incidence per 100 000
Age-standard (World) mortality per 100 000
8
6
4
2
0
0.6
0.4
0.2
0
1980
1988
1996
2004
2012
2020
Incidence male 0-85+
Incidence male 40-
Incidence male 50-
Incidence male 50+
Mortality male 0-85+

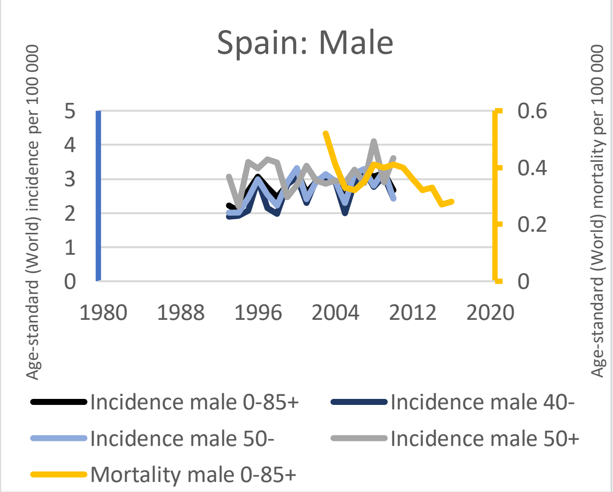
Spain: Male
Age-standard (World) incidence per 100 000
Age-standard (World) mortality per 100 000
5
4
3
2
1
0
0.6
0.4
0.2
0
1980
1988
1996
2004
2012
2020
Incidence male 0-85+
Incidence male 40-
Incidence male 50-
Incidence male 50+
Mortality male 0-85+

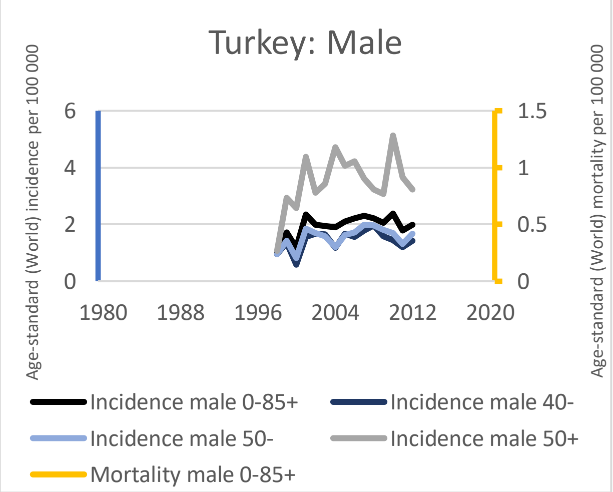
Turkey: Male
Age-standard (World) incidence per 100 000
Age-standard (World) mortality per 100 000
6
4
2
0
1.5
1
0.5
0
1980
1988
1996
2004
2012
2020
Incidence male 0-85+
Incidence male 40-
Incidence male 50-
Incidence male 50+
Mortality male 0-85+

Eastern Europe

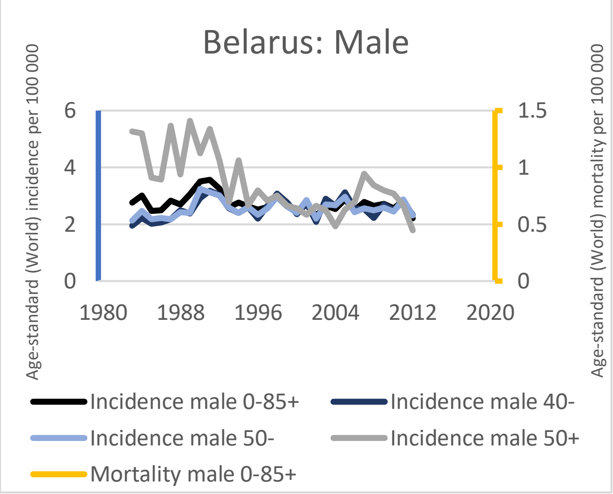

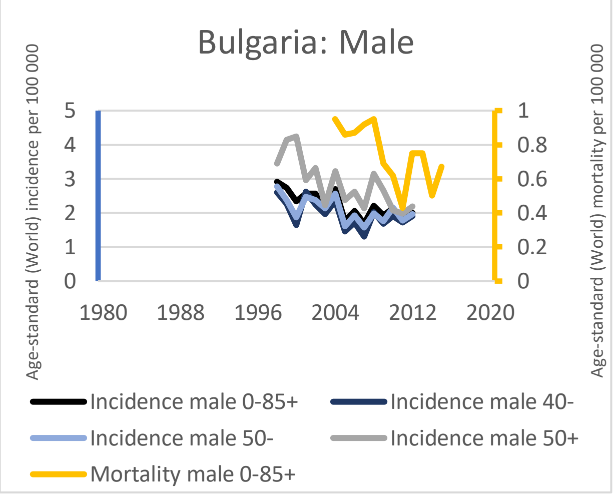

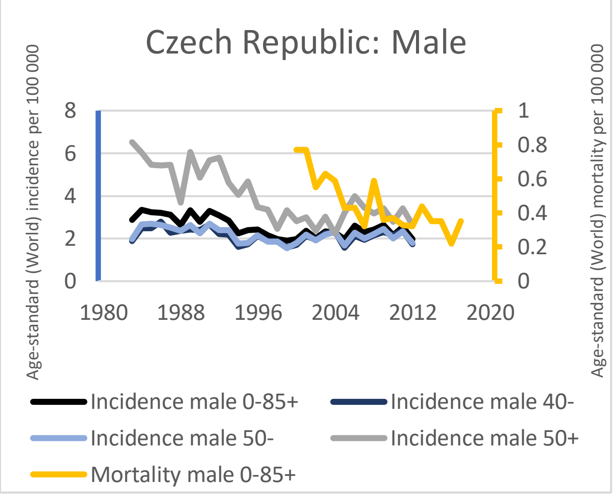

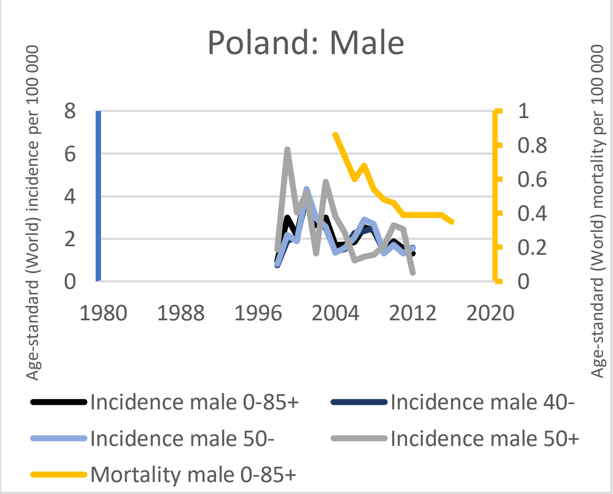

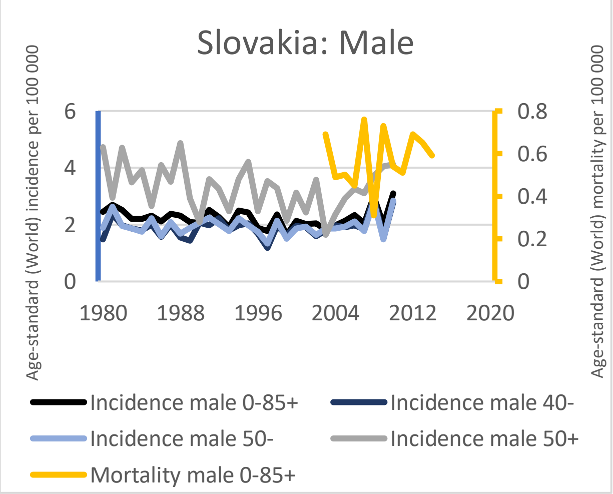

Africa		
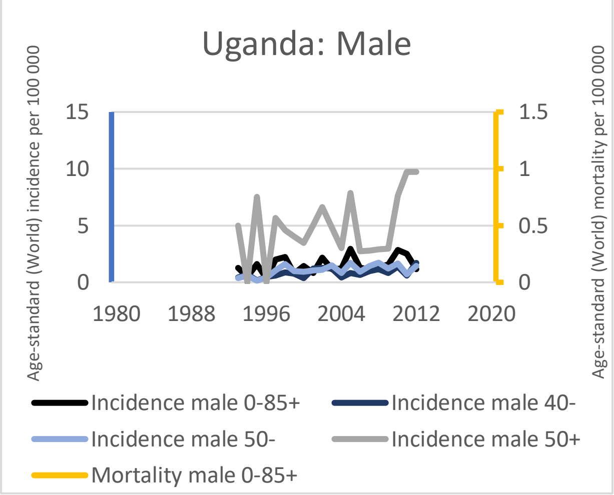Uganda: Male<br>Age-standard (World) incidence per 100 000<br>Age-standard (World) mortality per 100 000<br>15<br>10<br>5<br>0<br>1.5<br>1<br>0.5<br>0<br>1980<br>1988<br>1996<br>2004<br>2012<br>2020<br>Incidence male 0-85+<br>Incidence male 40-<br>Incidence male 50-<br>Incidence male 50+<br>Mortality male 0-85+		

Female

Asia

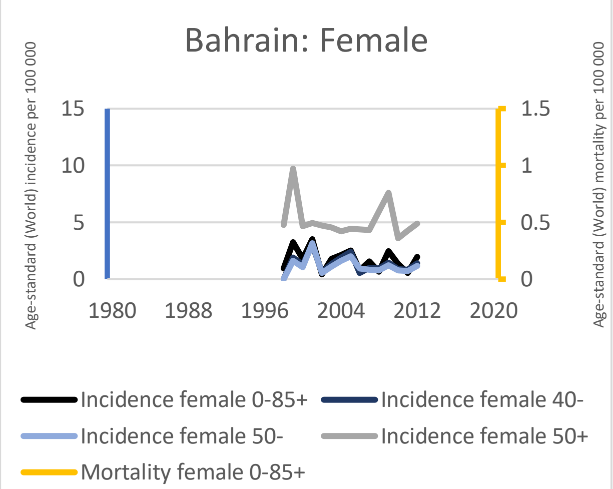
Bahrain: Female
Age-standard (World) incidence per 100 000
Age-standard (World) mortality per 100 000
15
10
5
0
1.5
1
0.5
0
1980
1988
1996
2004
2012
2020
Incidence female 0-85+
Incidence female 40-
Incidence female 50-
Incidence female 50+
Mortality female 0-85+

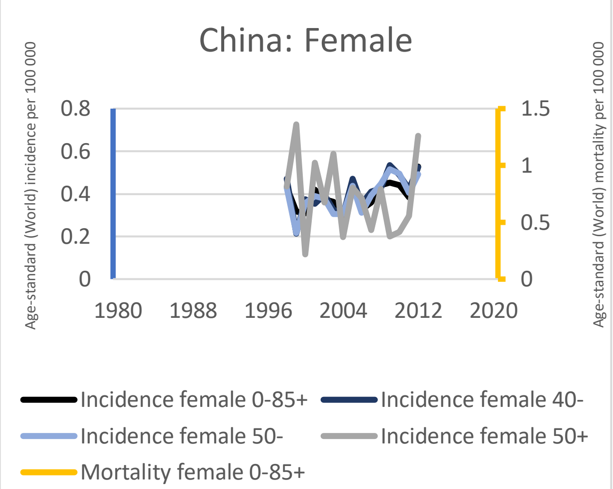
China: Female
Age-standard (World) incidence per 100 000
Age-standard (World) mortality per 100 000
0.8
0.6
0.4
0.2
0
1.5
1
0.5
0
1980
1988
1996
2004
2012
2020
Incidence female 0-85+
Incidence female 40-
Incidence female 50-
Incidence female 50+
Mortality female 0-85+

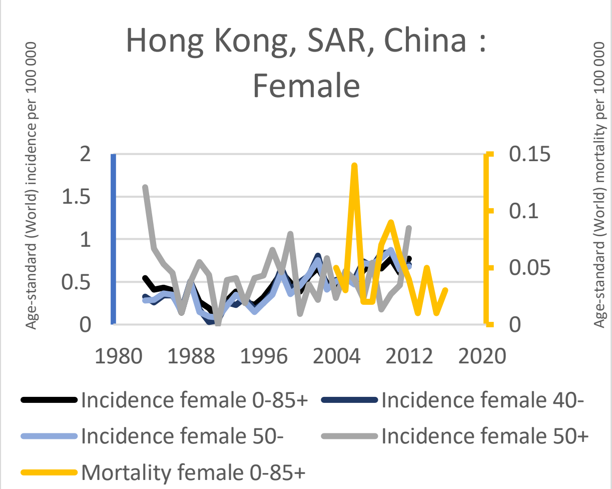
Hong Kong, SAR, China : Female
Age-standard (World) incidence per 100 000
Age-standard (World) mortality per 100 000
2
1.5
1
0.5
0
0.15
0.1
0.05
0
1980
1988
1996
2004
2012
2020
Incidence female 0-85+
Incidence female 40-
Incidence female 50-
Incidence female 50+
Mortality female 0-85+

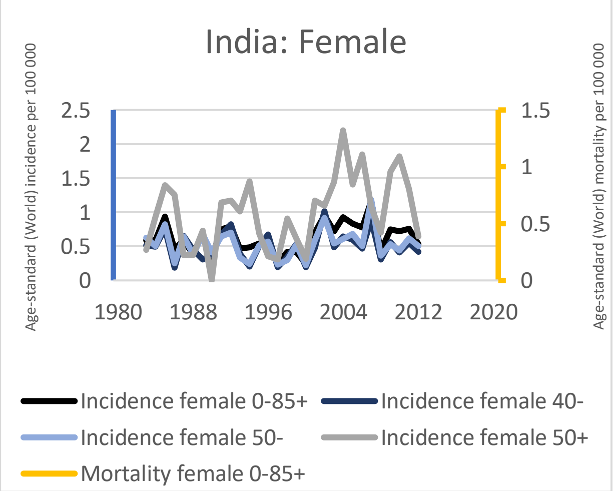
India: Female
Age-standard (World) incidence per 100 000
Age-standard (World) mortality per 100 000
2.5
2
1.5
1
0.5
0
1.5
1
0.5
0
1980
1988
1996
2004
2012
2020
Incidence female 0-85+
Incidence female 40-
Incidence female 50-
Incidence female 50+
Mortality female 0-85+

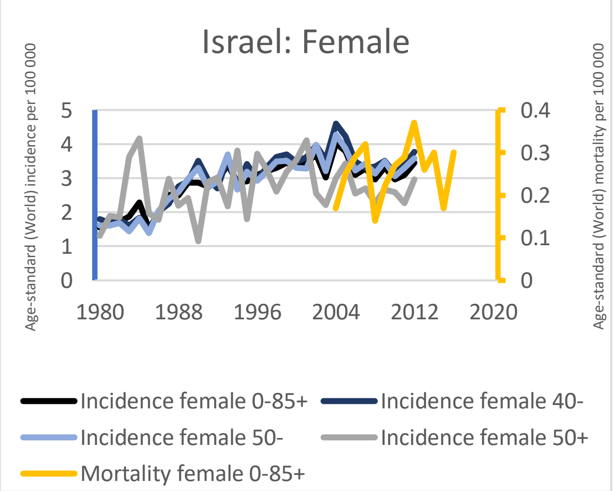
Israel: Female
Age-standard (World) incidence per 100 000
Age-standard (World) mortality per 100 000
5
4
3
2
1
0
0.4
0.3
0.2
0.1
0
1980
1988
1996
2004
2012
2020
Incidence female 0-85+
Incidence female 40-
Incidence female 50-
Incidence female 50+
Mortality female 0-85+

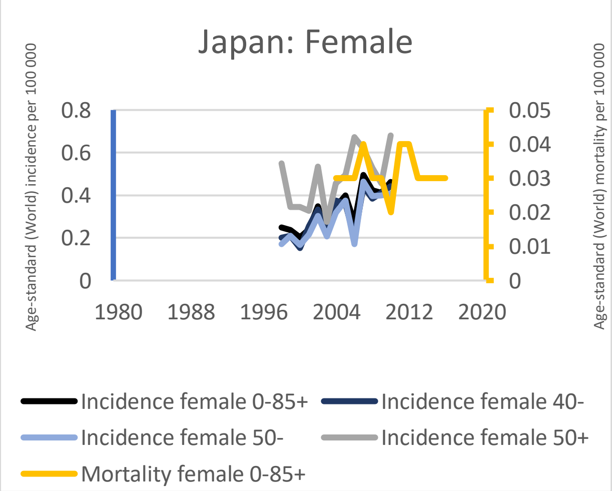
Japan: Female
Age-standard (World) incidence per 100 000
Age-standard (World) mortality per 100 000
0.8
0.6
0.4
0.2
0
0.05
0.04
0.03
0.02
0.01
0
1980
1988
1996
2004
2012
2020
Incidence female 0-85+
Incidence female 40-
Incidence female 50-
Incidence female 50+
Mortality female 0-85+

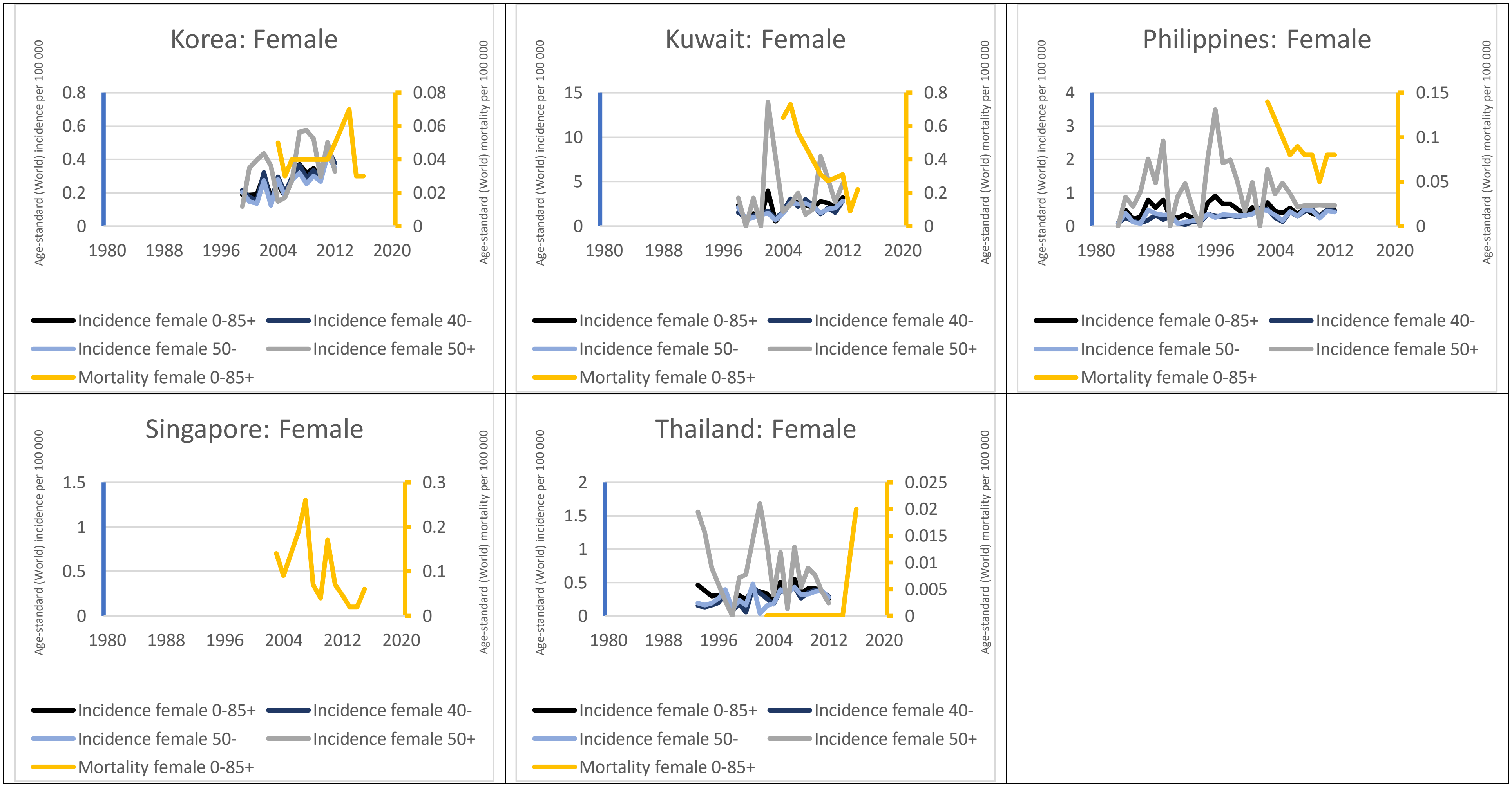

Korea: Female
Age-standard (World) incidence per 100 000
Age-standard (World) mortality per 100 000
0.8
0.6
0.4
0.2
0
0.08
0.06
0.04
0.02
0
1980 1988 1996 2004 2012 2020
Incidence female 0-85+
Incidence female 40-
Incidence female 50-
Incidence female 50+
Mortality female 0-85+
Kuwait: Female
Age-standard (World) incidence per 100 000
Age-standard (World) mortality per 100 000
15
10
5
0
0.8
0.6
0.4
0.2
0
1980 1988 1996 2004 2012 2020
Incidence female 0-85+
Incidence female 40-
Incidence female 50-
Incidence female 50+
Mortality female 0-85+
Philippines: Female
Age-standard (World) incidence per 100 000
Age-standard (World) mortality per 100 000
4
3
2
1
0
0.15
0.1
0.05
0
1980 1988 1996 2004 2012 2020
Incidence female 0-85+
Incidence female 40-
Incidence female 50-
Incidence female 50+
Mortality female 0-85+
Singapore: Female
Age-standard (World) incidence per 100 000
Age-standard (World) mortality per 100 000
1.5
1
0.5
0
0.3
0.2
0.1
0
1980 1988 1996 2004 2012 2020
Incidence female 0-85+
Incidence female 40-
Incidence female 50-
Incidence female 50+
Mortality female 0-85+
Thailand: Female
Age-standard (World) incidence per 100 000
Age-standard (World) mortality per 100 000
2
1.5
1
0.5
0
0.025
0.02
0.015
0.01
0.005
0
1980 1988 1996 2004 2012 2020
Incidence female 0-85+
Incidence female 40-
Incidence female 50-
Incidence female 50+
Mortality female 0-85+

Oceania

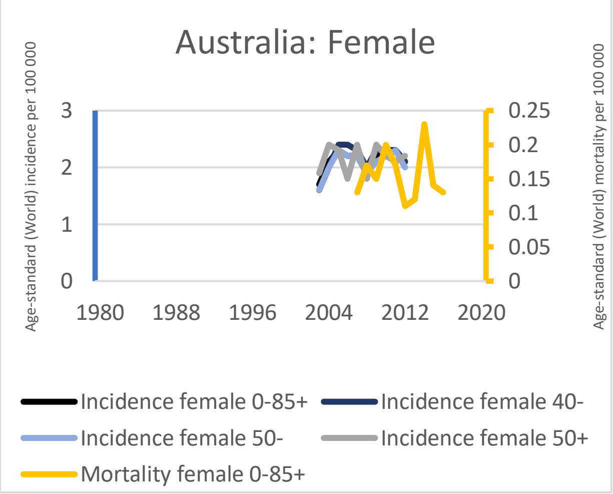

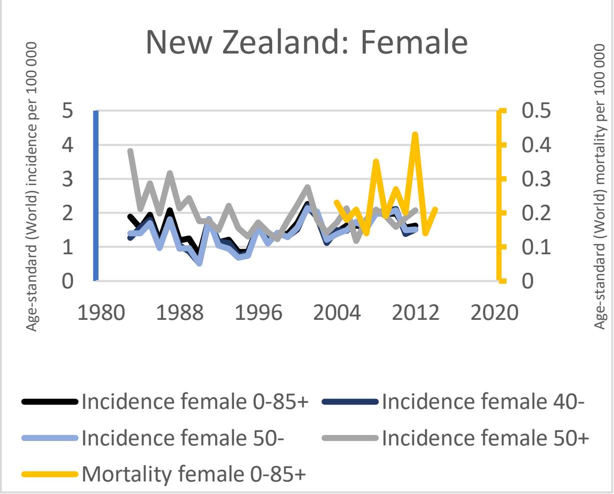

North America

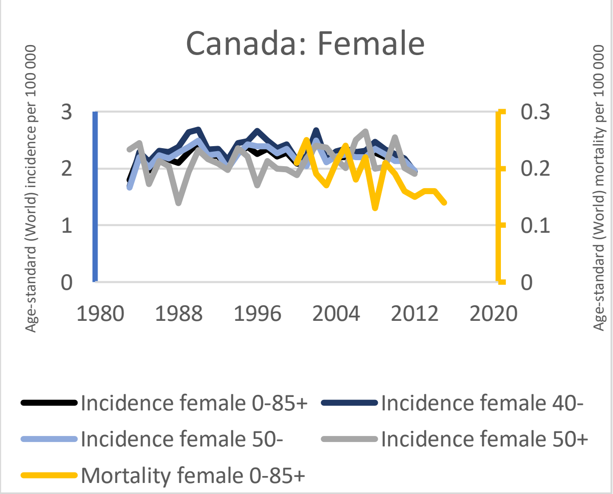

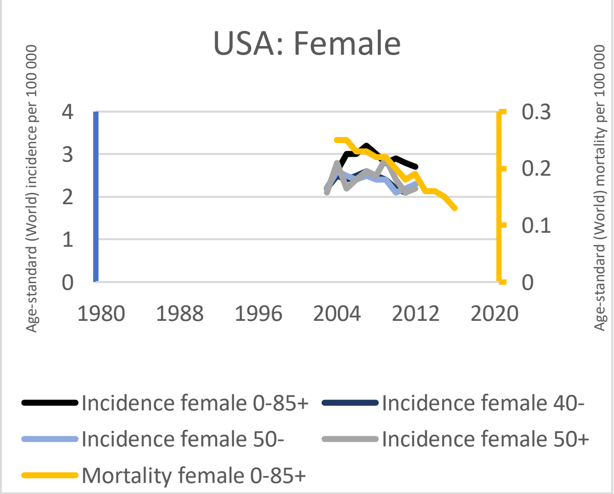

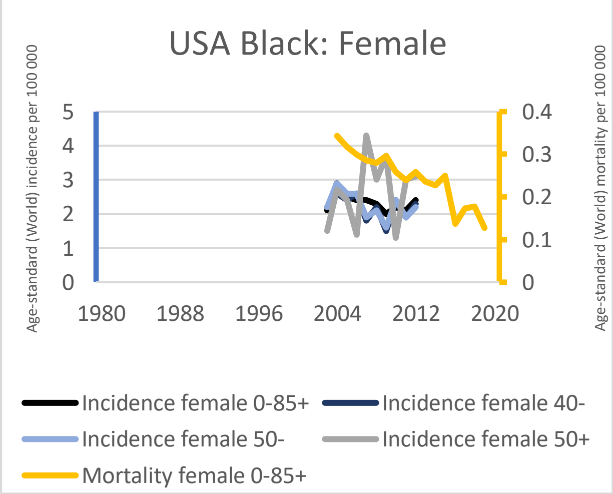

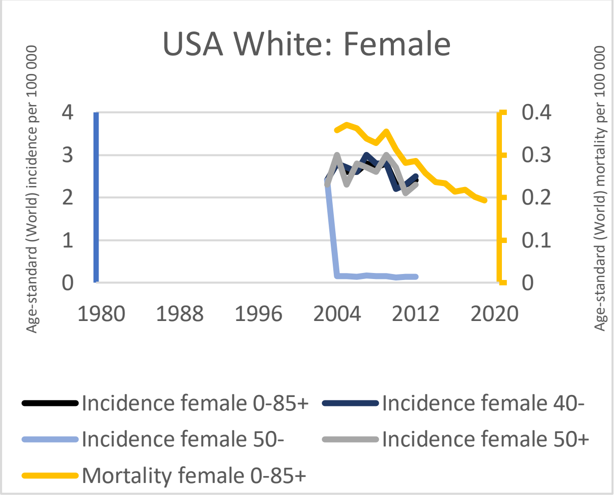

South America

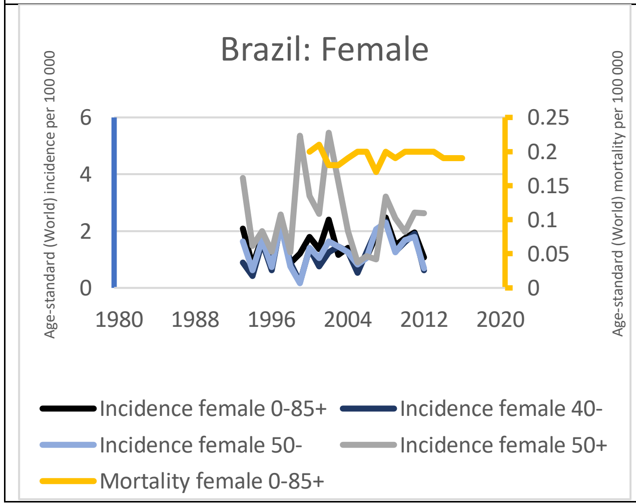

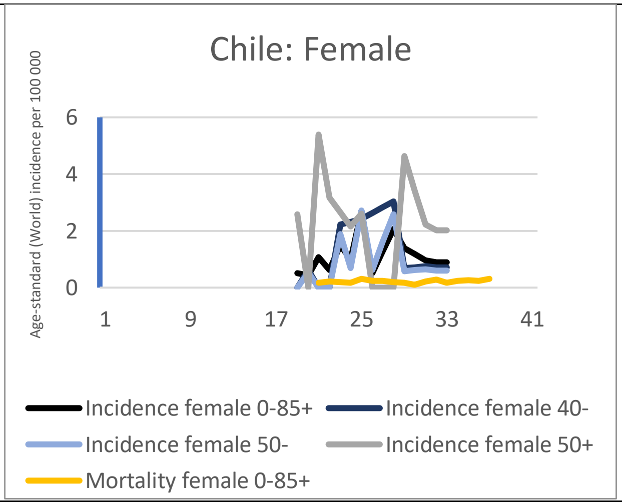

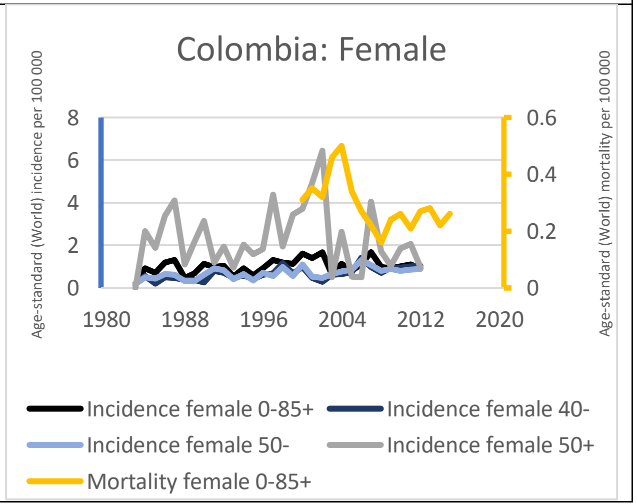

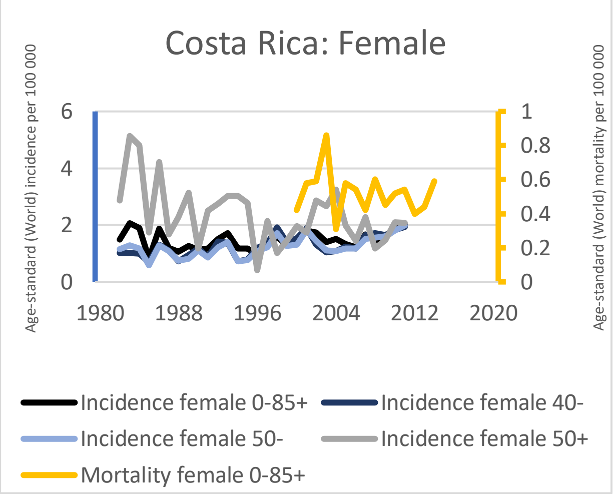

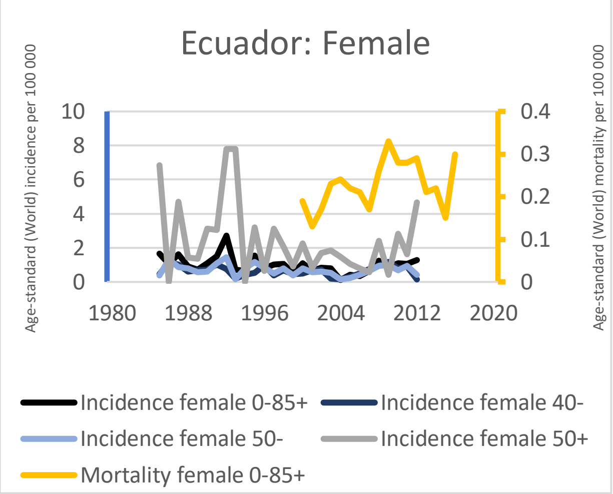

Northern Europe

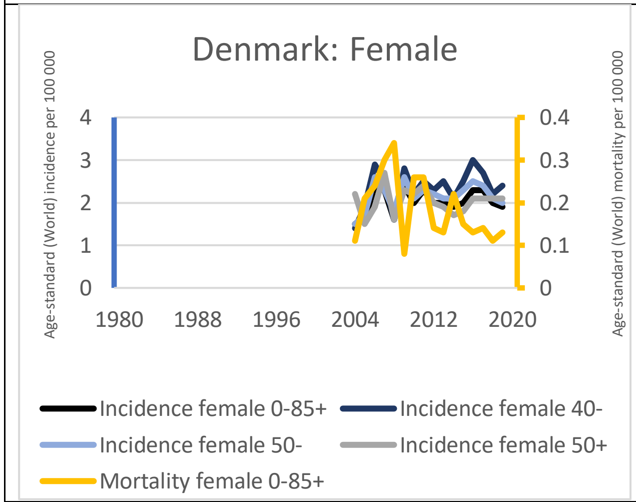

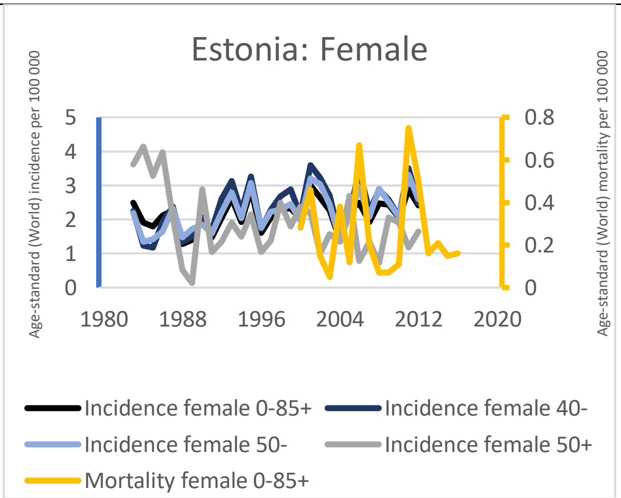

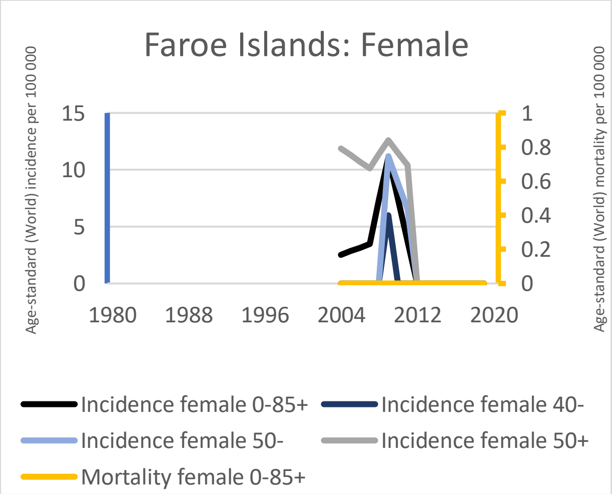

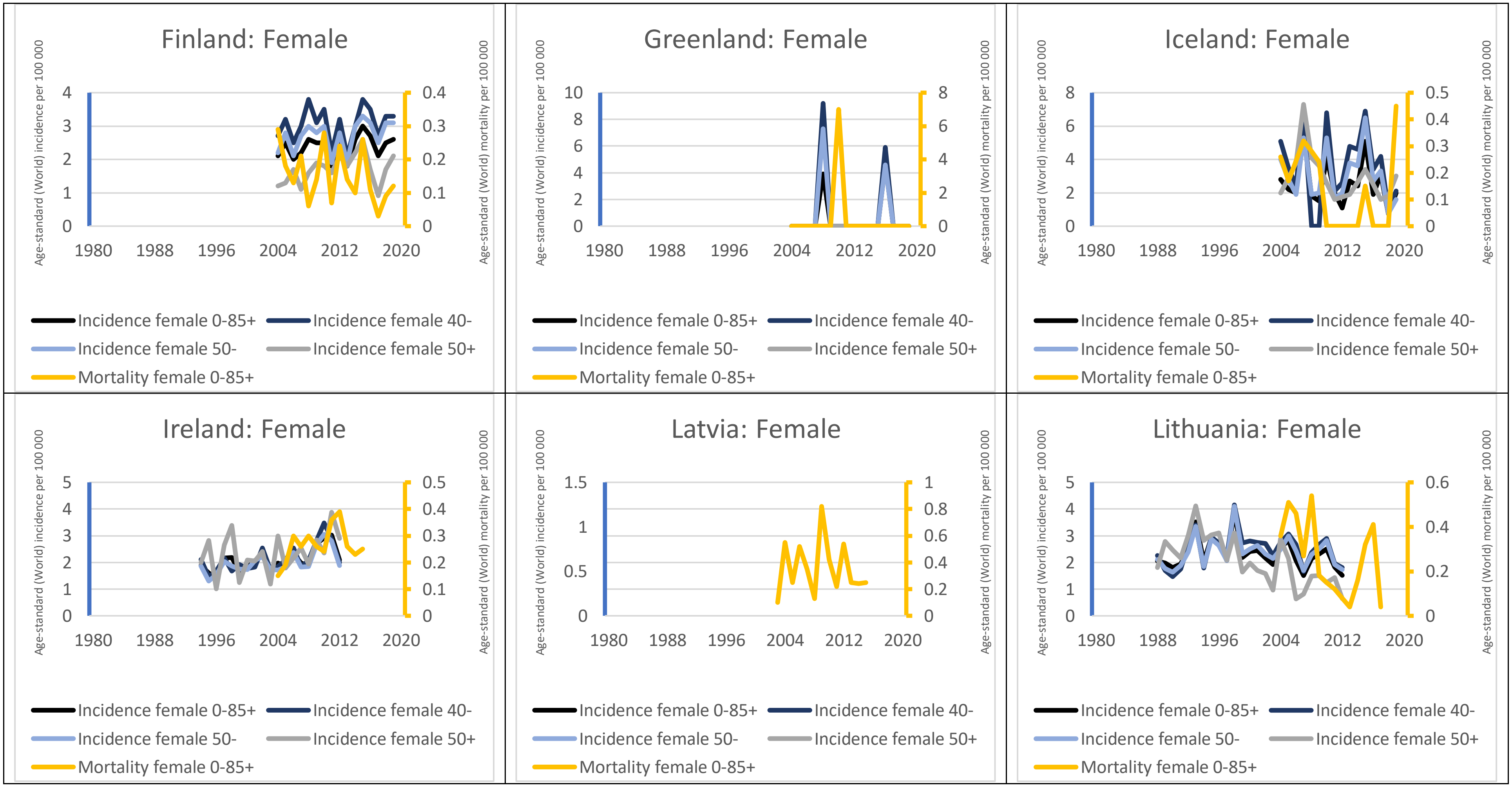
Finland: Female
Greenland: Female
Iceland: Female
Ireland: Female
Latvia: Female
Lithuania: Female
Age-standard (World) incidence per 100 000
Age-standard (World) mortality per 100 000
1980
1988
1996
2004
2012
2020
Incidence female 0-85+
Incidence female 40-
Incidence female 50-
Incidence female 50+
Mortality female 0-85+

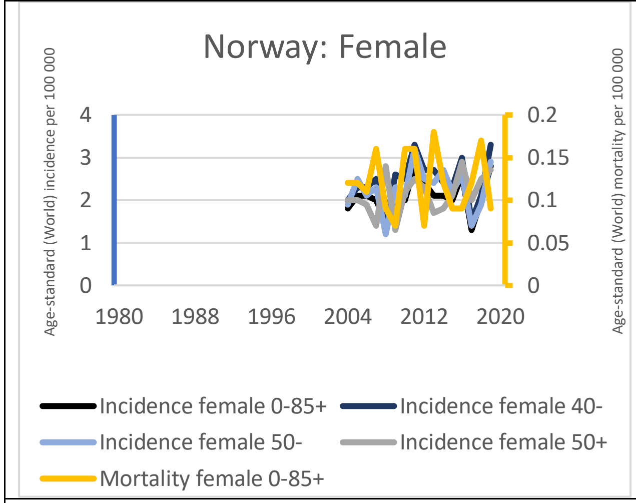

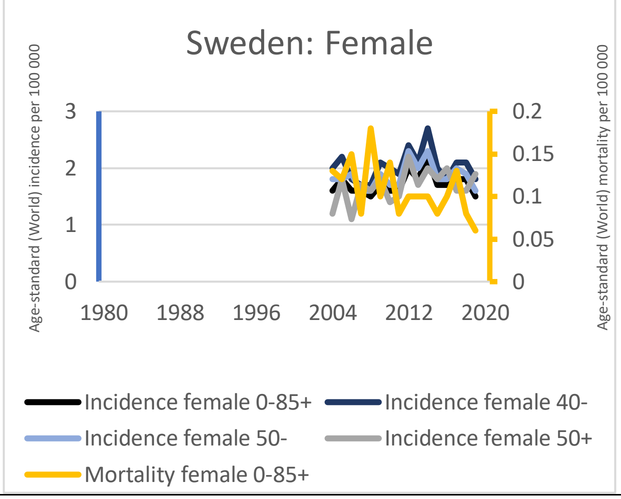

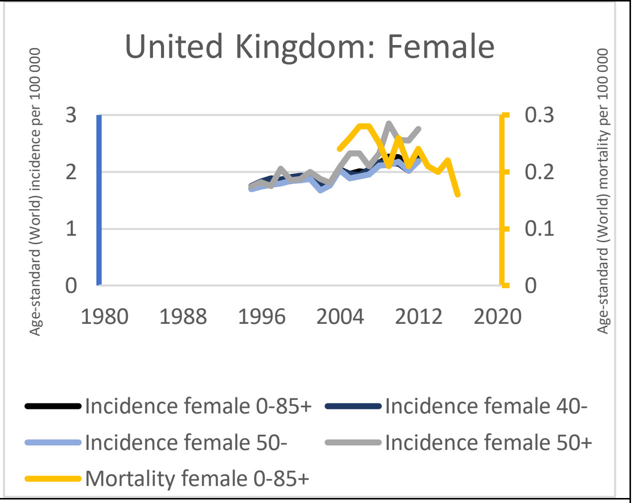

Western Europe

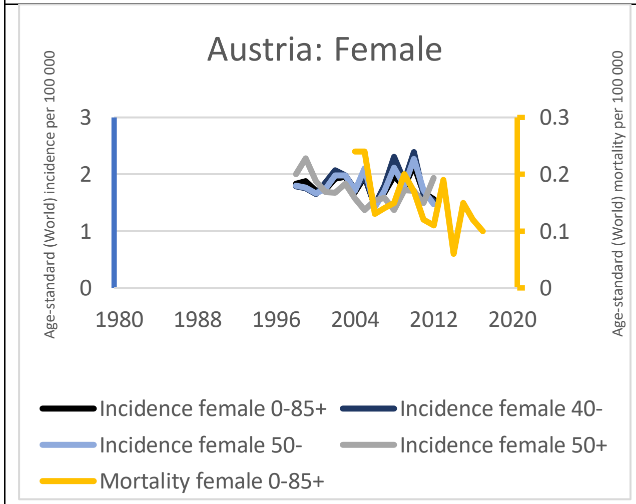

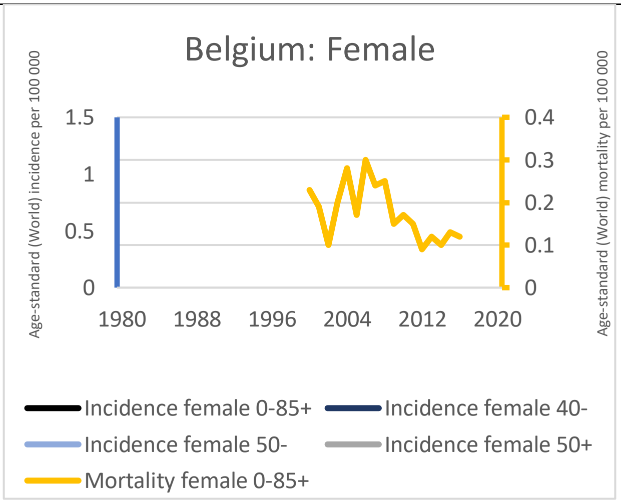

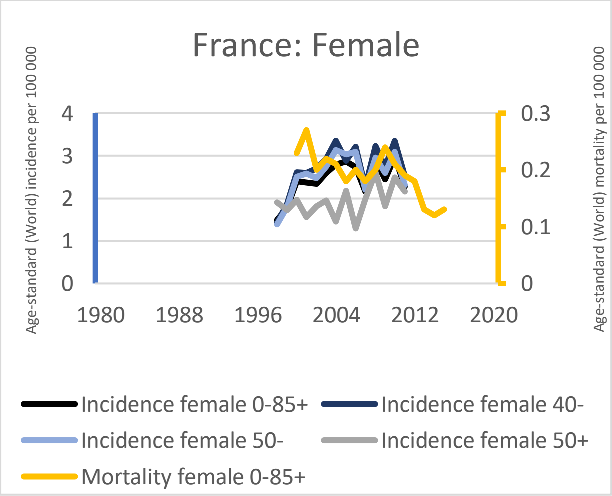

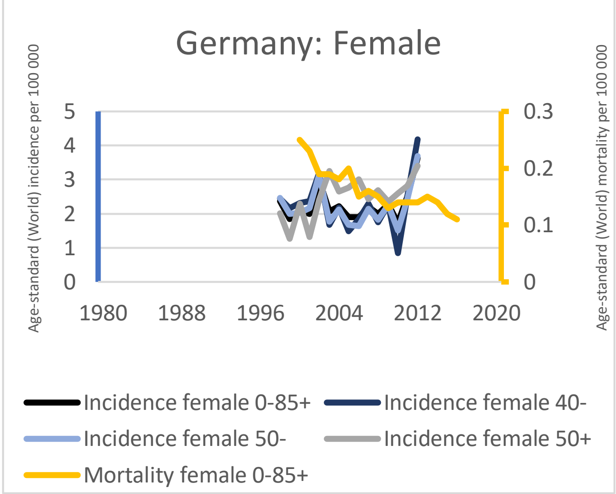

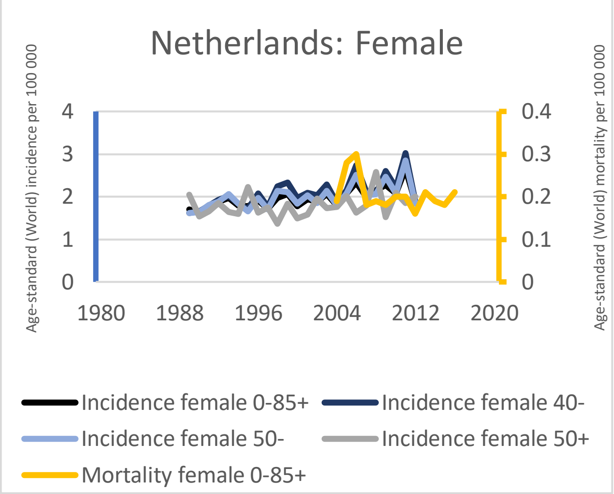

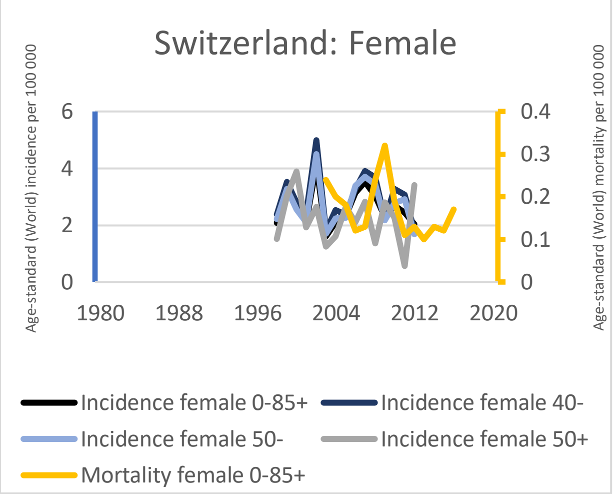

Southern Europe

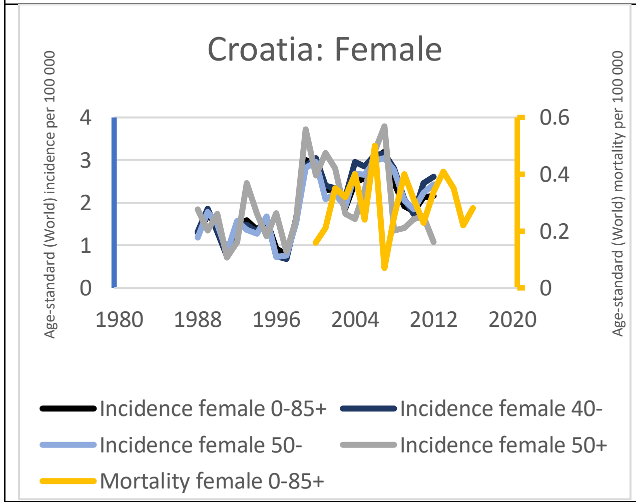

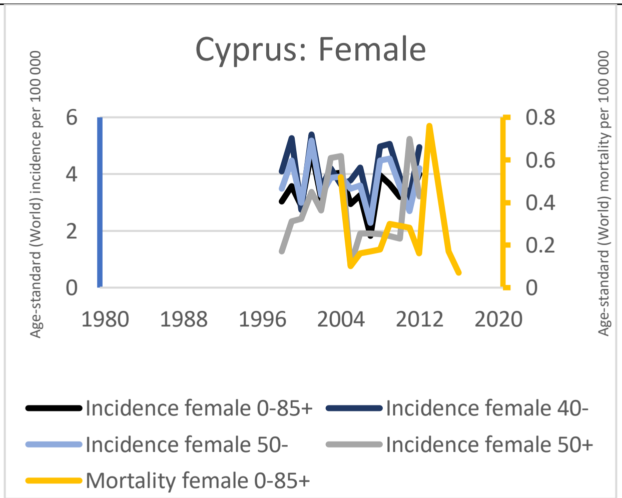

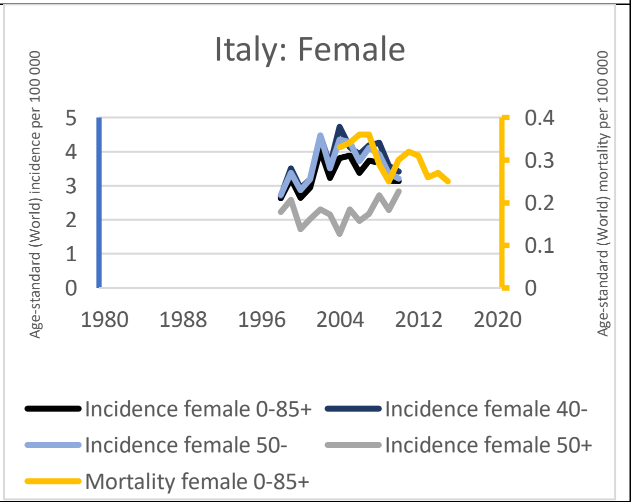

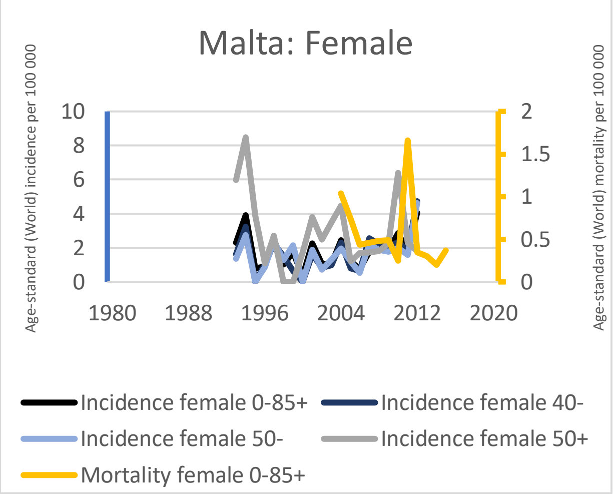
Malta: Female
Age-standard (World) incidence per 100 000
10
8
6
4
2
0
Age-standard (World) mortality per 100 000
2
1.5
1
0.5
0
1980 1988 1996 2004 2012 2020
Incidence female 0-85+
Incidence female 40-
Incidence female 50-
Incidence female 50+
Mortality female 0-85+

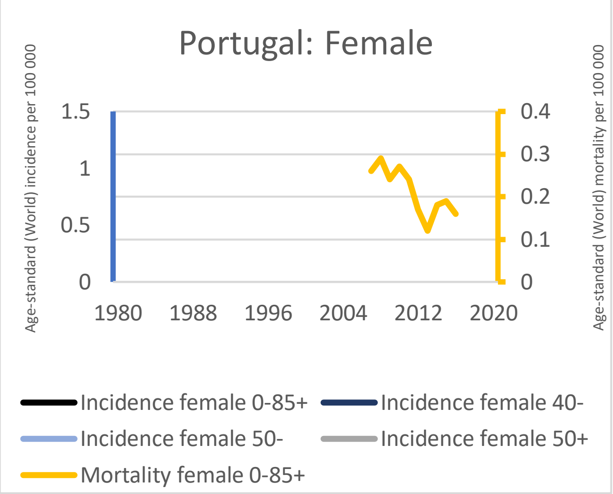
Portugal: Female
Age-standard (World) incidence per 100 000
1.5
1
0.5
0
Age-standard (World) mortality per 100 000
0.4
0.3
0.2
0.1
0
1980 1988 1996 2004 2012 2020
Incidence female 0-85+
Incidence female 40-
Incidence female 50-
Incidence female 50+
Mortality female 0-85+

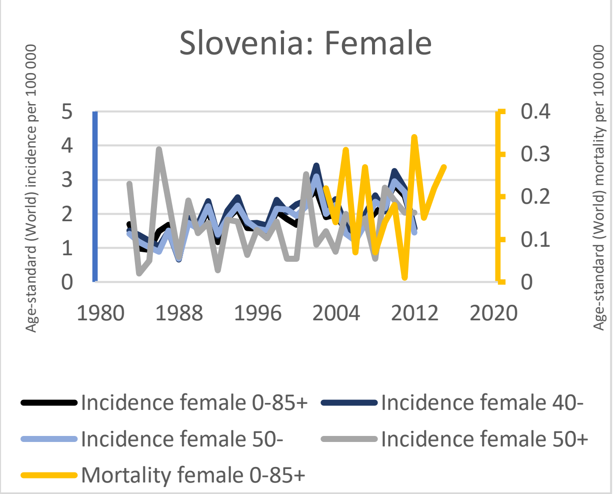
Slovenia: Female
Age-standard (World) incidence per 100 000
5
4
3
2
1
0
Age-standard (World) mortality per 100 000
0.4
0.3
0.2
0.1
0
1980 1988 1996 2004 2012 2020
Incidence female 0-85+
Incidence female 40-
Incidence female 50-
Incidence female 50+
Mortality female 0-85+

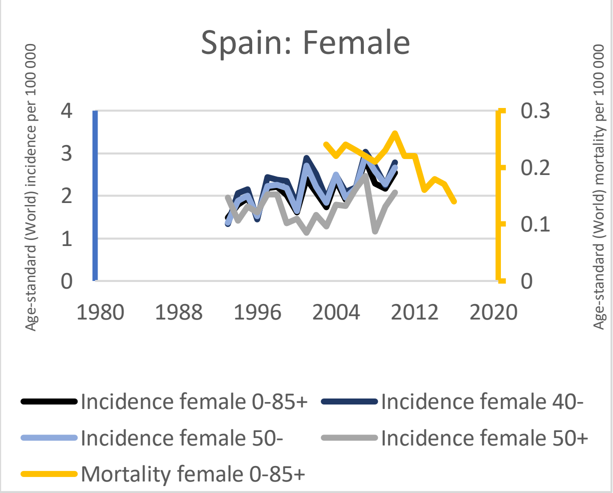
Spain: Female
Age-standard (World) incidence per 100 000
4
3
2
1
0
Age-standard (World) mortality per 100 000
0.3
0.2
0.1
0
1980 1988 1996 2004 2012 2020
Incidence female 0-85+
Incidence female 40-
Incidence female 50-
Incidence female 50+
Mortality female 0-85+

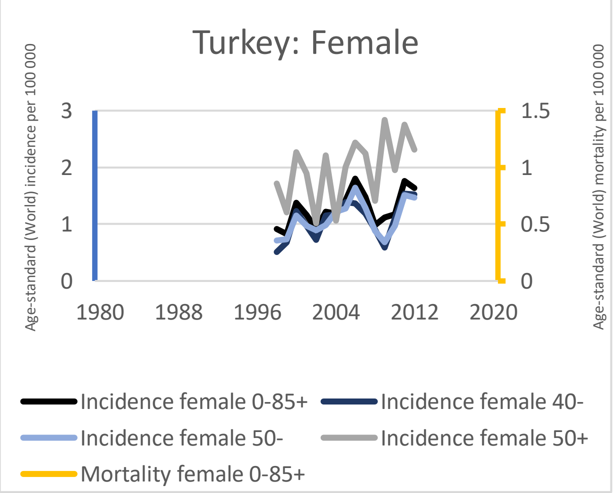
Turkey: Female
Age-standard (World) incidence per 100 000
3
2
1
0
Age-standard (World) mortality per 100 000
1.5
1
0.5
0
1980 1988 1996 2004 2012 2020
Incidence female 0-85+
Incidence female 40-
Incidence female 50-
Incidence female 50+
Mortality female 0-85+

Eastern Europe

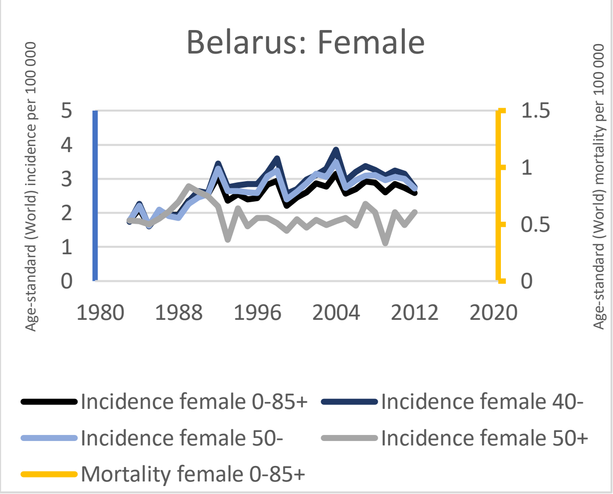

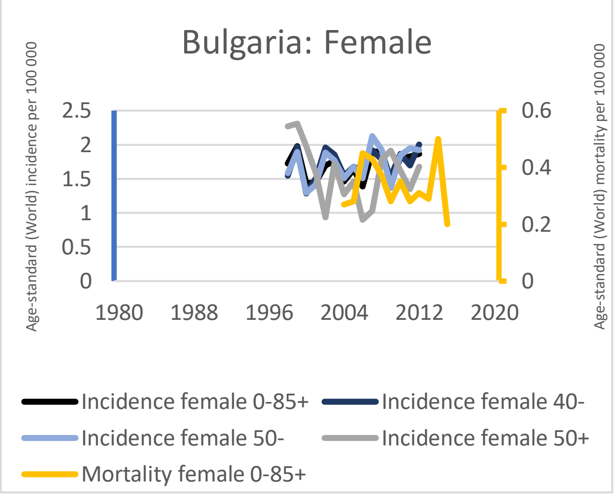

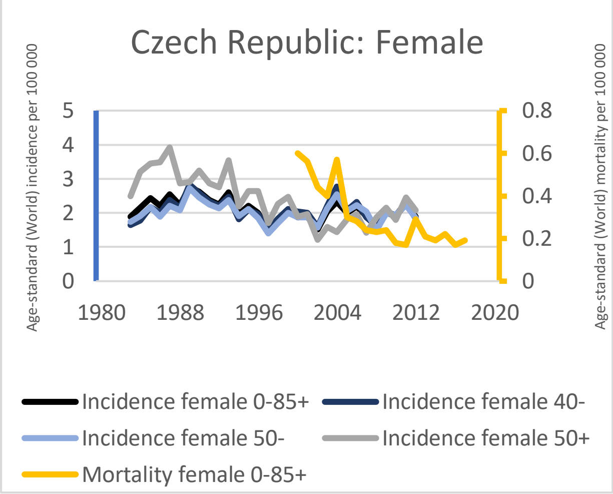

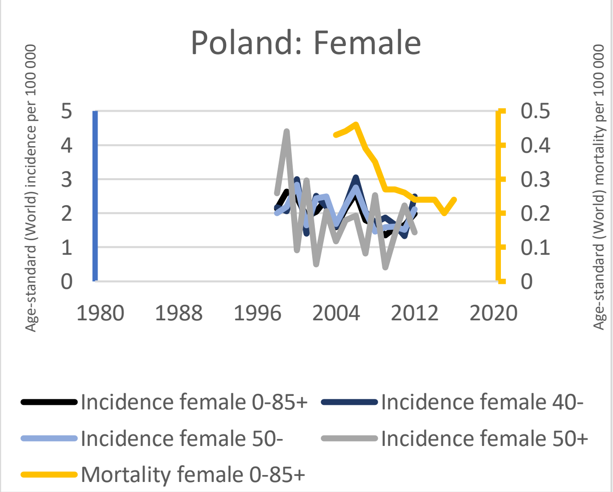

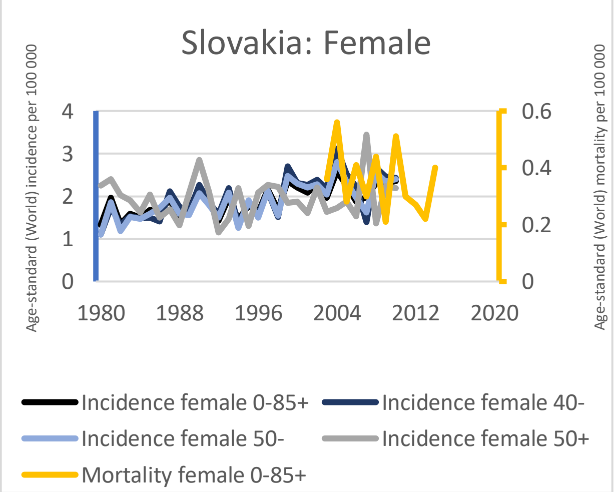

Africa		
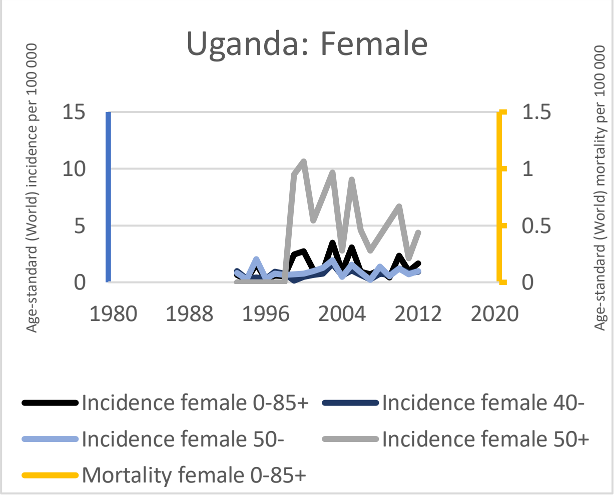Uganda: Female<br>Age-standard (World) incidence per 100 000<br>Age-standard (World) mortality per 100 000<br>15<br>10<br>5<br>0<br>1.5<br>1<br>0.5<br>0<br>1980<br>1988<br>1996<br>2004<br>2012<br>2020<br>Incidence female 0-85+<br>Incidence female 40-<br>Incidence female 50-<br>Incidence female 50+<br>Mortality female 0-85+		

Supplementary Figure 4. Results of joinpoint regression for individual countries

a.) Incidence male all ages

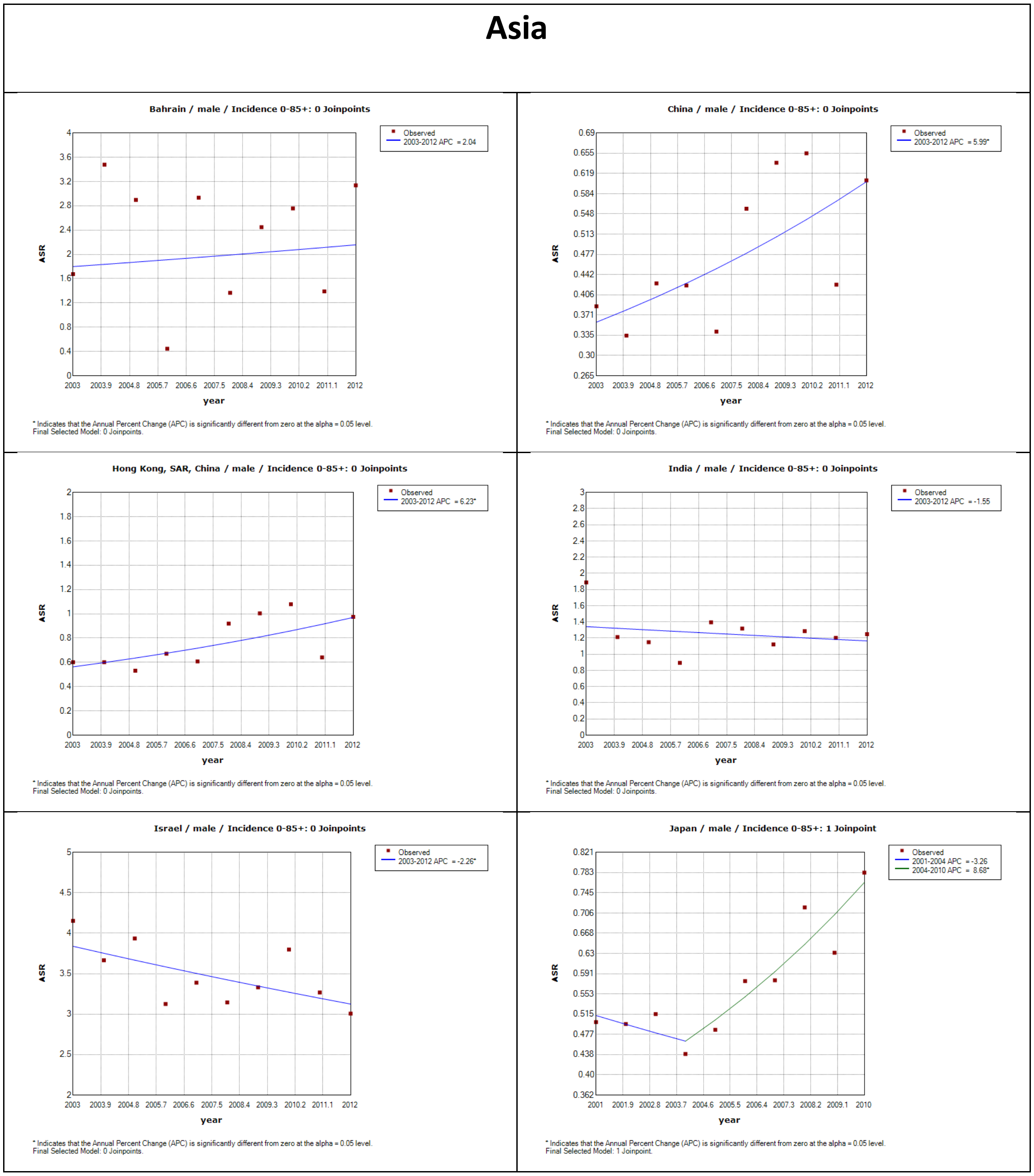

Korea / male / Incidence 0-85+: 1 Joinpoint

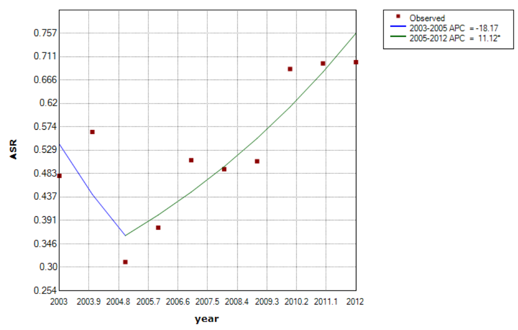

* Indicates that the Annual Percent Change (APC) is significantly different from zero at the alpha = 0.05 level.
Final Selected Model: 1 Joinpoint.

Kuwait / male / Incidence 0-85+: 0 Joinpoints

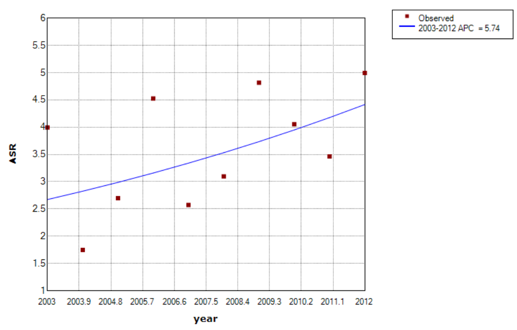

* Indicates that the Annual Percent Change (APC) is significantly different from zero at the alpha = 0.05 level.
Final Selected Model: 0 Joinpoints.

Philippines / male / Incidence 0-85+: 0 Joinpoints

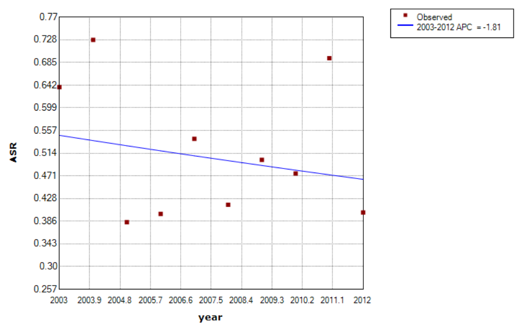

* Indicates that the Annual Percent Change (APC) is significantly different from zero at the alpha = 0.05 level.
Final Selected Model: 0 Joinpoints.

Thailand / male / Incidence 0-85+: 0 Joinpoints

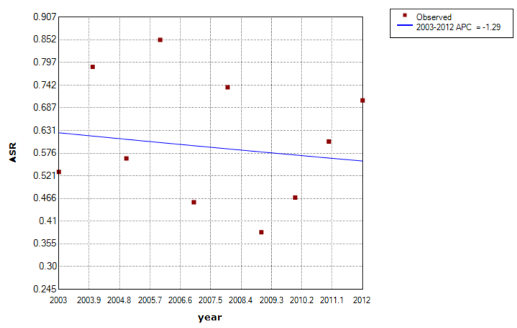

* Indicates that the Annual Percent Change (APC) is significantly different from zero at the alpha = 0.05 level.
Final Selected Model: 0 Joinpoints.

Oceania

Australia / male / Incidence 0-85+: 0 Joinpoints

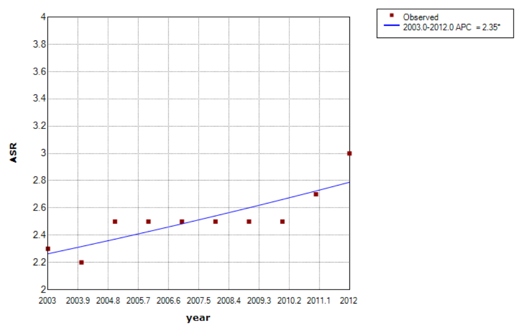

* Indicates that the Annual Percent Change (APC) is significantly different from zero at the alpha = 0.05 level.
Final Selected Model: 0 Joinpoints.

New Zealand / male / Incidence 0-85+: 0 Joinpoints

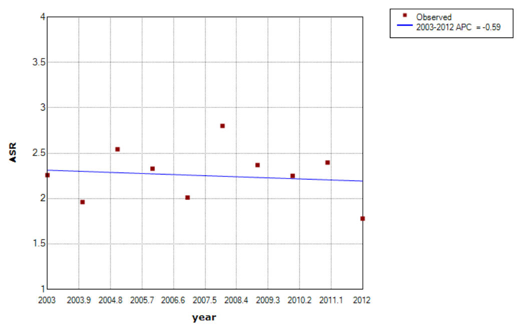

* Indicates that the Annual Percent Change (APC) is significantly different from zero at the alpha = 0.05 level.
Final Selected Model: 0 Joinpoints.

North America

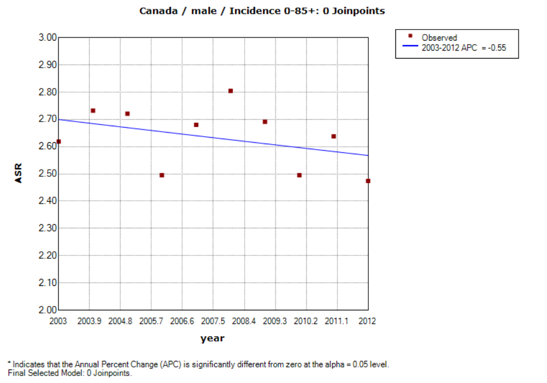

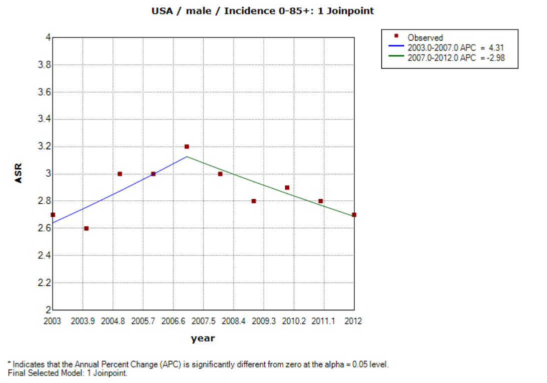

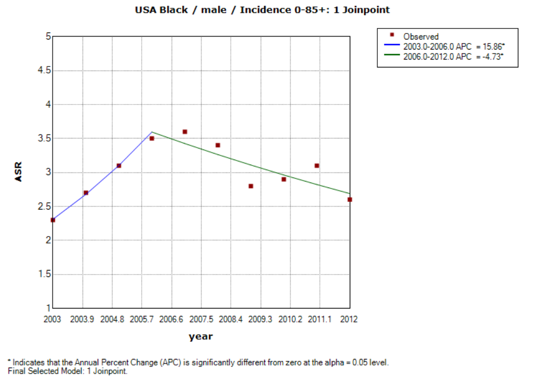

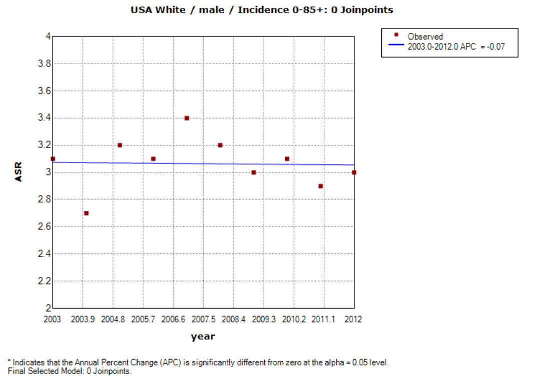

South America

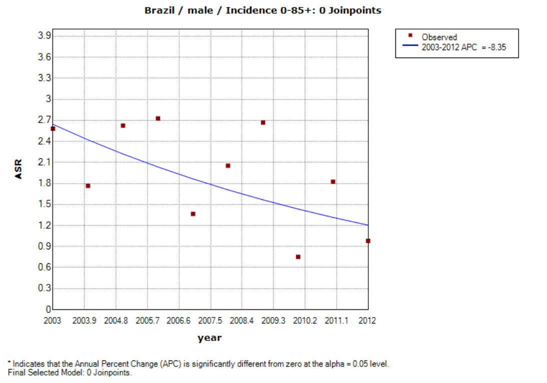

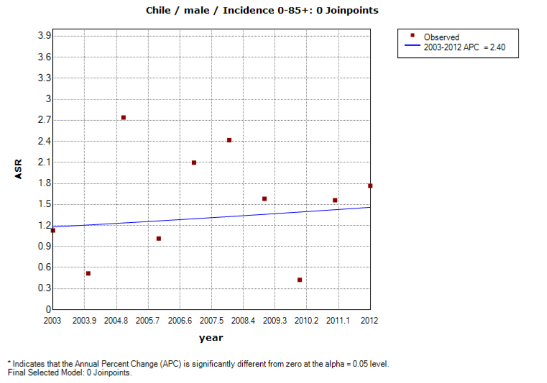

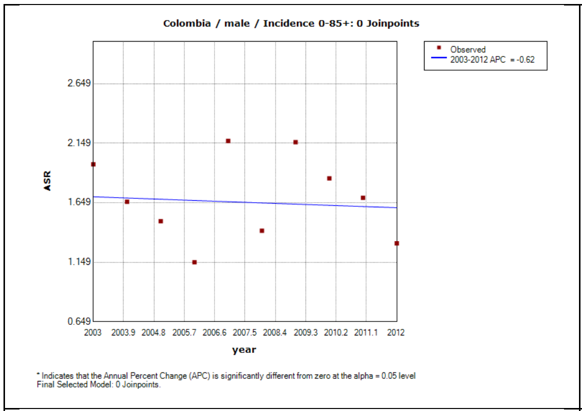

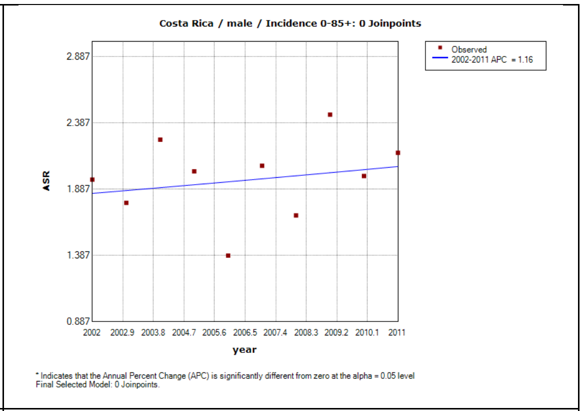

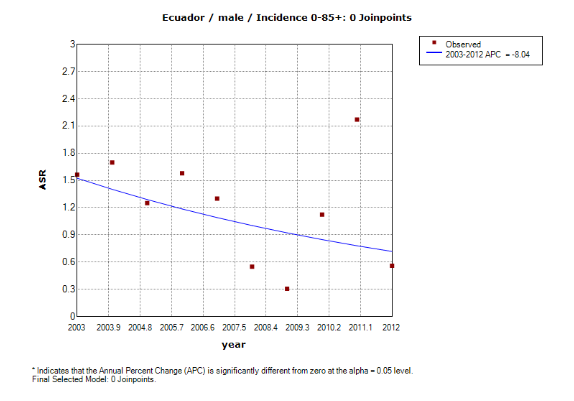

Northern Europe

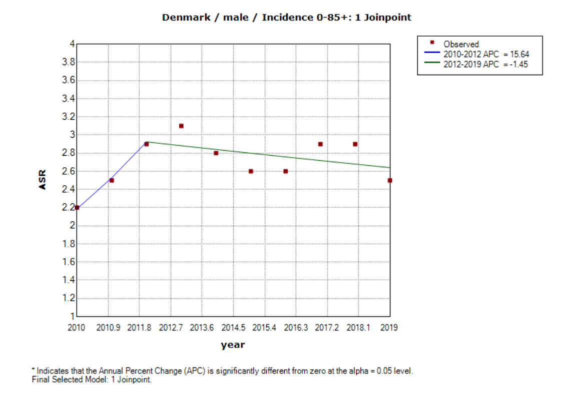

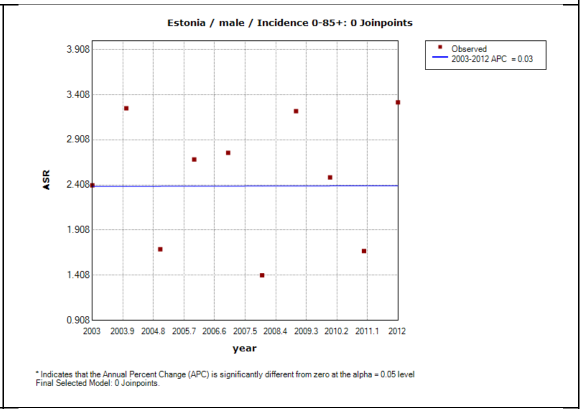

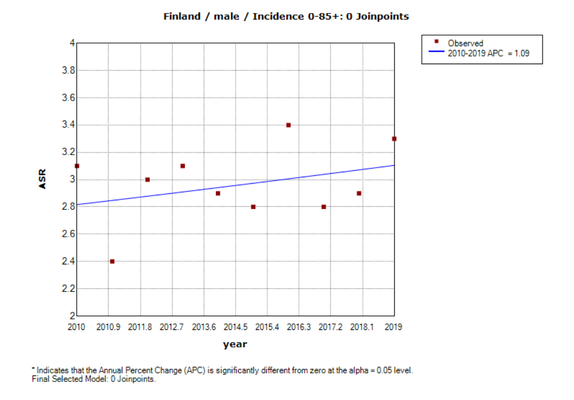

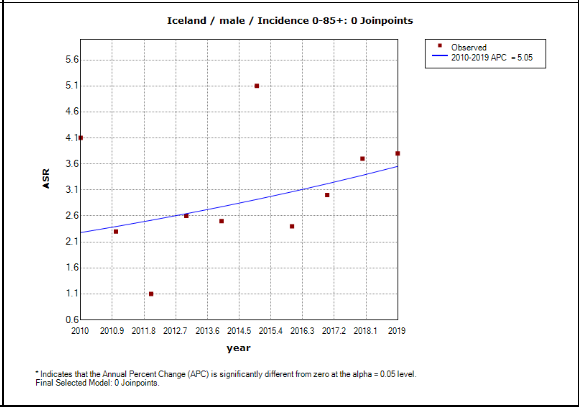

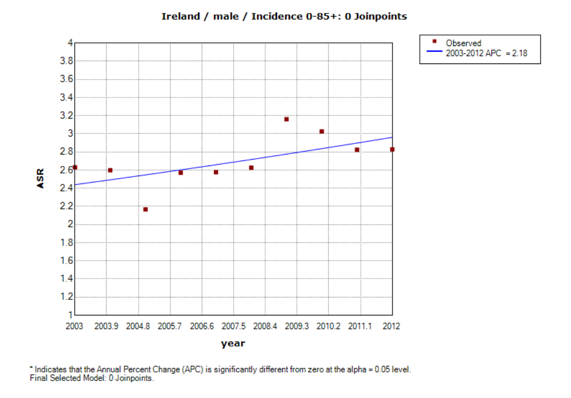

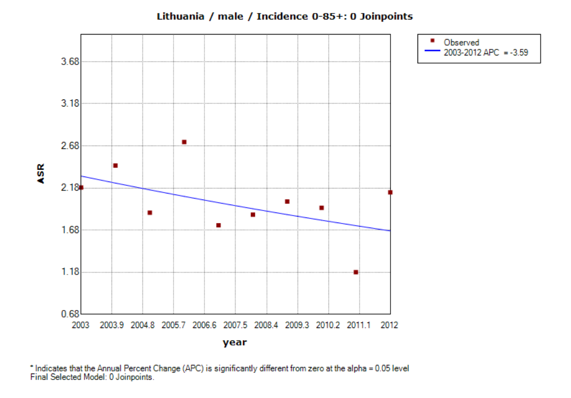

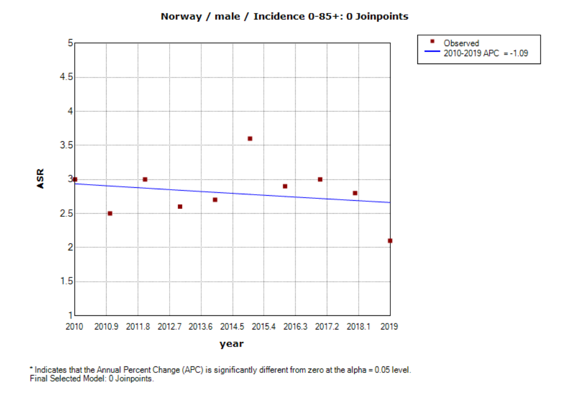

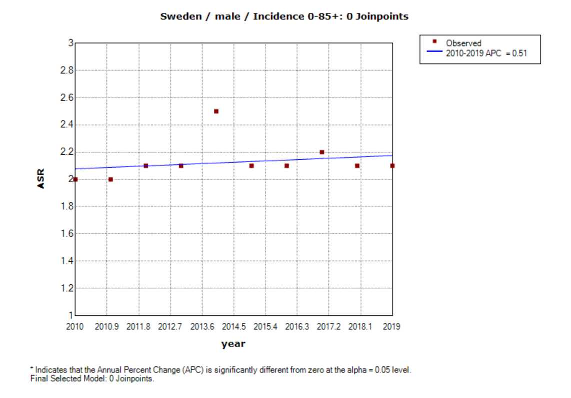

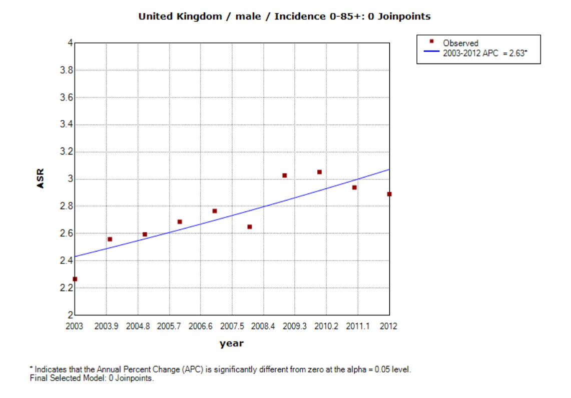

Western Europe

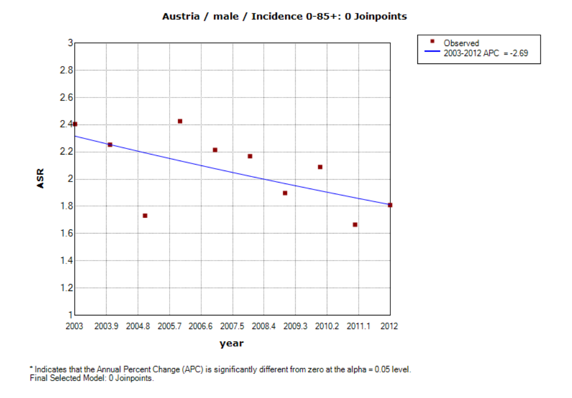

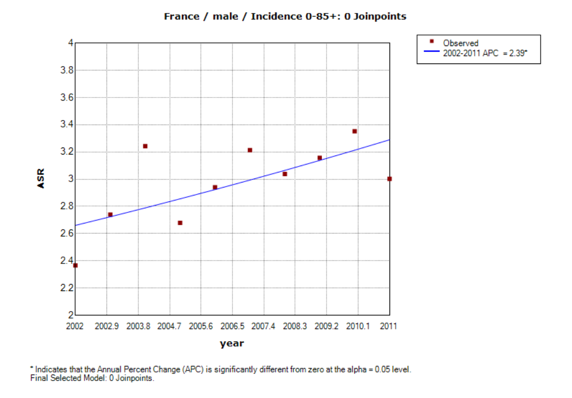

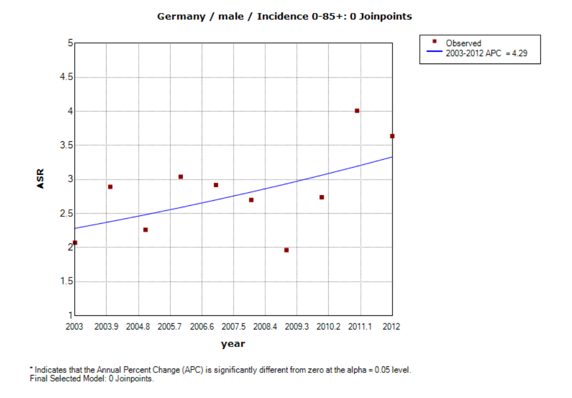

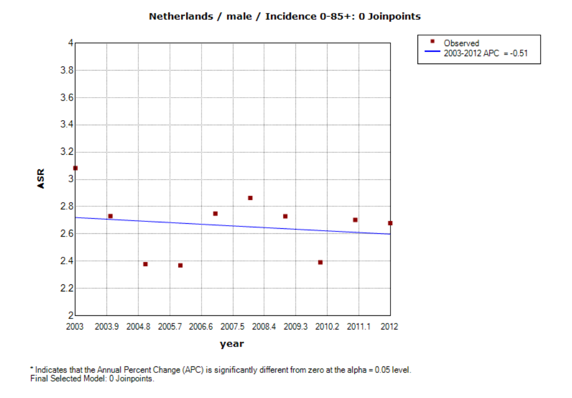

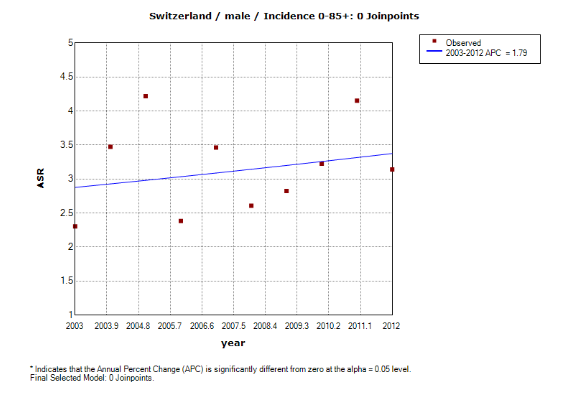

Southern Europe

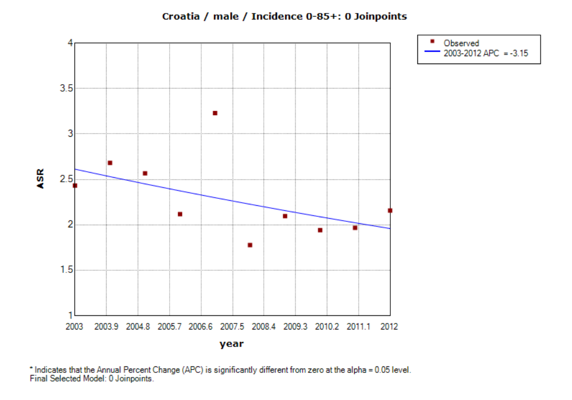

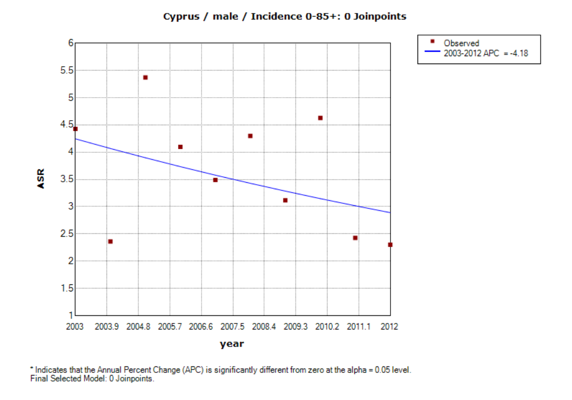

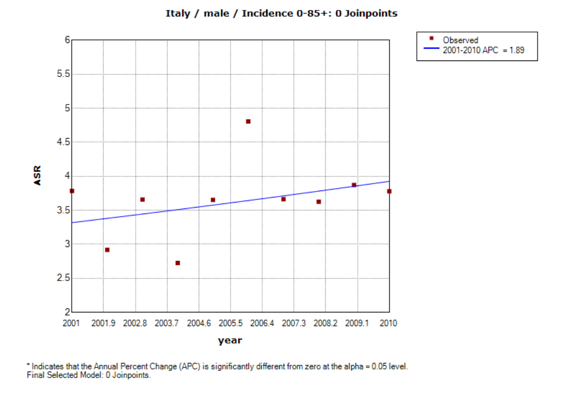

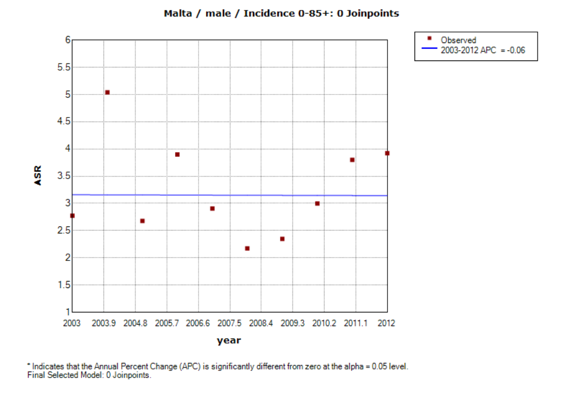

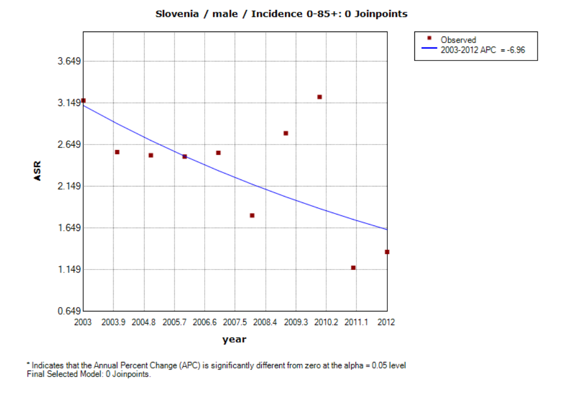

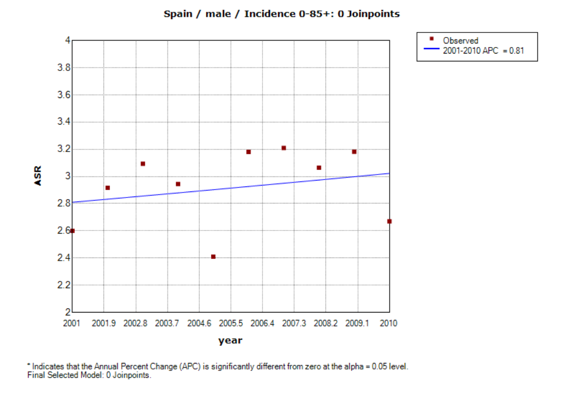

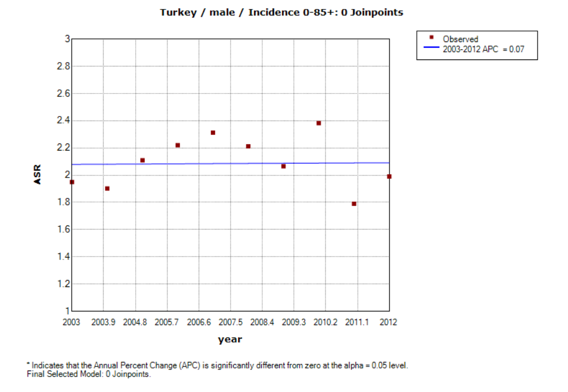

Eastern Europe

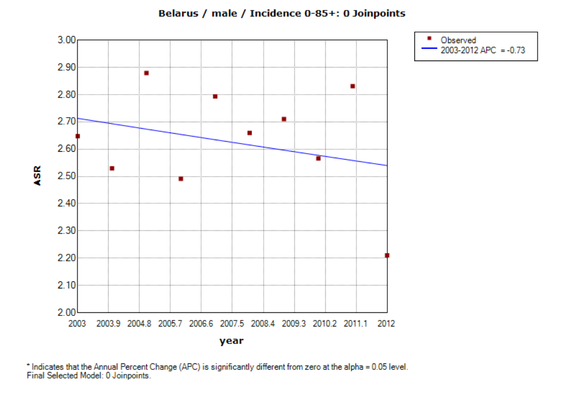

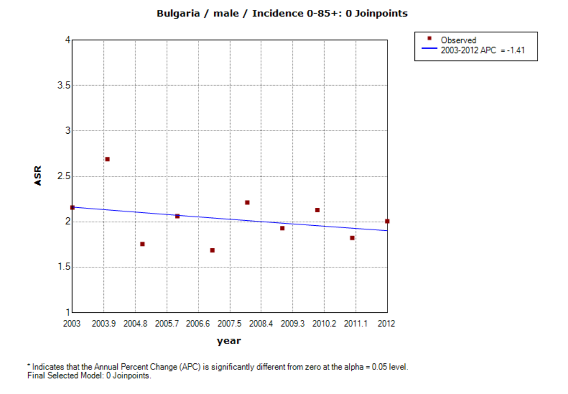

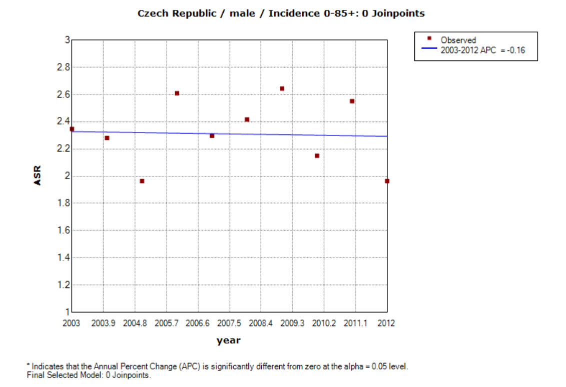
Czech Republic / male / Incidence 0-85+: 0 Joinpoints
Observed
2003-2012 APC = -0.16
ASR
year
* Indicates that the Annual Percent Change (APC) is significantly different from zero at the alpha = 0.05 level.
Final Selected Model: 0 Joinpoints.

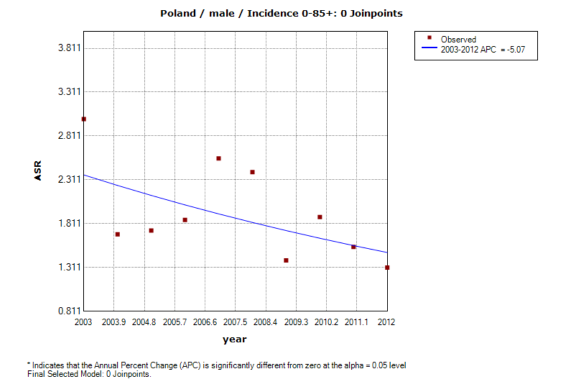
Poland / male / Incidence 0-85+: 0 Joinpoints
Observed
2003-2012 APC = -5.07
ASR
year
* Indicates that the Annual Percent Change (APC) is significantly different from zero at the alpha = 0.05 level
Final Selected Model: 0 Joinpoints.

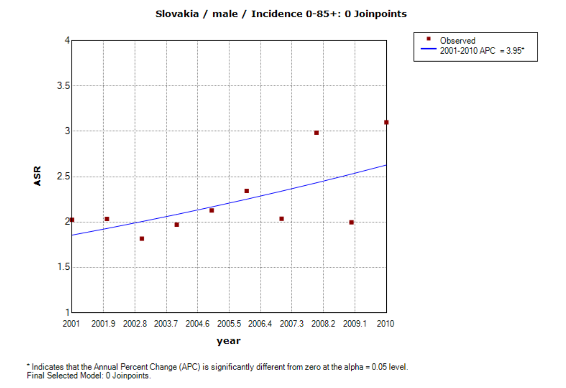
Slovakia / male / Incidence 0-85+: 0 Joinpoints
Observed
2001-2010 APC = 3.95*
ASR
year
* Indicates that the Annual Percent Change (APC) is significantly different from zero at the alpha = 0.05 level.
Final Selected Model: 0 Joinpoints.

Africa

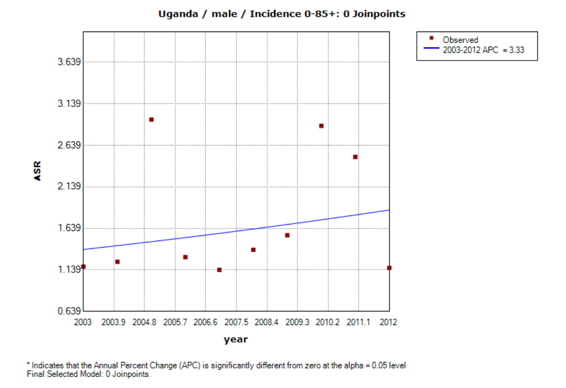
Uganda / male / Incidence 0-85+: 0 Joinpoints
Observed
2003-2012 APC = 3.33
ASR
year
* Indicates that the Annual Percent Change (APC) is significantly different from zero at the alpha = 0.05 level
Final Selected Model: 0 Joinpoints.

b.) Incidence female all ages

Asia

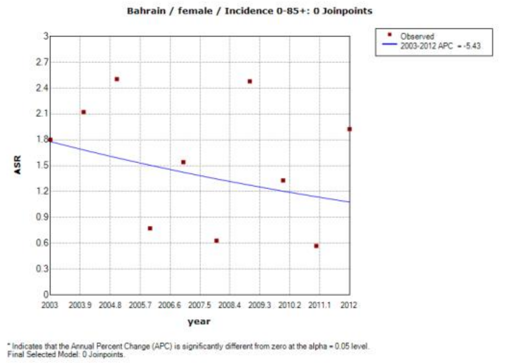

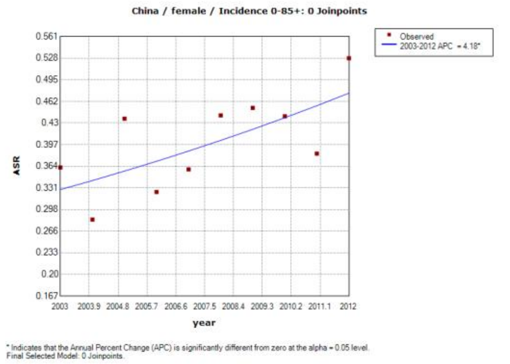

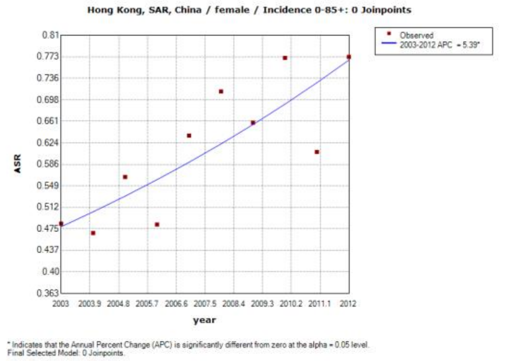

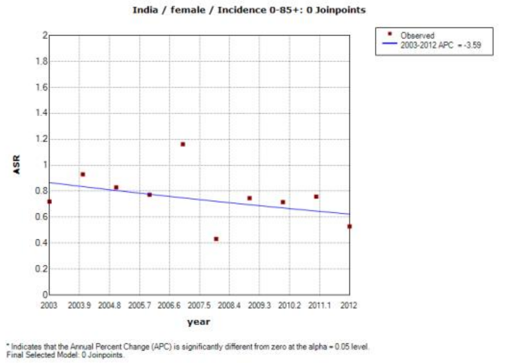

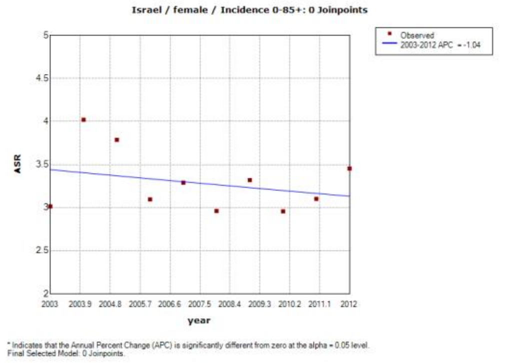

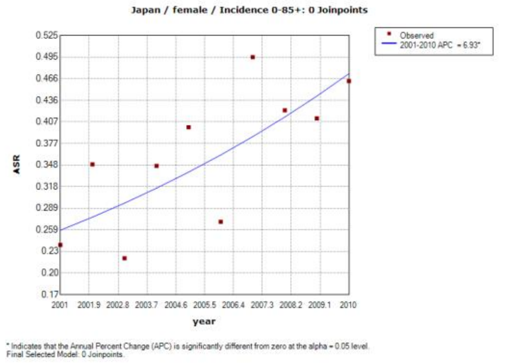

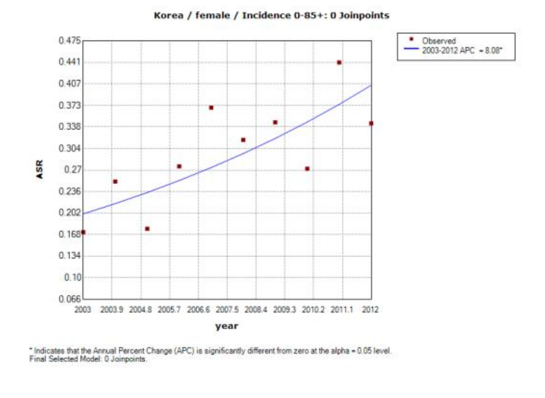
Korea / female / Incidence 0-85+: 0 Joinpoints
Observed
2003-2012 APC = 8.08*
ASR
year
* Indicates that the Annual Percent Change (APC) is significantly different from zero at the alpha = 0.05 level.
Final Selected Model: 0 Joinpoints.

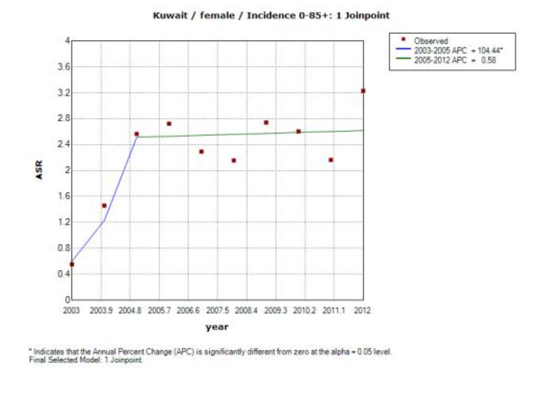
Kuwait / female / Incidence 0-85+: 1 Joinpoint
Observed
2003-2005 APC = 104.44*
2005-2012 APC = 0.58
ASR
year
* Indicates that the Annual Percent Change (APC) is significantly different from zero at the alpha = 0.05 level.
Final Selected Model: 1 Joinpoint.

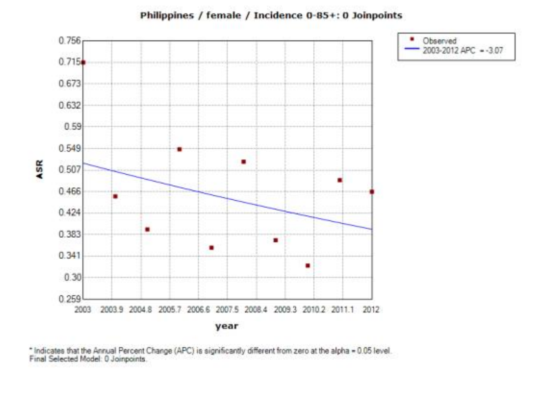
Philippines / female / Incidence 0-85+: 0 Joinpoints
Observed
2003-2012 APC = -3.07
ASR
year
* Indicates that the Annual Percent Change (APC) is significantly different from zero at the alpha = 0.05 level.
Final Selected Model: 0 Joinpoints.

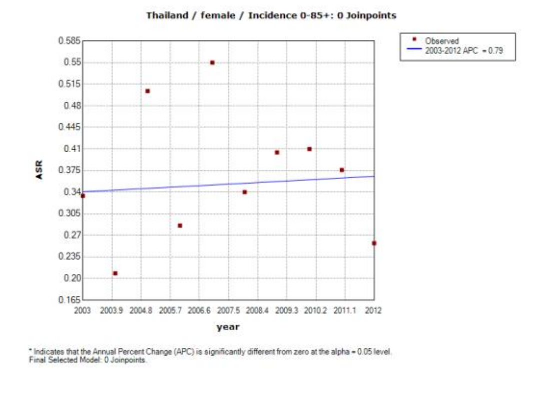
Thailand / female / Incidence 0-85+: 0 Joinpoints
Observed
2003-2012 APC = 0.79
ASR
year
* Indicates that the Annual Percent Change (APC) is significantly different from zero at the alpha = 0.05 level.
Final Selected Model: 0 Joinpoints.

Oceania

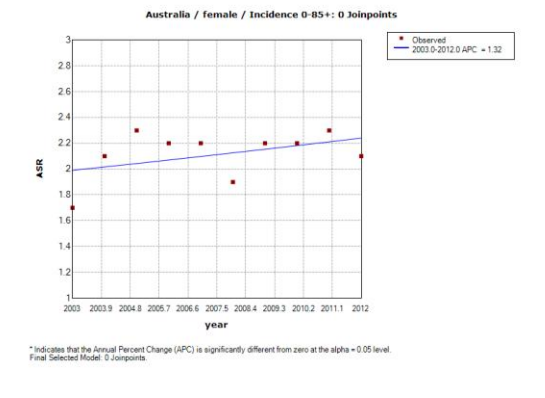
Australia / female / Incidence 0-85+: 0 Joinpoints
Observed
2003.0-2012.0 APC = 1.32
ASR
year
* Indicates that the Annual Percent Change (APC) is significantly different from zero at the alpha = 0.05 level.
Final Selected Model: 0 Joinpoints.

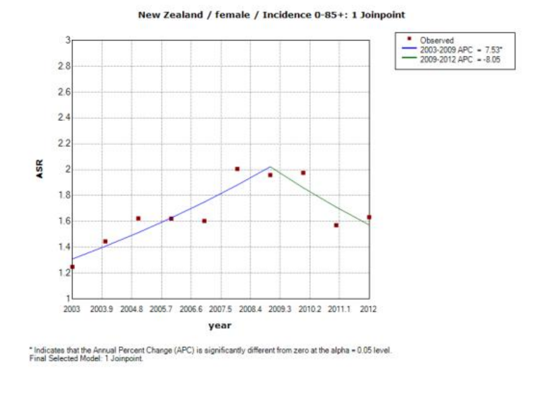
New Zealand / female / Incidence 0-85+: 1 Joinpoint
Observed
2003-2009 APC = 7.53*
2009-2012 APC = -8.05
ASR
year
* Indicates that the Annual Percent Change (APC) is significantly different from zero at the alpha = 0.05 level.
Final Selected Model: 1 Joinpoint.

North America

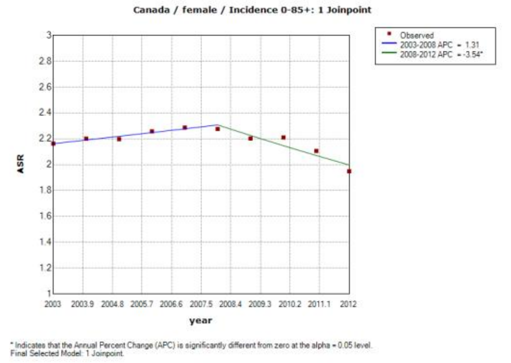

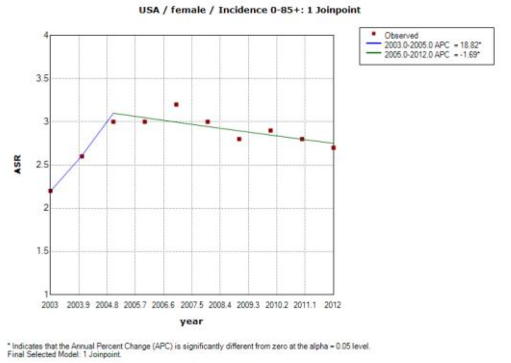

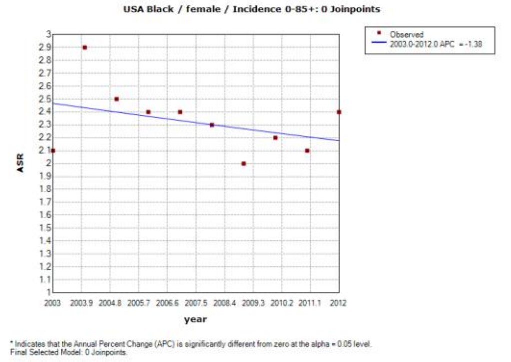

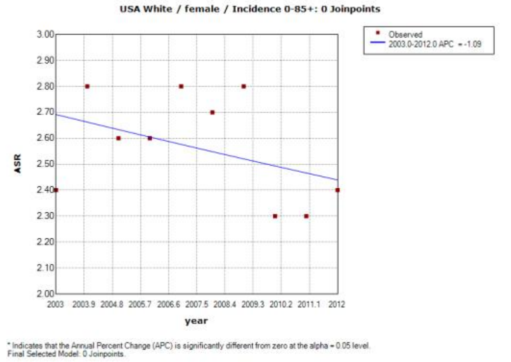

South America

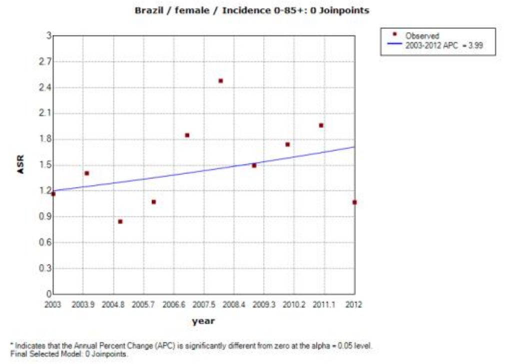

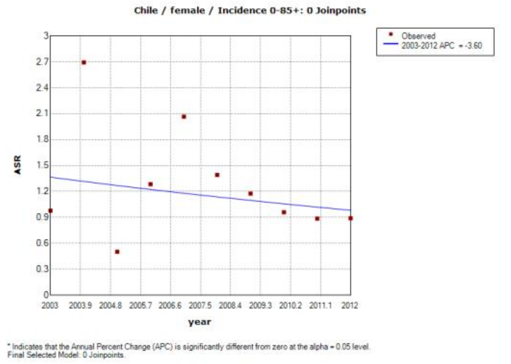

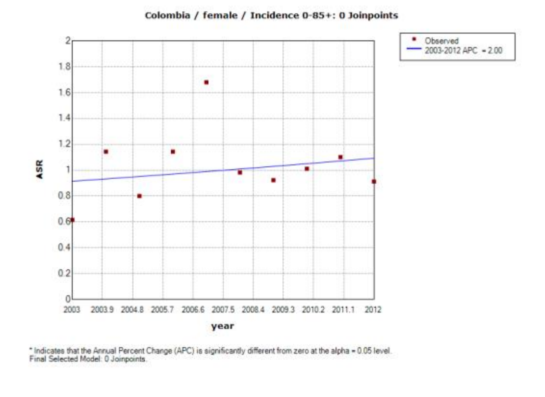

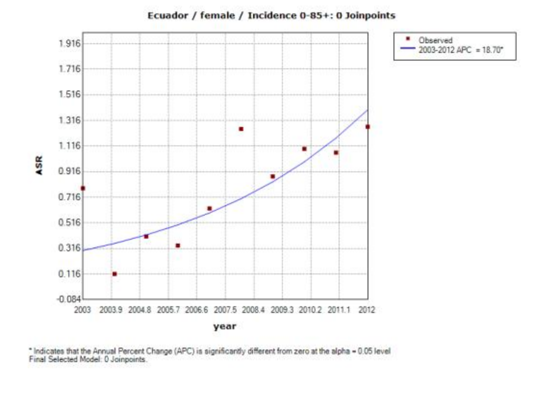

Northern Europe

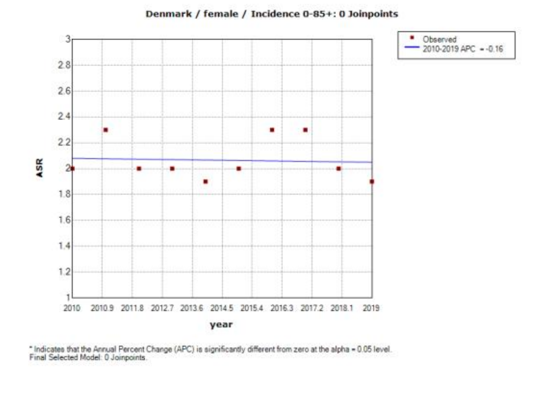

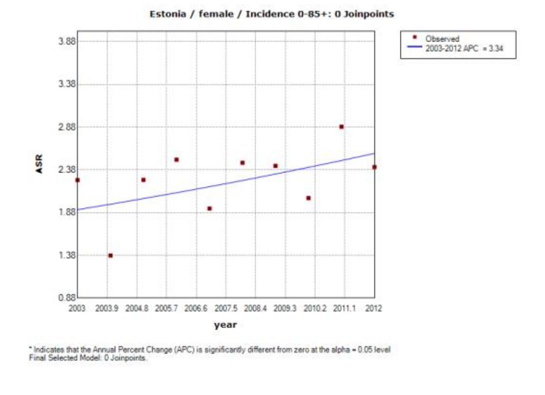

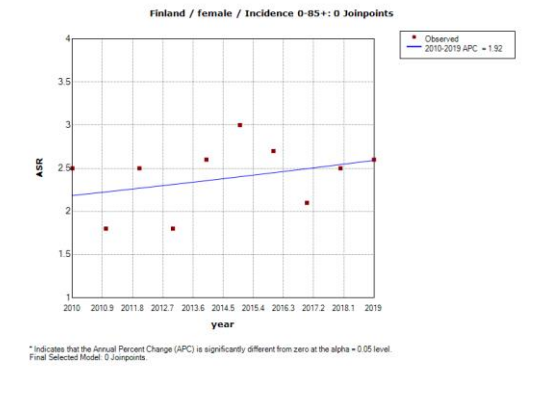

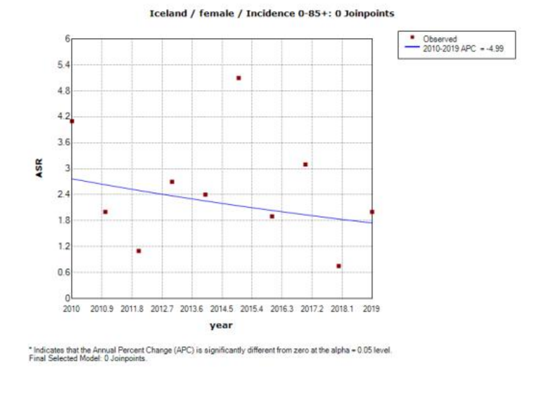

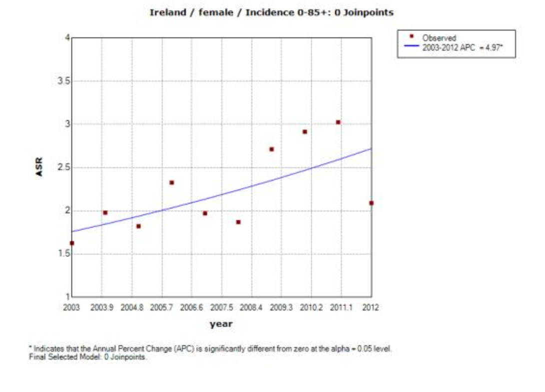

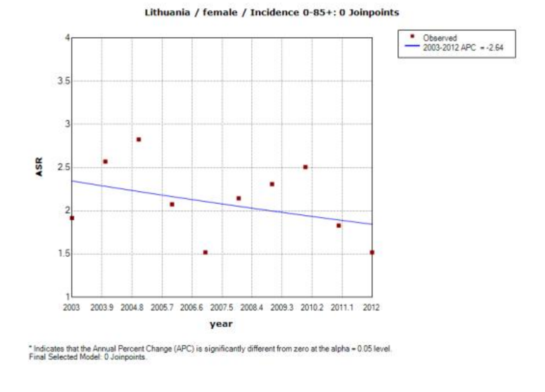

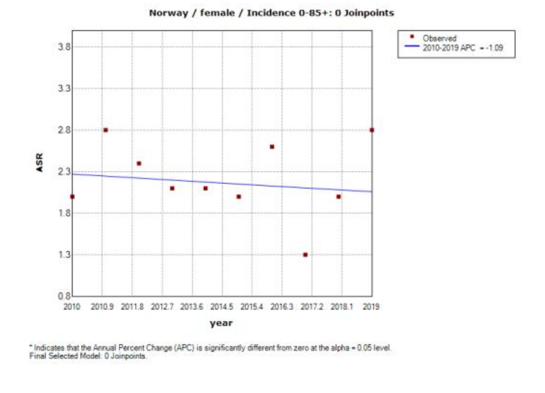

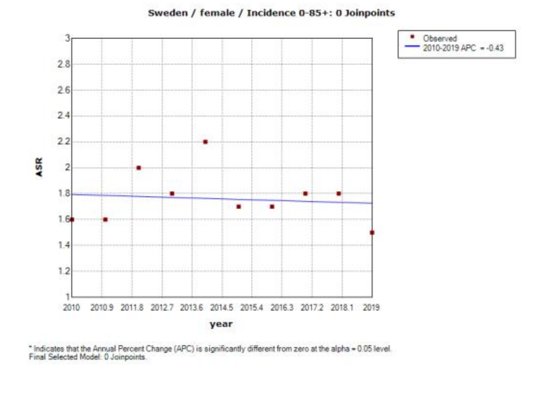

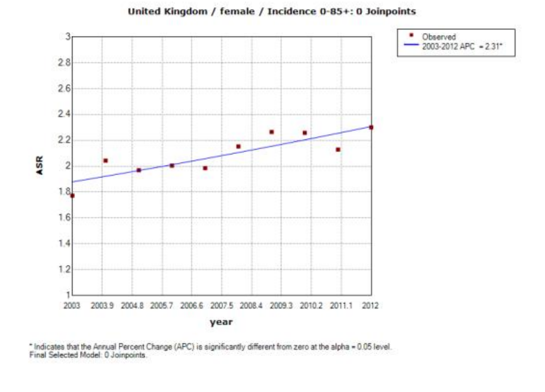

Western Europe

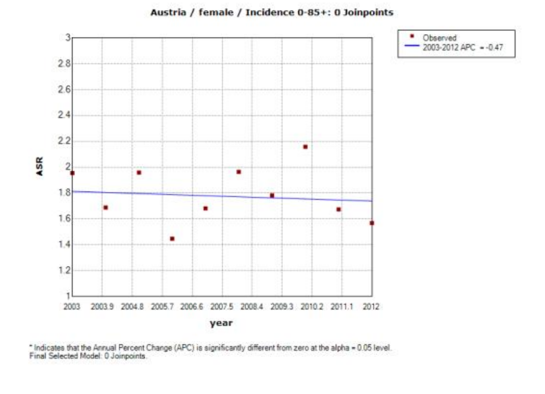

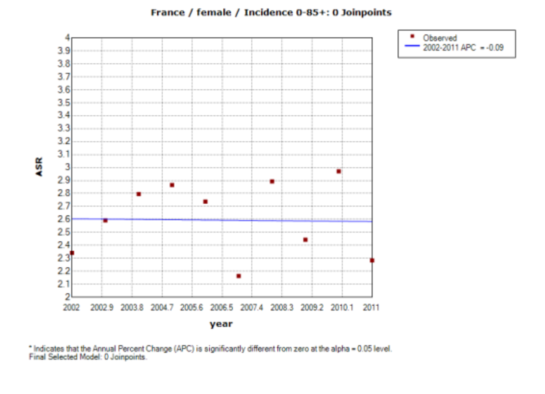

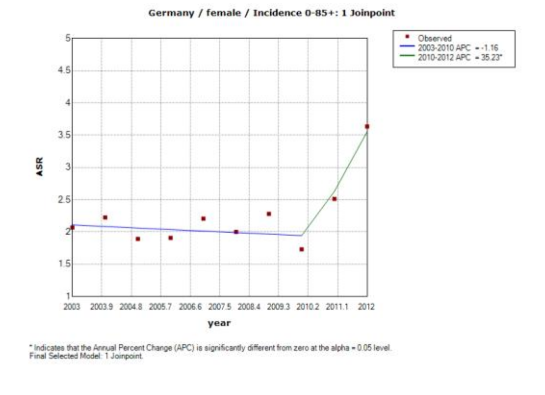

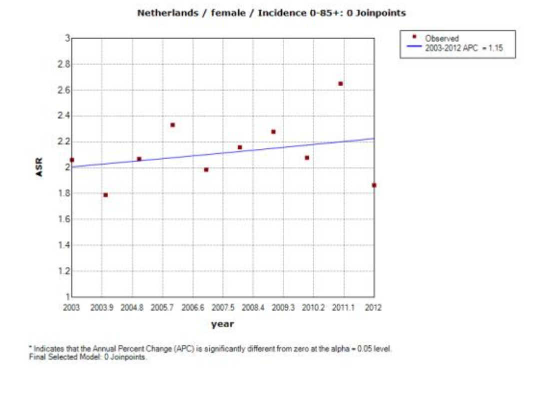

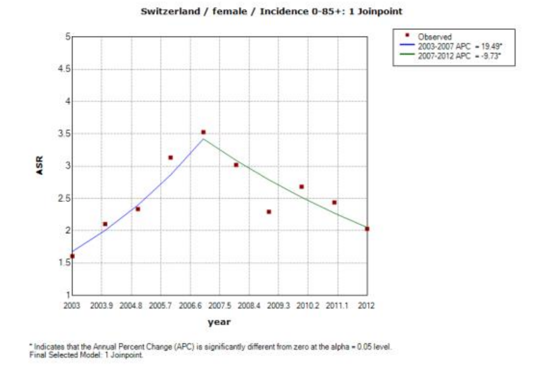

Southern Europe

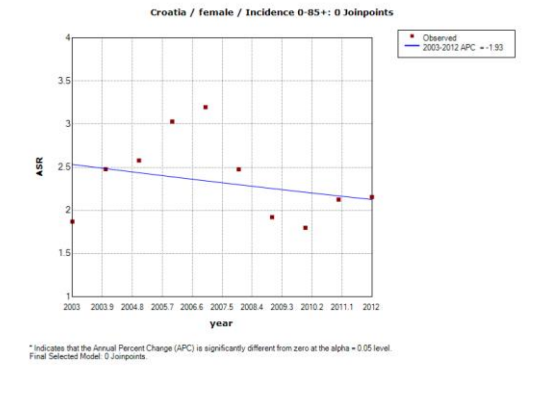

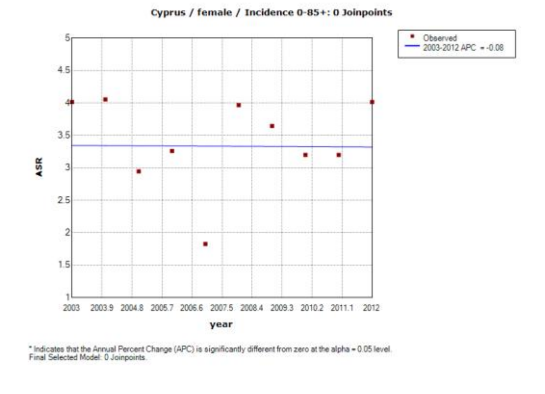

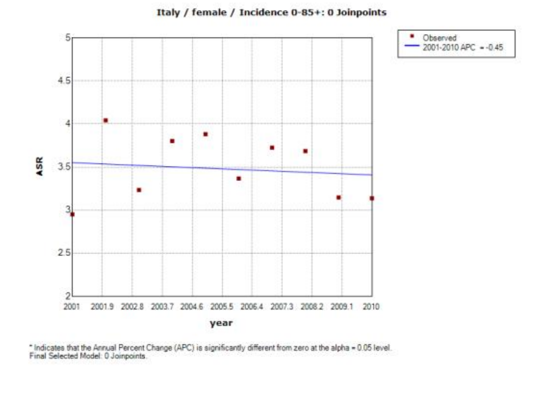

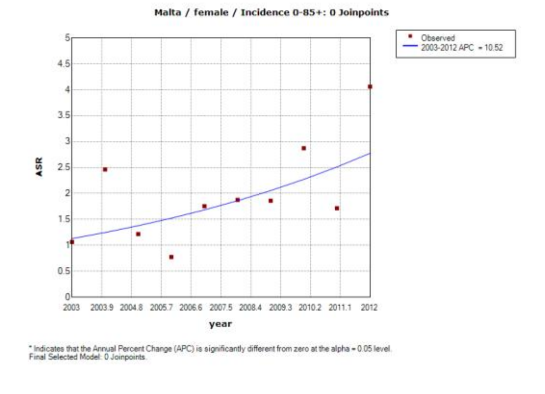

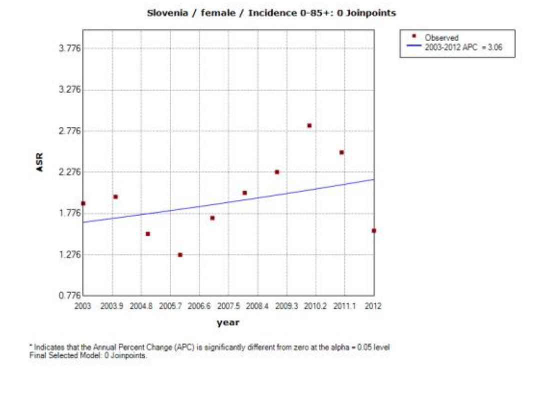

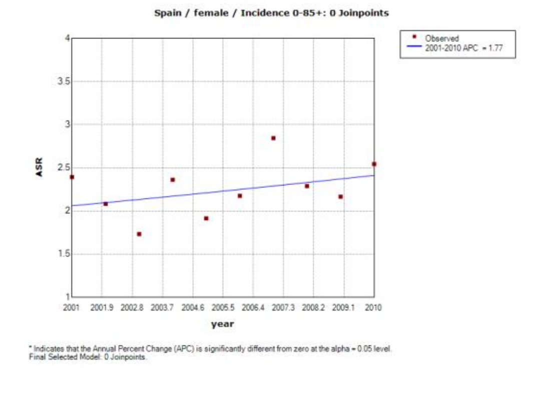

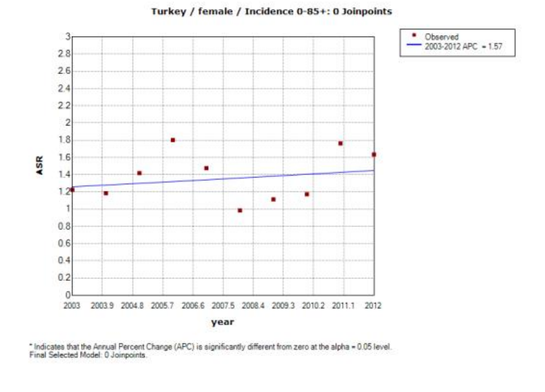

Eastern Europe

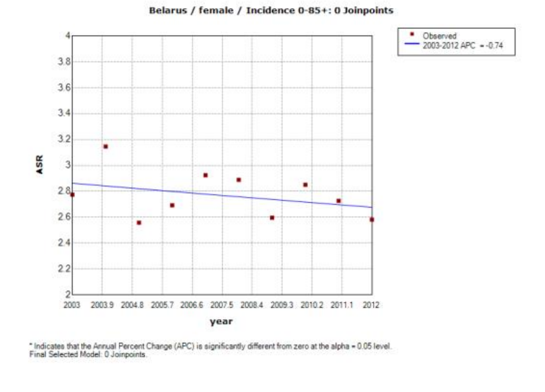

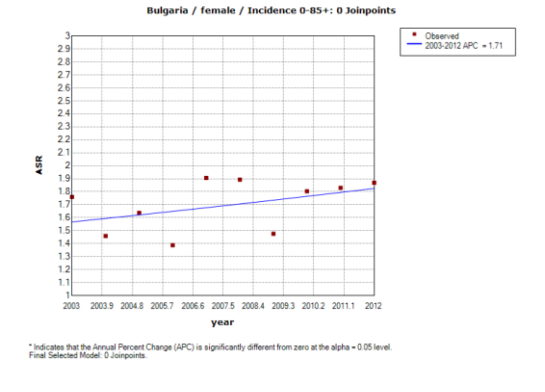

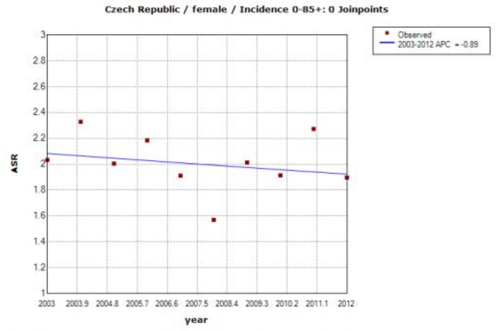

* Indicates that the Annual Percent Change (APC) is significantly different from zero at the alpha = 0.05 level.
Final Selected Model: 0 Joinpoints.

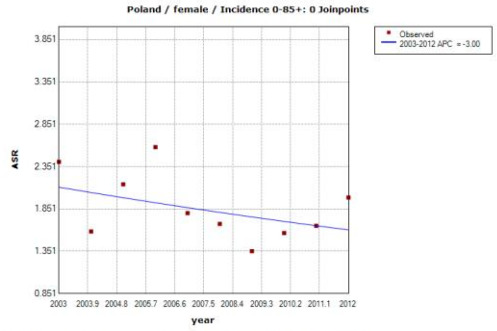

* Indicates that the Annual Percent Change (APC) is significantly different from zero at the alpha = 0.05 level.
Final Selected Model: 0 Joinpoints.

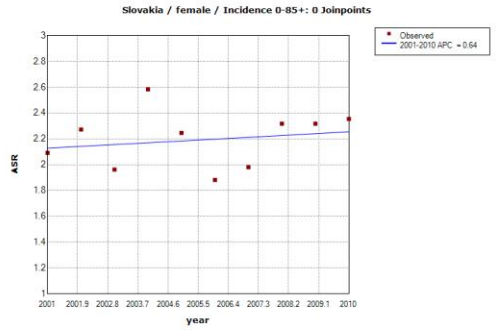

* Indicates that the Annual Percent Change (APC) is significantly different from zero at the alpha = 0.05 level.
Final Selected Model: 0 Joinpoints.

Africa

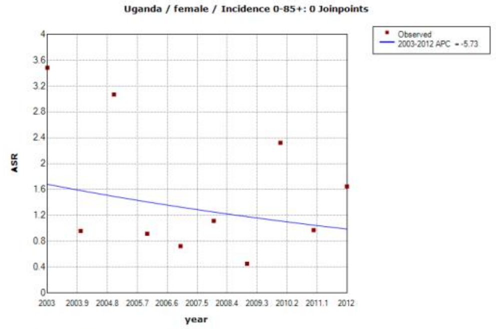

* Indicates that the Annual Percent Change (APC) is significantly different from zero at the alpha = 0.05 level.
Final Selected Model: 0 Joinpoints.

c.) Incidence male below 40 years old

Asia

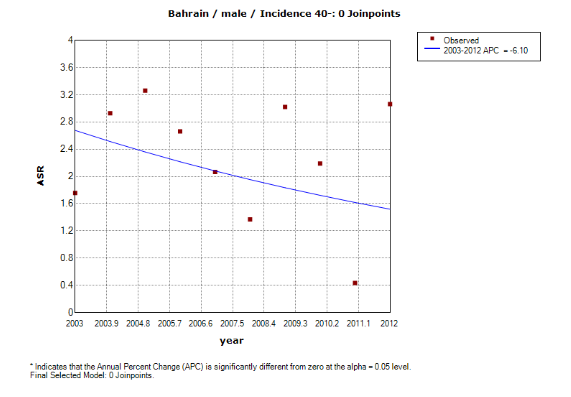

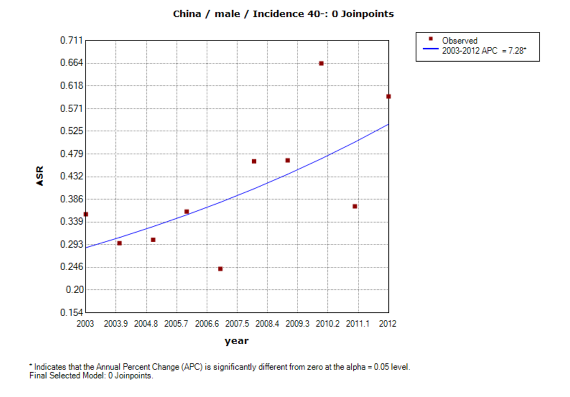

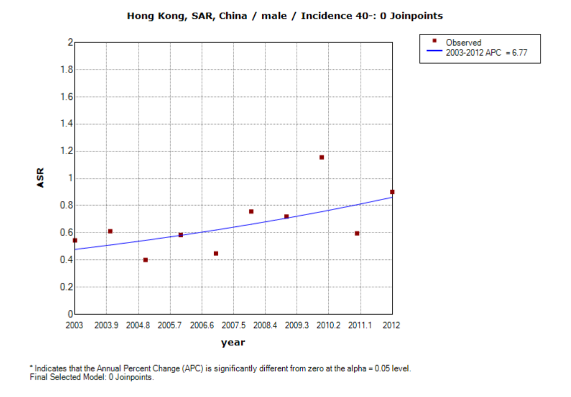

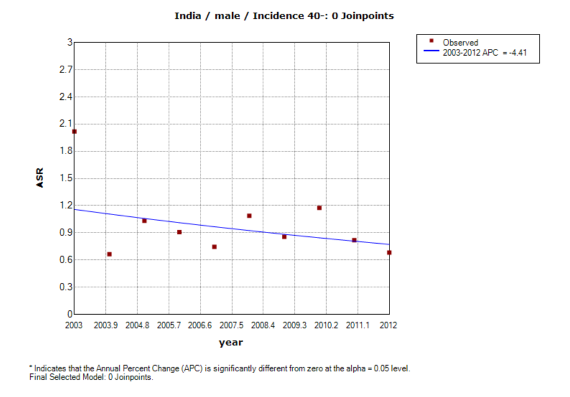

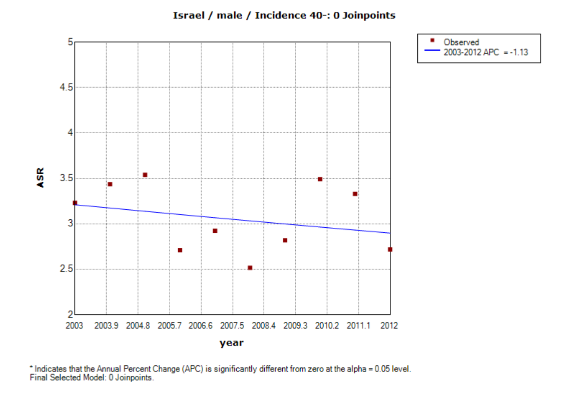

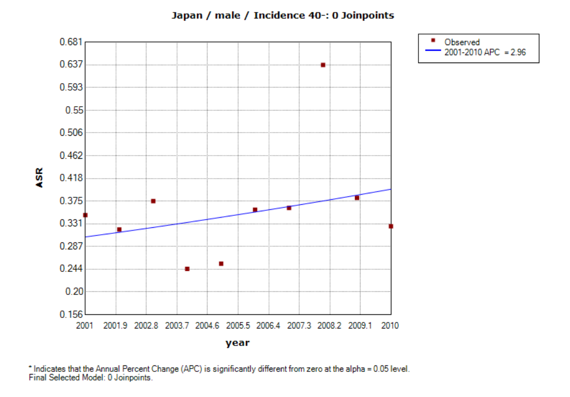

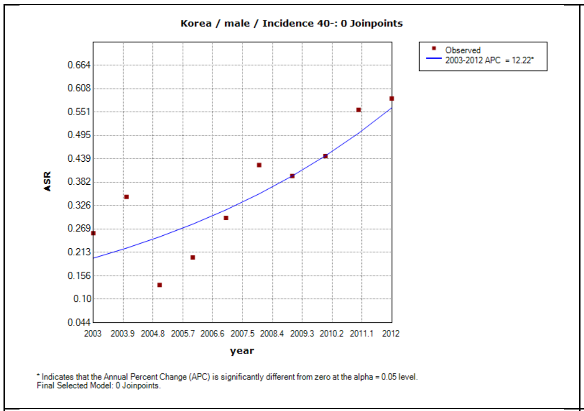
Korea / male / Incidence 40-: 0 Joinpoints
Observed
2003-2012 APC = 12.22*
ASR
0.664
0.608
0.551
0.495
0.439
0.382
0.326
0.269
0.213
0.156
0.10
0.044
2003
2003.9
2004.8
2005.7
2006.6
2007.5
2008.4
2009.3
2010.2
2011.1
2012
year
* Indicates that the Annual Percent Change (APC) is significantly different from zero at the alpha = 0.05 level.
Final Selected Model: 0 Joinpoints.

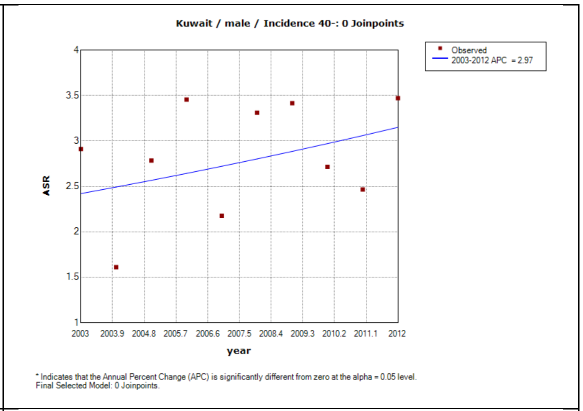
Kuwait / male / Incidence 40-: 0 Joinpoints
Observed
2003-2012 APC = 2.97
ASR
4
3.5
3
2.5
2
1.5
1
2003
2003.9
2004.8
2005.7
2006.6
2007.5
2008.4
2009.3
2010.2
2011.1
2012
year
* Indicates that the Annual Percent Change (APC) is significantly different from zero at the alpha = 0.05 level.
Final Selected Model: 0 Joinpoints.

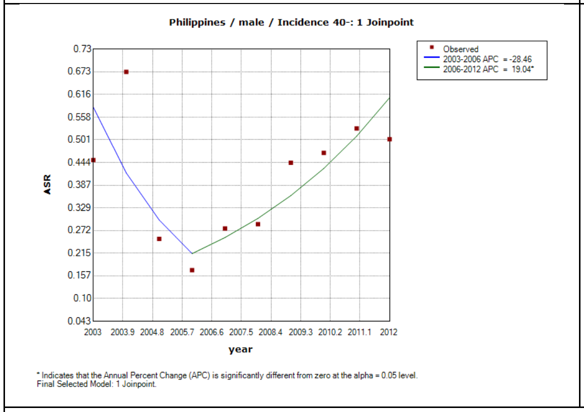
Philippines / male / Incidence 40-: 1 Joinpoint
Observed
2003-2006 APC = -28.46
2006-2012 APC = 19.04*
ASR
0.73
0.673
0.616
0.558
0.501
0.444
0.387
0.329
0.272
0.215
0.157
0.10
0.043
2003
2003.9
2004.8
2005.7
2006.6
2007.5
2008.4
2009.3
2010.2
2011.1
2012
year
* Indicates that the Annual Percent Change (APC) is significantly different from zero at the alpha = 0.05 level.
Final Selected Model: 1 Joinpoint.

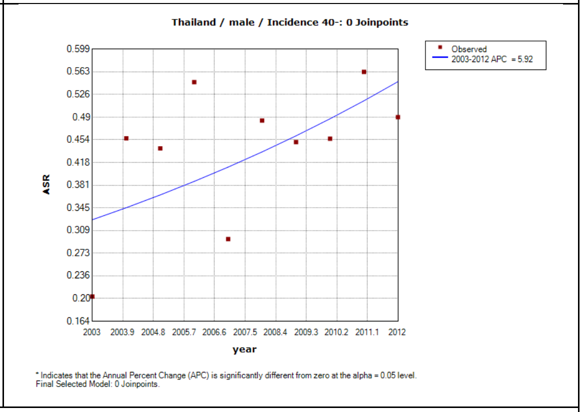
Thailand / male / Incidence 40-: 0 Joinpoints
Observed
2003-2012 APC = 5.92
ASR
0.599
0.563
0.526
0.49
0.454
0.418
0.381
0.345
0.309
0.273
0.236
0.20
0.164
2003
2003.9
2004.8
2005.7
2006.6
2007.5
2008.4
2009.3
2010.2
2011.1
2012
year
* Indicates that the Annual Percent Change (APC) is significantly different from zero at the alpha = 0.05 level.
Final Selected Model: 0 Joinpoints.

Oceania

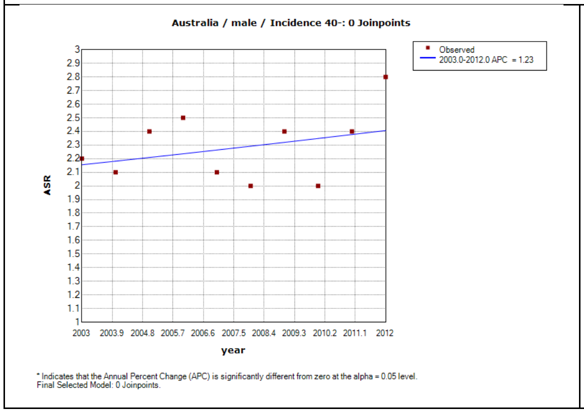
Australia / male / Incidence 40-: 0 Joinpoints
Observed
2003.0-2012.0 APC = 1.23
ASR
3
2.9
2.8
2.7
2.6
2.5
2.4
2.3
2.2
2.1
2
1.9
1.8
1.7
1.6
1.5
1.4
1.3
1.2
1.1
1
2003
2003.9
2004.8
2005.7
2006.6
2007.5
2008.4
2009.3
2010.2
2011.1
2012
year
* Indicates that the Annual Percent Change (APC) is significantly different from zero at the alpha = 0.05 level.
Final Selected Model: 0 Joinpoints.

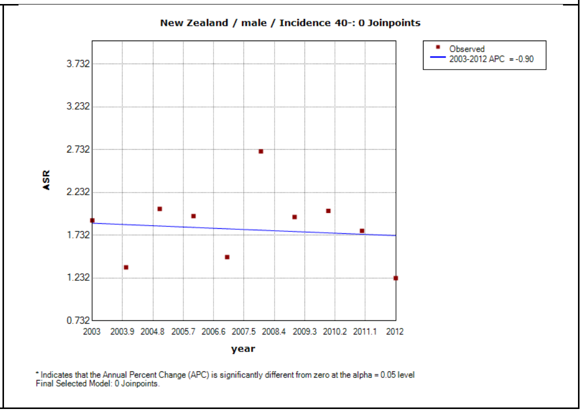
New Zealand / male / Incidence 40-: 0 Joinpoints
Observed
2003-2012 APC = -0.90
ASR
3.732
3.232
2.732
2.232
1.732
1.232
0.732
2003
2003.9
2004.8
2005.7
2006.6
2007.5
2008.4
2009.3
2010.2
2011.1
2012
year
* Indicates that the Annual Percent Change (APC) is significantly different from zero at the alpha = 0.05 level
Final Selected Model: 0 Joinpoints.

North America

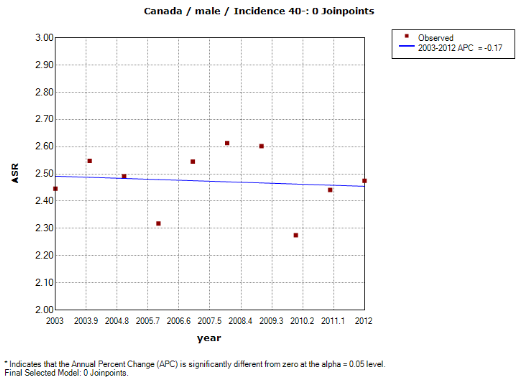

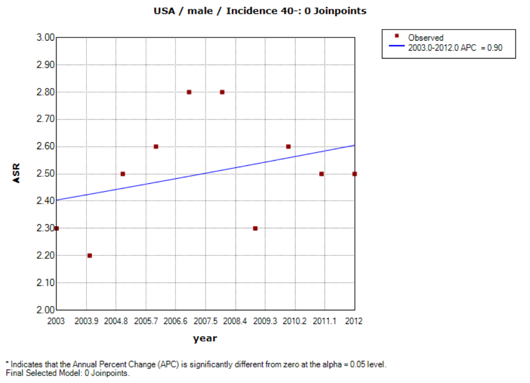

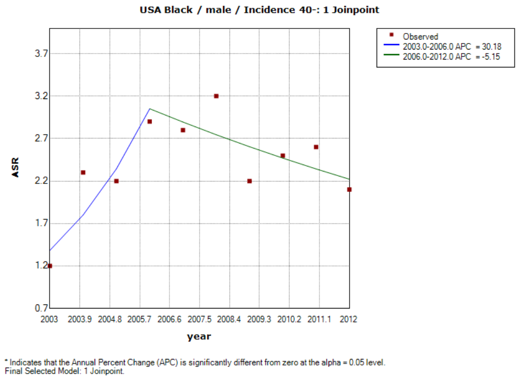

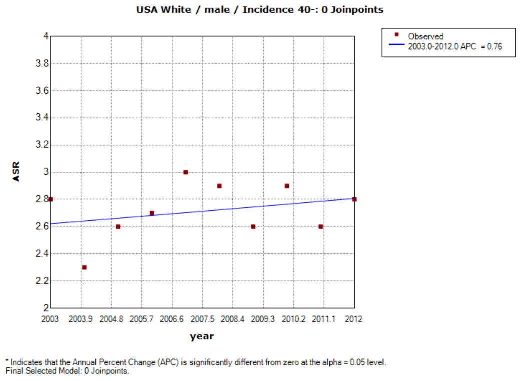

South America

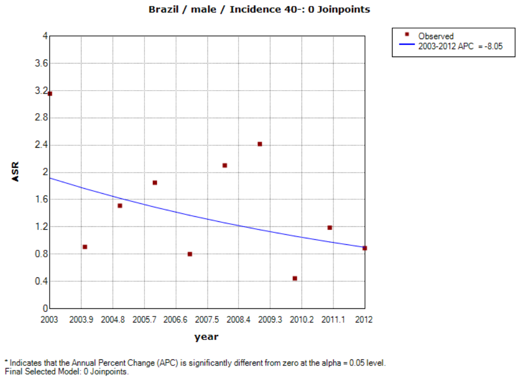

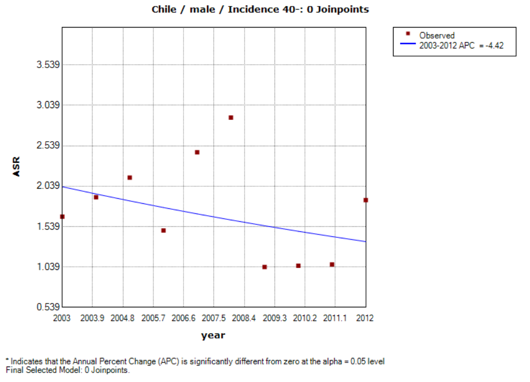

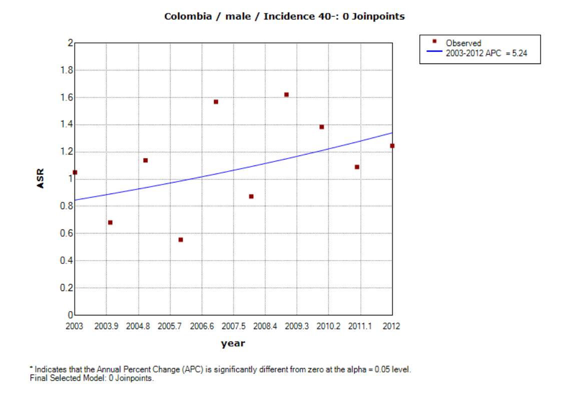

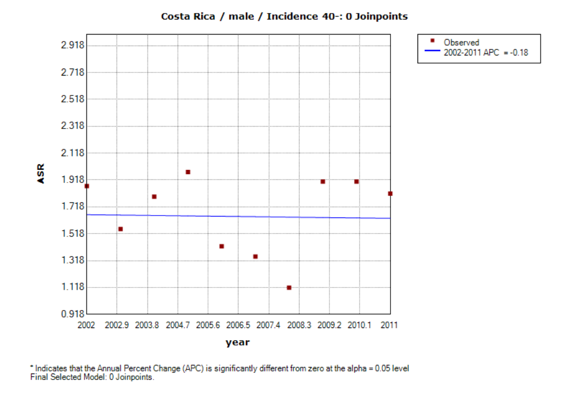

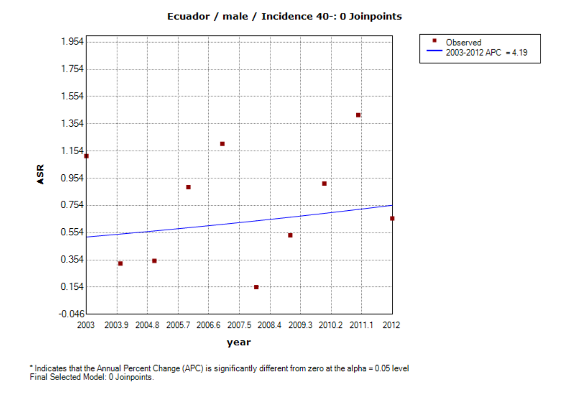

Northern Europe

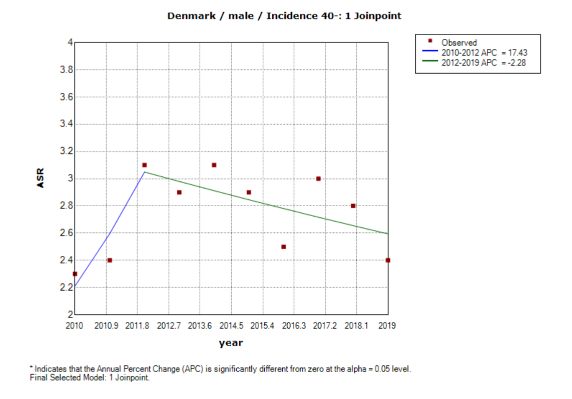

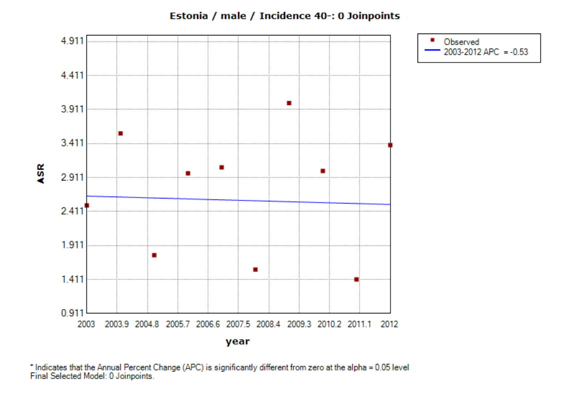

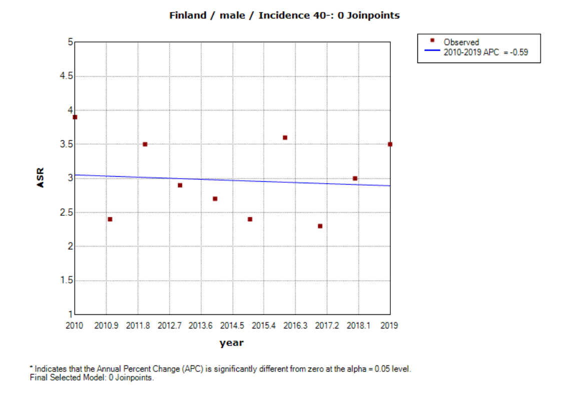

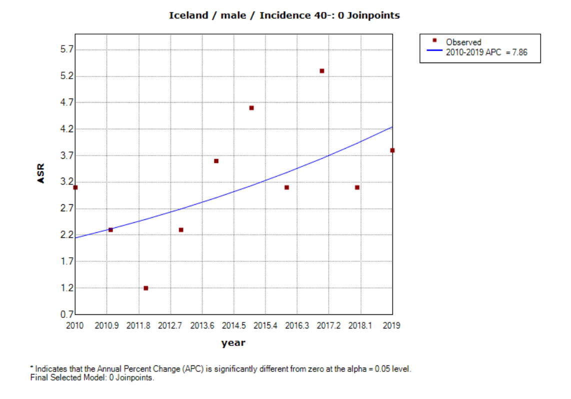

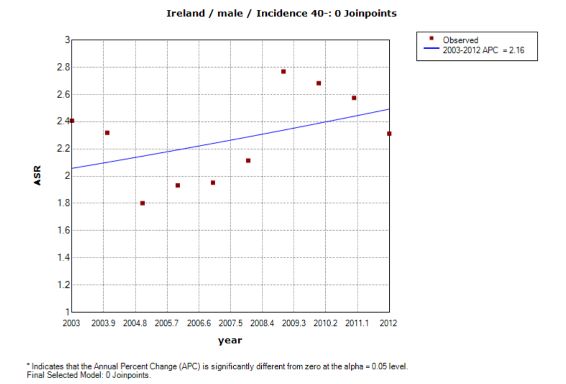

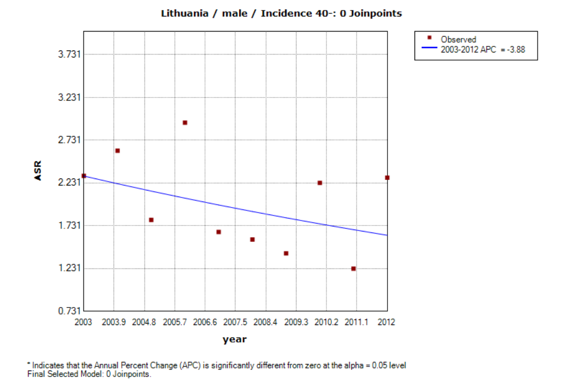

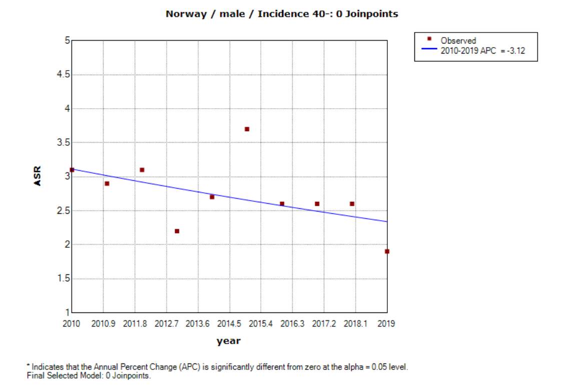

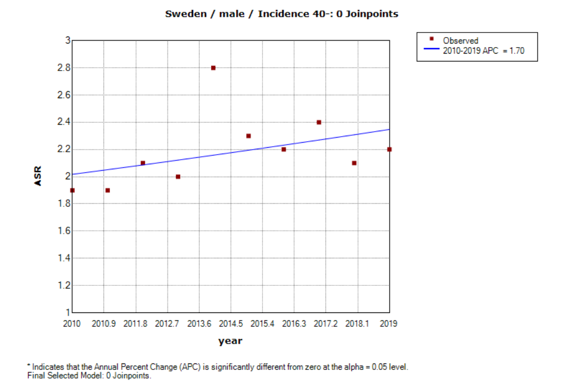

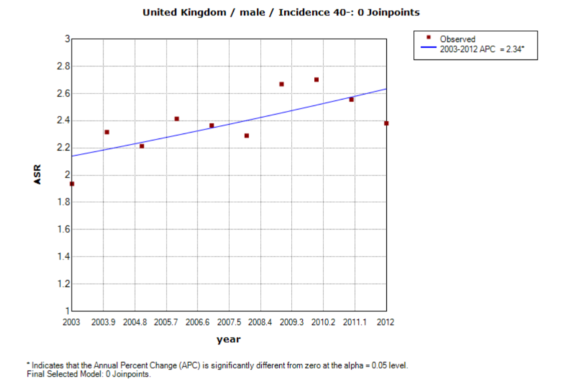

Western Europe

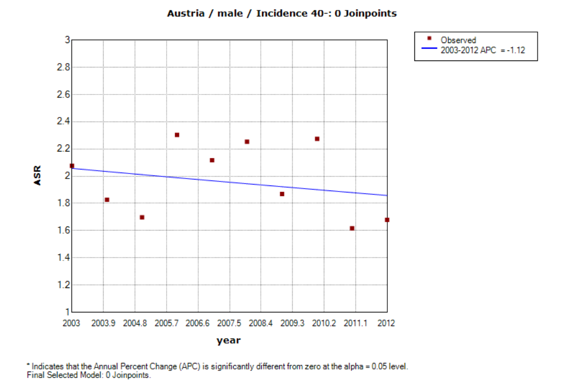

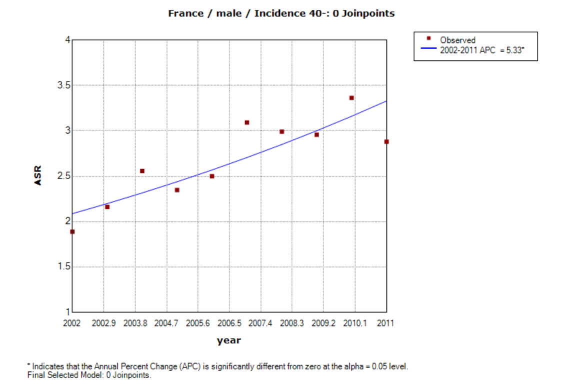

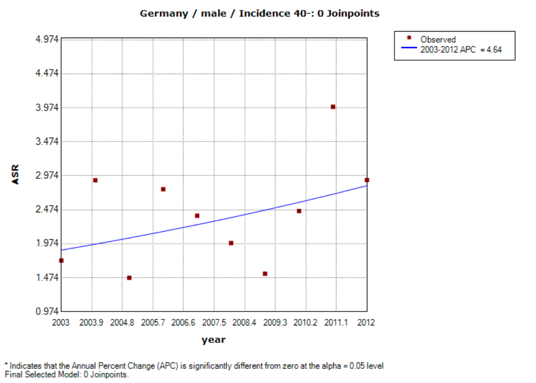

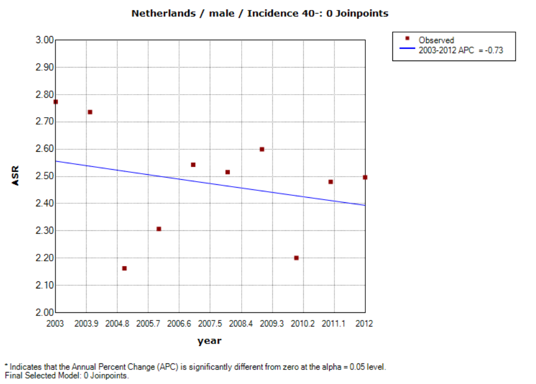

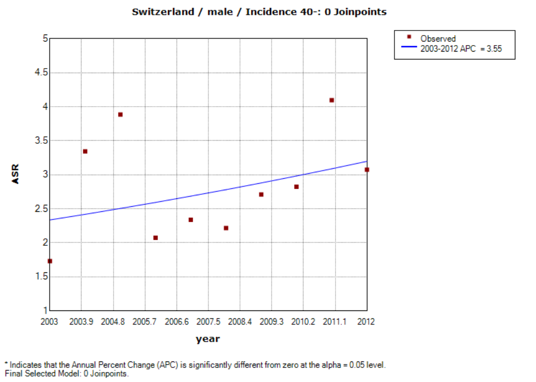

Southern Europe

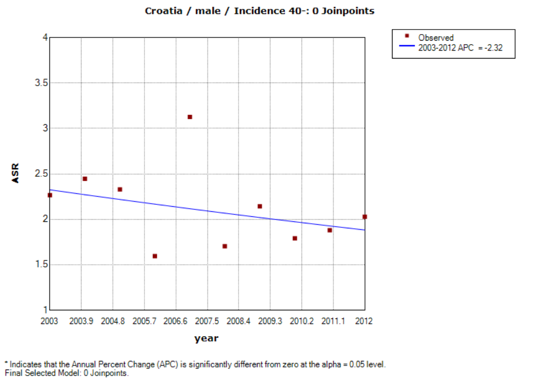

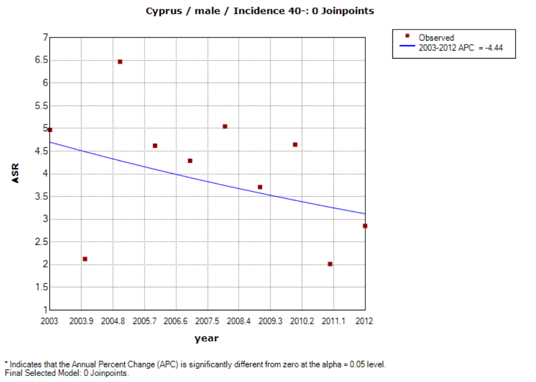

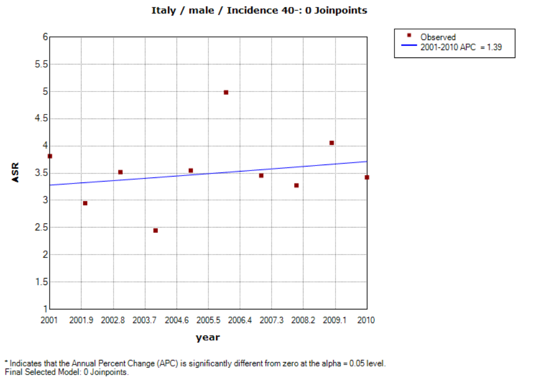

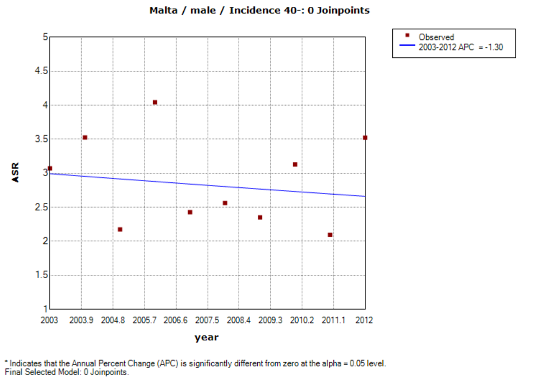

Slovenia / male / Incidence 40-: 0 Joinpoints

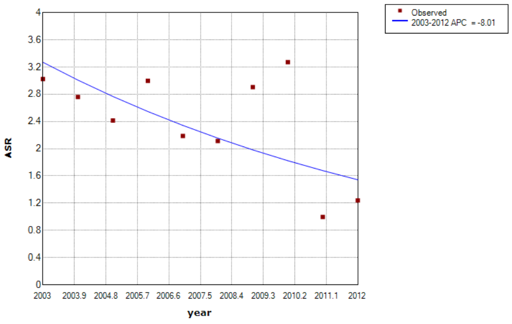

* Indicates that the Annual Percent Change (APC) is significantly different from zero at the alpha = 0.05 level.
Final Selected Model: 0 Joinpoints.

Spain / male / Incidence 40-: 0 Joinpoints

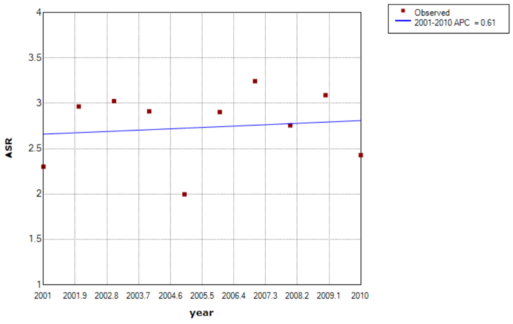

* Indicates that the Annual Percent Change (APC) is significantly different from zero at the alpha = 0.05 level.
Final Selected Model: 0 Joinpoints.

Turkey / male / Incidence 40-: 0 Joinpoints

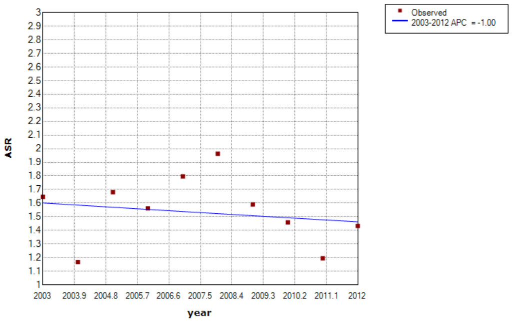

* Indicates that the Annual Percent Change (APC) is significantly different from zero at the alpha = 0.05 level.
Final Selected Model: 0 Joinpoints.

Eastern Europe

Belarus / male / Incidence 40-: 0 Joinpoints

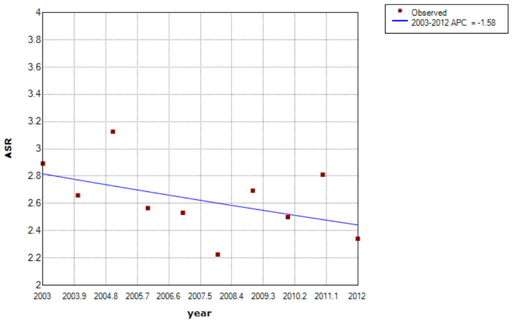

* Indicates that the Annual Percent Change (APC) is significantly different from zero at the alpha = 0.05 level.
Final Selected Model: 0 Joinpoints.

Bulgaria / male / Incidence 40-: 0 Joinpoints

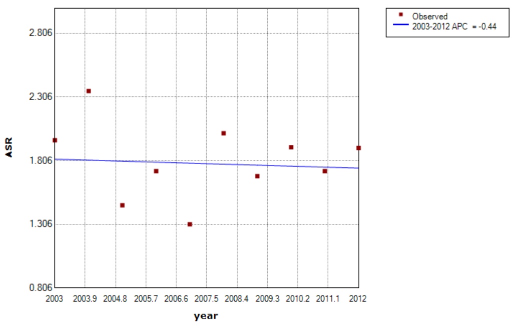

* Indicates that the Annual Percent Change (APC) is significantly different from zero at the alpha = 0.05 level
Final Selected Model: 0 Joinpoints.

Czech Republic / male / Incidence 40-: 0 Joinpoints

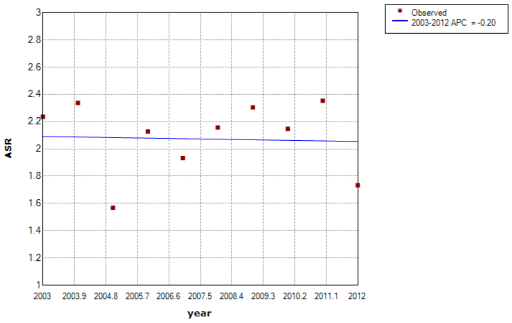

* Indicates that the Annual Percent Change (APC) is significantly different from zero at the alpha = 0.05 level.
Final Selected Model: 0 Joinpoints.

Poland / male / Incidence 40-: 0 Joinpoints

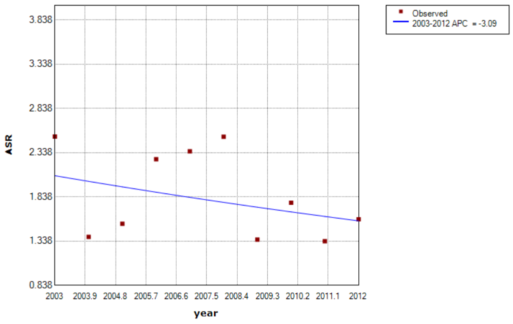

* Indicates that the Annual Percent Change (APC) is significantly different from zero at the alpha = 0.05 level
Final Selected Model: 0 Joinpoints.

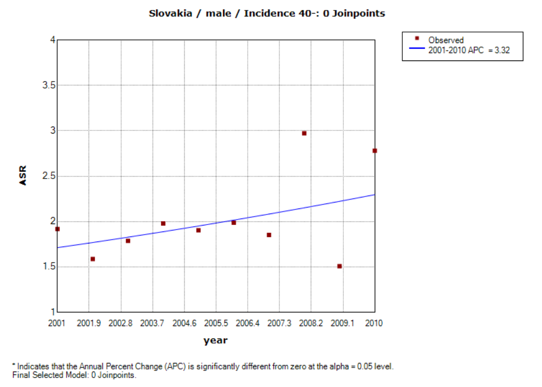
Slovakia / male / Incidence 40-: 0 Joinpoints
Observed
2001-2010 APC = 3.32
ASR
year
* Indicates that the Annual Percent Change (APC) is significantly different from zero at the alpha = 0.05 level.
Final Selected Model: 0 Joinpoints.

Africa

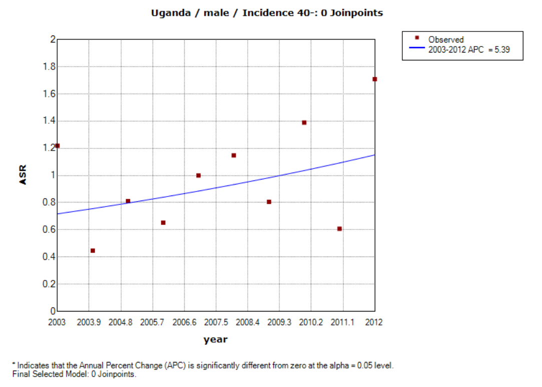
Uganda / male / Incidence 40-: 0 Joinpoints
Observed
2003-2012 APC = 5.39
ASR
year
* Indicates that the Annual Percent Change (APC) is significantly different from zero at the alpha = 0.05 level.
Final Selected Model: 0 Joinpoints.

d.) Incidence female below 40 years old

Asia

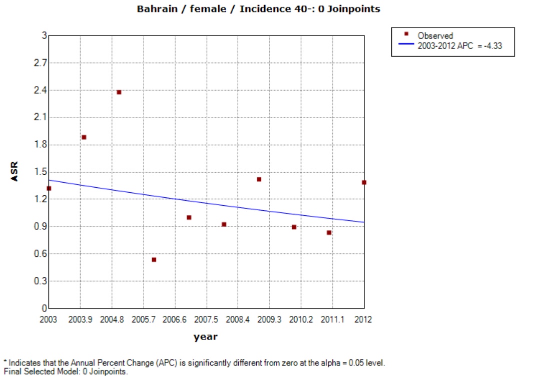

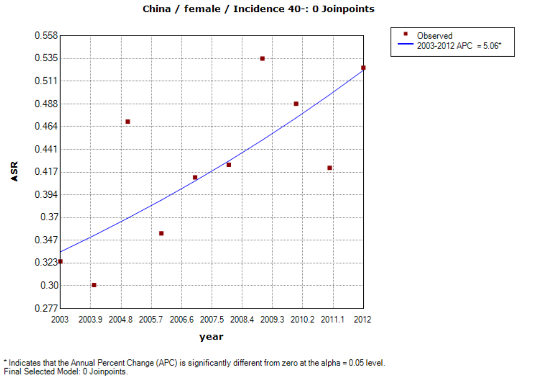

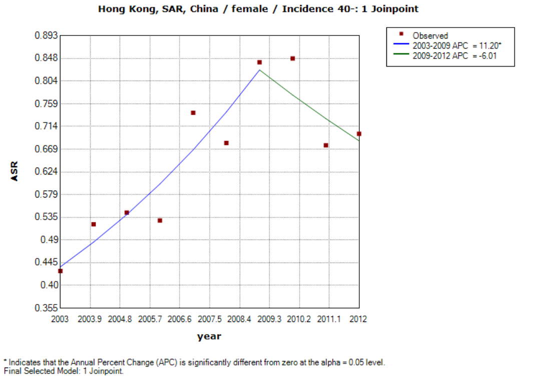

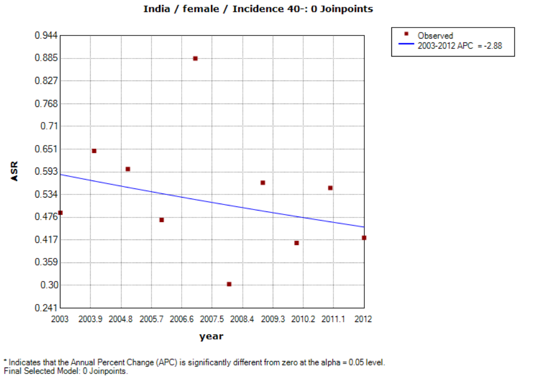

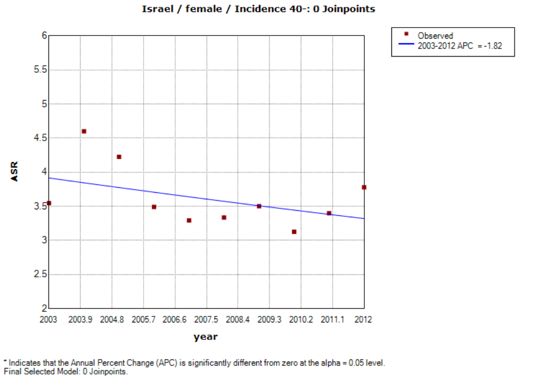

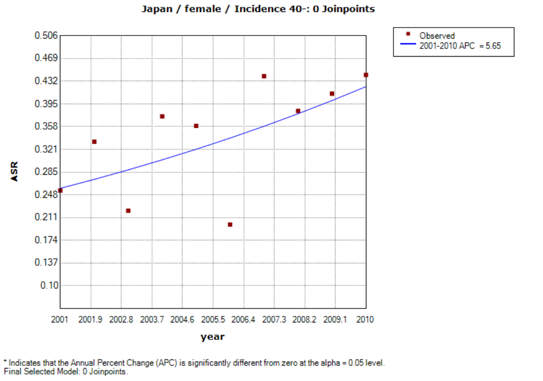

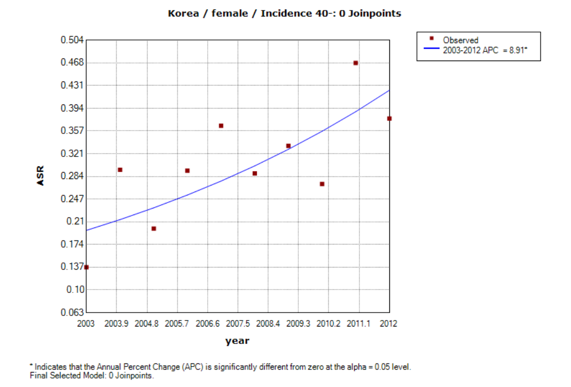
Korea / female / Incidence 40-: 0 Joinpoints
Observed
2003-2012 APC = 8.91*
0.504
0.468
0.431
0.394
0.357
0.321
0.284
0.247
0.21
0.174
0.137
0.10
0.063
ASR
2003 2003.9 2004.8 2005.7 2006.6 2007.5 2008.4 2009.3 2010.2 2011.1 2012
year
* Indicates that the Annual Percent Change (APC) is significantly different from zero at the alpha = 0.05 level.
Final Selected Model: 0 Joinpoints.

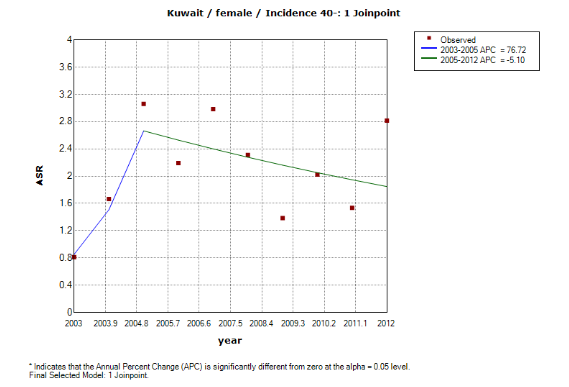
Kuwait / female / Incidence 40-: 1 Joinpoint
Observed
2003-2005 APC = 76.72
2005-2012 APC = -5.10
4
3.6
3.2
2.8
2.4
2
1.6
1.2
0.8
0.4
0
ASR
2003 2003.9 2004.8 2005.7 2006.6 2007.5 2008.4 2009.3 2010.2 2011.1 2012
year
* Indicates that the Annual Percent Change (APC) is significantly different from zero at the alpha = 0.05 level.
Final Selected Model: 1 Joinpoint.

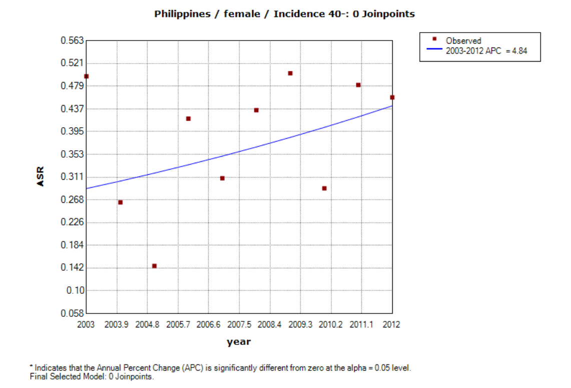
Philippines / female / Incidence 40-: 0 Joinpoints
Observed
2003-2012 APC = 4.84
0.563
0.521
0.479
0.437
0.395
0.353
0.311
0.268
0.226
0.184
0.142
0.10
0.058
ASR
2003 2003.9 2004.8 2005.7 2006.6 2007.5 2008.4 2009.3 2010.2 2011.1 2012
year
* Indicates that the Annual Percent Change (APC) is significantly different from zero at the alpha = 0.05 level.
Final Selected Model: 0 Joinpoints.

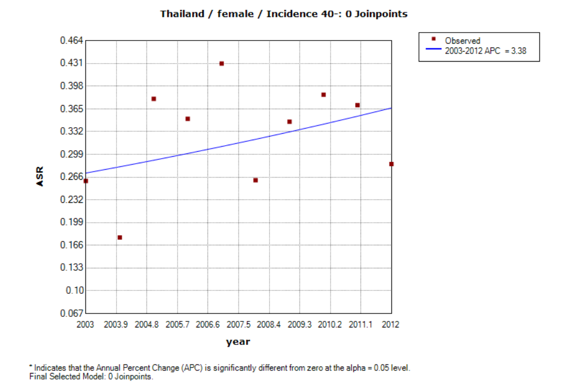
Thailand / female / Incidence 40-: 0 Joinpoints
Observed
2003-2012 APC = 3.38
0.464
0.431
0.398
0.365
0.332
0.299
0.266
0.232
0.199
0.166
0.133
0.10
0.067
ASR
2003 2003.9 2004.8 2005.7 2006.6 2007.5 2008.4 2009.3 2010.2 2011.1 2012
year
* Indicates that the Annual Percent Change (APC) is significantly different from zero at the alpha = 0.05 level.
Final Selected Model: 0 Joinpoints.

Oceania

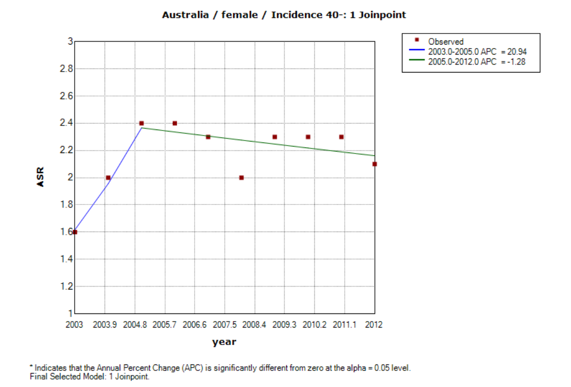
Australia / female / Incidence 40-: 1 Joinpoint
Observed
2003.0-2005.0 APC = 20.94
2005.0-2012.0 APC = -1.28
3
2.8
2.6
2.4
2.2
2
1.8
1.6
1.4
1.2
1
ASR
2003 2003.9 2004.8 2005.7 2006.6 2007.5 2008.4 2009.3 2010.2 2011.1 2012
year
* Indicates that the Annual Percent Change (APC) is significantly different from zero at the alpha = 0.05 level.
Final Selected Model: 1 Joinpoint.

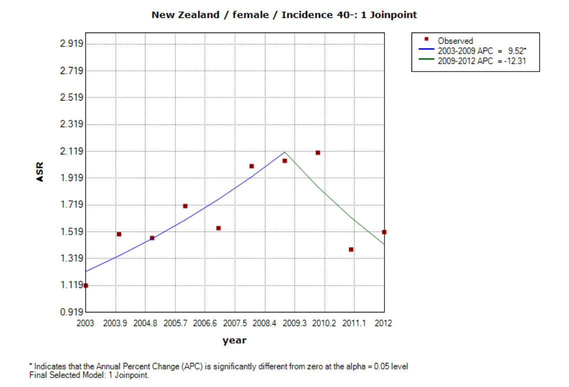
New Zealand / female / Incidence 40-: 1 Joinpoint
Observed
2003-2009 APC = 9.52*
2009-2012 APC = -12.31
2.919
2.719
2.519
2.319
2.119
1.919
1.719
1.519
1.319
1.119
0.919
ASR
2003 2003.9 2004.8 2005.7 2006.6 2007.5 2008.4 2009.3 2010.2 2011.1 2012
year
* Indicates that the Annual Percent Change (APC) is significantly different from zero at the alpha = 0.05 level
Final Selected Model: 1 Joinpoint.

North America

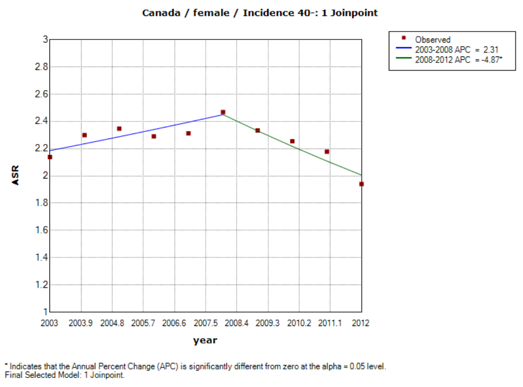

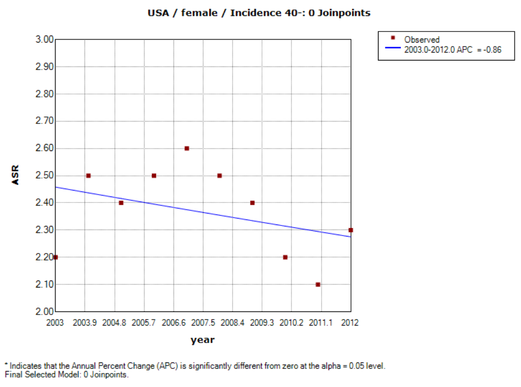

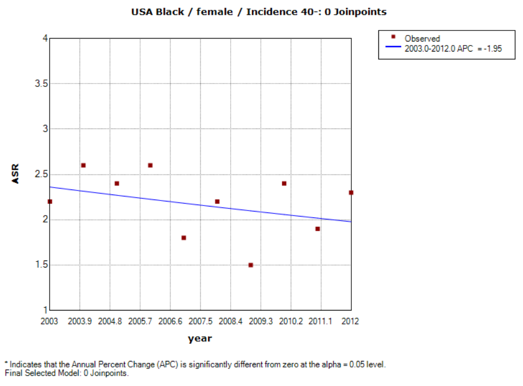

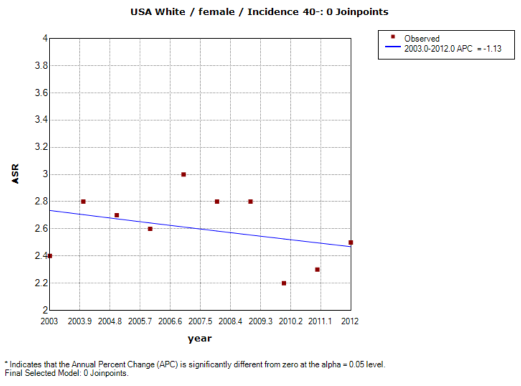

South America

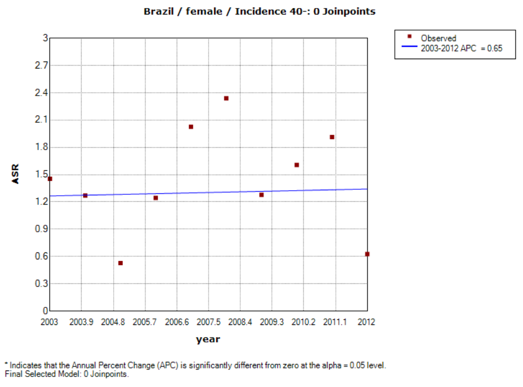

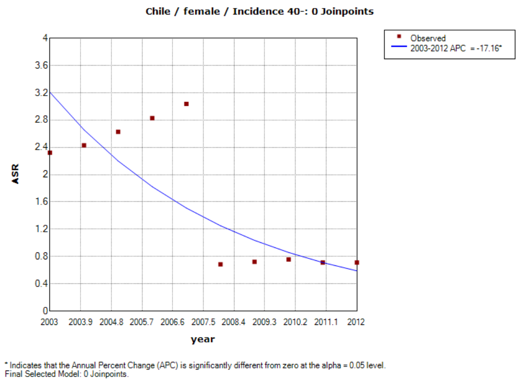

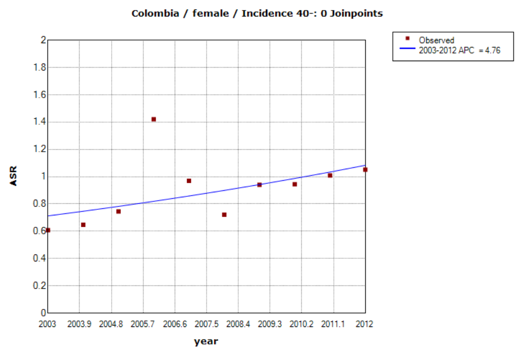

* Indicates that the Annual Percent Change (APC) is significantly different from zero at the alpha = 0.05 level.
Final Selected Model: 0 Joinpoints.

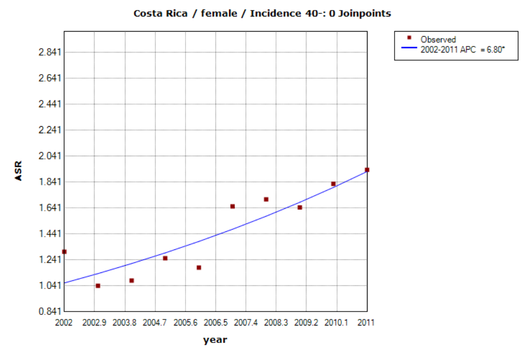

* Indicates that the Annual Percent Change (APC) is significantly different from zero at the alpha = 0.05 level
Final Selected Model: 0 Joinpoints.

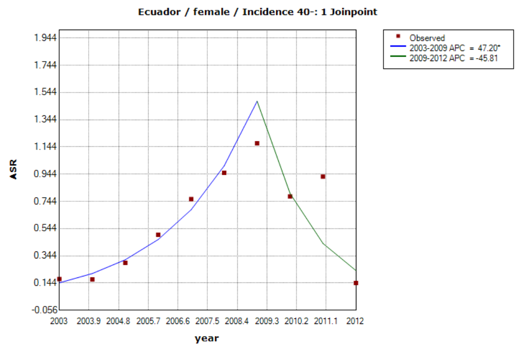

* Indicates that the Annual Percent Change (APC) is significantly different from zero at the alpha = 0.05 level
Final Selected Model: 1 Joinpoint.

Northern Europe

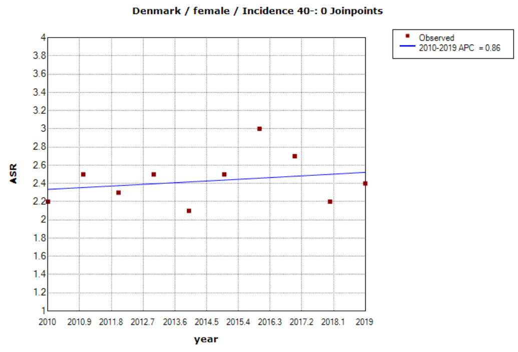

* Indicates that the Annual Percent Change (APC) is significantly different from zero at the alpha = 0.05 level.
Final Selected Model: 0 Joinpoints.

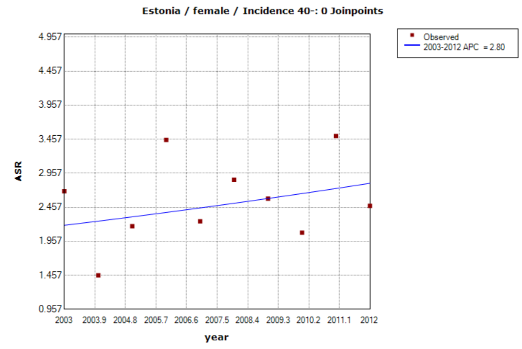

* Indicates that the Annual Percent Change (APC) is significantly different from zero at the alpha = 0.05 level
Final Selected Model: 0 Joinpoints.

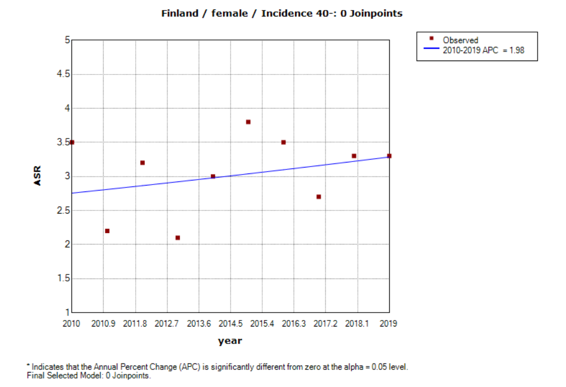

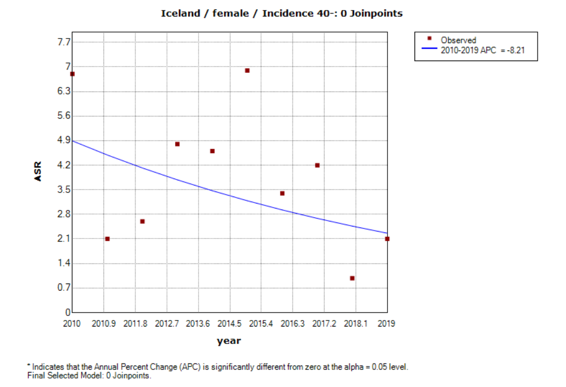

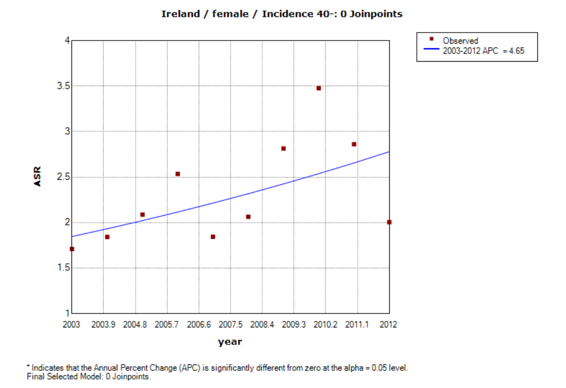

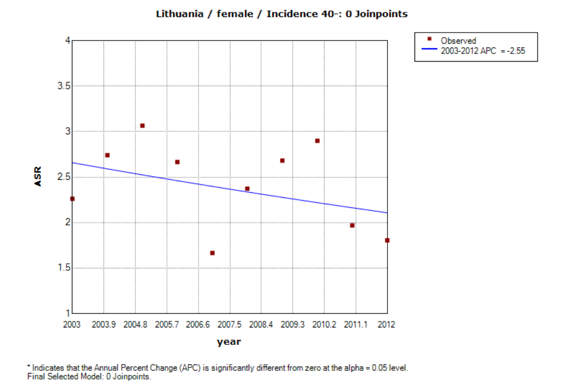

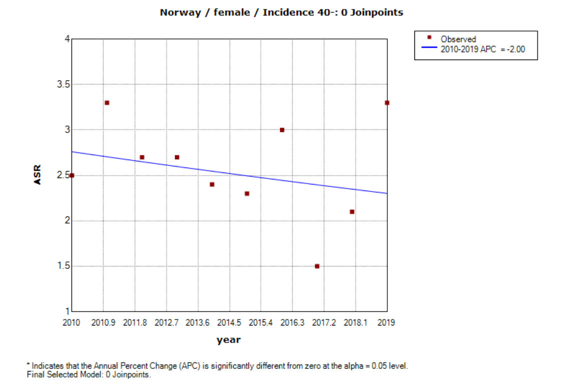

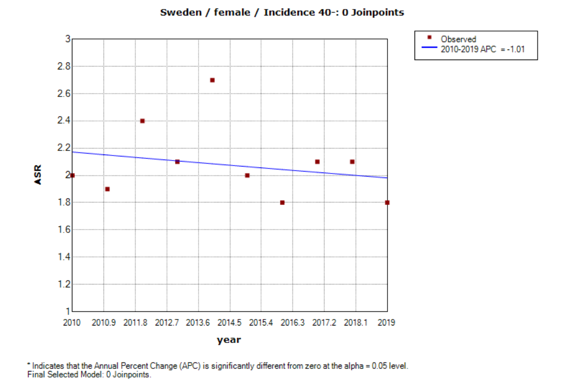

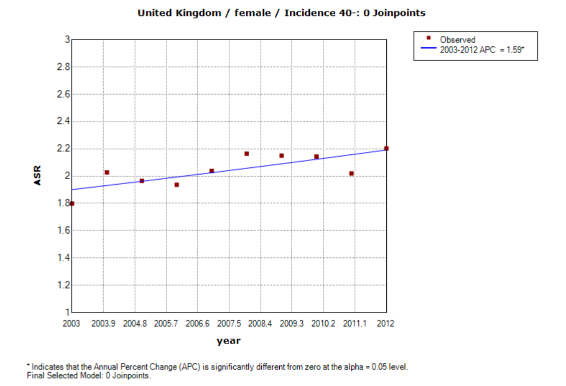

Western Europe

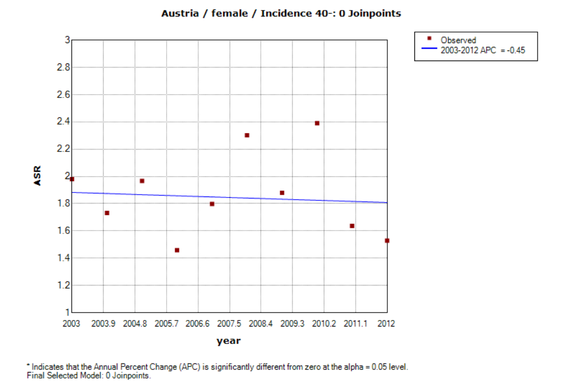

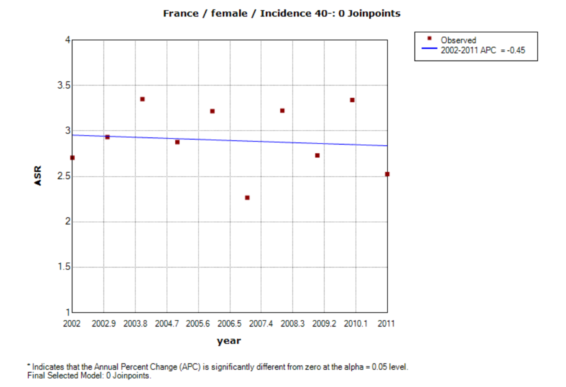

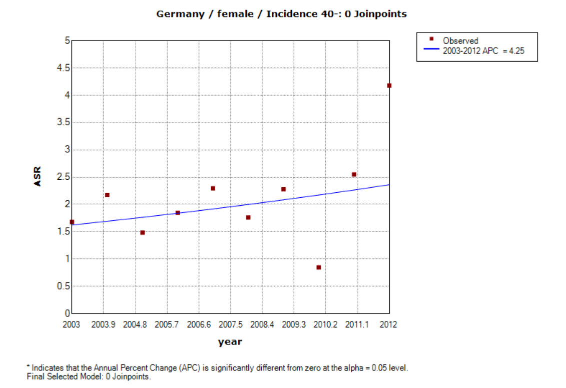

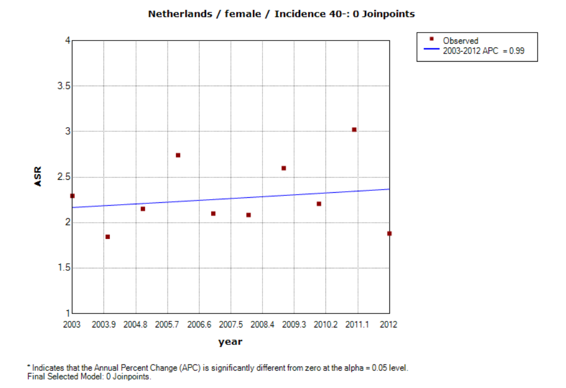

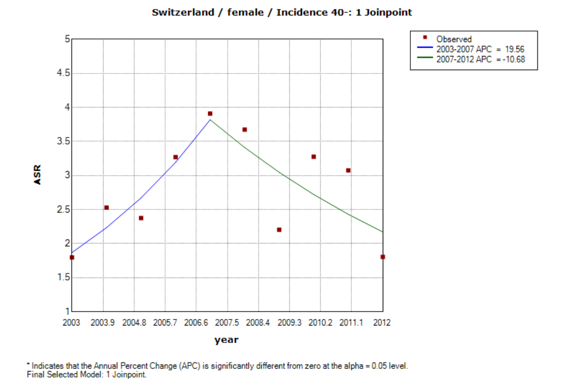

Southern Europe

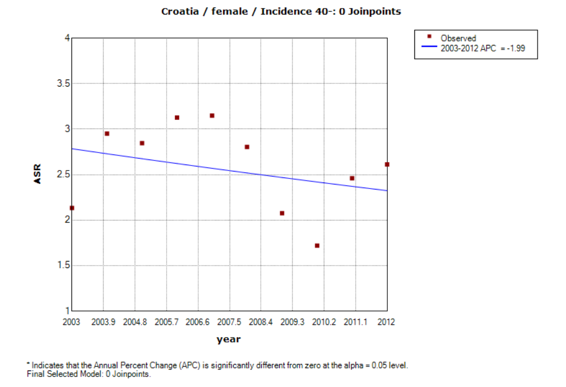

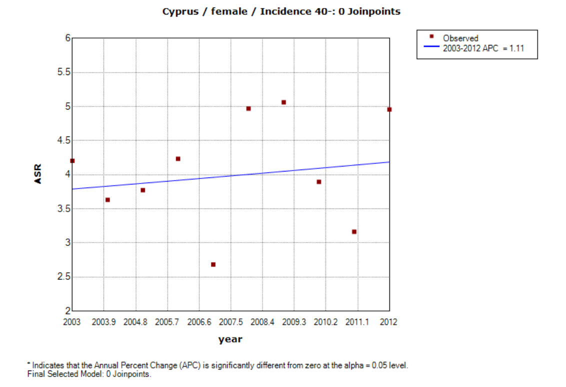

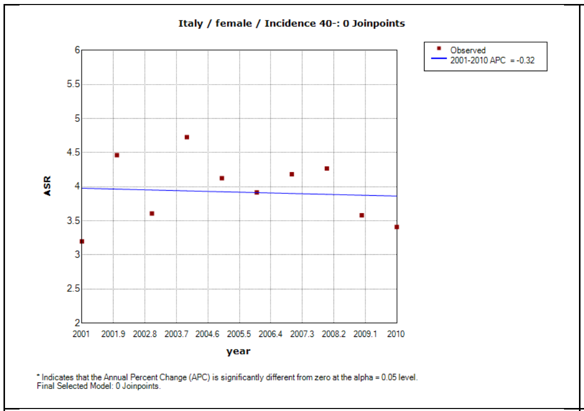

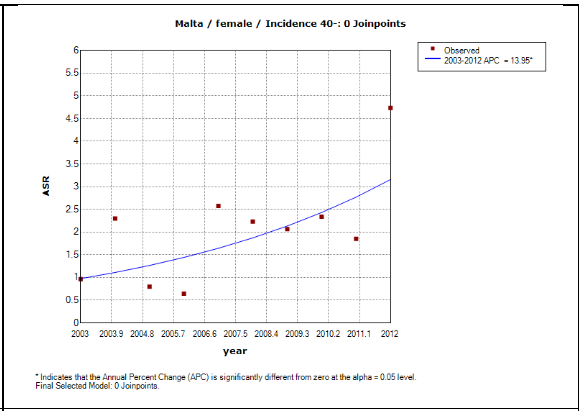

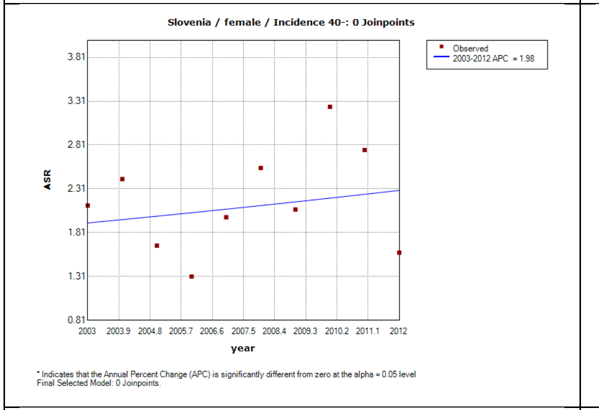

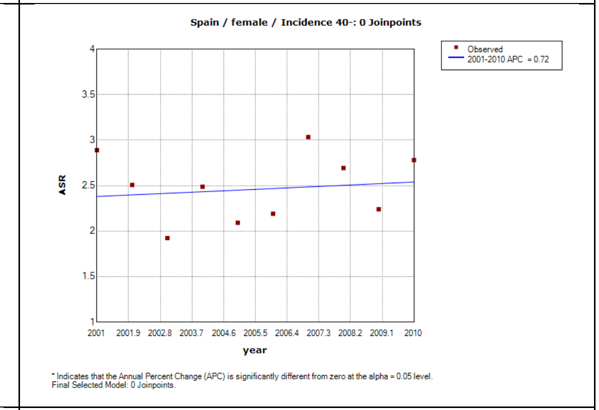

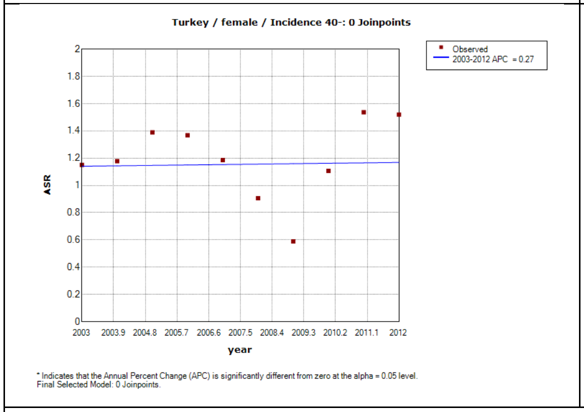

Eastern Europe

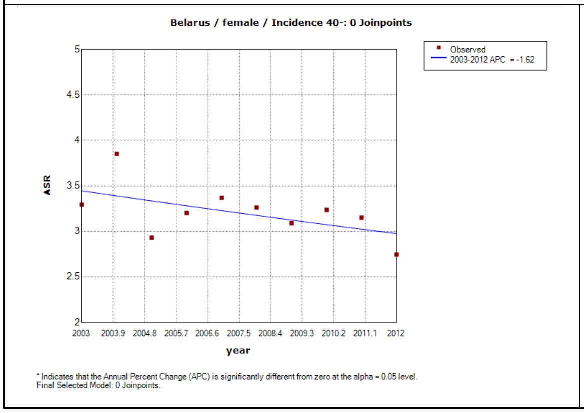

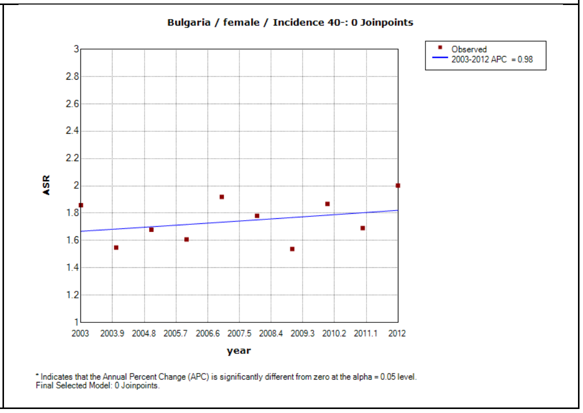

Czech Republic / female / Incidence 40-: 0 Joinpoints

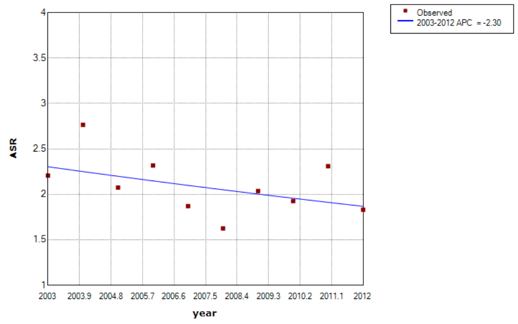

* Indicates that the Annual Percent Change (APC) is significantly different from zero at the alpha = 0.05 level.
Final Selected Model: 0 Joinpoints.

Poland / female / Incidence 40-: 0 Joinpoints

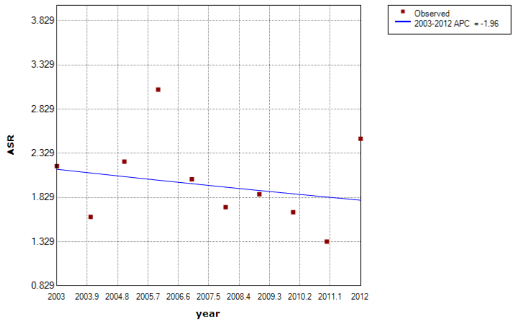

* Indicates that the Annual Percent Change (APC) is significantly different from zero at the alpha = 0.05 level.
Final Selected Model: 0 Joinpoints.

Slovakia / female / Incidence 40-: 0 Joinpoints

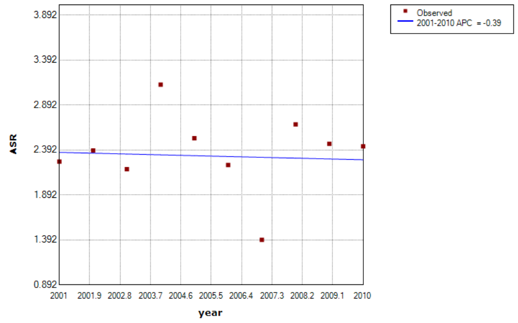

* Indicates that the Annual Percent Change (APC) is significantly different from zero at the alpha = 0.05 level.
Final Selected Model: 0 Joinpoints.

Africa

Uganda / female / Incidence 40-: 0 Joinpoints

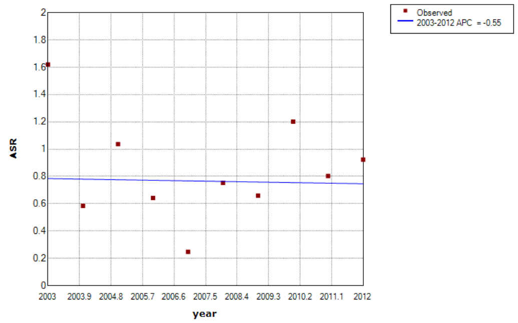

* Indicates that the Annual Percent Change (APC) is significantly different from zero at the alpha = 0.05 level.
Final Selected Model: 0 Joinpoints.

e.) Incidence male below 50 years old

Asia

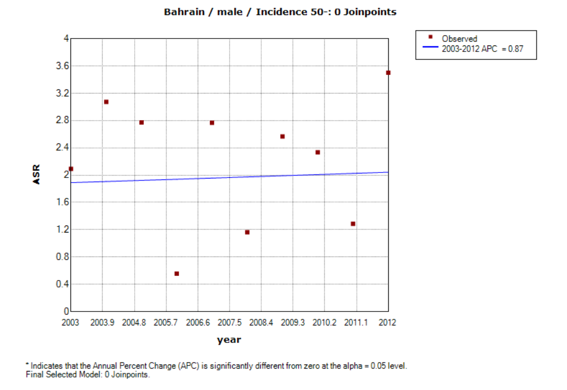

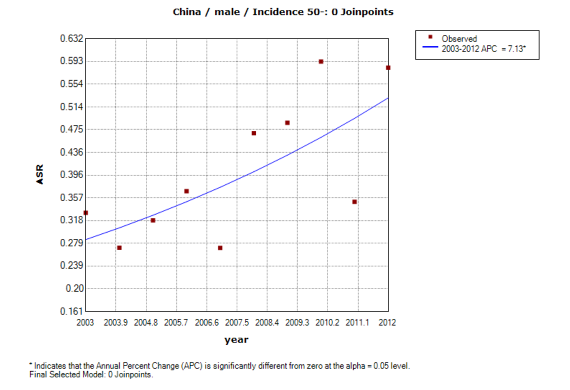

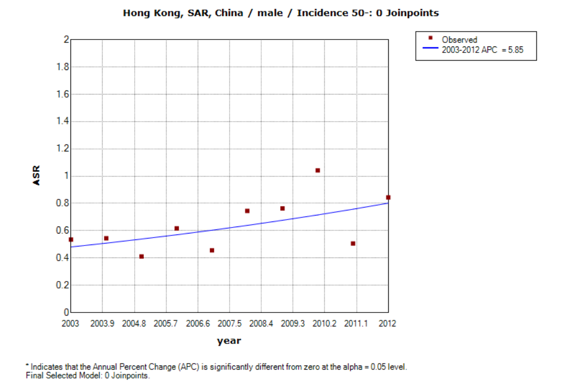

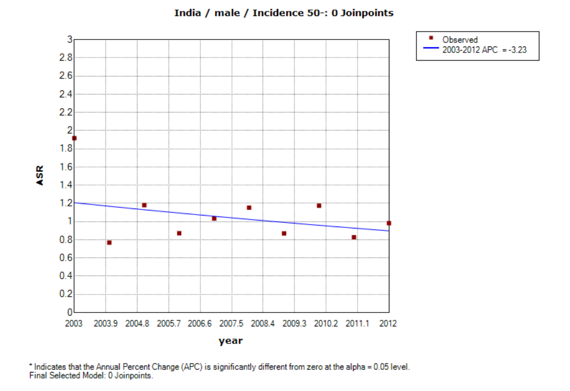

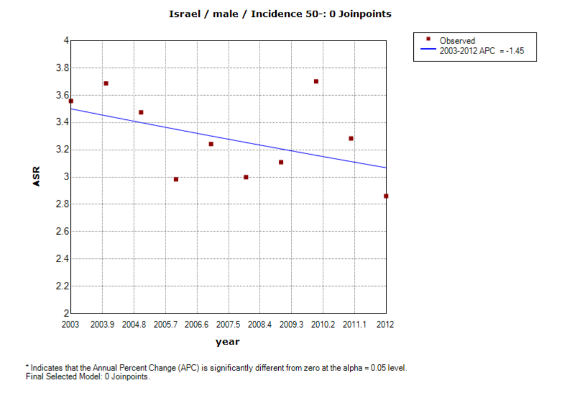

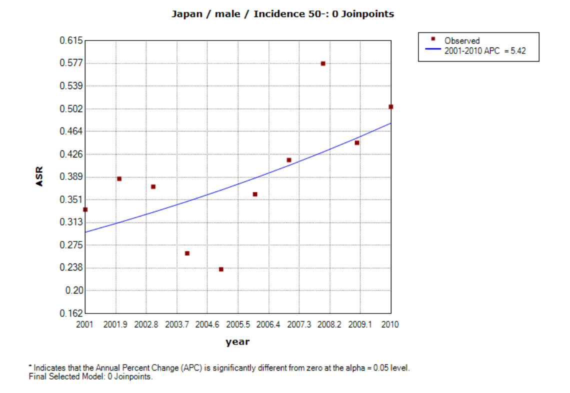

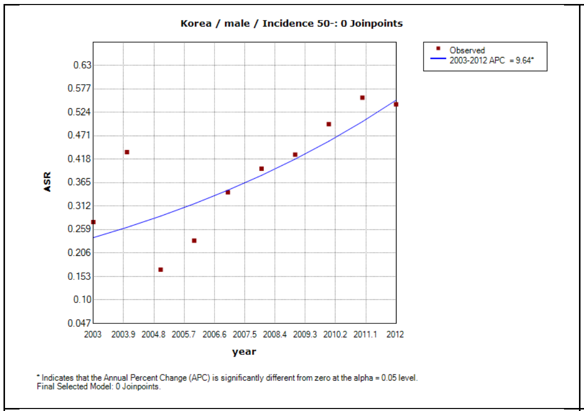
Korea / male / Incidence 50-: 0 Joinpoints
Observed
2003-2012 APC = 9.64*
ASR
year
* Indicates that the Annual Percent Change (APC) is significantly different from zero at the alpha = 0.05 level.
Final Selected Model: 0 Joinpoints.

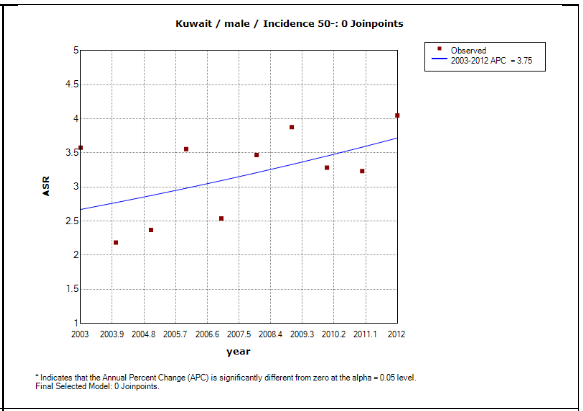
Kuwait / male / Incidence 50-: 0 Joinpoints
Observed
2003-2012 APC = 3.75
ASR
year
* Indicates that the Annual Percent Change (APC) is significantly different from zero at the alpha = 0.05 level.
Final Selected Model: 0 Joinpoints.

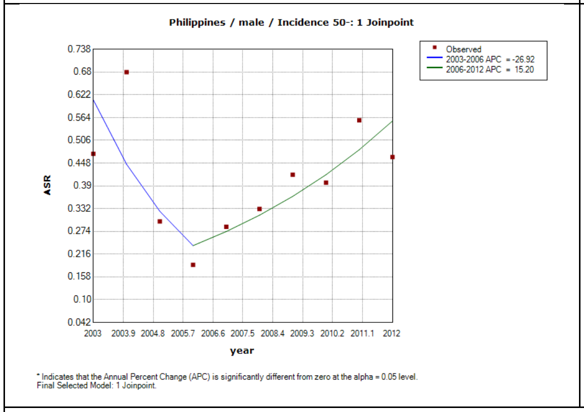
Philippines / male / Incidence 50-: 1 Joinpoint
Observed
2003-2006 APC = -26.92
2006-2012 APC = 15.20
ASR
year
* Indicates that the Annual Percent Change (APC) is significantly different from zero at the alpha = 0.05 level.
Final Selected Model: 1 Joinpoint.

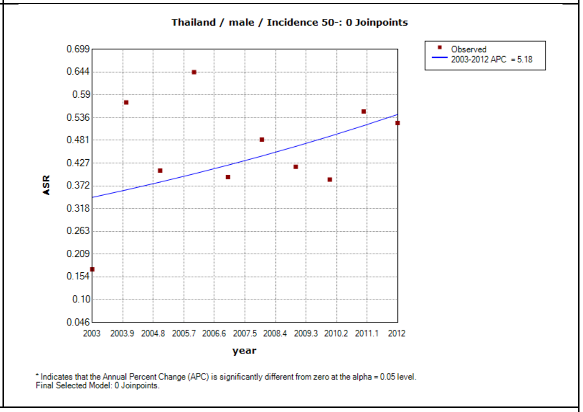
Thailand / male / Incidence 50-: 0 Joinpoints
Observed
2003-2012 APC = 5.18
ASR
year
* Indicates that the Annual Percent Change (APC) is significantly different from zero at the alpha = 0.05 level.
Final Selected Model: 0 Joinpoints.

Oceania

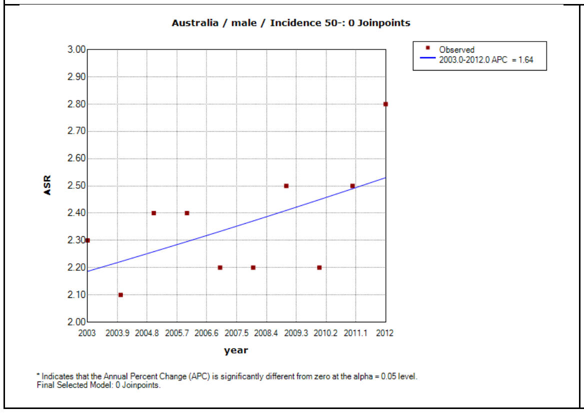
Australia / male / Incidence 50-: 0 Joinpoints
Observed
2003.0-2012.0 APC = 1.64
ASR
year
* Indicates that the Annual Percent Change (APC) is significantly different from zero at the alpha = 0.05 level.
Final Selected Model: 0 Joinpoints.

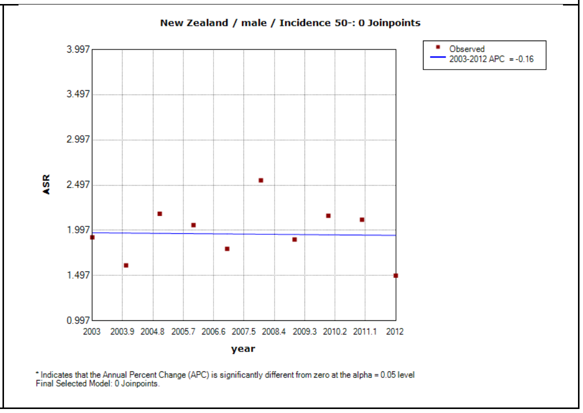
New Zealand / male / Incidence 50-: 0 Joinpoints
Observed
2003-2012 APC = -0.16
ASR
year
* Indicates that the Annual Percent Change (APC) is significantly different from zero at the alpha = 0.05 level.
Final Selected Model: 0 Joinpoints.

North America

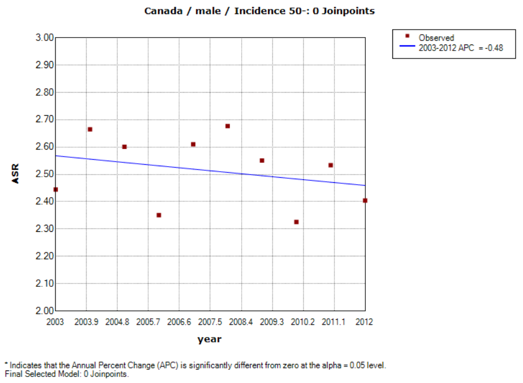

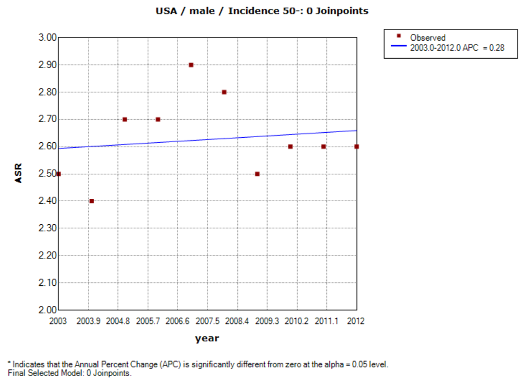

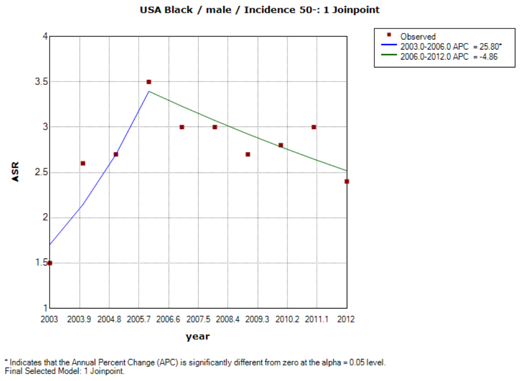

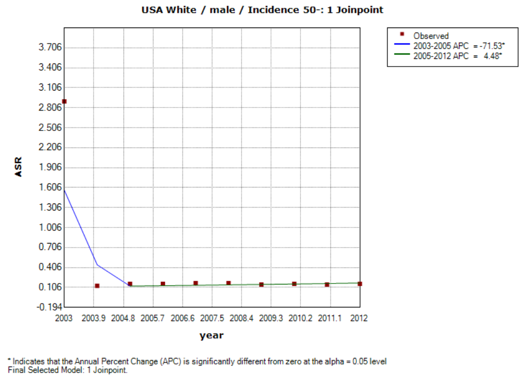

South America

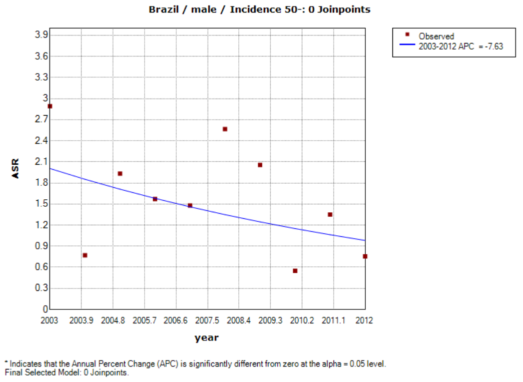

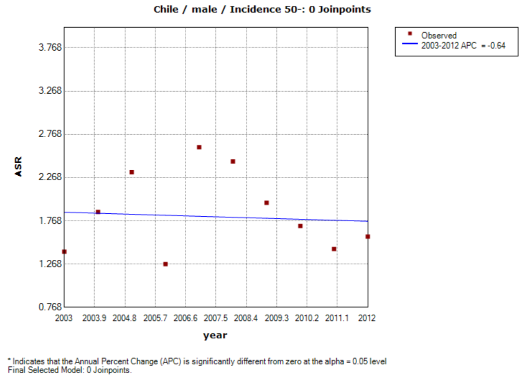

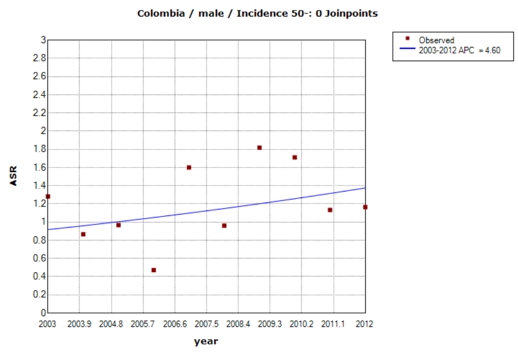

* Indicates that the Annual Percent Change (APC) is significantly different from zero at the alpha = 0.05 level.
Final Selected Model: 0 Joinpoints.

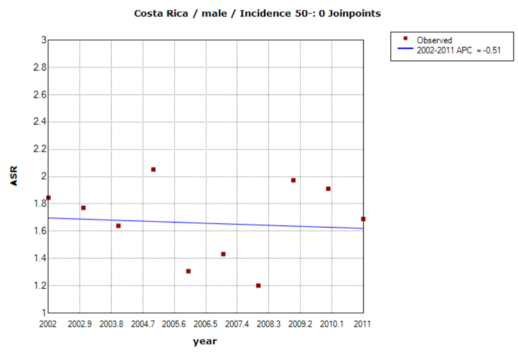

* Indicates that the Annual Percent Change (APC) is significantly different from zero at the alpha = 0.05 level.
Final Selected Model: 0 Joinpoints.

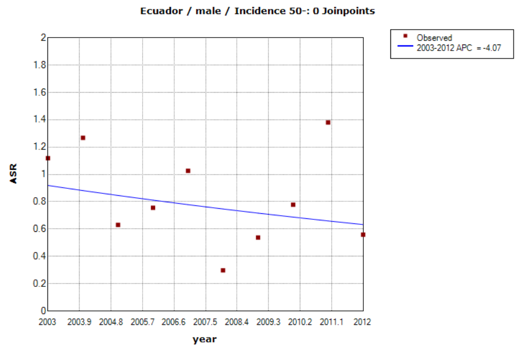

* Indicates that the Annual Percent Change (APC) is significantly different from zero at the alpha = 0.05 level.
Final Selected Model: 0 Joinpoints.

Northern Europe

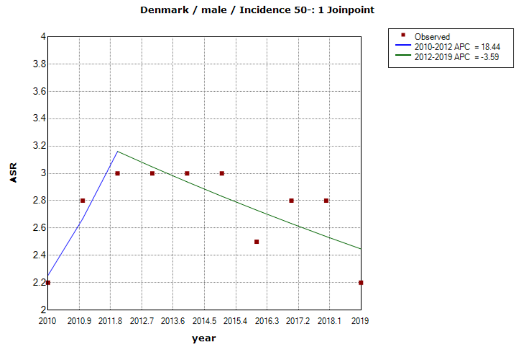

* Indicates that the Annual Percent Change (APC) is significantly different from zero at the alpha = 0.05 level.
Final Selected Model: 1 Joinpoint.

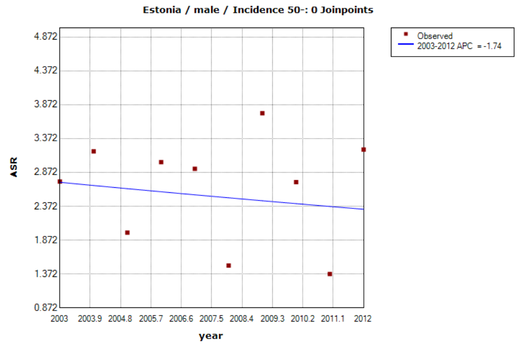

* Indicates that the Annual Percent Change (APC) is significantly different from zero at the alpha = 0.05 level.
Final Selected Model: 0 Joinpoints.

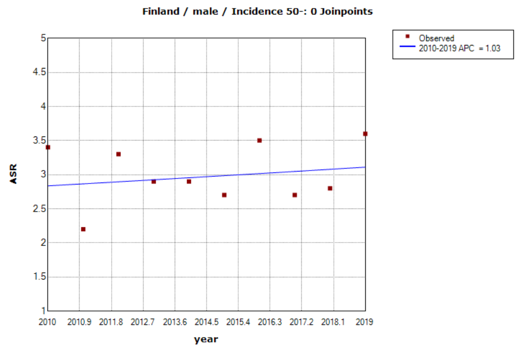

* Indicates that the Annual Percent Change (APC) is significantly different from zero at the alpha = 0.05 level.
Final Selected Model: 0 Joinpoints.

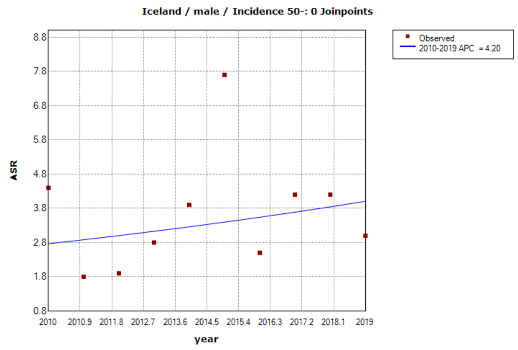

* Indicates that the Annual Percent Change (APC) is significantly different from zero at the alpha = 0.05 level.
Final Selected Model: 0 Joinpoints.

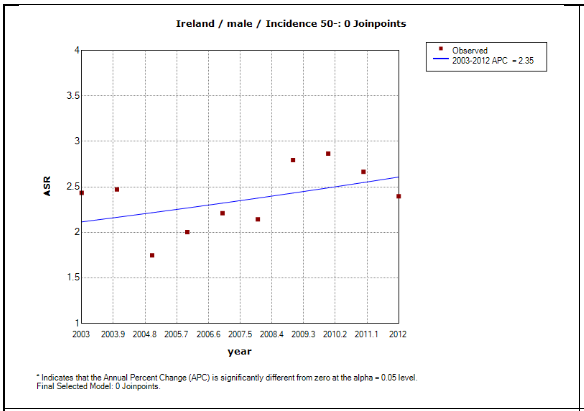

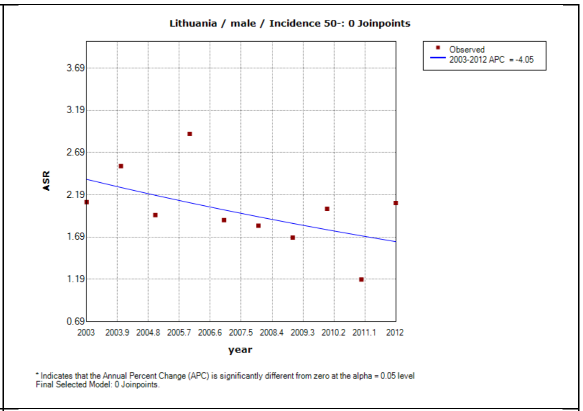

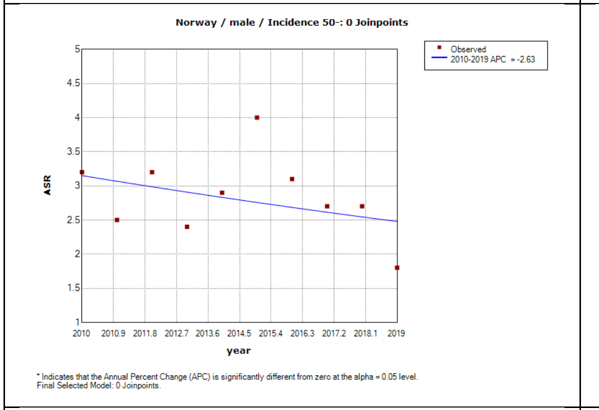

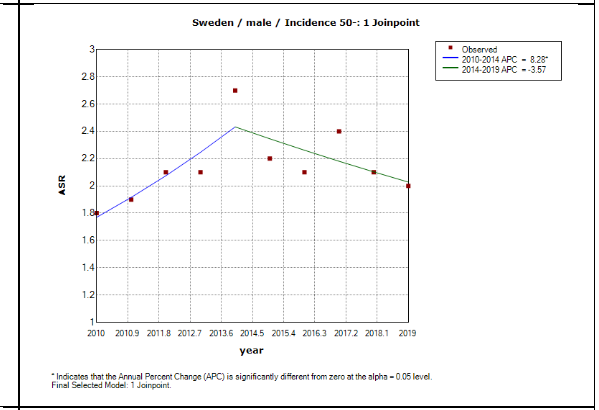

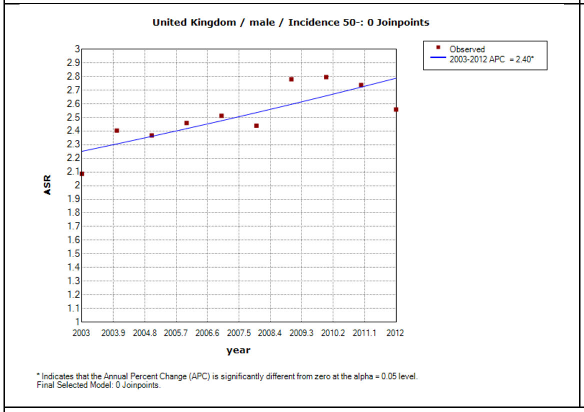

Western Europe

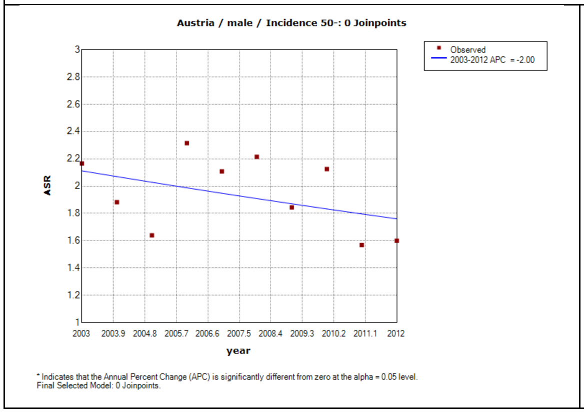

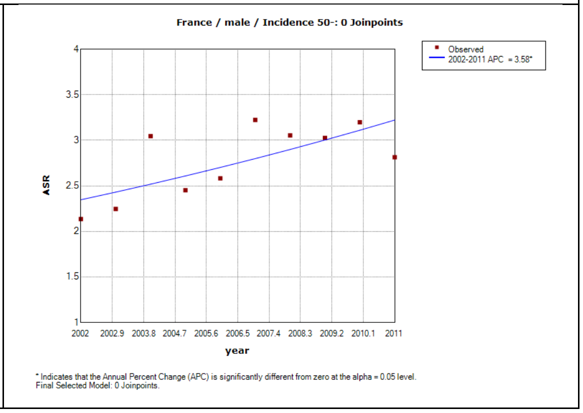

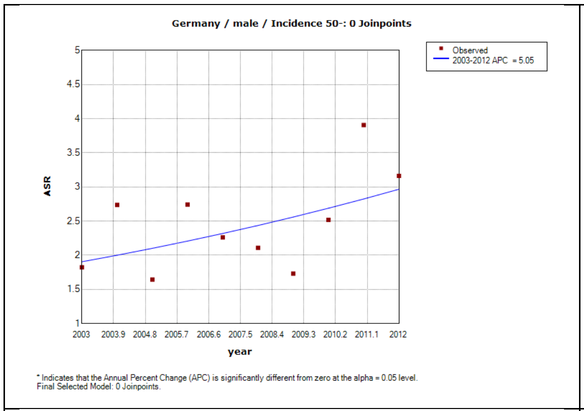

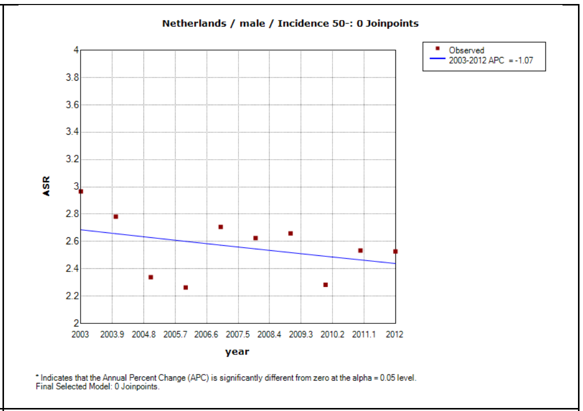

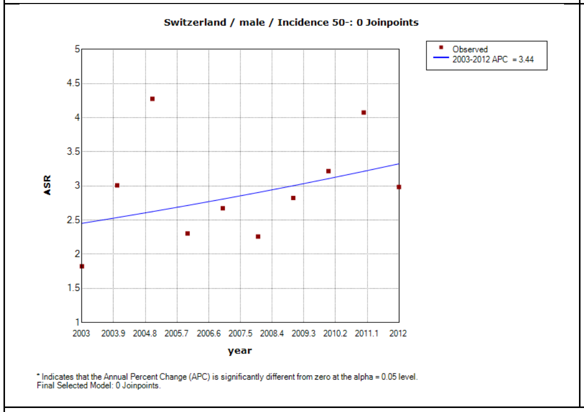

Southern Europe

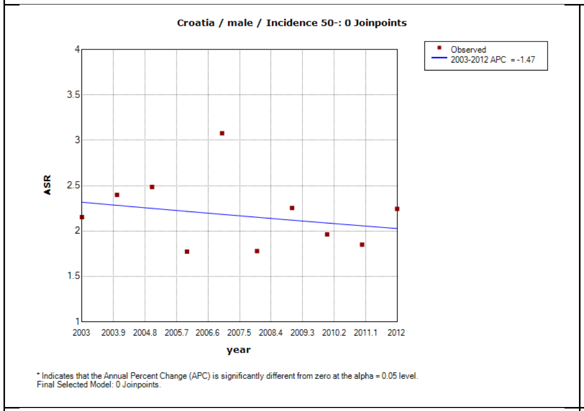

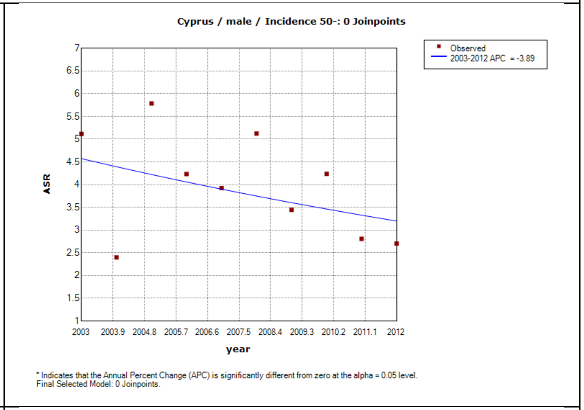

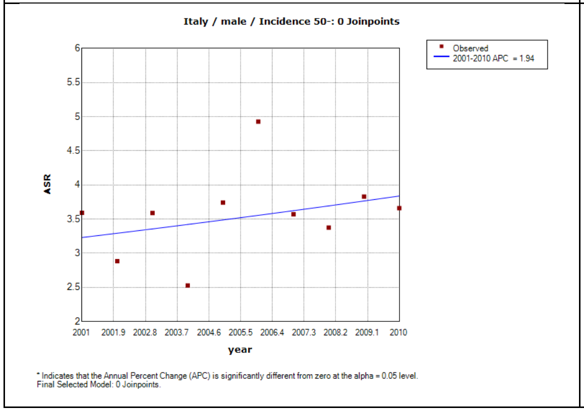

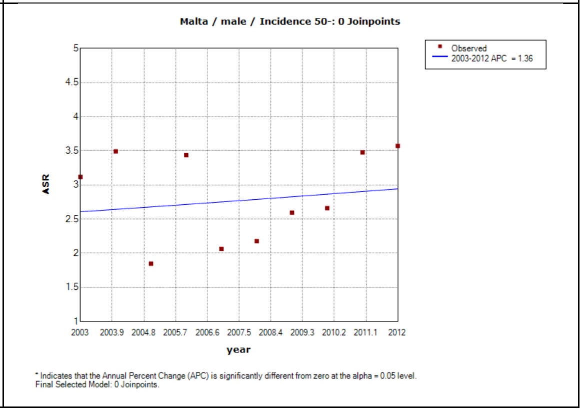

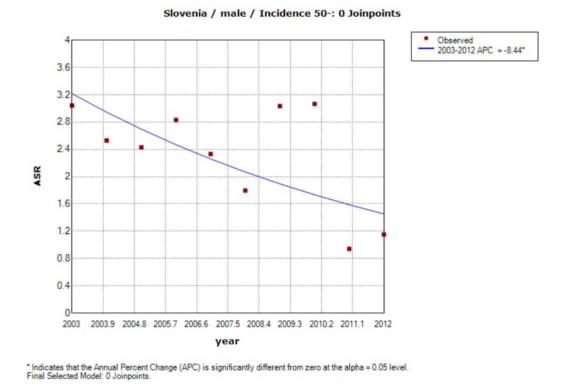

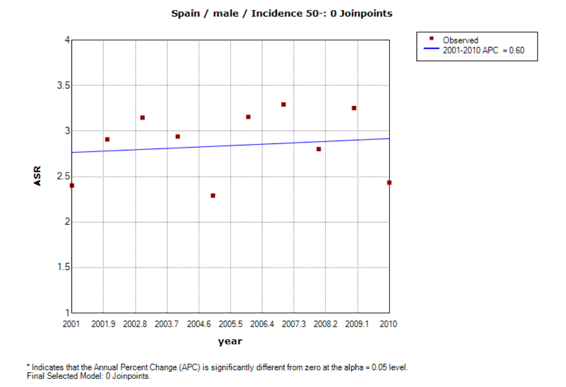

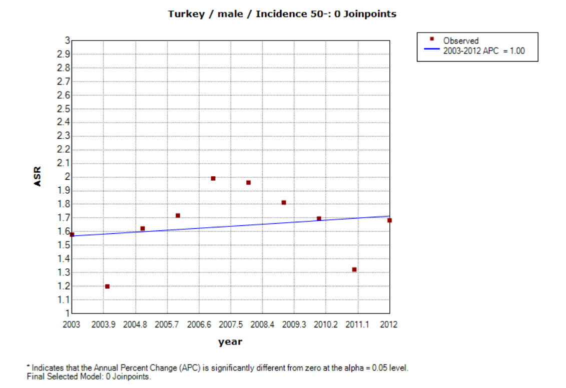

Eastern Europe

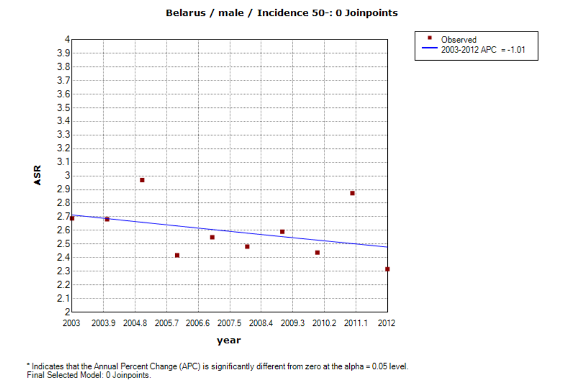

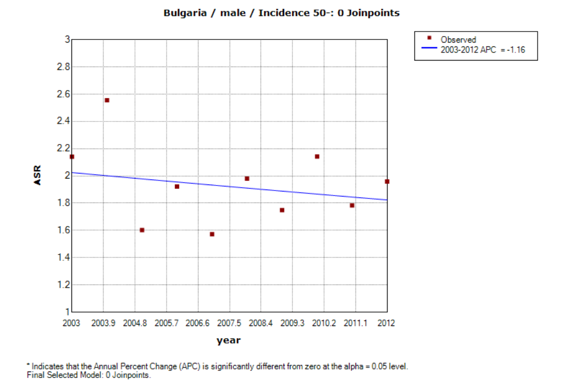

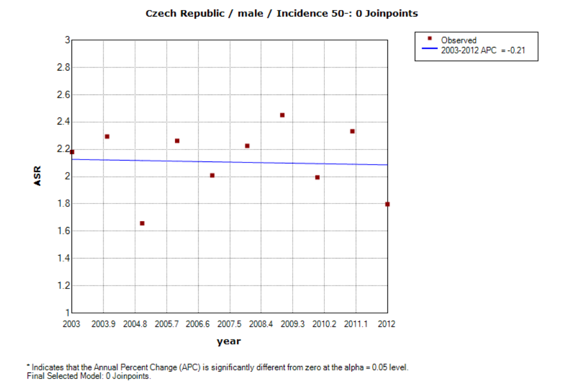

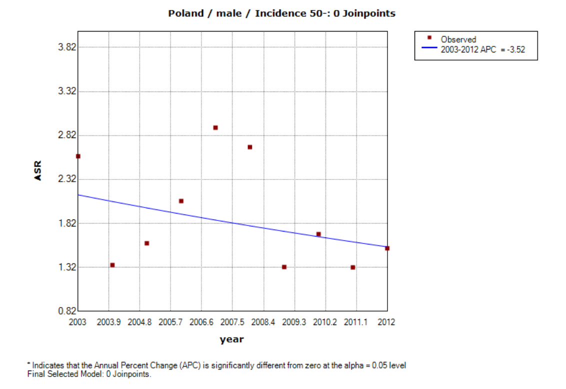

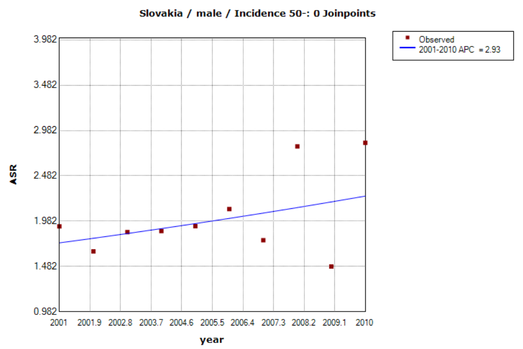

* Indicates that the Annual Percent Change (APC) is significantly different from zero at the alpha = 0.05 level
Final Selected Model: 0 Joinpoints.

Africa

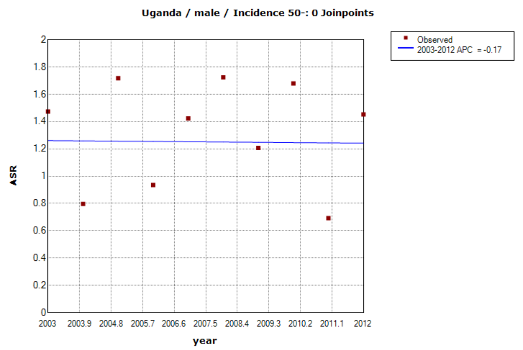

* Indicates that the Annual Percent Change (APC) is significantly different from zero at the alpha = 0.05 level.
Final Selected Model: 0 Joinpoints.

f.) Incidence female below 50 years old

Asia

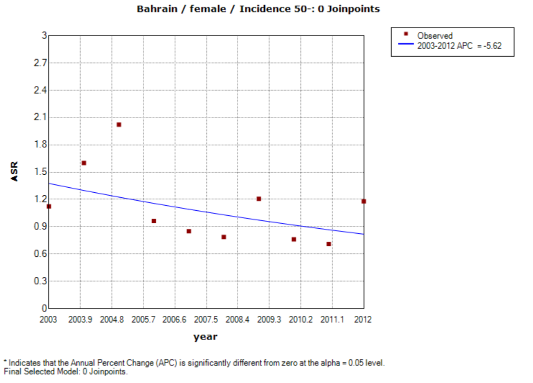

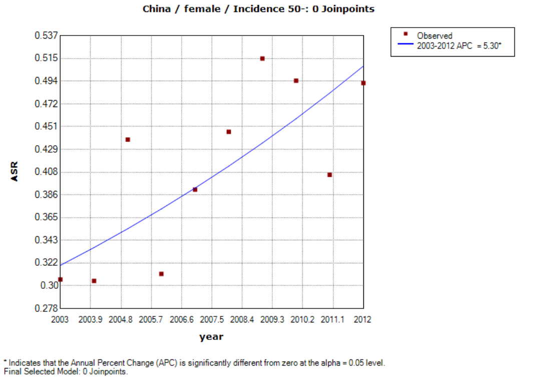

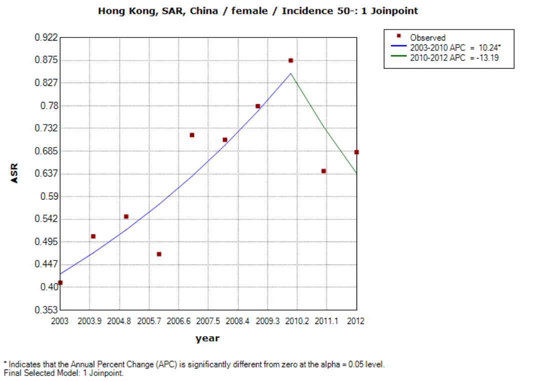

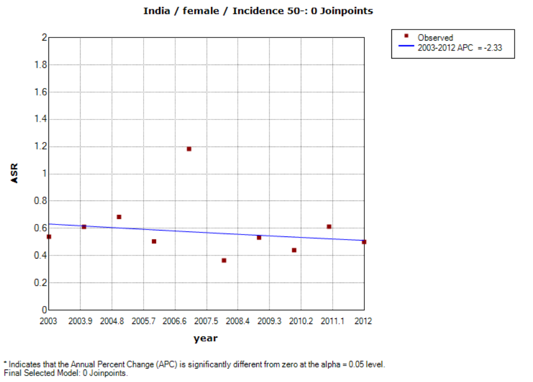

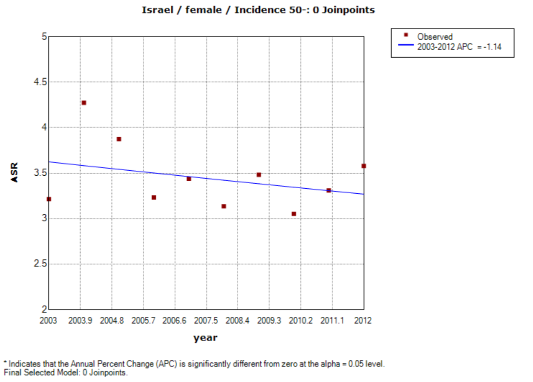

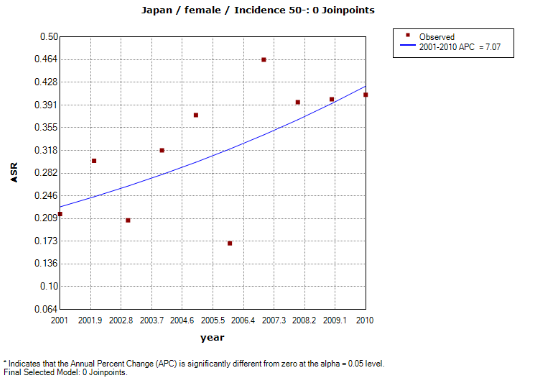

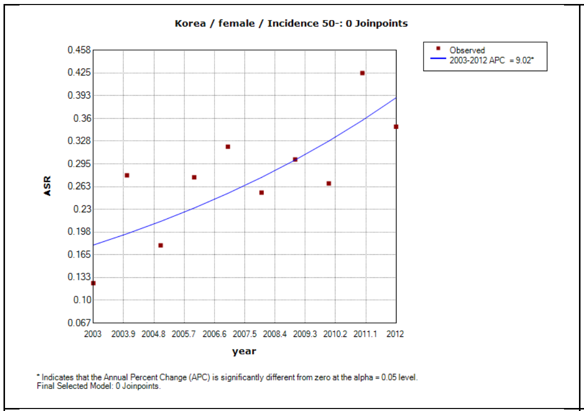
Korea / female / Incidence 50-: 0 Joinpoints
Observed
2003-2012 APC = 9.02*
ASR
year
* Indicates that the Annual Percent Change (APC) is significantly different from zero at the alpha = 0.05 level.
Final Selected Model: 0 Joinpoints.

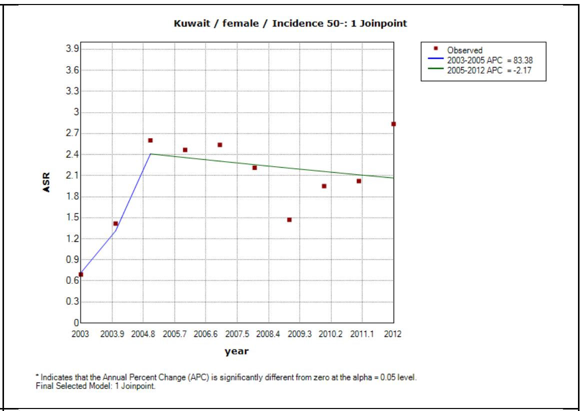
Kuwait / female / Incidence 50-: 1 Joinpoint
Observed
2003-2005 APC = 83.38
2005-2012 APC = -2.17
ASR
year
* Indicates that the Annual Percent Change (APC) is significantly different from zero at the alpha = 0.05 level.
Final Selected Model: 1 Joinpoint.

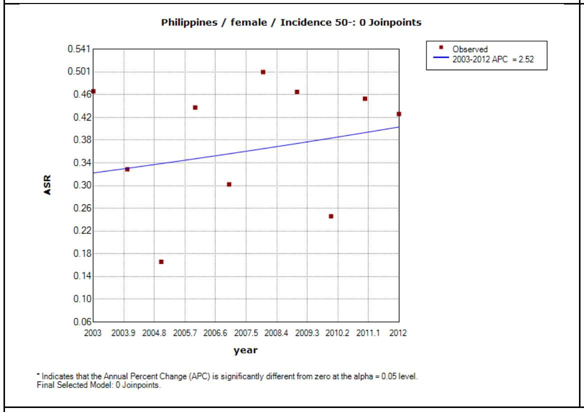
Philippines / female / Incidence 50-: 0 Joinpoints
Observed
2003-2012 APC = 2.52
ASR
year
* Indicates that the Annual Percent Change (APC) is significantly different from zero at the alpha = 0.05 level.
Final Selected Model: 0 Joinpoints.

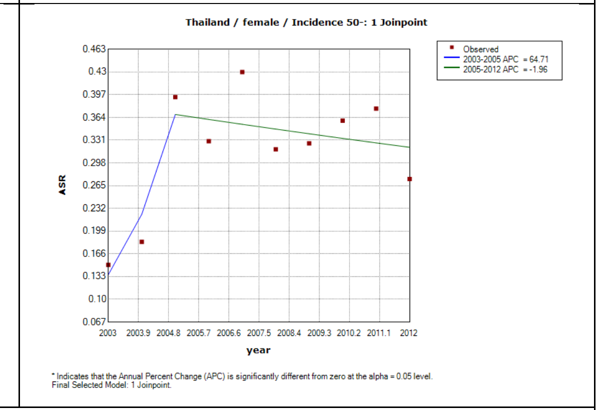
Thailand / female / Incidence 50-: 1 Joinpoint
Observed
2003-2005 APC = 64.71
2005-2012 APC = -1.96
ASR
year
* Indicates that the Annual Percent Change (APC) is significantly different from zero at the alpha = 0.05 level.
Final Selected Model: 1 Joinpoint.

Oceania

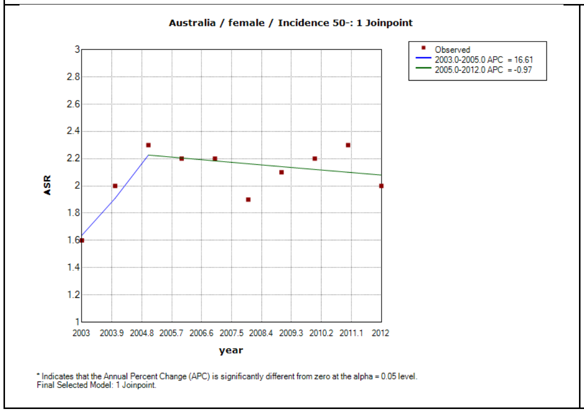
Australia / female / Incidence 50-: 1 Joinpoint
Observed
2003.0-2005.0 APC = 16.61
2005.0-2012.0 APC = -0.97
ASR
year
* Indicates that the Annual Percent Change (APC) is significantly different from zero at the alpha = 0.05 level.
Final Selected Model: 1 Joinpoint.

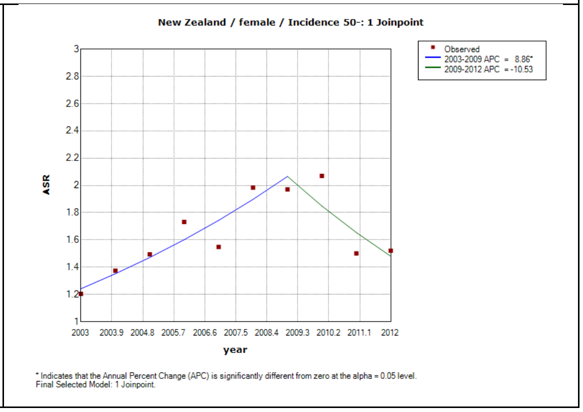
New Zealand / female / Incidence 50-: 1 Joinpoint
Observed
2003-2009 APC = 8.86*
2009-2012 APC = -10.53
ASR
year
* Indicates that the Annual Percent Change (APC) is significantly different from zero at the alpha = 0.05 level.
Final Selected Model: 1 Joinpoint.

North America

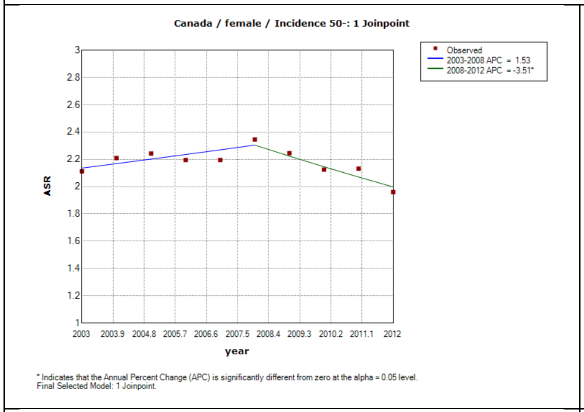

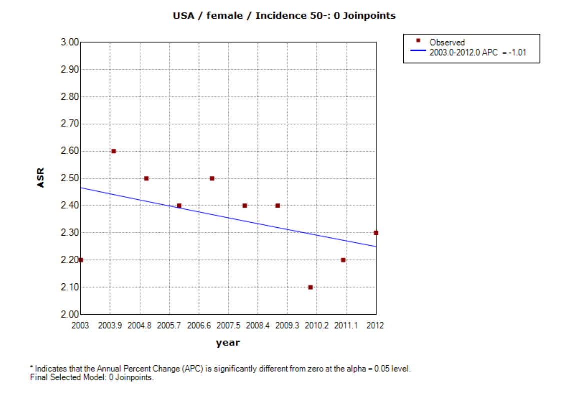

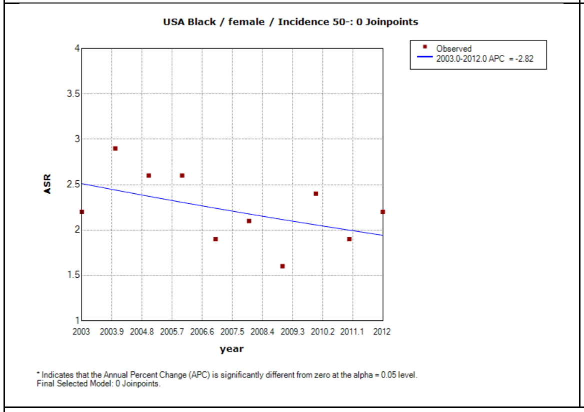

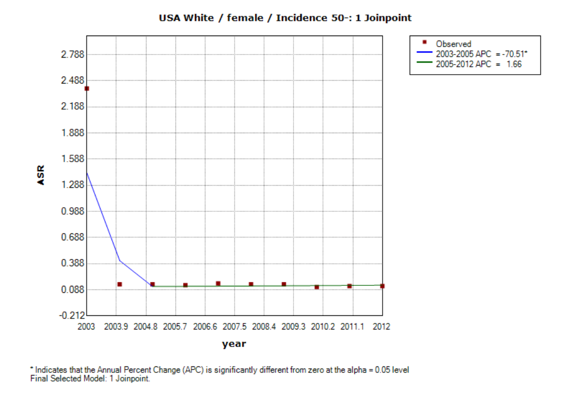

South America

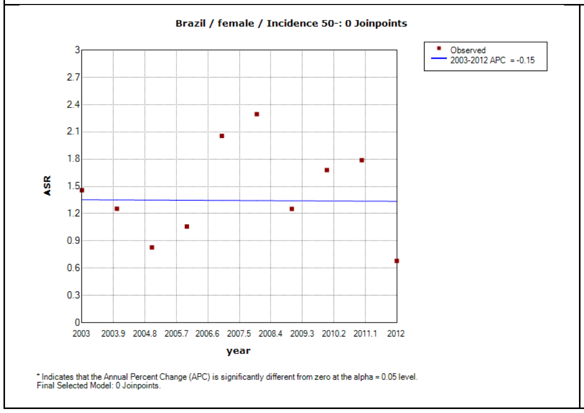

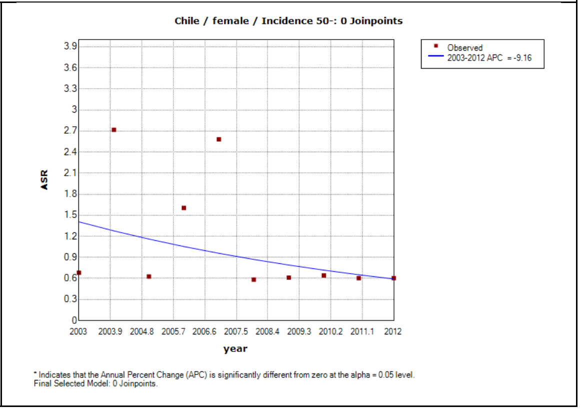

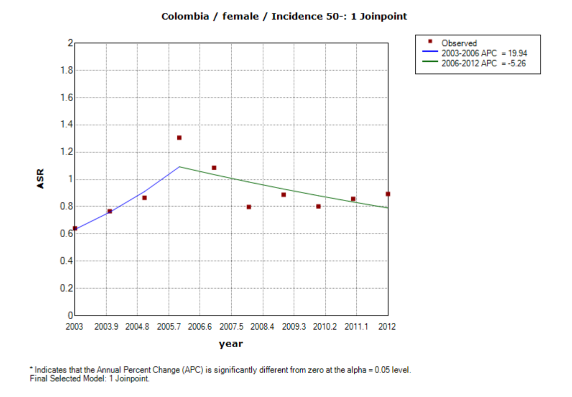

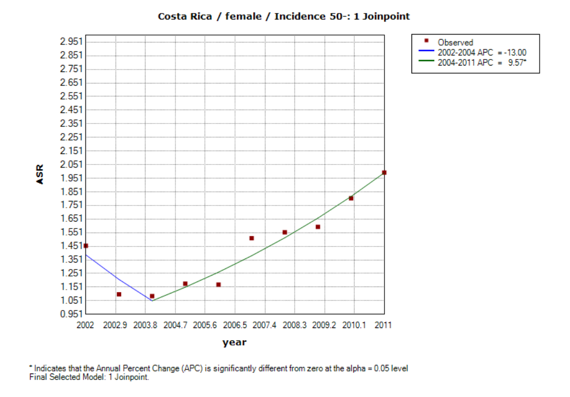

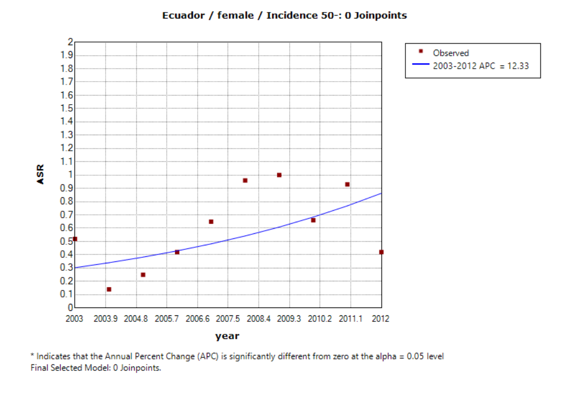

Northern Europe

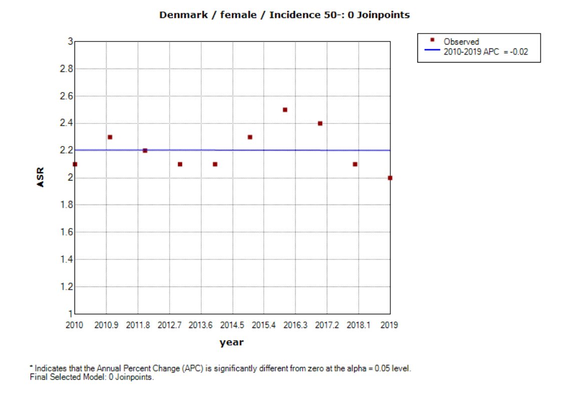

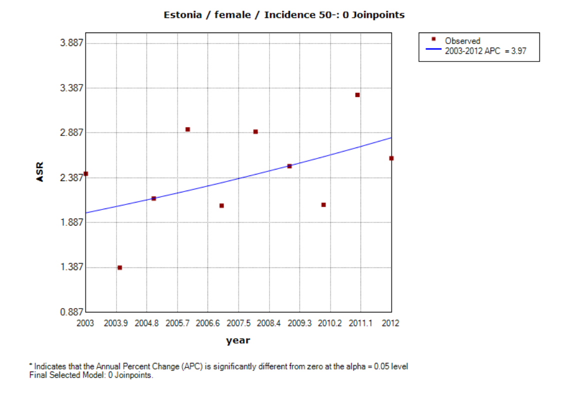

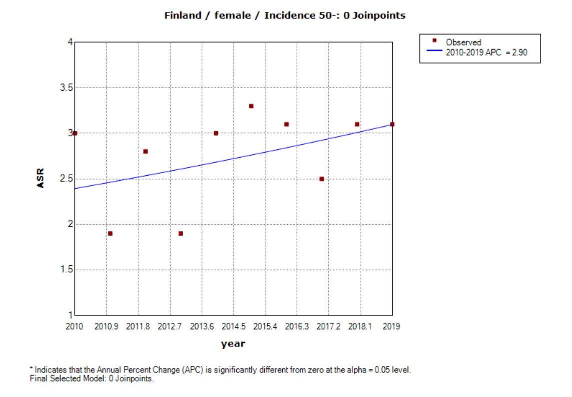

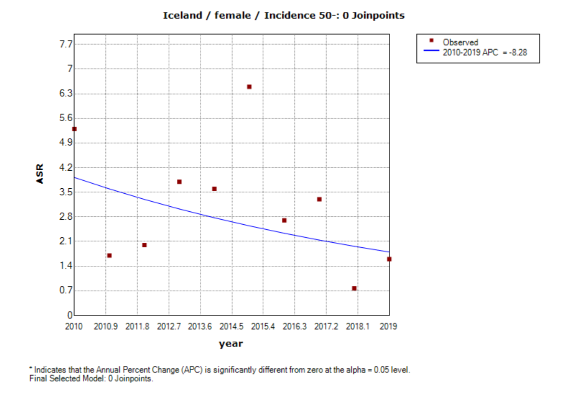

Ireland / female / Incidence 50-: 0 Joinpoints

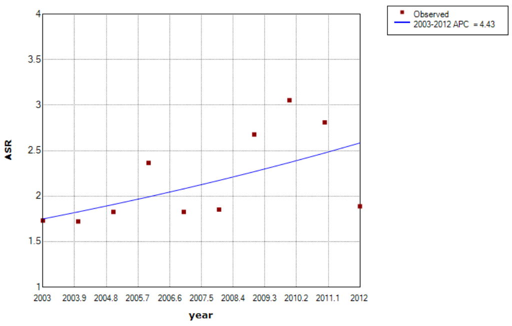

* Indicates that the Annual Percent Change (APC) is significantly different from zero at the alpha = 0.05 level.
Final Selected Model: 0 Joinpoints.

Lithuania / female / Incidence 50-: 0 Joinpoints

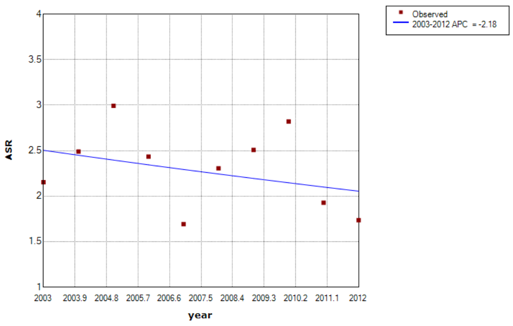

* Indicates that the Annual Percent Change (APC) is significantly different from zero at the alpha = 0.05 level.
Final Selected Model: 0 Joinpoints.

Norway / female / Incidence 50-: 0 Joinpoints

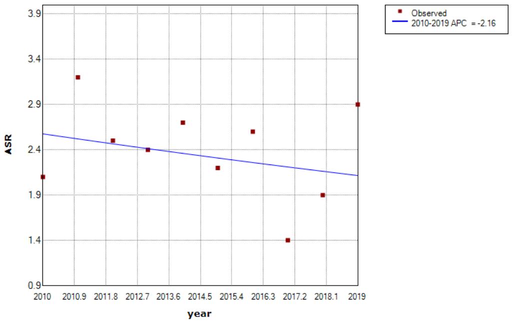

* Indicates that the Annual Percent Change (APC) is significantly different from zero at the alpha = 0.05 level.
Final Selected Model: 0 Joinpoints.

Sweden / female / Incidence 50-: 0 Joinpoints

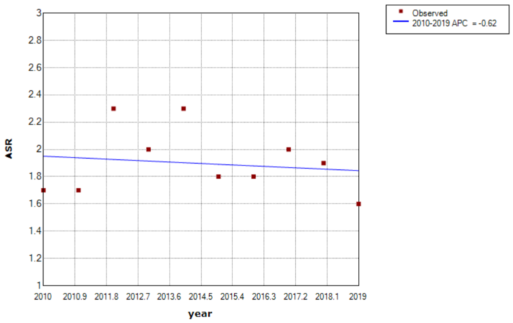

* Indicates that the Annual Percent Change (APC) is significantly different from zero at the alpha = 0.05 level.
Final Selected Model: 0 Joinpoints.

United Kingdom / female / Incidence 50-: 0 Joinpoints

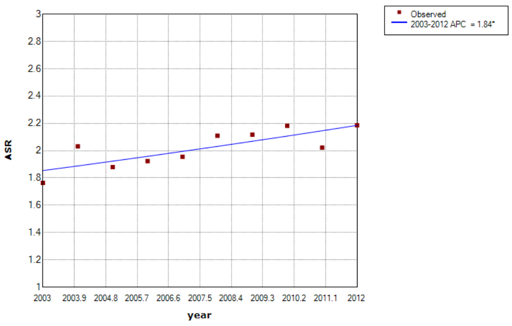

* Indicates that the Annual Percent Change (APC) is significantly different from zero at the alpha = 0.05 level.
Final Selected Model: 0 Joinpoints.

Western Europe

Austria / female / Incidence 50-: 0 Joinpoints

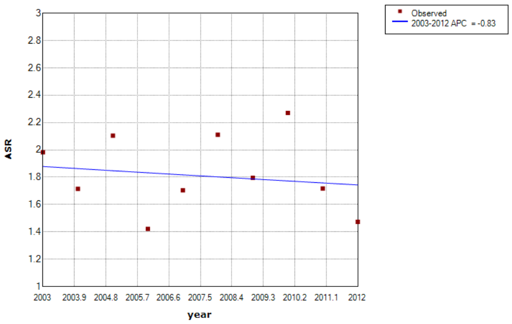

* Indicates that the Annual Percent Change (APC) is significantly different from zero at the alpha = 0.05 level.
Final Selected Model: 0 Joinpoints.

France / female / Incidence 50-: 0 Joinpoints

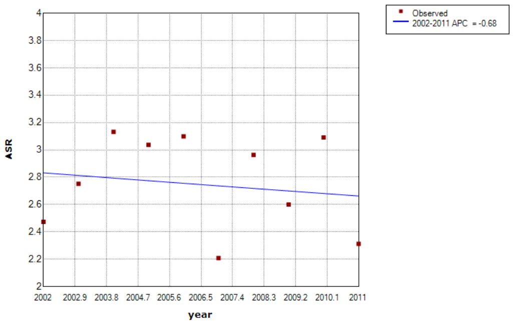

* Indicates that the Annual Percent Change (APC) is significantly different from zero at the alpha = 0.05 level.
Final Selected Model: 0 Joinpoints.

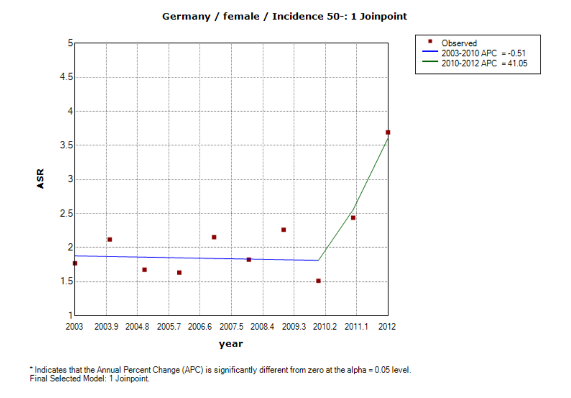

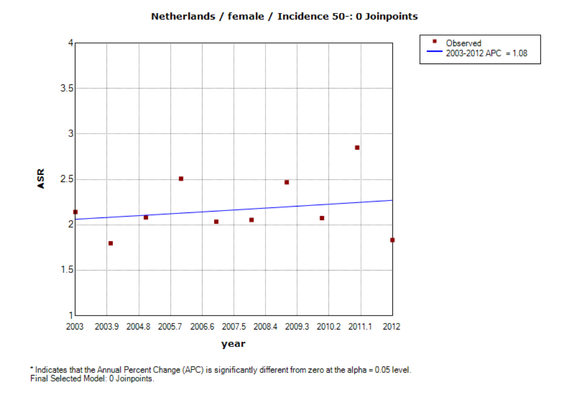

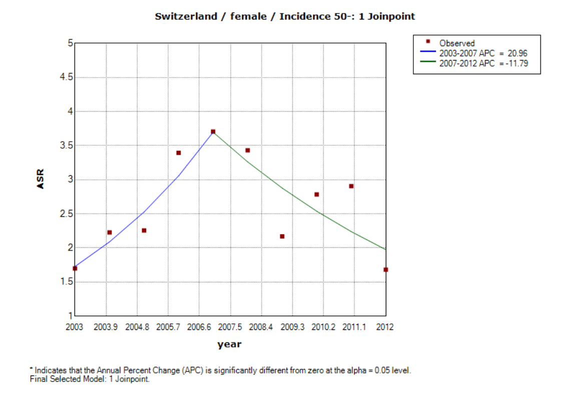

Southern Europe

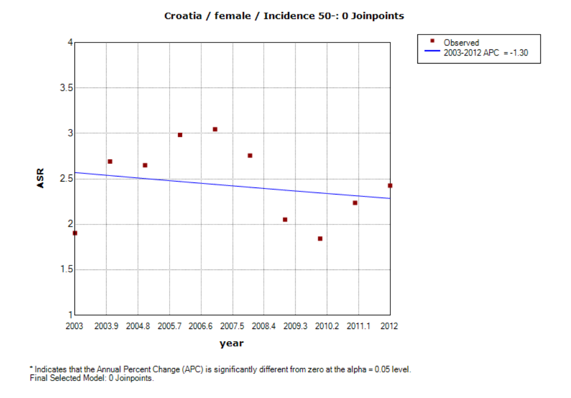

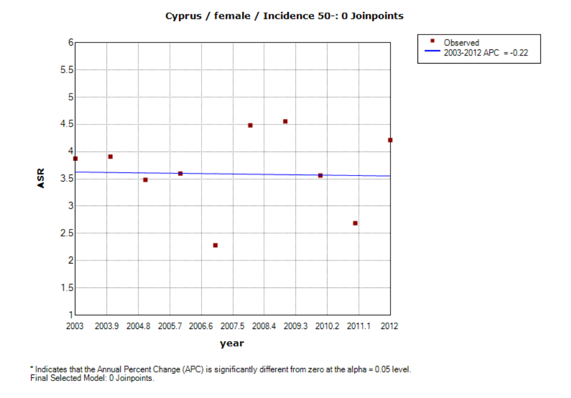

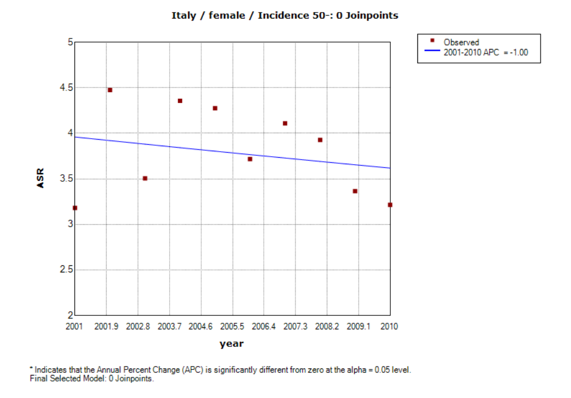

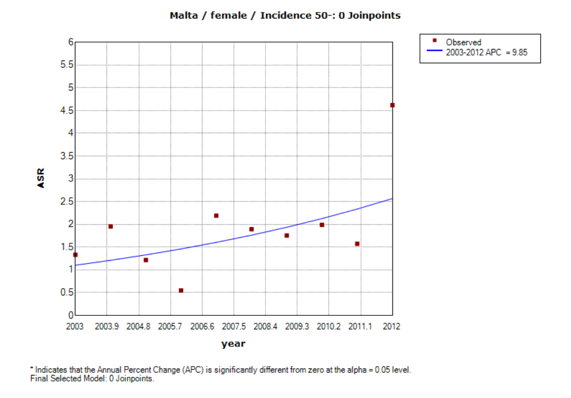

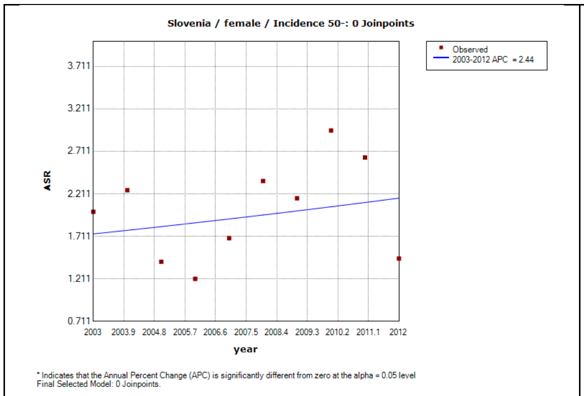

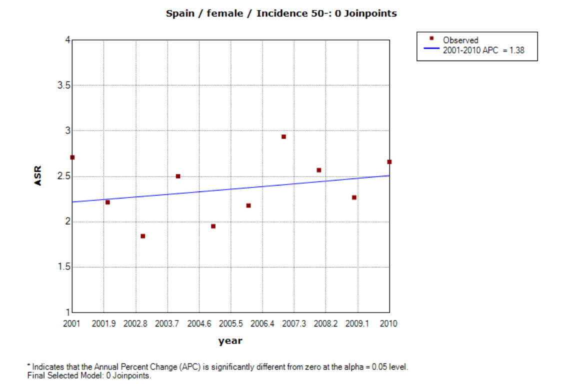

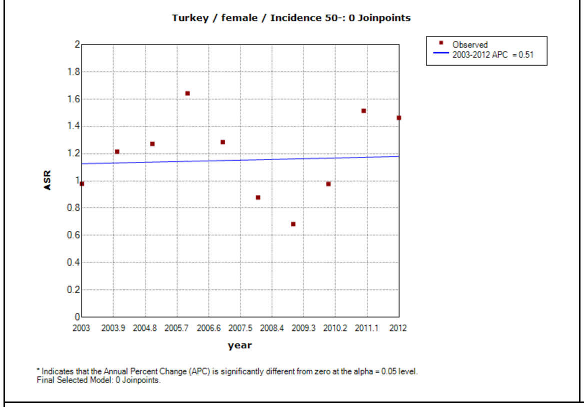

Eastern Europe

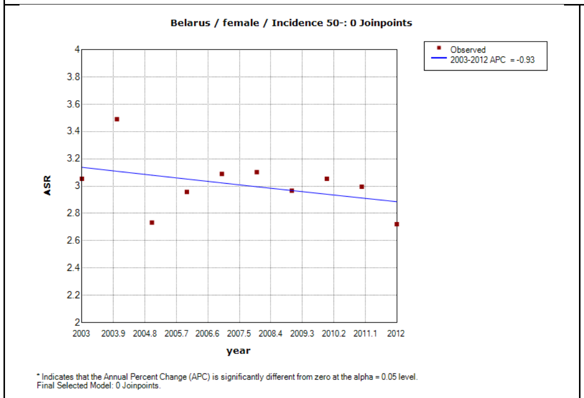

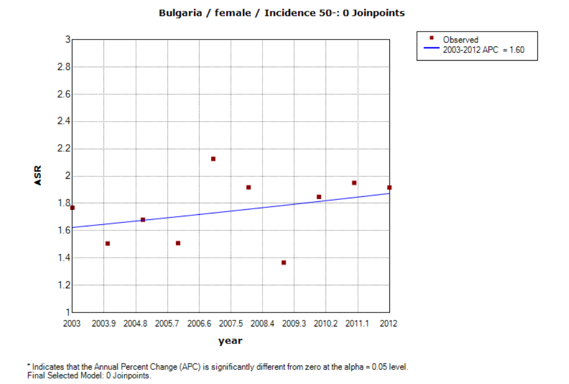

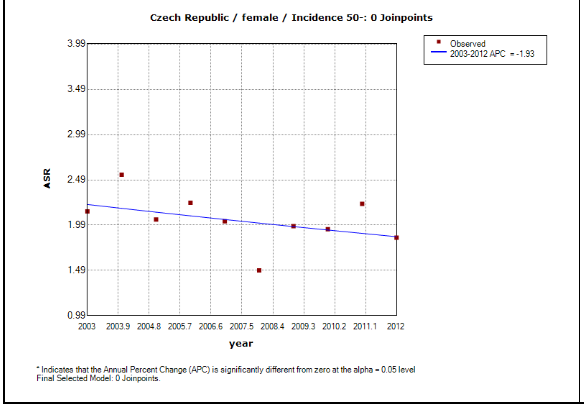

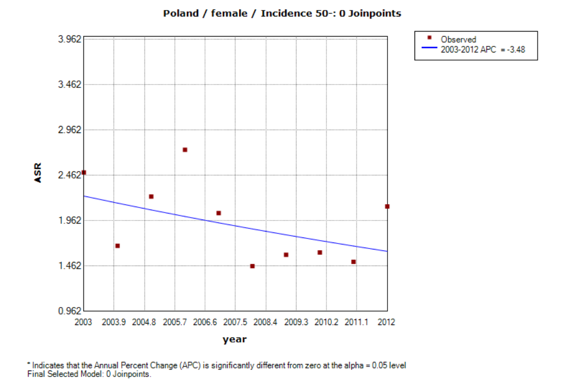

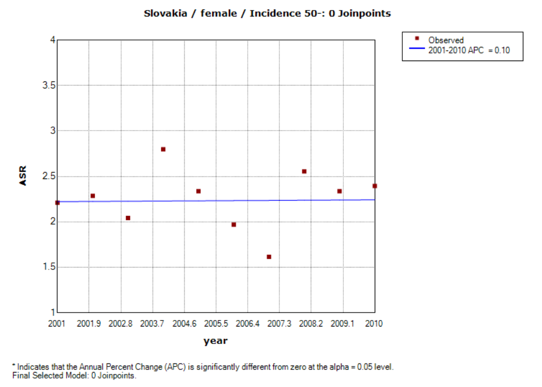
Slovakia / female / Incidence 50-: 0 Joinpoints
Observed
2001-2010 APC = 0.10
ASR
year
* Indicates that the Annual Percent Change (APC) is significantly different from zero at the alpha = 0.05 level.
Final Selected Model: 0 Joinpoints.

Africa

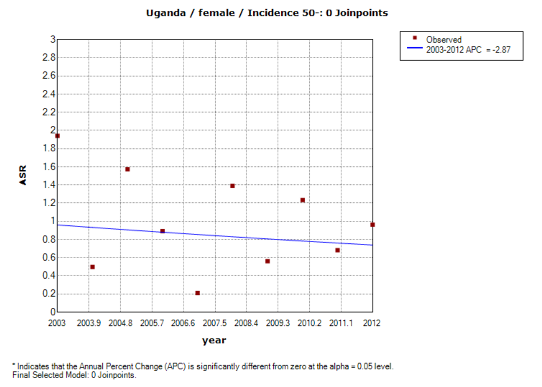
Uganda / female / Incidence 50-: 0 Joinpoints
Observed
2003-2012 APC = -2.87
ASR
year
* Indicates that the Annual Percent Change (APC) is significantly different from zero at the alpha = 0.05 level.
Final Selected Model: 0 Joinpoints.

g.) Incidence male above 50 years old

Asia

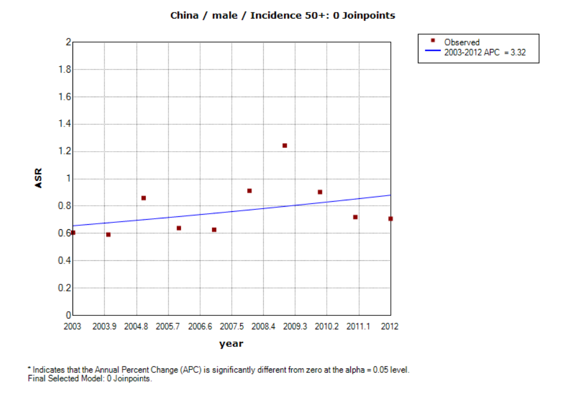

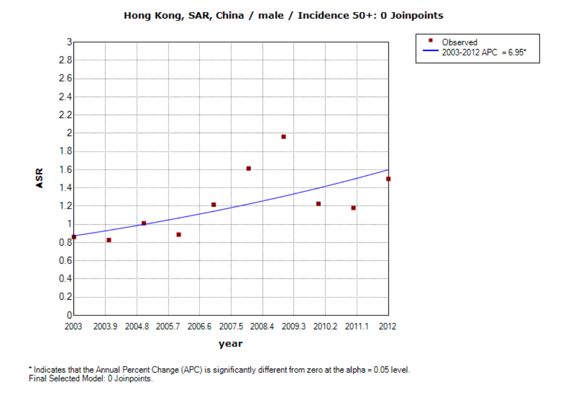

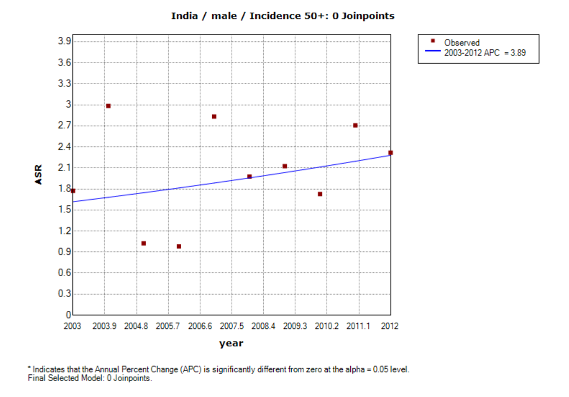

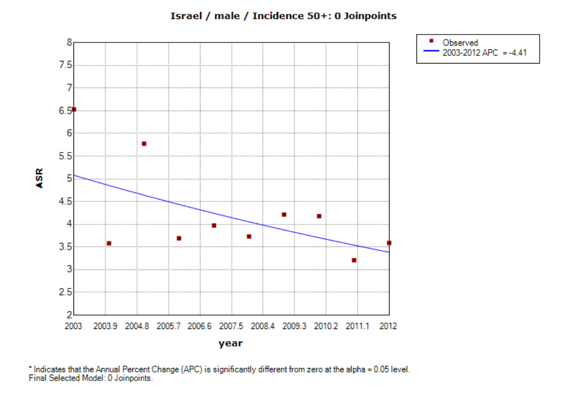

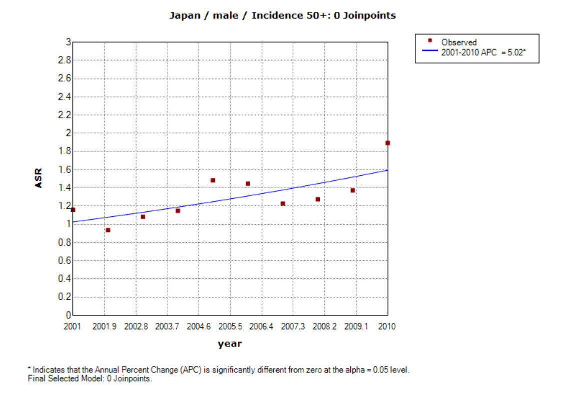

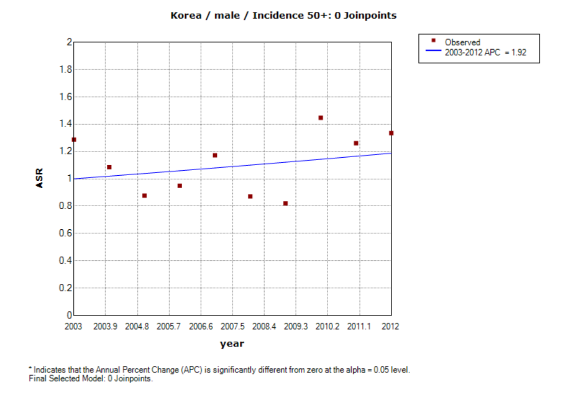

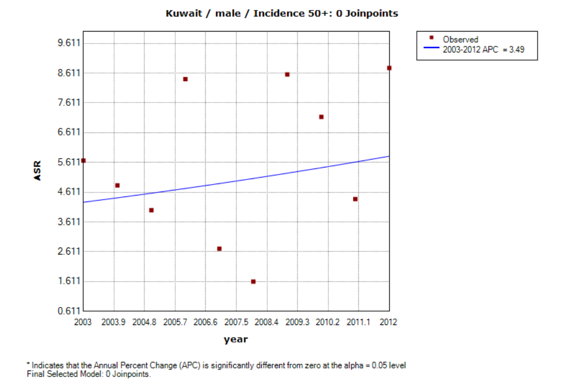
Kuwait / male / Incidence 50+: 0 Joinpoints
Observed
2003-2012 APC = 3.49
ASR
9.611
8.611
7.611
6.611
5.611
4.611
3.611
2.611
1.611
0.611
2003 2003.9 2004.8 2005.7 2006.6 2007.5 2008.4 2009.3 2010.2 2011.1 2012
year
* Indicates that the Annual Percent Change (APC) is significantly different from zero at the alpha = 0.05 level
Final Selected Model: 0 Joinpoints.

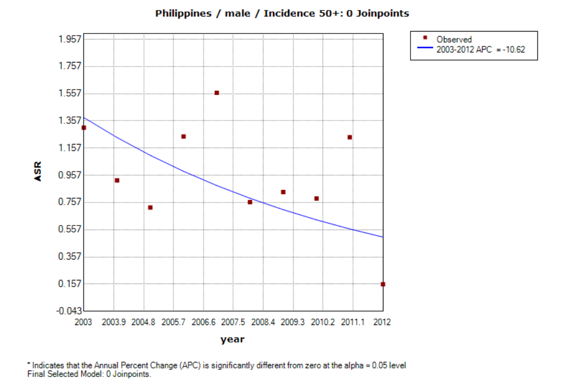
Philippines / male / Incidence 50+: 0 Joinpoints
Observed
2003-2012 APC = -10.62
ASR
1.957
1.757
1.557
1.357
1.157
0.957
0.757
0.557
0.357
0.157
-0.043
2003 2003.9 2004.8 2005.7 2006.6 2007.5 2008.4 2009.3 2010.2 2011.1 2012
year
* Indicates that the Annual Percent Change (APC) is significantly different from zero at the alpha = 0.05 level
Final Selected Model: 0 Joinpoints.

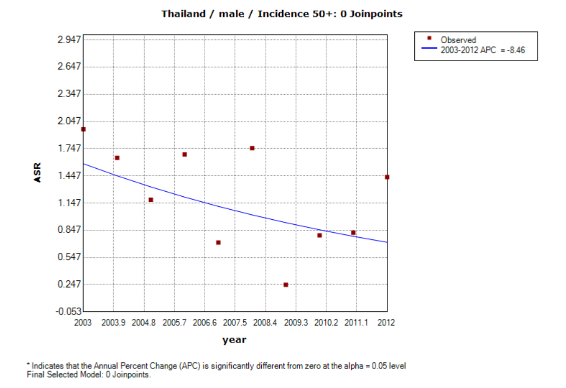
Thailand / male / Incidence 50+: 0 Joinpoints
Observed
2003-2012 APC = -8.46
ASR
2.947
2.647
2.347
2.047
1.747
1.447
1.147
0.847
0.547
0.247
-0.053
2003 2003.9 2004.8 2005.7 2006.6 2007.5 2008.4 2009.3 2010.2 2011.1 2012
year
* Indicates that the Annual Percent Change (APC) is significantly different from zero at the alpha = 0.05 level
Final Selected Model: 0 Joinpoints.

Oceania

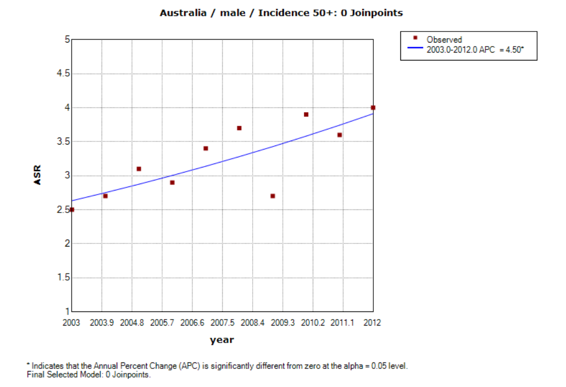
Australia / male / Incidence 50+: 0 Joinpoints
Observed
2003.0-2012.0 APC = 4.50*
ASR
5
4.5
4
3.5
3
2.5
2
1.5
1
2003 2003.9 2004.8 2005.7 2006.6 2007.5 2008.4 2009.3 2010.2 2011.1 2012
year
* Indicates that the Annual Percent Change (APC) is significantly different from zero at the alpha = 0.05 level.
Final Selected Model: 0 Joinpoints.

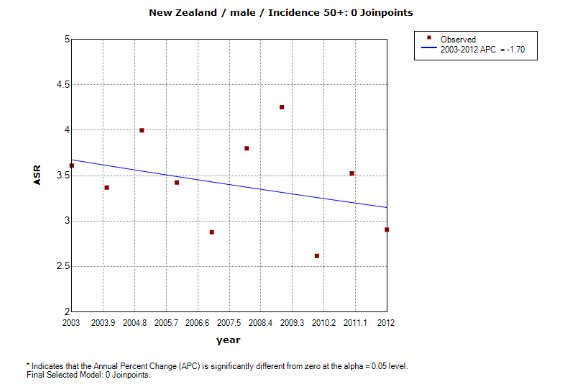
New Zealand / male / Incidence 50+: 0 Joinpoints
Observed
2003-2012 APC = -1.70
ASR
5
4.5
4
3.5
3
2.5
2
2003 2003.9 2004.8 2005.7 2006.6 2007.5 2008.4 2009.3 2010.2 2011.1 2012
year
* Indicates that the Annual Percent Change (APC) is significantly different from zero at the alpha = 0.05 level.
Final Selected Model: 0 Joinpoints.

North America

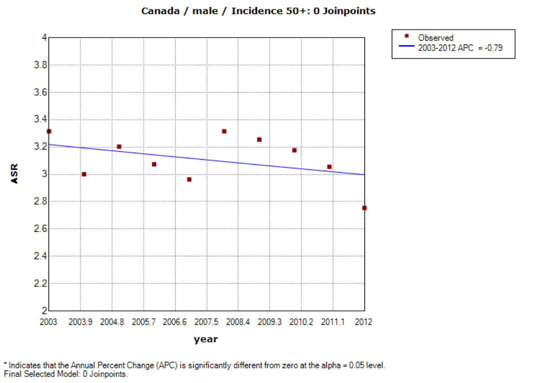

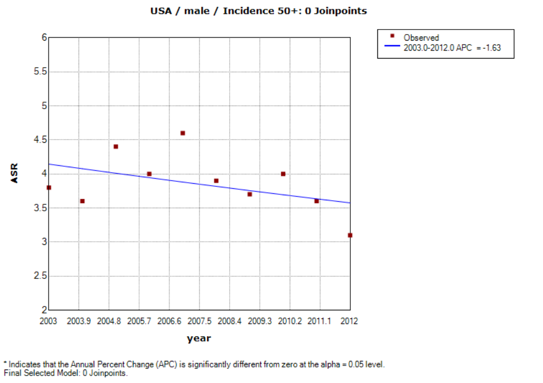

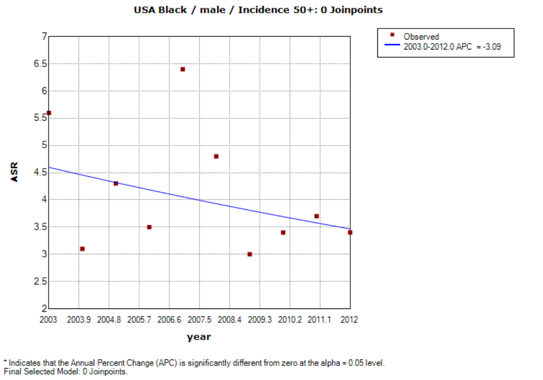

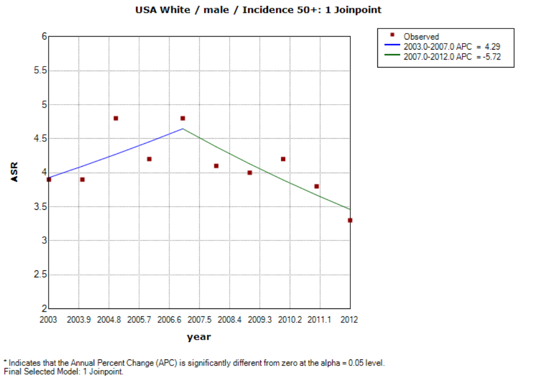

South America

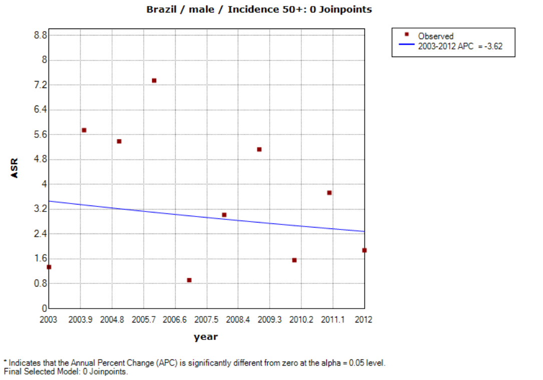

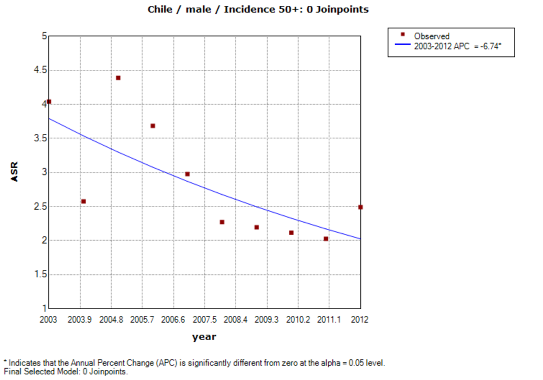

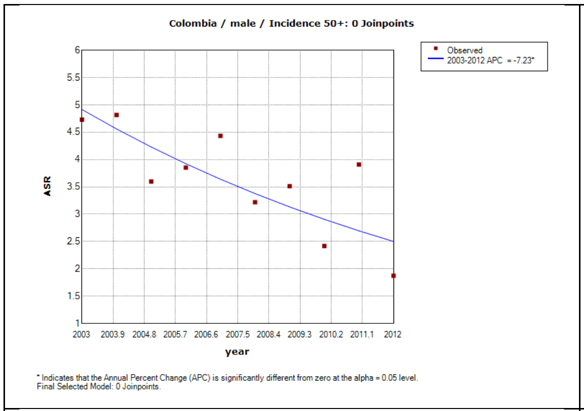

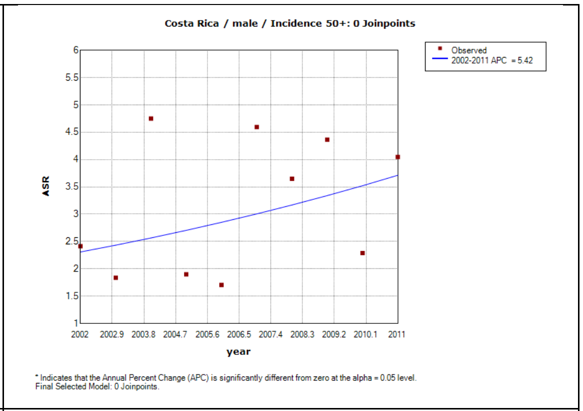

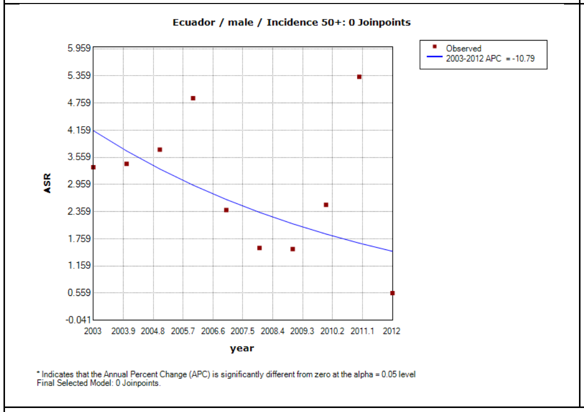

Northern Europe

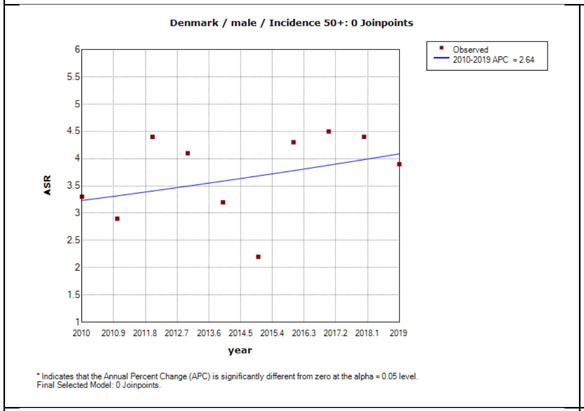

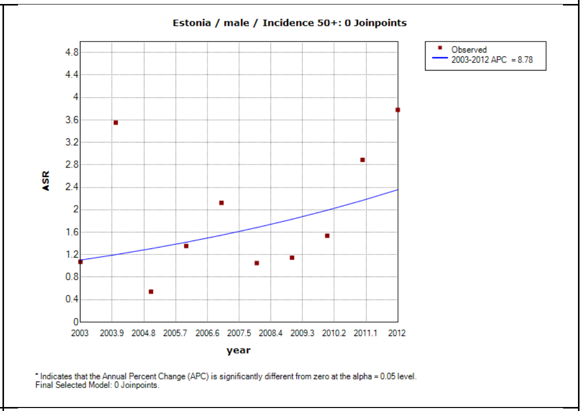

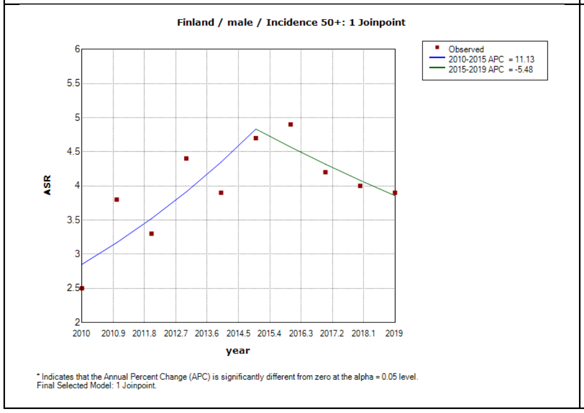

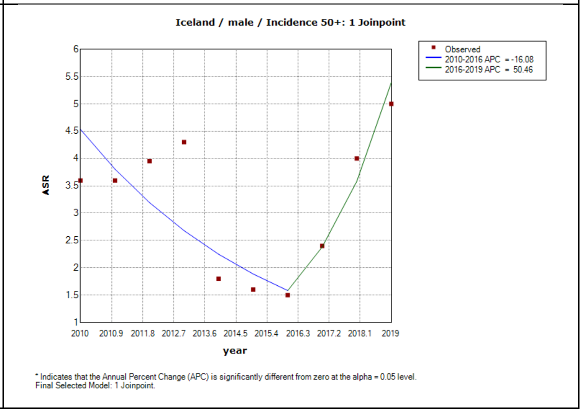

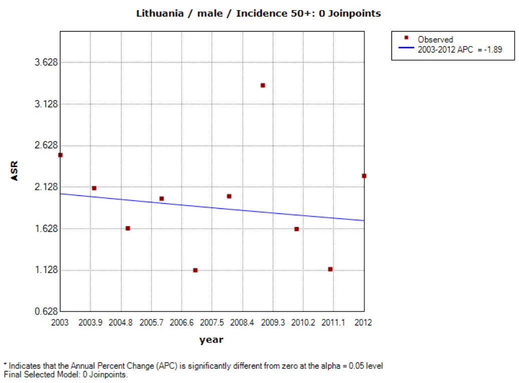

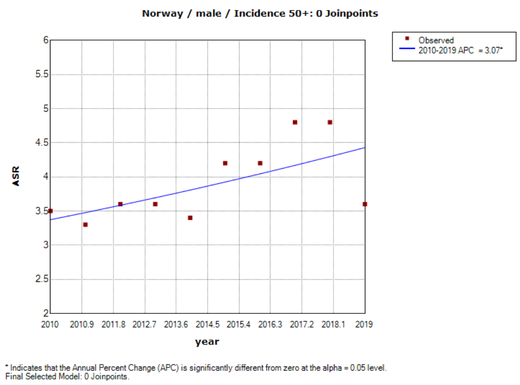

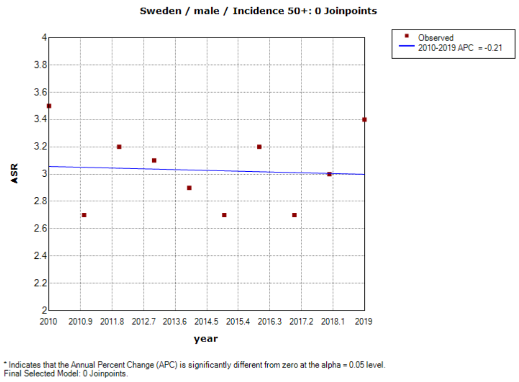

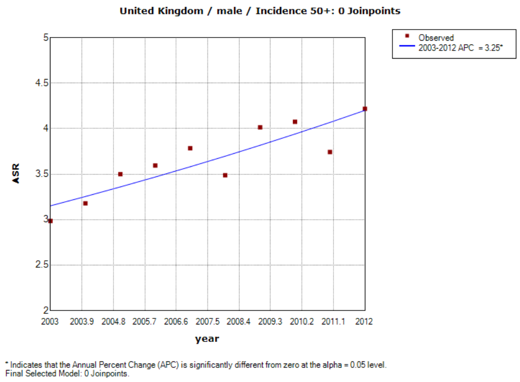

Western Europe

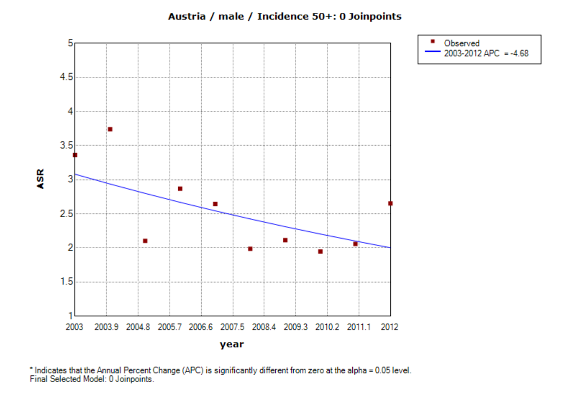

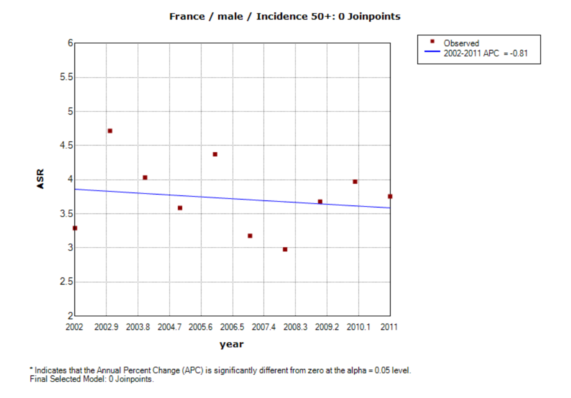

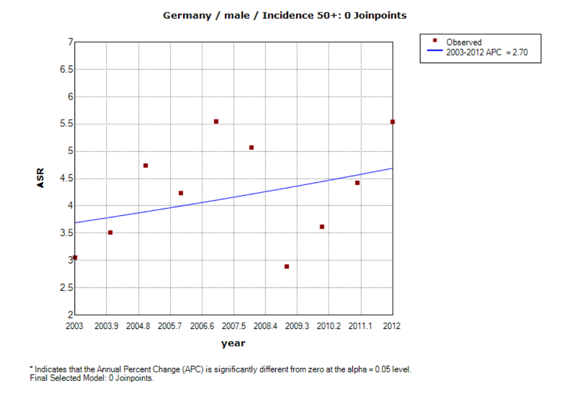

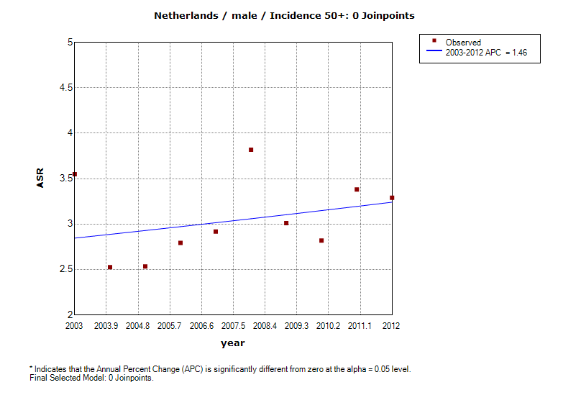

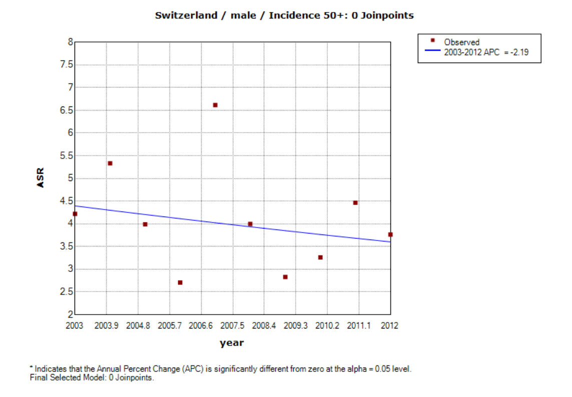

Southern Europe

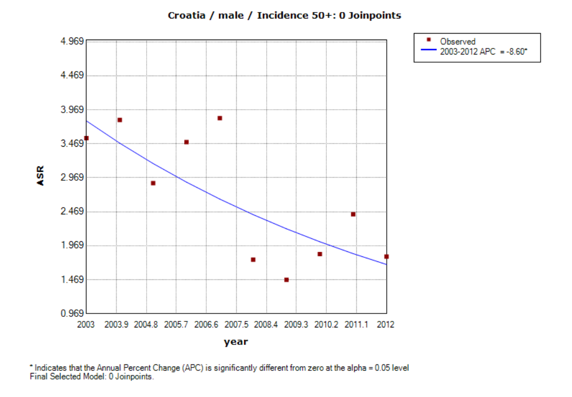

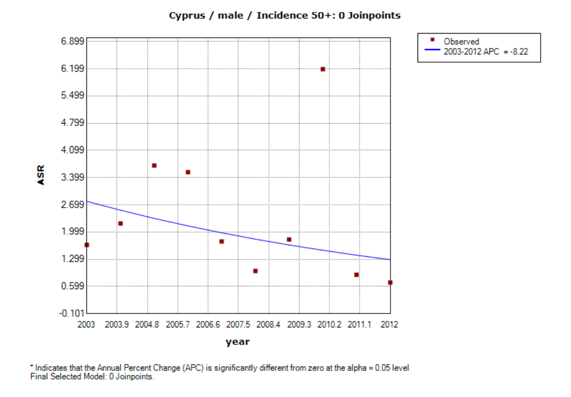

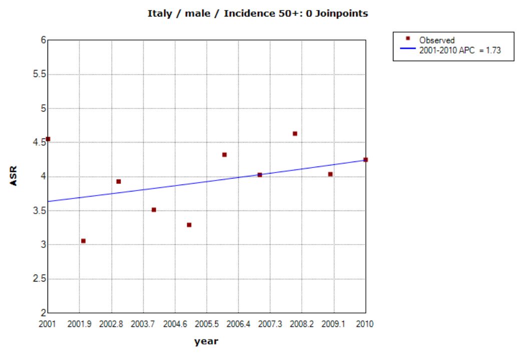

* Indicates that the Annual Percent Change (APC) is significantly different from zero at the alpha = 0.05 level.
Final Selected Model: 0 Joinpoints.

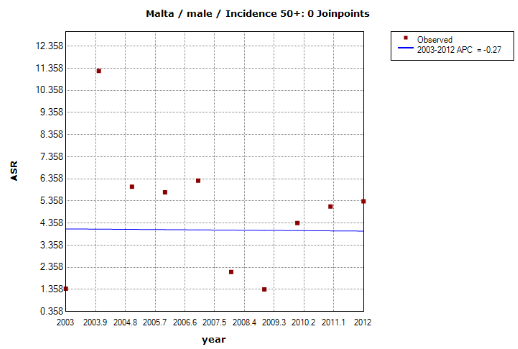

* Indicates that the Annual Percent Change (APC) is significantly different from zero at the alpha = 0.05 level
Final Selected Model: 0 Joinpoints.

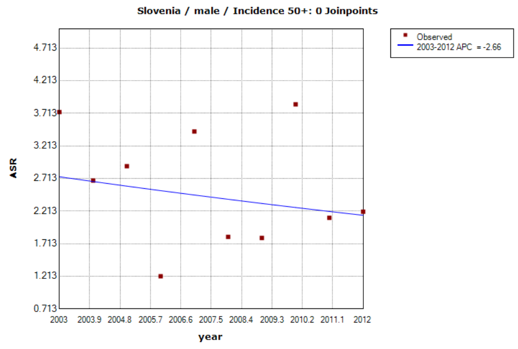

* Indicates that the Annual Percent Change (APC) is significantly different from zero at the alpha = 0.05 level
Final Selected Model: 0 Joinpoints.

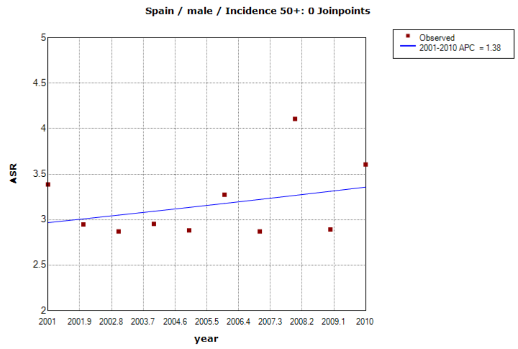

* Indicates that the Annual Percent Change (APC) is significantly different from zero at the alpha = 0.05 level.
Final Selected Model: 0 Joinpoints.

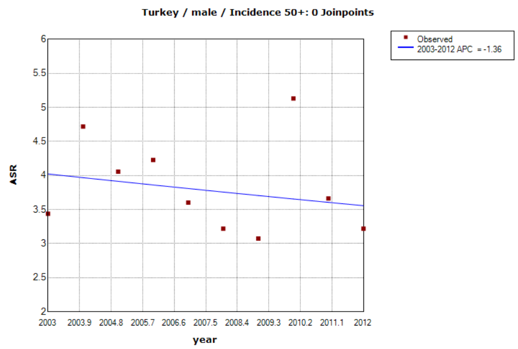

* Indicates that the Annual Percent Change (APC) is significantly different from zero at the alpha = 0.05 level.
Final Selected Model: 0 Joinpoints.

Eastern Europe

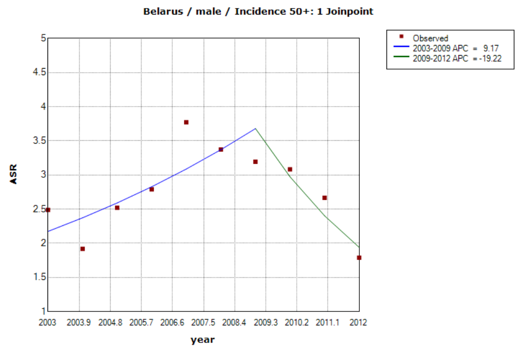

* Indicates that the Annual Percent Change (APC) is significantly different from zero at the alpha = 0.05 level.
Final Selected Model: 1 Joinpoint.

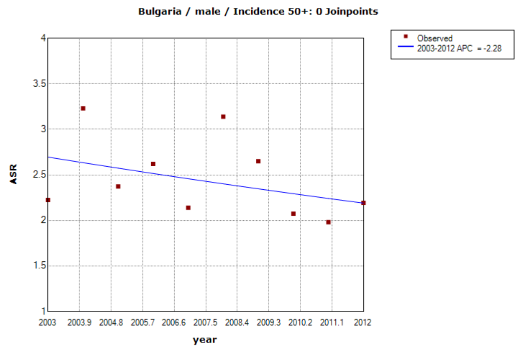

* Indicates that the Annual Percent Change (APC) is significantly different from zero at the alpha = 0.05 level.
Final Selected Model: 0 Joinpoints.

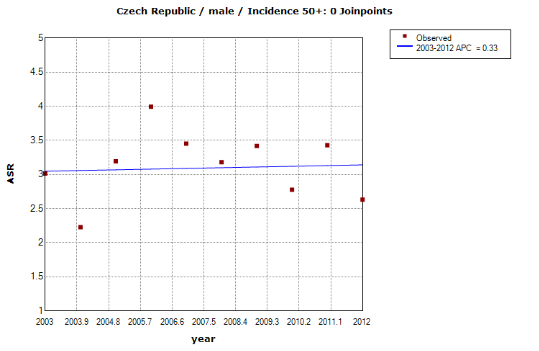

* Indicates that the Annual Percent Change (APC) is significantly different from zero at the alpha = 0.05 level.
Final Selected Model: 0 Joinpoints.

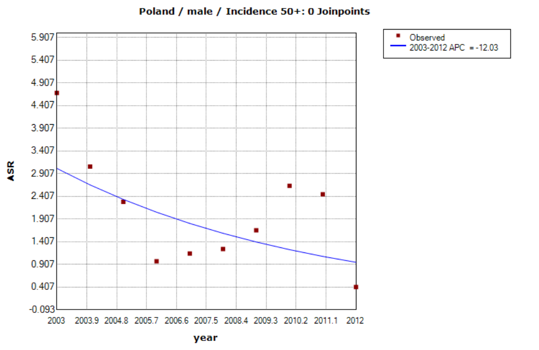

* Indicates that the Annual Percent Change (APC) is significantly different from zero at the alpha = 0.05 level
Final Selected Model: 0 Joinpoints.

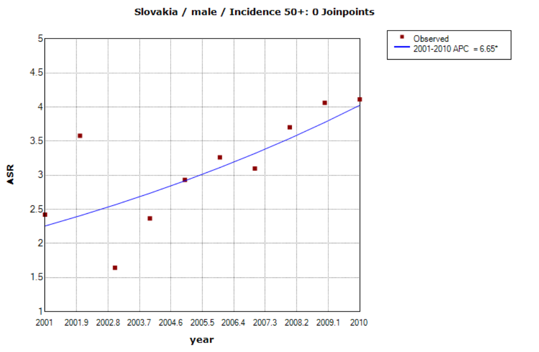

* Indicates that the Annual Percent Change (APC) is significantly different from zero at the alpha = 0.05 level.
Final Selected Model: 0 Joinpoints.

Africa

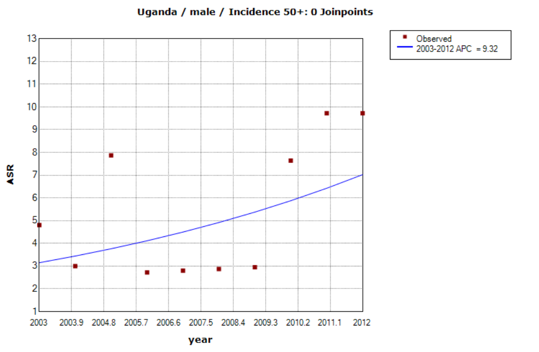

* Indicates that the Annual Percent Change (APC) is significantly different from zero at the alpha = 0.05 level.
Final Selected Model: 0 Joinpoints.

h.) Incidence female above 50 years old

Asia

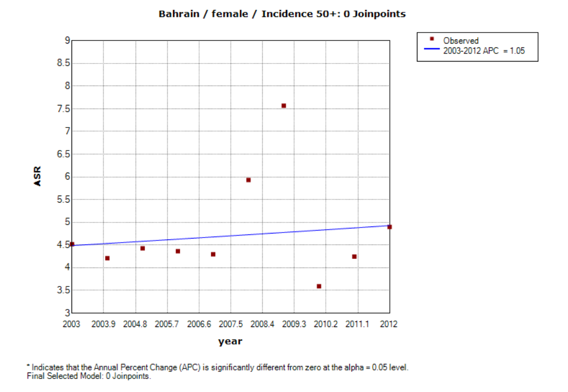

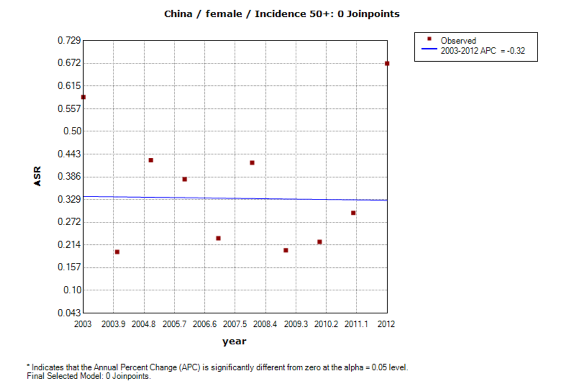

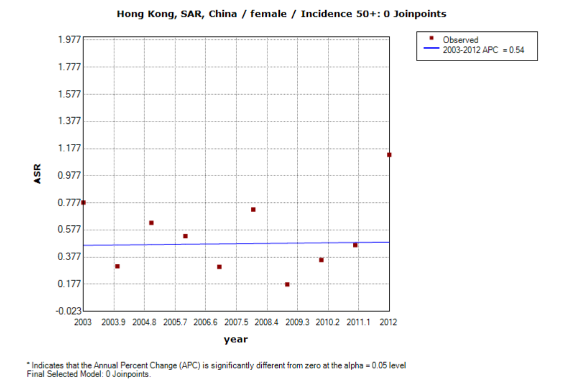

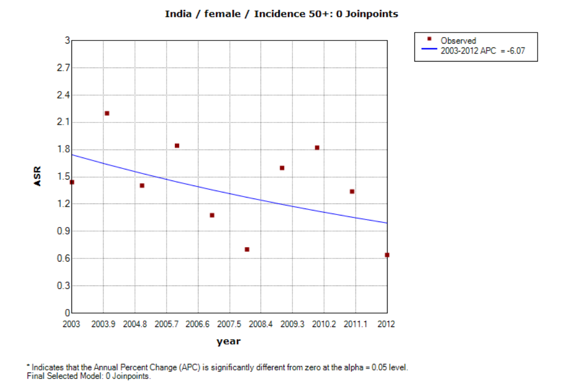

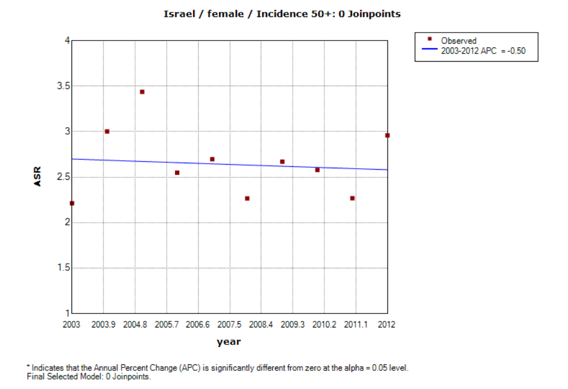

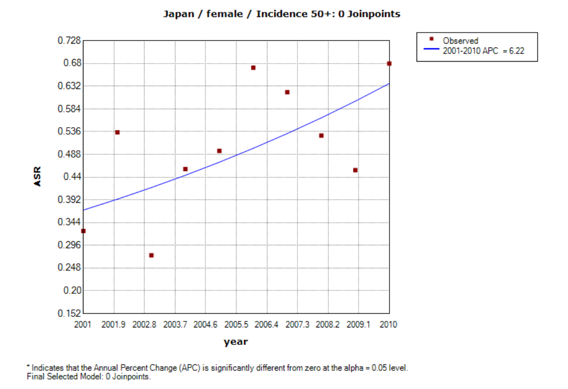

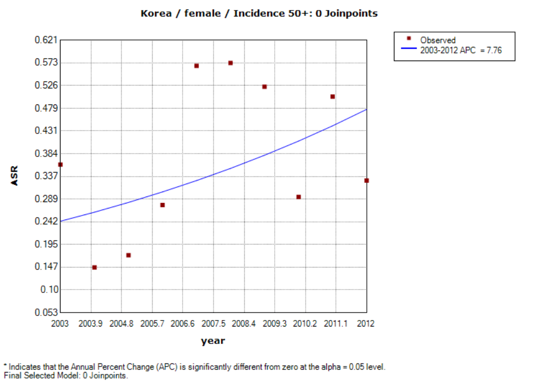
Korea / female / Incidence 50+: 0 Joinpoints
Observed
2003-2012 APC = 7.76
ASR
year
* Indicates that the Annual Percent Change (APC) is significantly different from zero at the alpha = 0.05 level.
Final Selected Model: 0 Joinpoints.

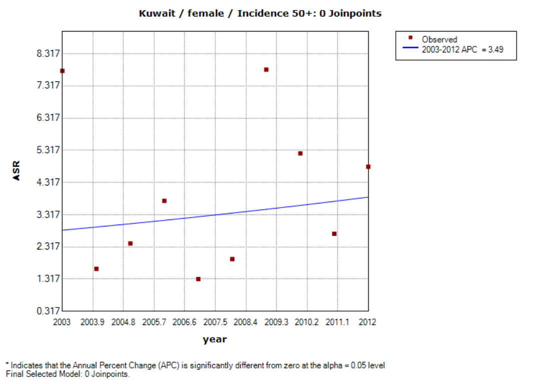
Kuwait / female / Incidence 50+: 0 Joinpoints
Observed
2003-2012 APC = 3.49
ASR
year
* Indicates that the Annual Percent Change (APC) is significantly different from zero at the alpha = 0.05 level
Final Selected Model: 0 Joinpoints.

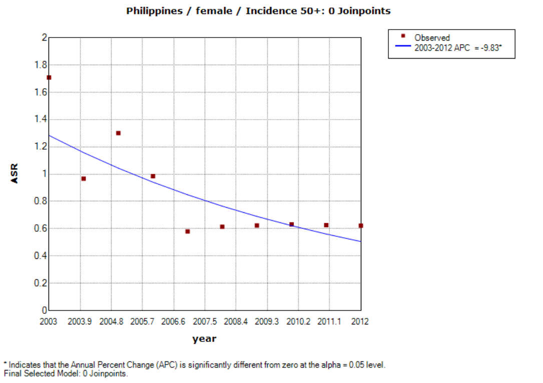
Philippines / female / Incidence 50+: 0 Joinpoints
Observed
2003-2012 APC = -9.83*
ASR
year
* Indicates that the Annual Percent Change (APC) is significantly different from zero at the alpha = 0.05 level.
Final Selected Model: 0 Joinpoints.

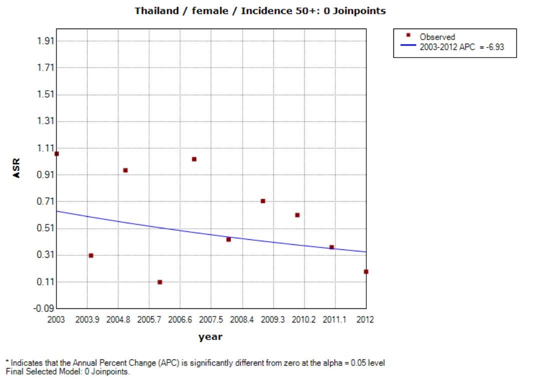
Thailand / female / Incidence 50+: 0 Joinpoints
Observed
2003-2012 APC = -6.93
ASR
year
* Indicates that the Annual Percent Change (APC) is significantly different from zero at the alpha = 0.05 level
Final Selected Model: 0 Joinpoints.

Oceania

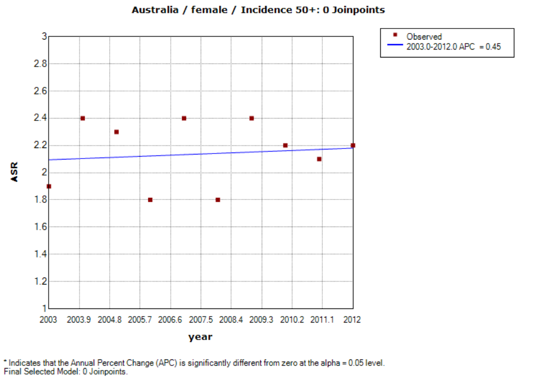
Australia / female / Incidence 50+: 0 Joinpoints
Observed
2003.0-2012.0 APC = 0.45
ASR
year
* Indicates that the Annual Percent Change (APC) is significantly different from zero at the alpha = 0.05 level.
Final Selected Model: 0 Joinpoints.

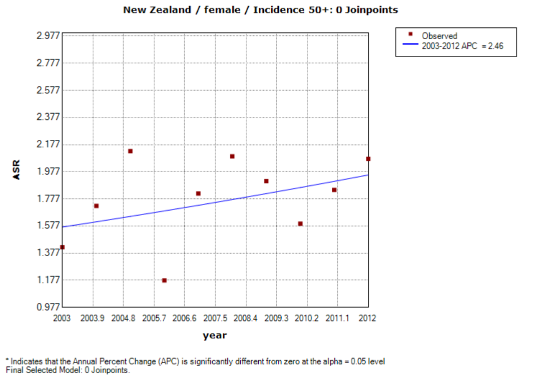
New Zealand / female / Incidence 50+: 0 Joinpoints
Observed
2003-2012 APC = 2.46
ASR
year
* Indicates that the Annual Percent Change (APC) is significantly different from zero at the alpha = 0.05 level
Final Selected Model: 0 Joinpoints.

North America

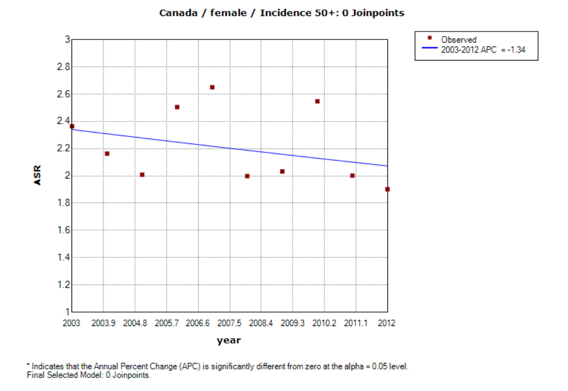

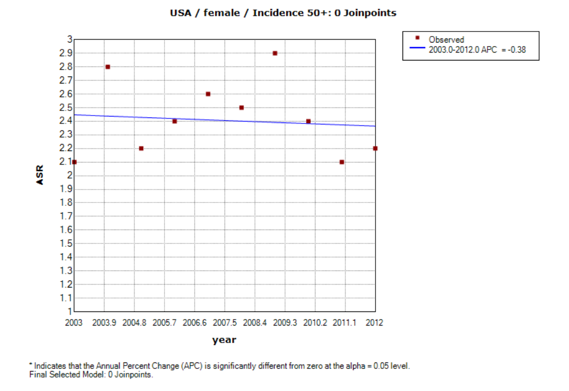

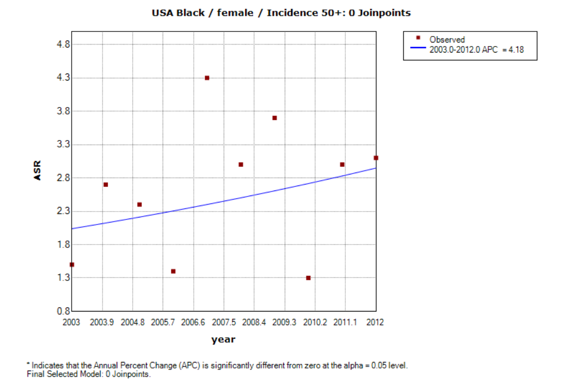

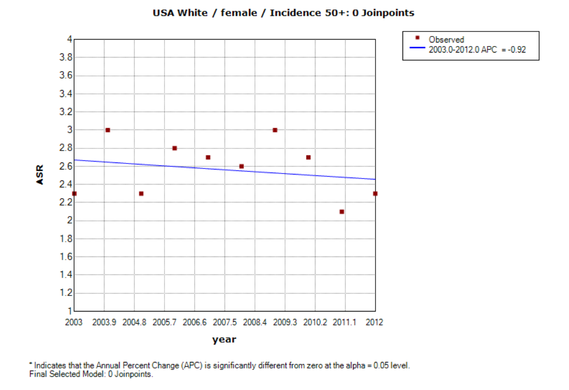

South America

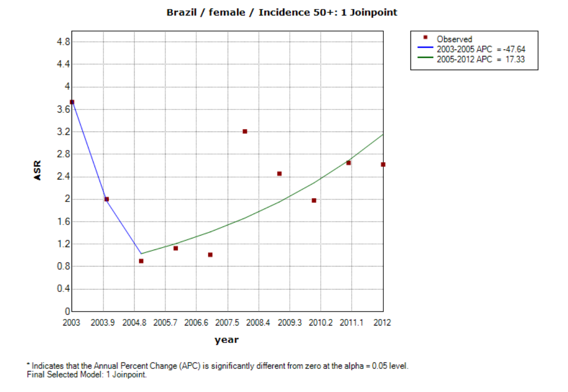

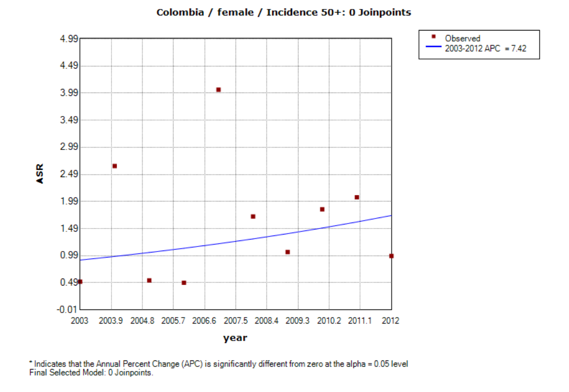

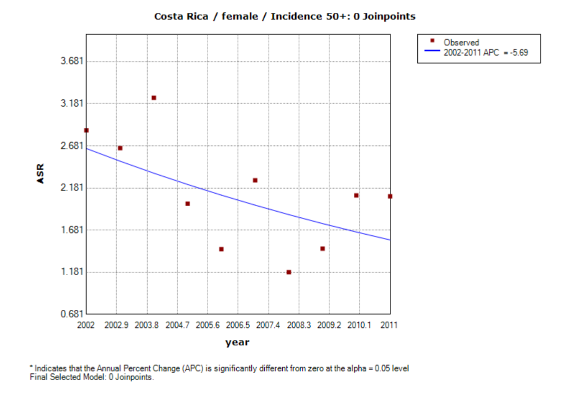

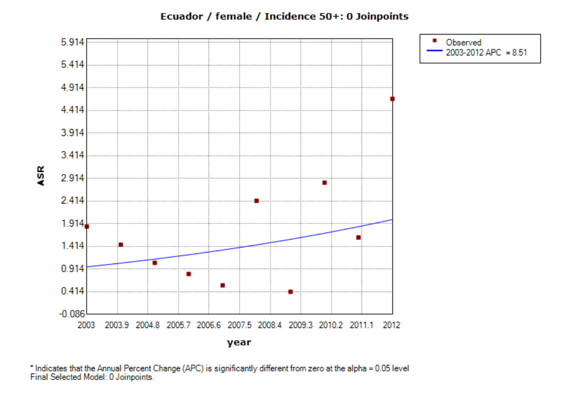

Northern Europe

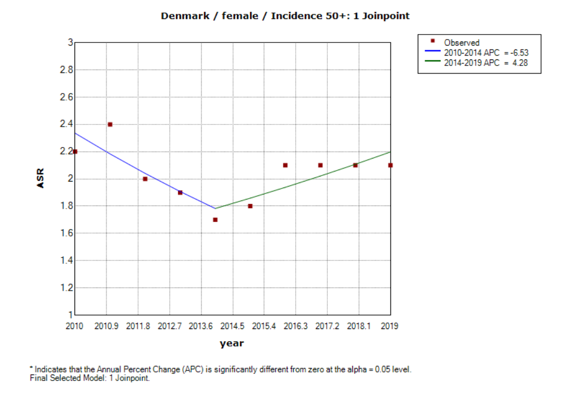

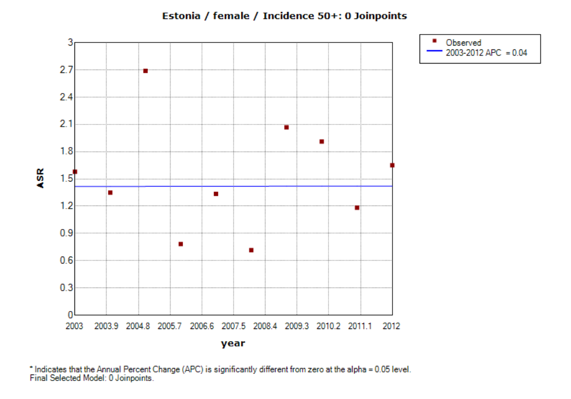

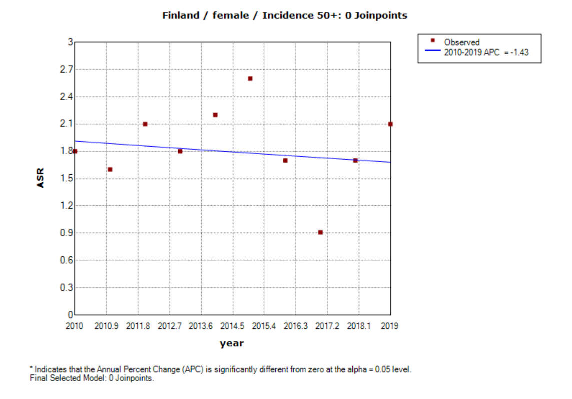

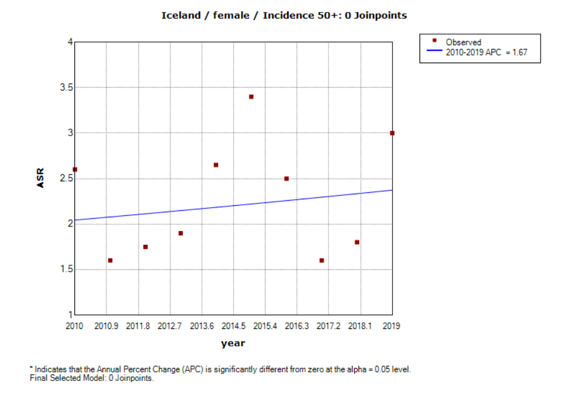

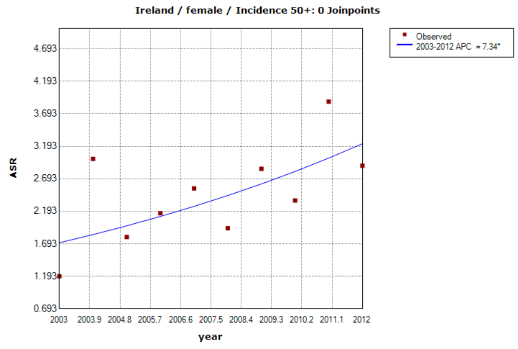

* Indicates that the Annual Percent Change (APC) is significantly different from zero at the alpha = 0.05 level
Final Selected Model: 0 Joinpoints.

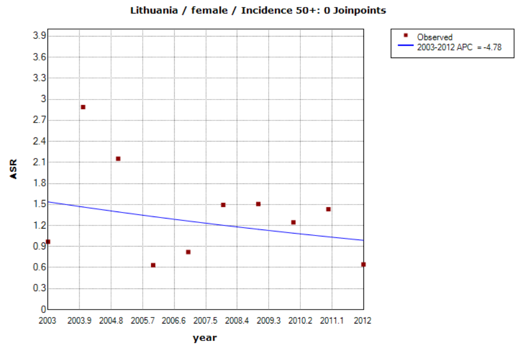

* Indicates that the Annual Percent Change (APC) is significantly different from zero at the alpha = 0.05 level.
Final Selected Model: 0 Joinpoints.

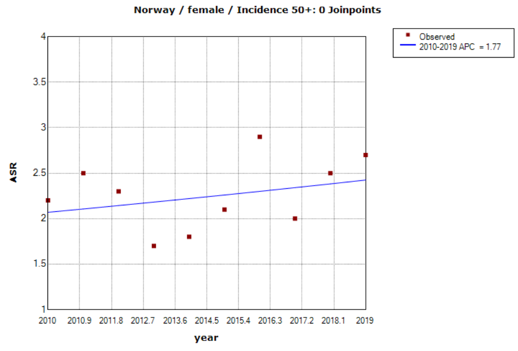

* Indicates that the Annual Percent Change (APC) is significantly different from zero at the alpha = 0.05 level.
Final Selected Model: 0 Joinpoints.

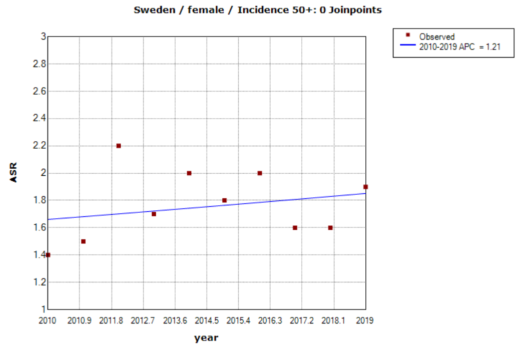

* Indicates that the Annual Percent Change (APC) is significantly different from zero at the alpha = 0.05 level.
Final Selected Model: 0 Joinpoints.

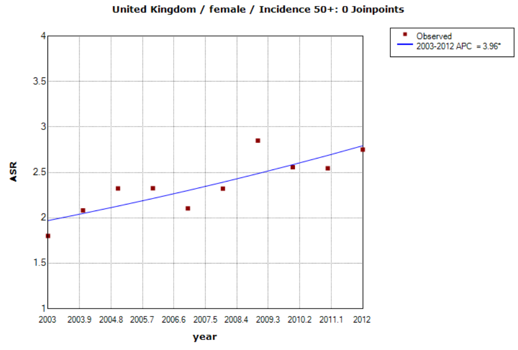

* Indicates that the Annual Percent Change (APC) is significantly different from zero at the alpha = 0.05 level.
Final Selected Model: 0 Joinpoints.

Western Europe

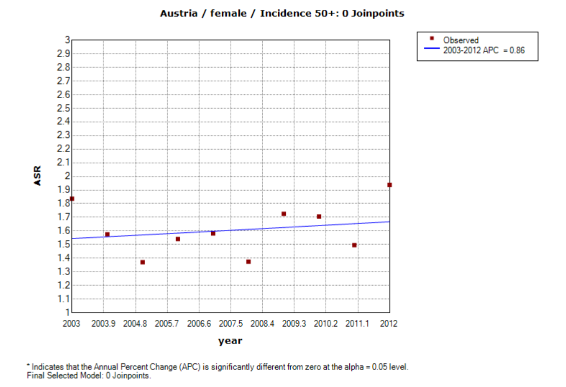

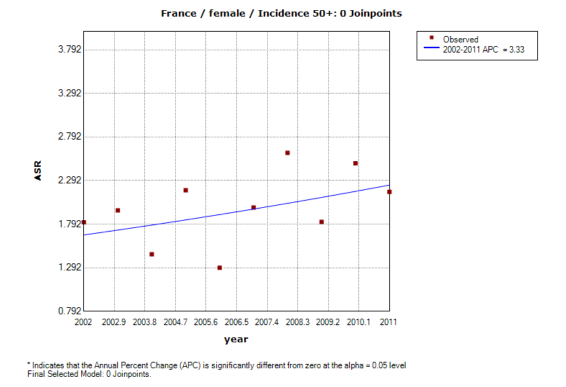

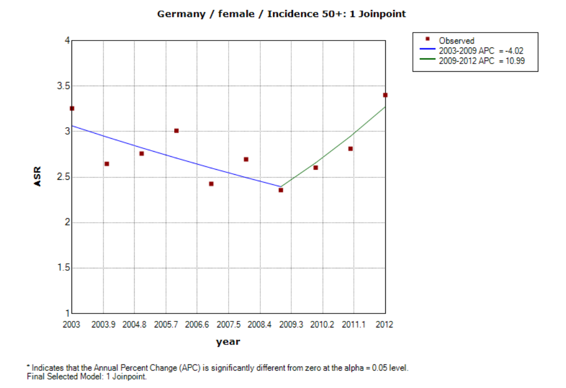

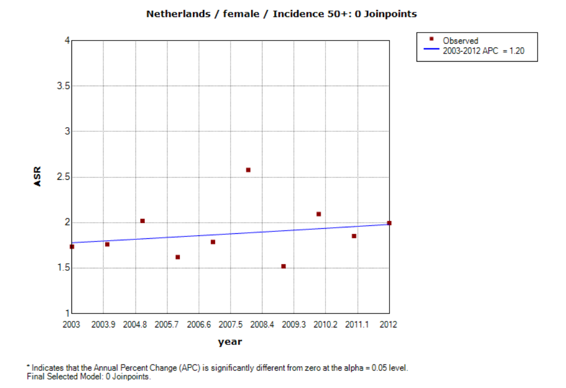

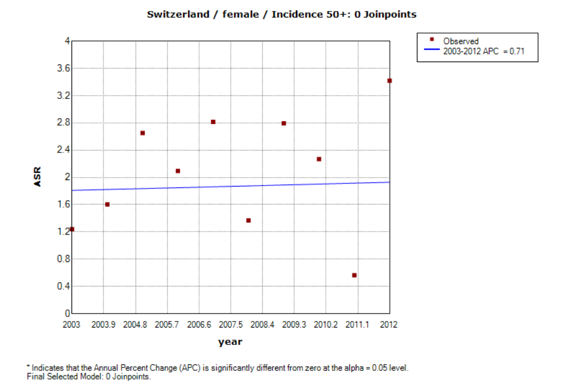

Southern Europe

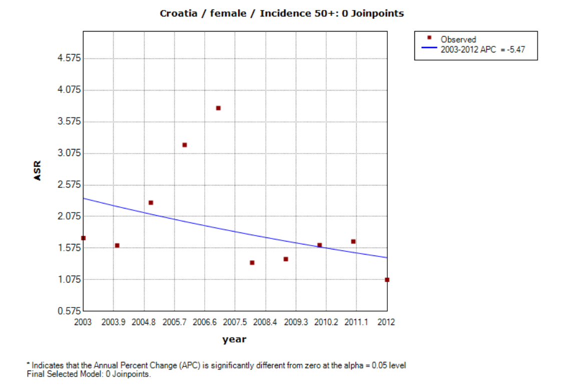

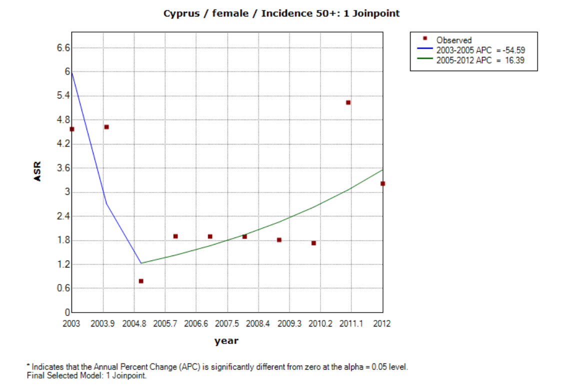

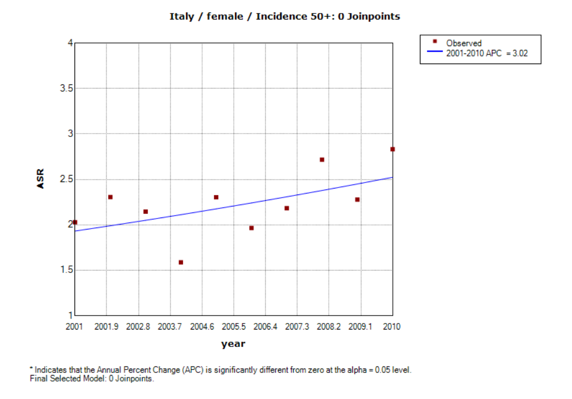

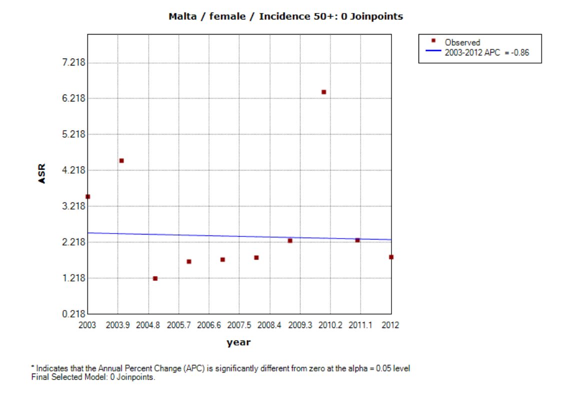

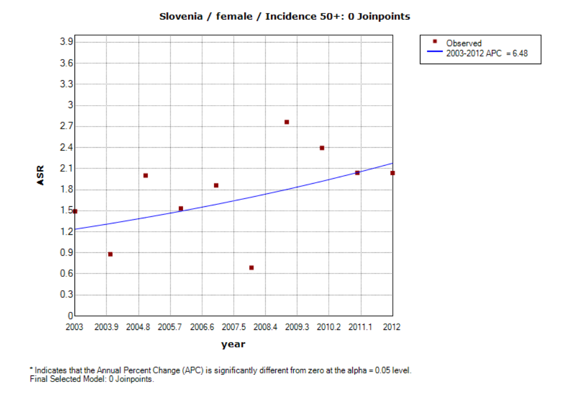

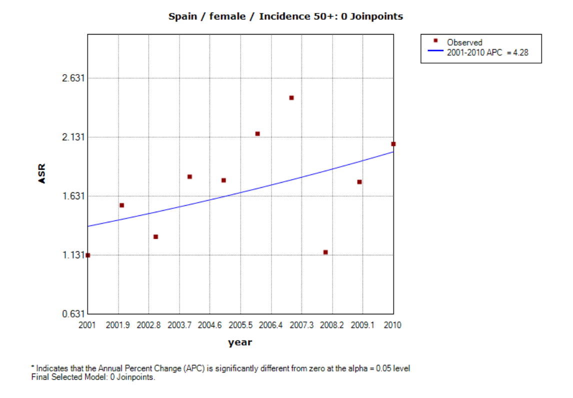

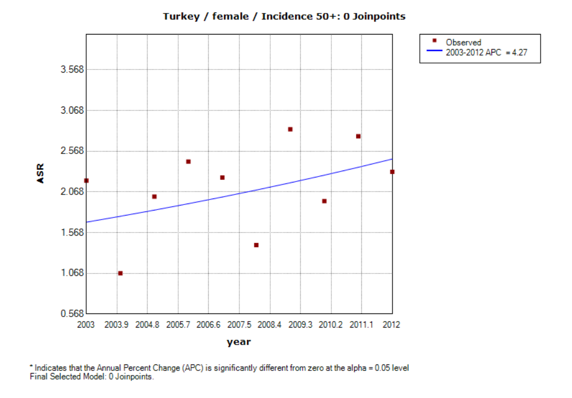

Eastern Europe

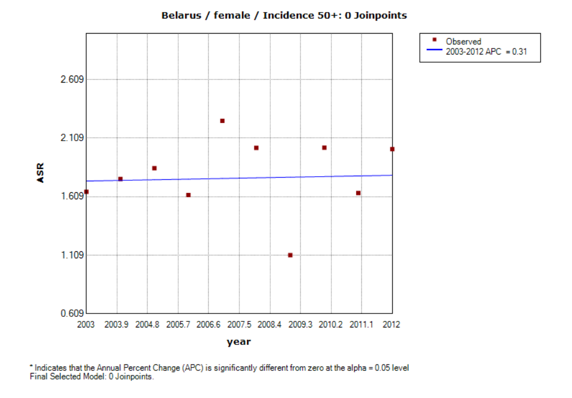

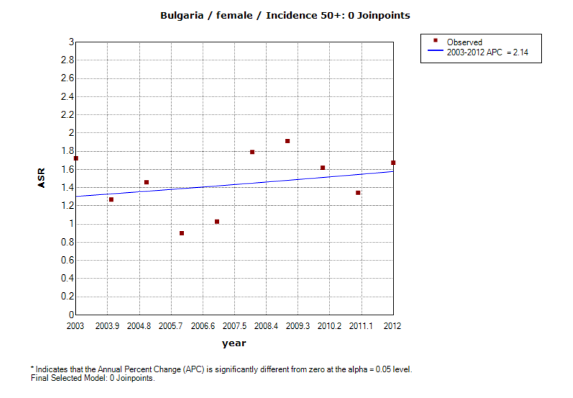

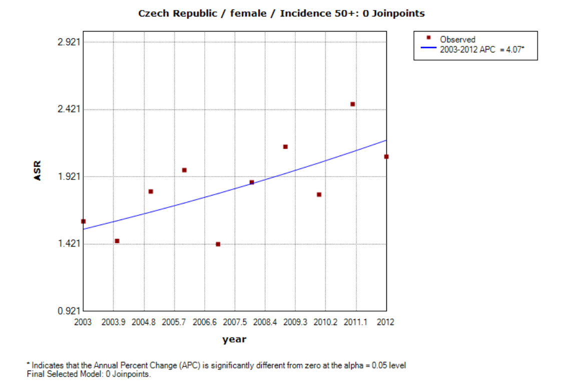
Czech Republic / female / Incidence 50+: 0 Joinpoints
Observed
2003-2012 APC = 4.07*
ASR
year
* Indicates that the Annual Percent Change (APC) is significantly different from zero at the alpha = 0.05 level
Final Selected Model: 0 Joinpoints.

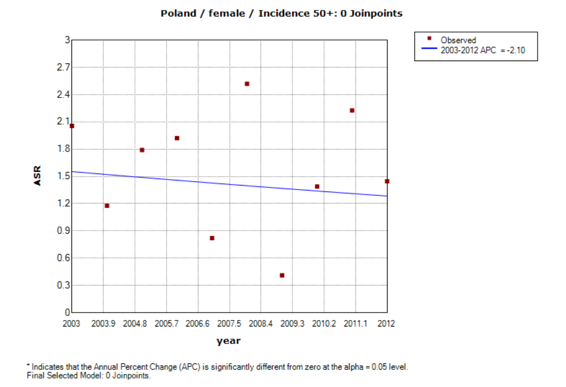
Poland / female / Incidence 50+: 0 Joinpoints
Observed
2003-2012 APC = -2.10
ASR
year
* Indicates that the Annual Percent Change (APC) is significantly different from zero at the alpha = 0.05 level.
Final Selected Model: 0 Joinpoints.

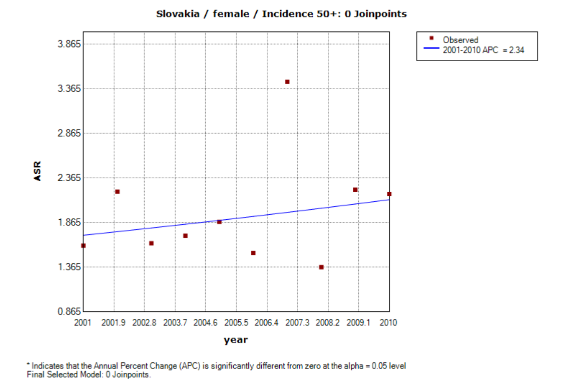
Slovakia / female / Incidence 50+: 0 Joinpoints
Observed
2001-2010 APC = 2.34
ASR
year
* Indicates that the Annual Percent Change (APC) is significantly different from zero at the alpha = 0.05 level
Final Selected Model: 0 Joinpoints.

Africa

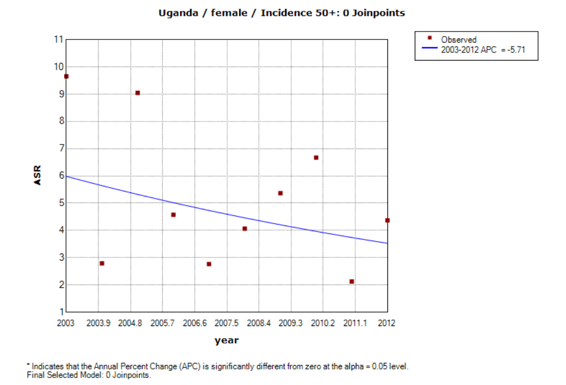
Uganda / female / Incidence 50+: 0 Joinpoints
Observed
2003-2012 APC = -5.71
ASR
year
* Indicates that the Annual Percent Change (APC) is significantly different from zero at the alpha = 0.05 level.
Final Selected Model: 0 Joinpoints.

i.) Mortality male all ages

Asia

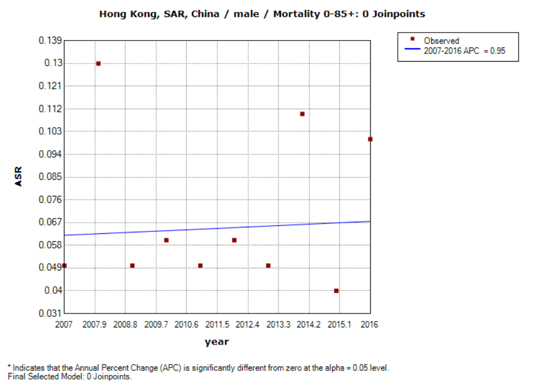

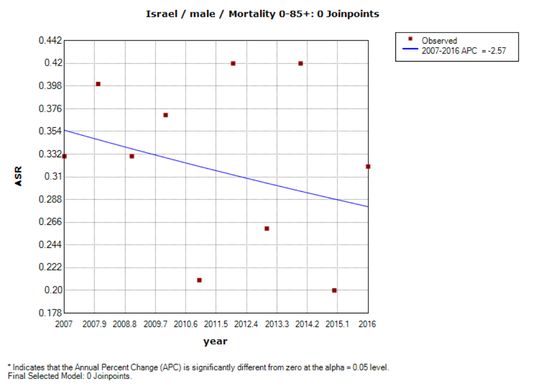

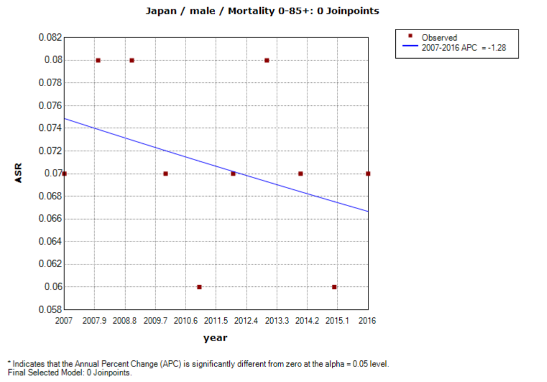

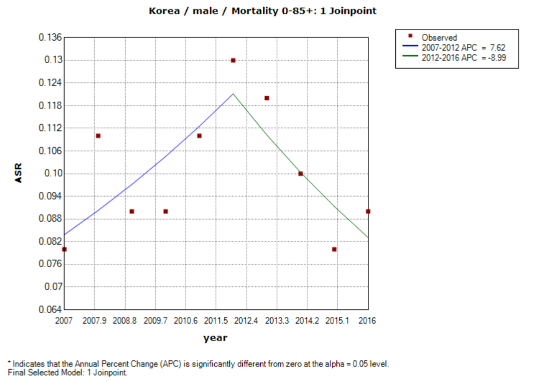

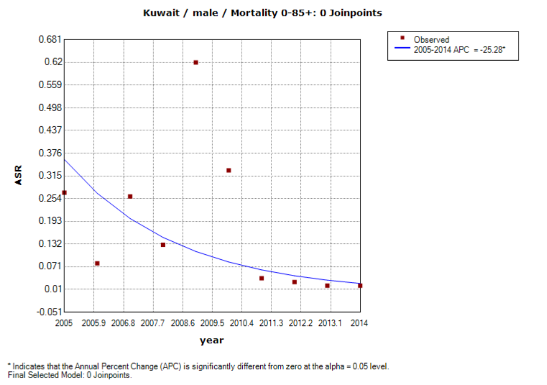

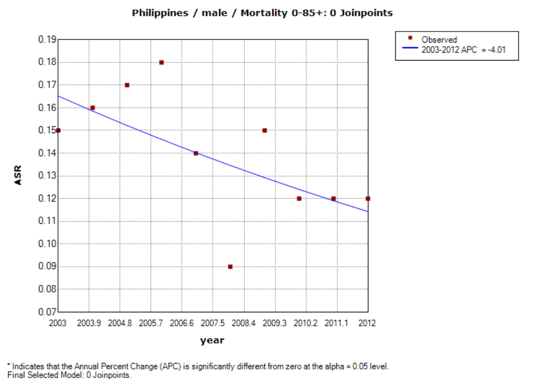

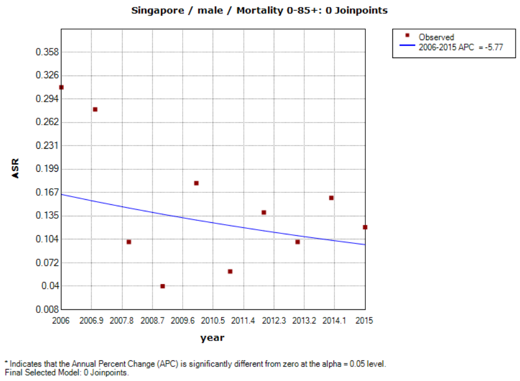
Singapore / male / Mortality 0-85+: 0 Joinpoints
Observed
2006-2015 APC = -5.77
ASR
0.358
0.326
0.294
0.262
0.231
0.199
0.167
0.135
0.104
0.072
0.04
0.008
2006
2006.9
2007.8
2008.7
2009.6
2010.5
2011.4
2012.3
2013.2
2014.1
2015
year
* Indicates that the Annual Percent Change (APC) is significantly different from zero at the alpha = 0.05 level.
Final Selected Model: 0 Joinpoints.

Oceania

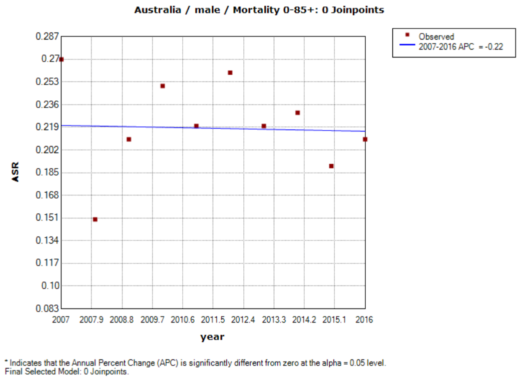
Australia / male / Mortality 0-85+: 0 Joinpoints
Observed
2007-2016 APC = -0.22
ASR
0.287
0.27
0.253
0.236
0.219
0.202
0.185
0.168
0.151
0.134
0.117
0.10
0.083
2007
2007.9
2008.8
2009.7
2010.6
2011.5
2012.4
2013.3
2014.2
2015.1
2016
year
* Indicates that the Annual Percent Change (APC) is significantly different from zero at the alpha = 0.05 level.
Final Selected Model: 0 Joinpoints.

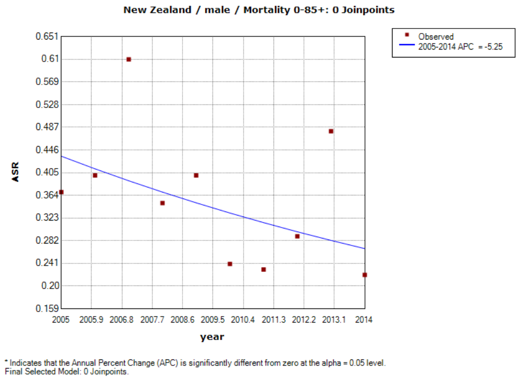
New Zealand / male / Mortality 0-85+: 0 Joinpoints
Observed
2005-2014 APC = -5.25
ASR
0.651
0.61
0.569
0.528
0.487
0.446
0.405
0.364
0.323
0.282
0.241
0.20
0.159
2005
2005.9
2006.8
2007.7
2008.6
2009.5
2010.4
2011.3
2012.2
2013.1
2014
year
* Indicates that the Annual Percent Change (APC) is significantly different from zero at the alpha = 0.05 level.
Final Selected Model: 0 Joinpoints.

North America

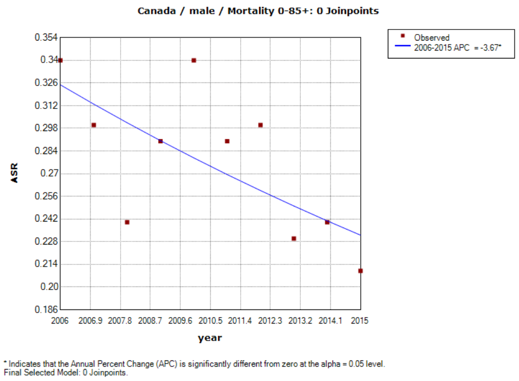

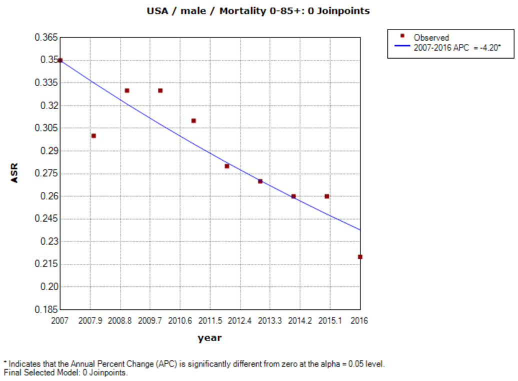

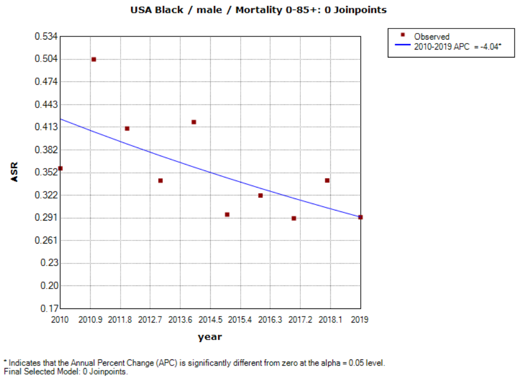

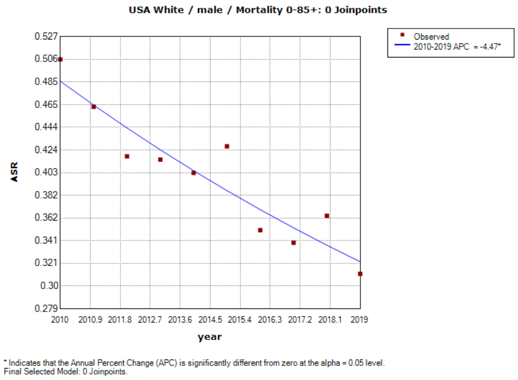

South America

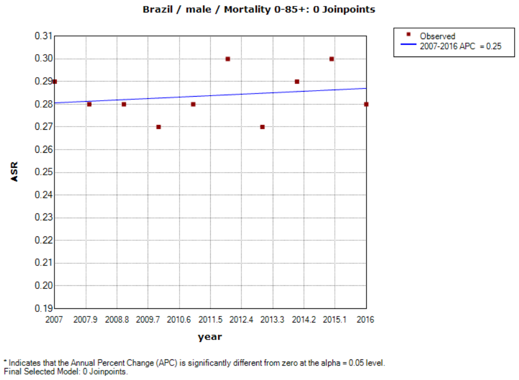

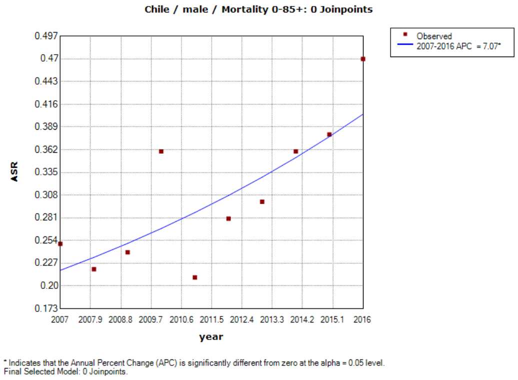

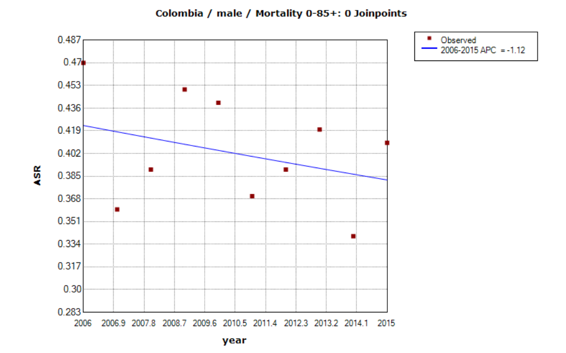

* Indicates that the Annual Percent Change (APC) is significantly different from zero at the alpha = 0.05 level.
Final Selected Model: 0 Joinpoints.

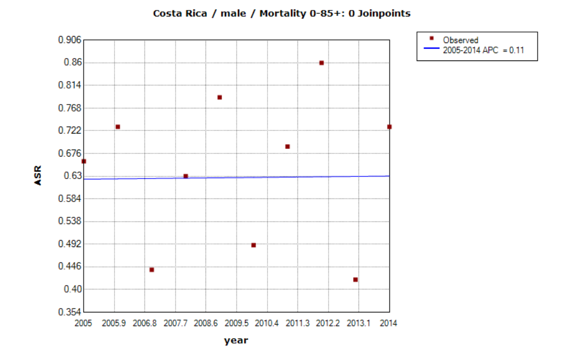

* Indicates that the Annual Percent Change (APC) is significantly different from zero at the alpha = 0.05 level.
Final Selected Model: 0 Joinpoints.

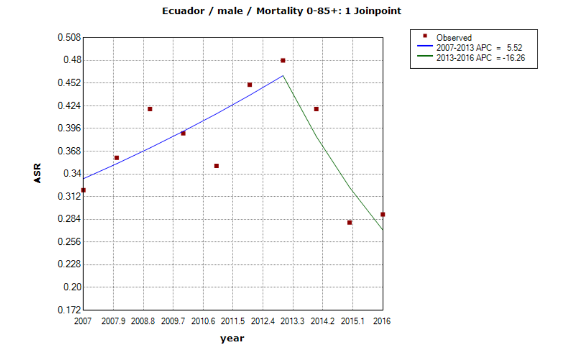

* Indicates that the Annual Percent Change (APC) is significantly different from zero at the alpha = 0.05 level.
Final Selected Model: 1 Joinpoint.

Northern Europe

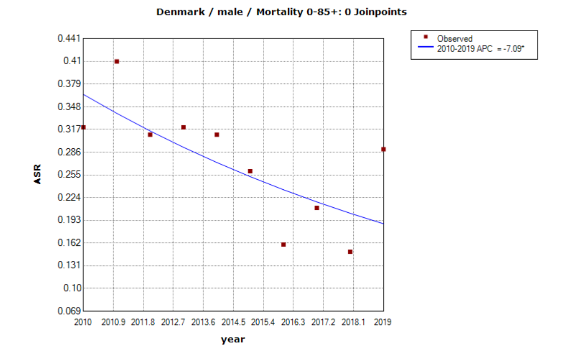

* Indicates that the Annual Percent Change (APC) is significantly different from zero at the alpha = 0.05 level.
Final Selected Model: 0 Joinpoints.

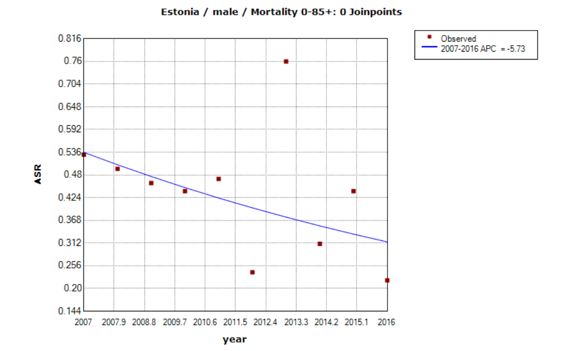

* Indicates that the Annual Percent Change (APC) is significantly different from zero at the alpha = 0.05 level.
Final Selected Model: 0 Joinpoints.

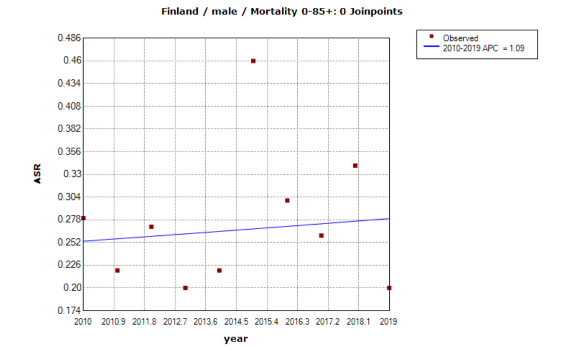

* Indicates that the Annual Percent Change (APC) is significantly different from zero at the alpha = 0.05 level.
Final Selected Model: 0 Joinpoints.

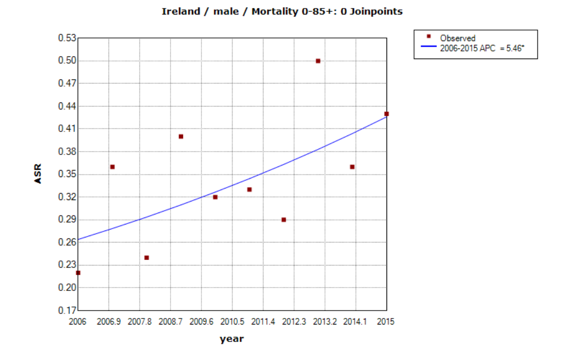

* Indicates that the Annual Percent Change (APC) is significantly different from zero at the alpha = 0.05 level.
Final Selected Model: 0 Joinpoints.

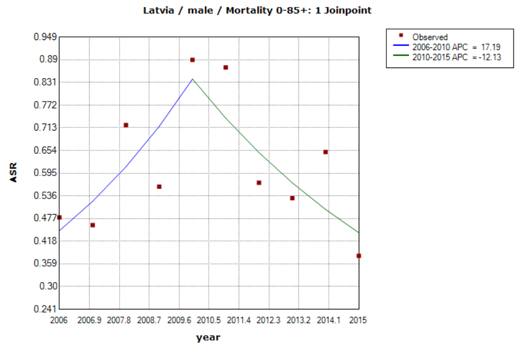

* Indicates that the Annual Percent Change (APC) is significantly different from zero at the alpha = 0.05 level.
Final Selected Model: 1 Joinpoint.

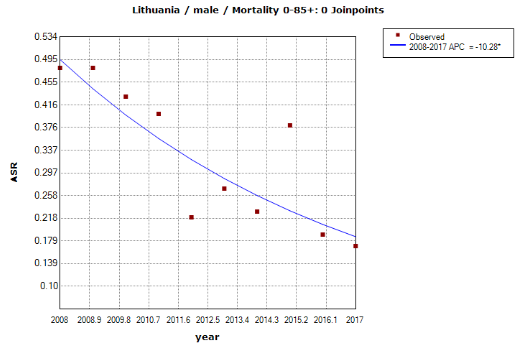

* Indicates that the Annual Percent Change (APC) is significantly different from zero at the alpha = 0.05 level.
Final Selected Model: 0 Joinpoints.

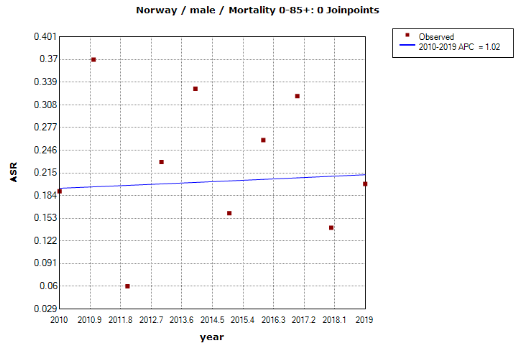

* Indicates that the Annual Percent Change (APC) is significantly different from zero at the alpha = 0.05 level.
Final Selected Model: 0 Joinpoints.

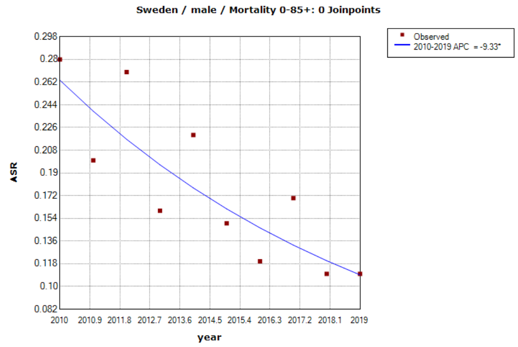

* Indicates that the Annual Percent Change (APC) is significantly different from zero at the alpha = 0.05 level.
Final Selected Model: 0 Joinpoints.

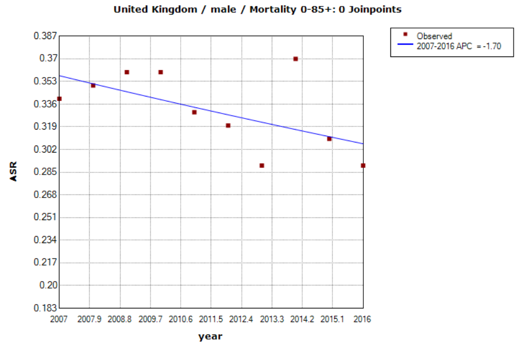

* Indicates that the Annual Percent Change (APC) is significantly different from zero at the alpha = 0.05 level.
Final Selected Model: 0 Joinpoints.

Western Europe

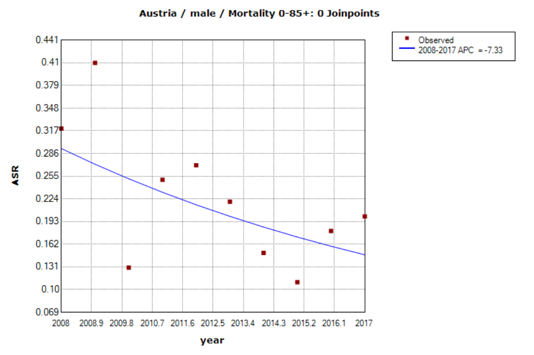

* Indicates that the Annual Percent Change (APC) is significantly different from zero at the alpha = 0.05 level.
Final Selected Model: 0 Joinpoints.

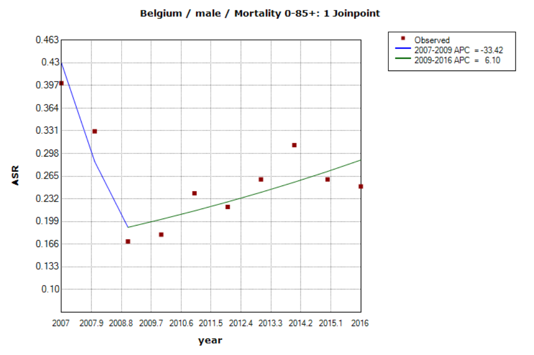

* Indicates that the Annual Percent Change (APC) is significantly different from zero at the alpha = 0.05 level.
Final Selected Model: 1 Joinpoint.

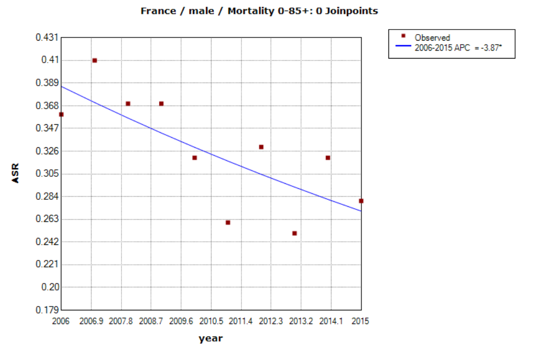

* Indicates that the Annual Percent Change (APC) is significantly different from zero at the alpha = 0.05 level.
Final Selected Model: 0 Joinpoints.

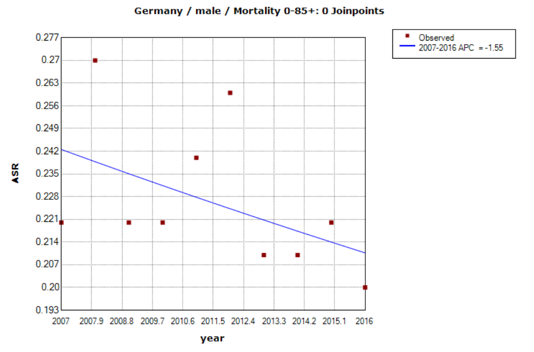

* Indicates that the Annual Percent Change (APC) is significantly different from zero at the alpha = 0.05 level.
Final Selected Model: 0 Joinpoints.

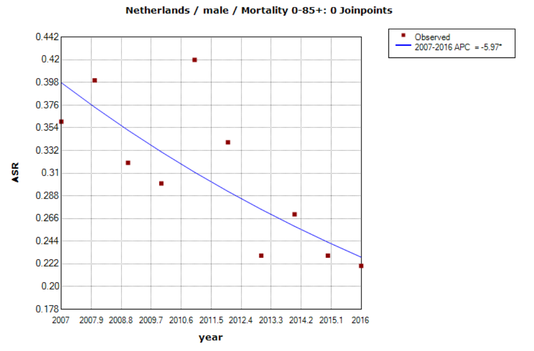

* Indicates that the Annual Percent Change (APC) is significantly different from zero at the alpha = 0.05 level.
Final Selected Model: 0 Joinpoints.

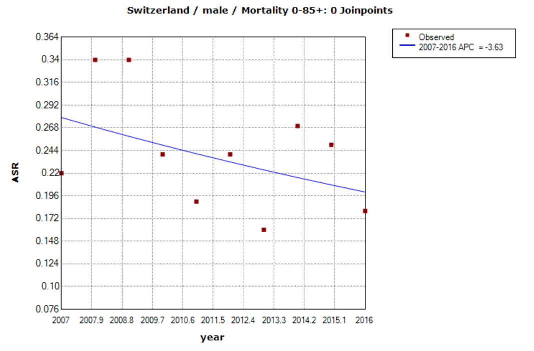

* Indicates that the Annual Percent Change (APC) is significantly different from zero at the alpha = 0.05 level.
Final Selected Model: 0 Joinpoints.

Southern Europe

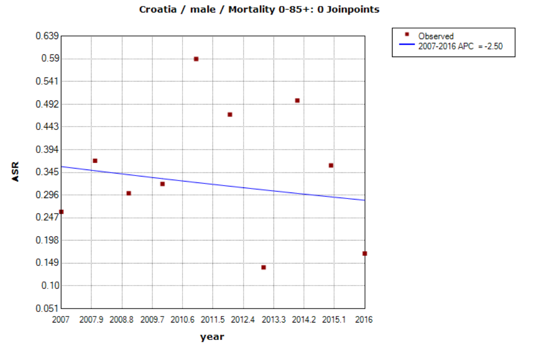

* Indicates that the Annual Percent Change (APC) is significantly different from zero at the alpha = 0.05 level.
Final Selected Model: 0 Joinpoints.

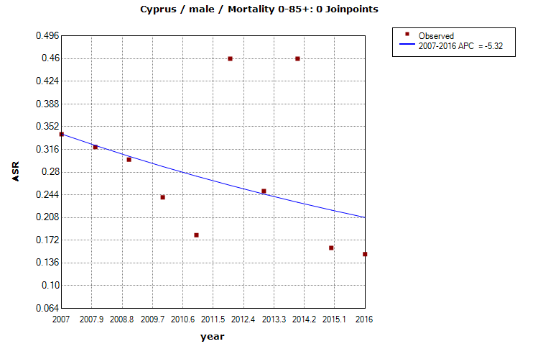

* Indicates that the Annual Percent Change (APC) is significantly different from zero at the alpha = 0.05 level.
Final Selected Model: 0 Joinpoints.

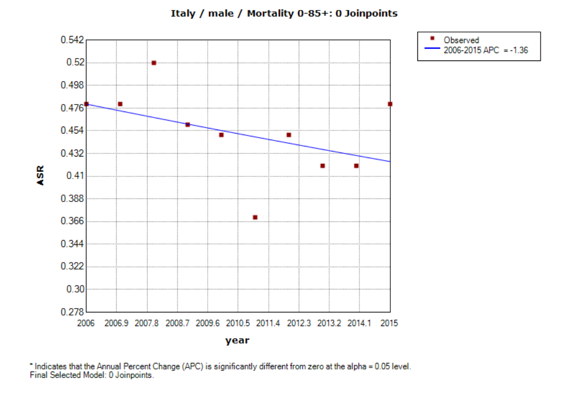

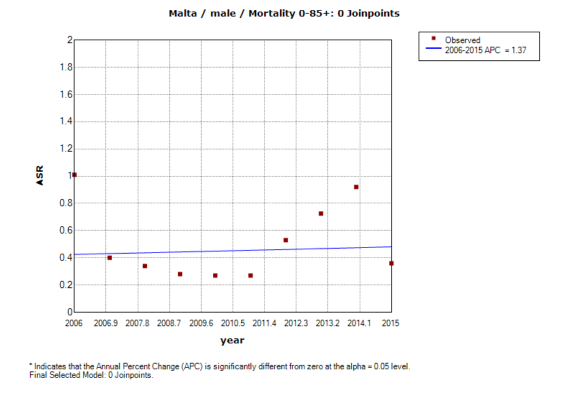

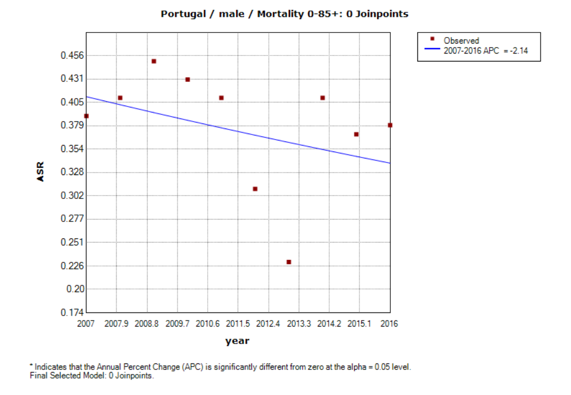

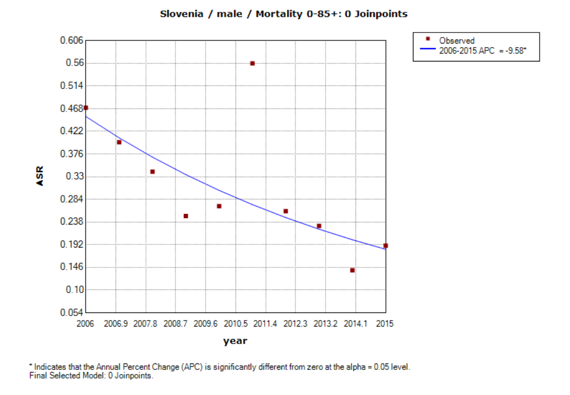

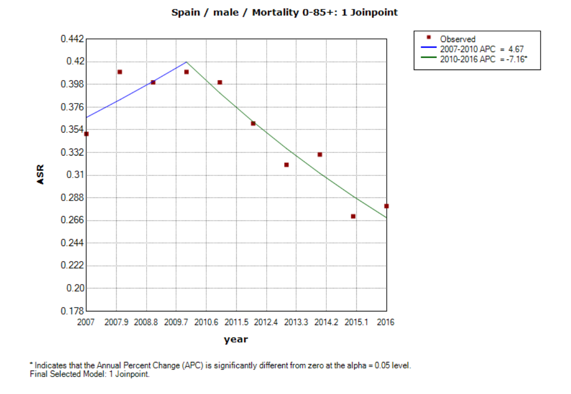

Eastern Europe

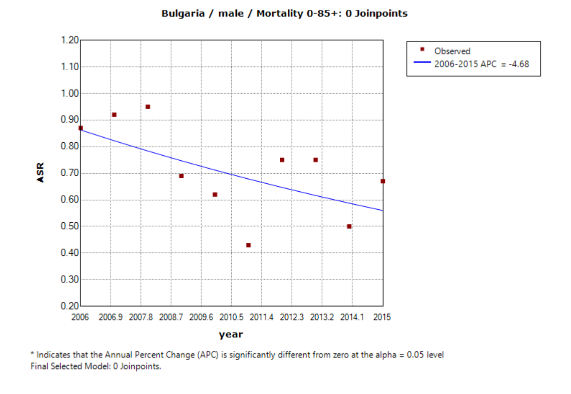

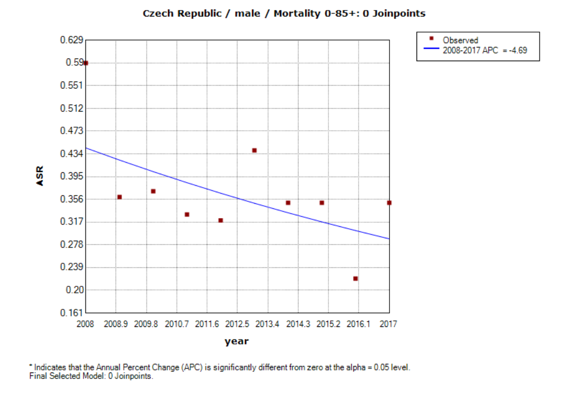

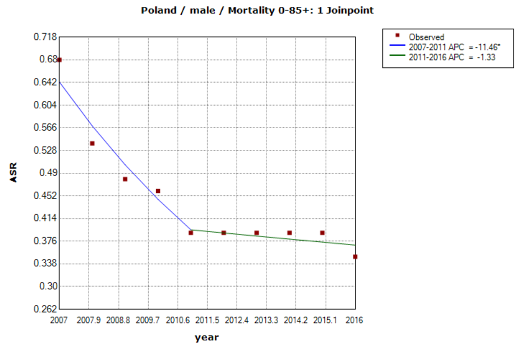

* Indicates that the Annual Percent Change (APC) is significantly different from zero at the alpha = 0.05 level.
Final Selected Model: 1 Joinpoint.

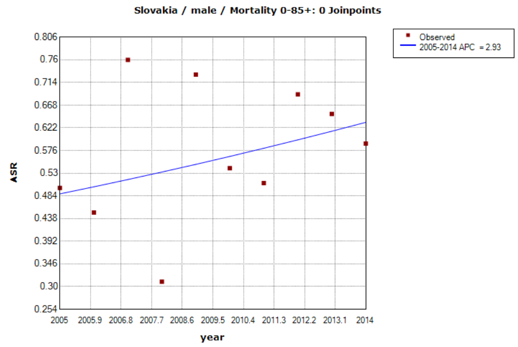

* Indicates that the Annual Percent Change (APC) is significantly different from zero at the alpha = 0.05 level.
Final Selected Model: 0 Joinpoints.

j.) Mortality female all ages

Asia

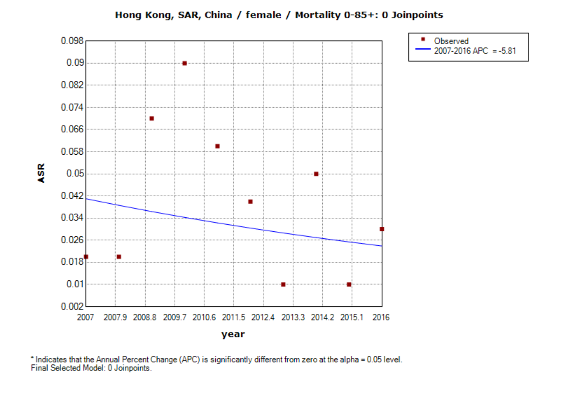

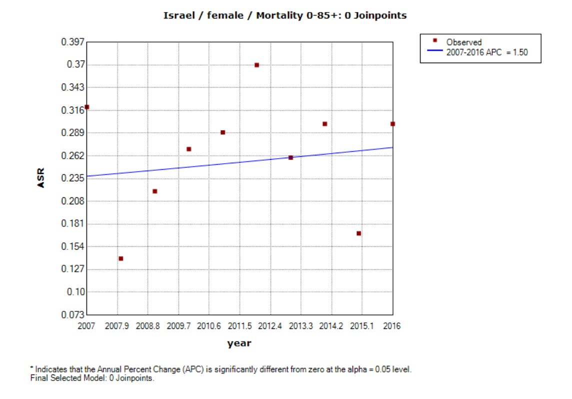

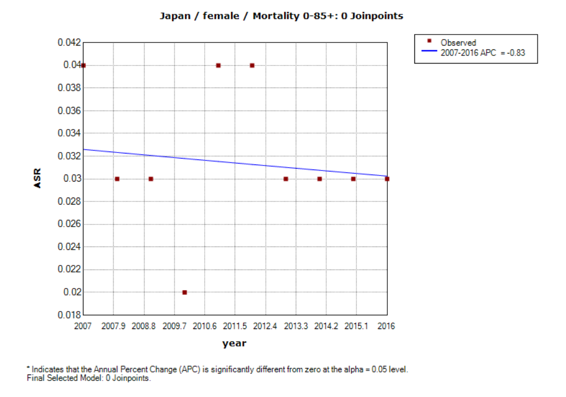

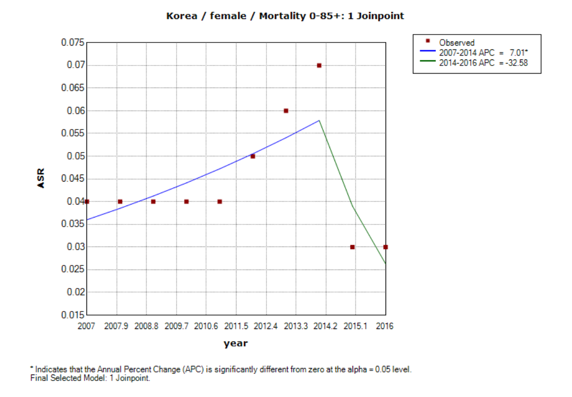

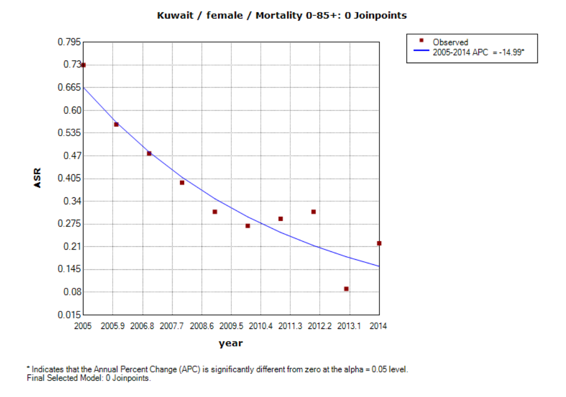

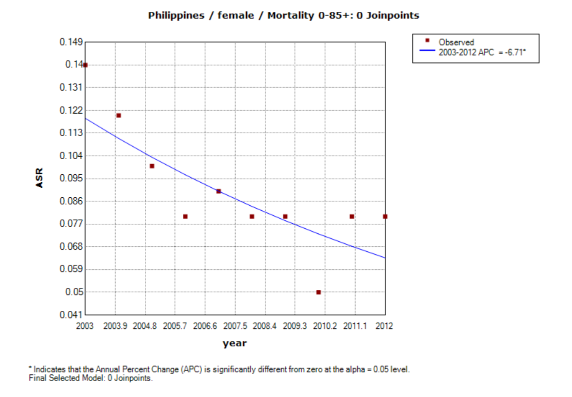

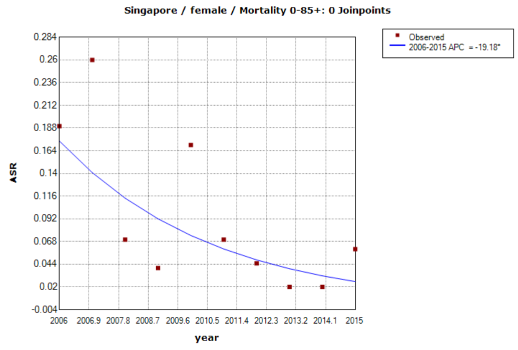

* Indicates that the Annual Percent Change (APC) is significantly different from zero at the alpha = 0.05 level.
Final Selected Model: 0 Joinpoints.

Oceania

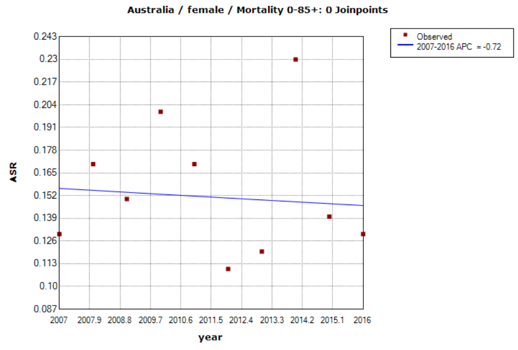

* Indicates that the Annual Percent Change (APC) is significantly different from zero at the alpha = 0.05 level.
Final Selected Model: 0 Joinpoints.

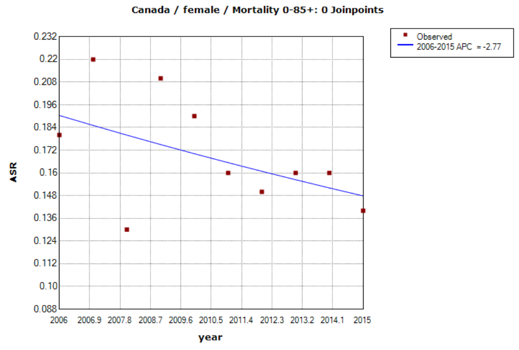

* Indicates that the Annual Percent Change (APC) is significantly different from zero at the alpha = 0.05 level.
Final Selected Model: 0 Joinpoints.

North America

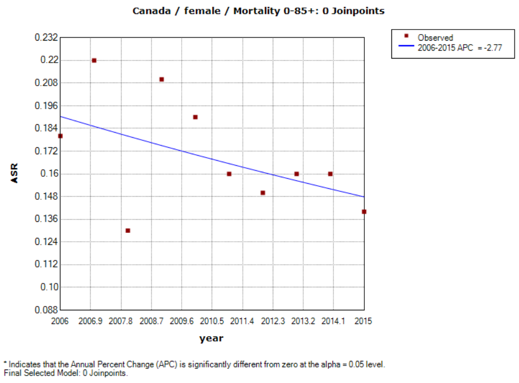

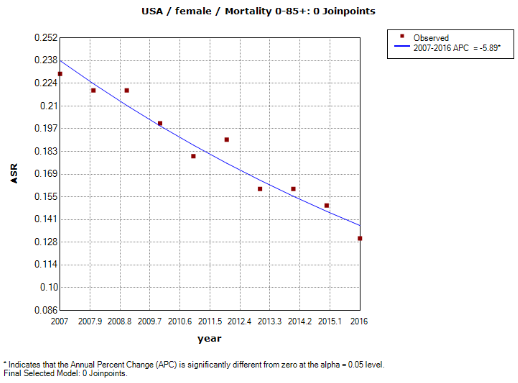

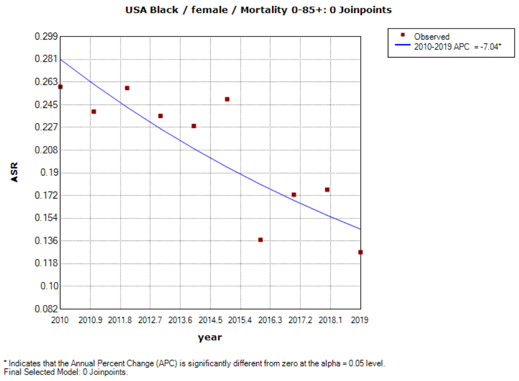

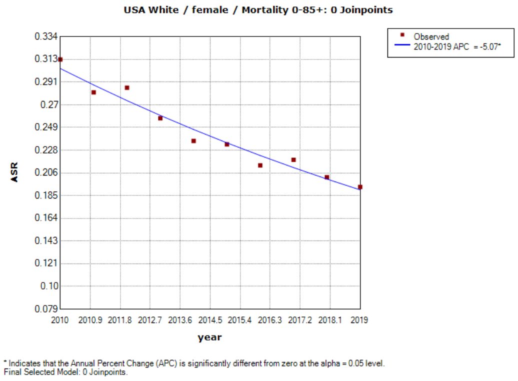

South America

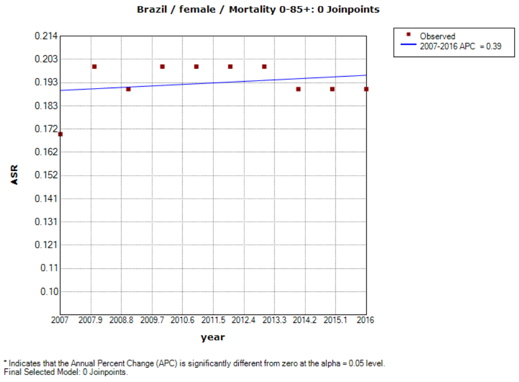

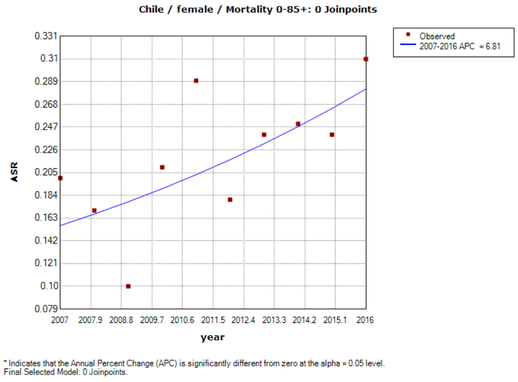

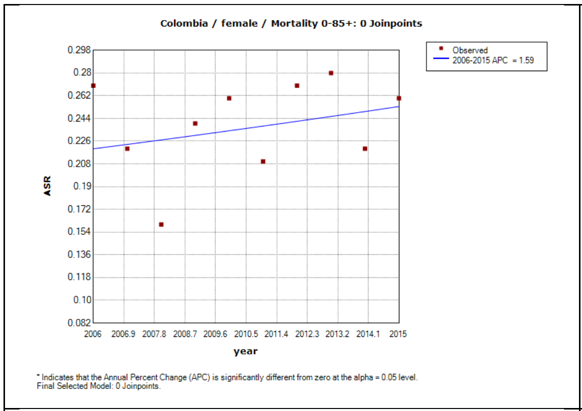

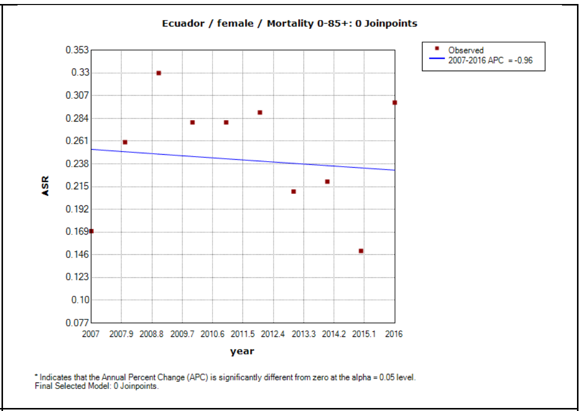

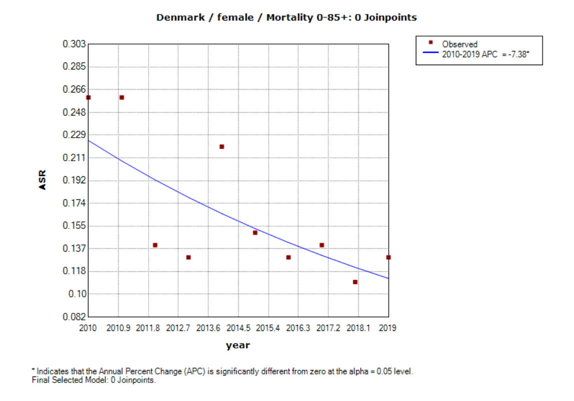

Northern Europe

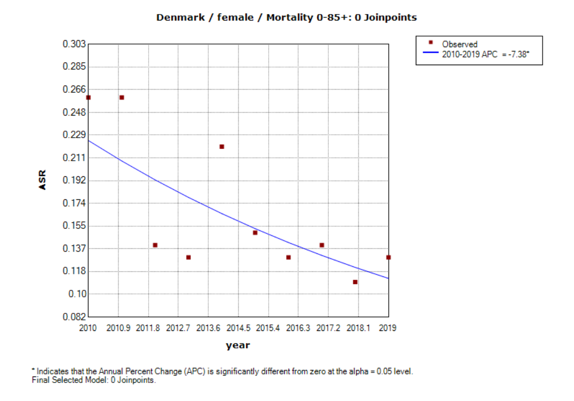

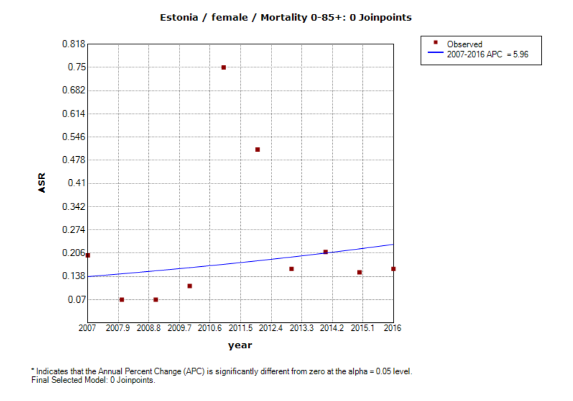

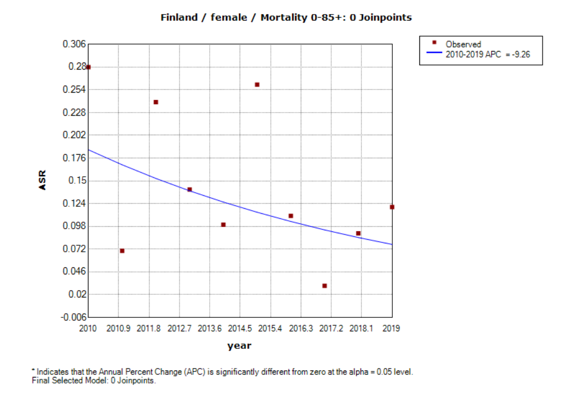

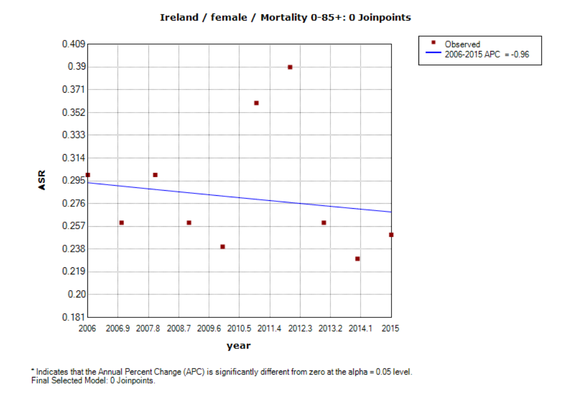

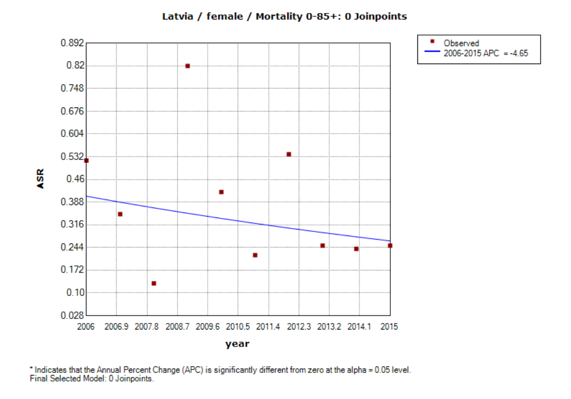

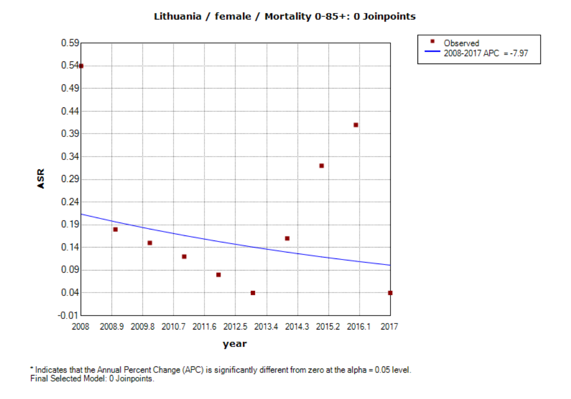

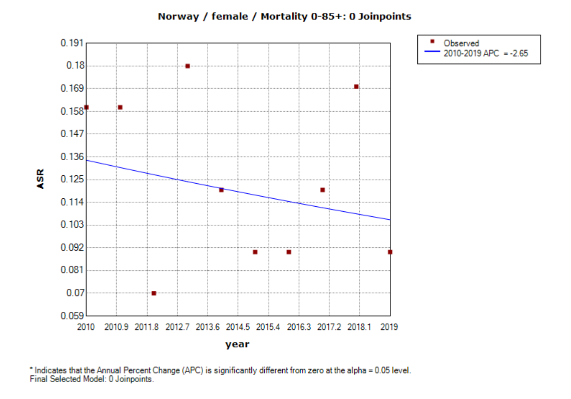

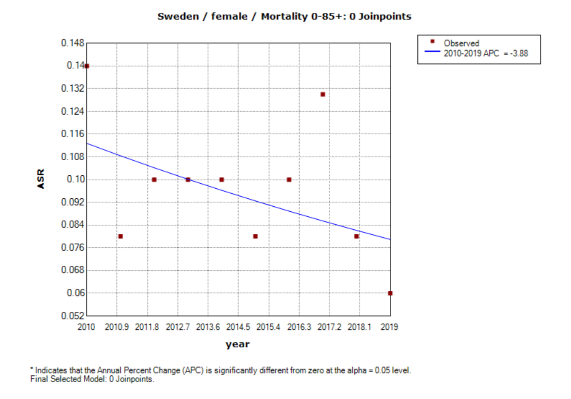

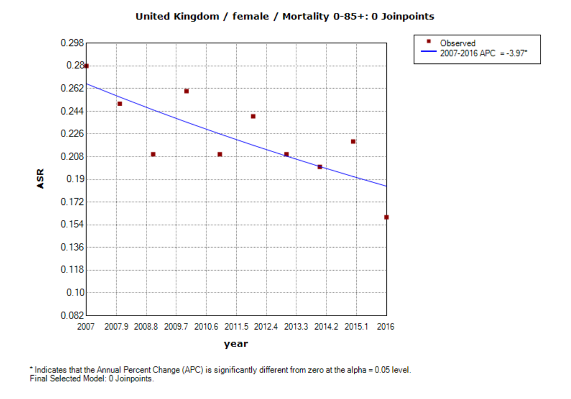

Western Europe

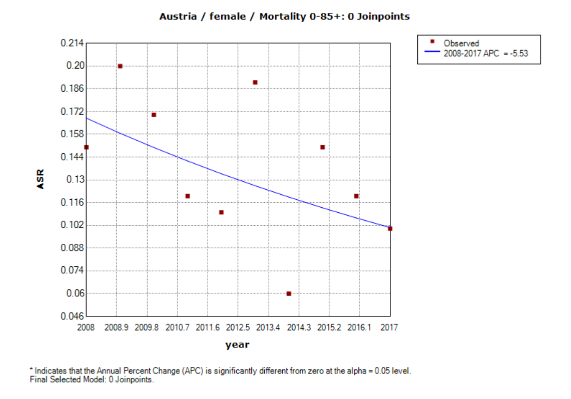

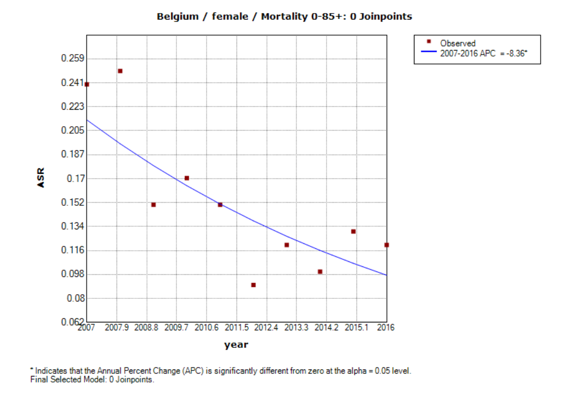

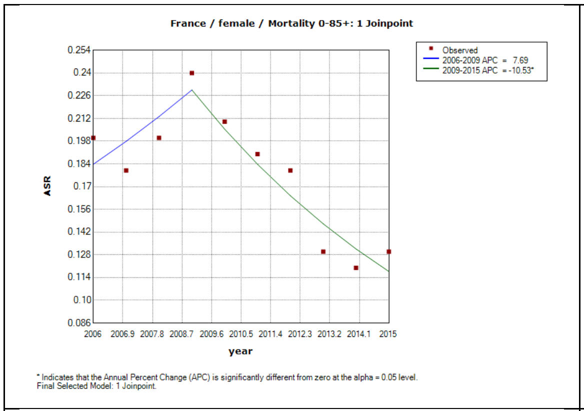

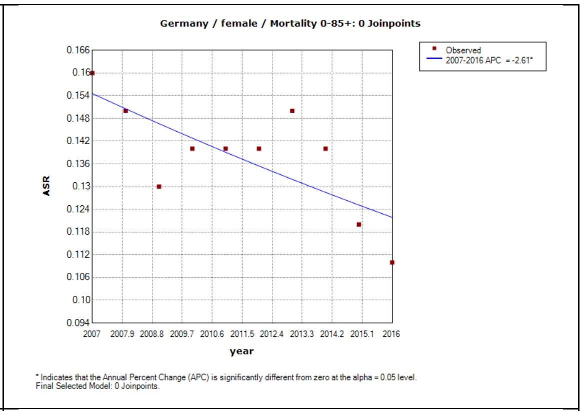

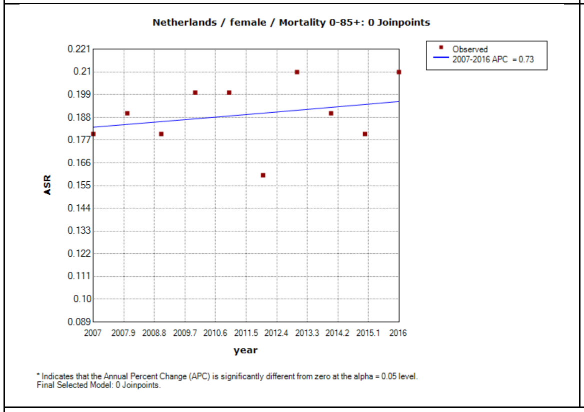

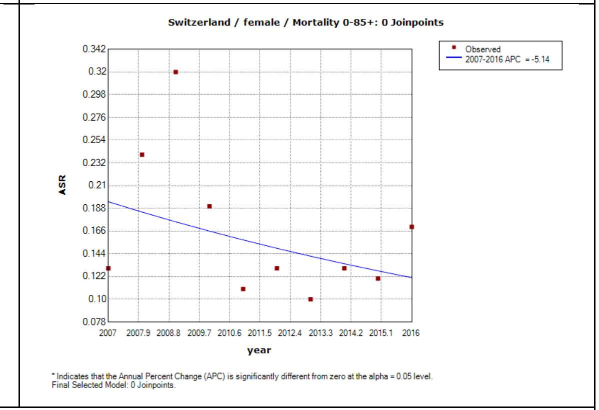

Southern Europe

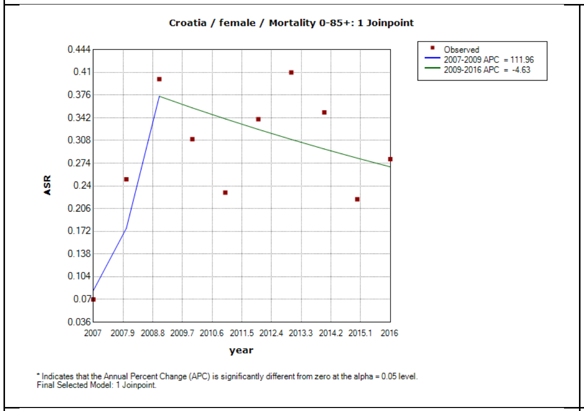

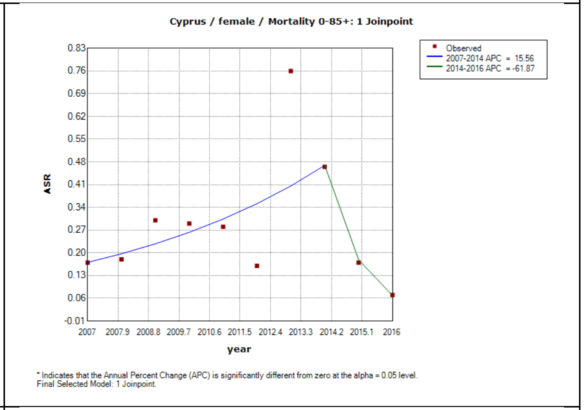

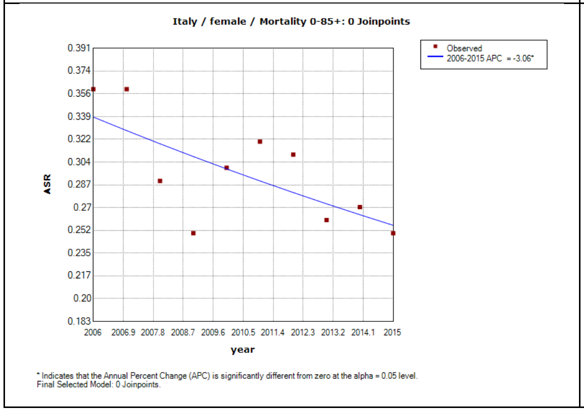

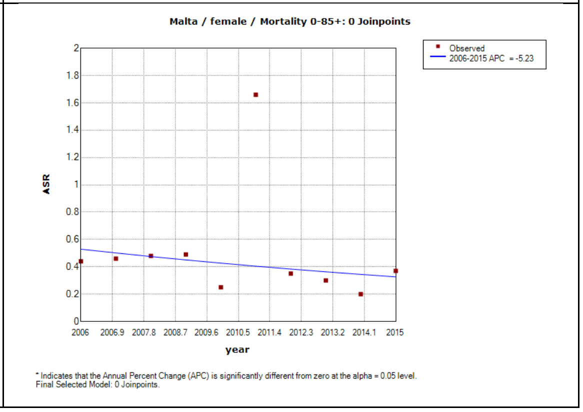

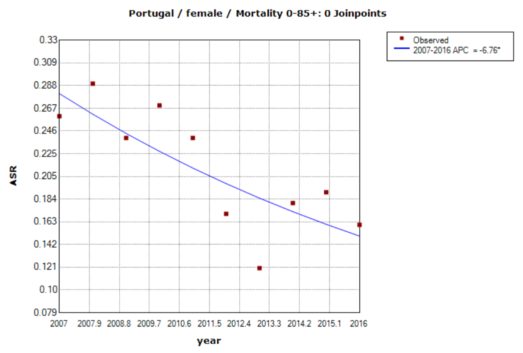

* Indicates that the Annual Percent Change (APC) is significantly different from zero at the alpha = 0.05 level.
Final Selected Model: 0 Joinpoints.

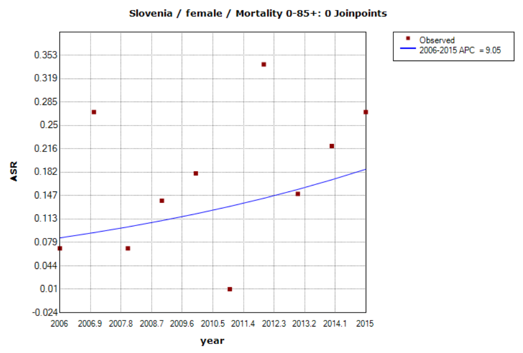

* Indicates that the Annual Percent Change (APC) is significantly different from zero at the alpha = 0.05 level.
Final Selected Model: 0 Joinpoints.

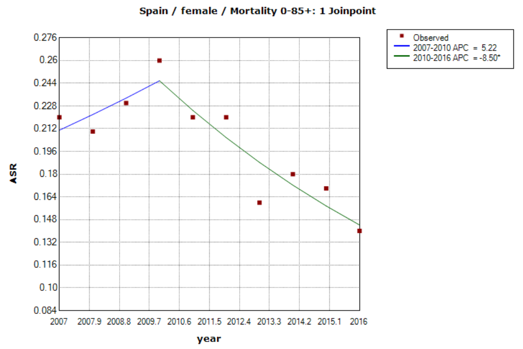

* Indicates that the Annual Percent Change (APC) is significantly different from zero at the alpha = 0.05 level.
Final Selected Model: 1 Joinpoint.

Eastern Europe

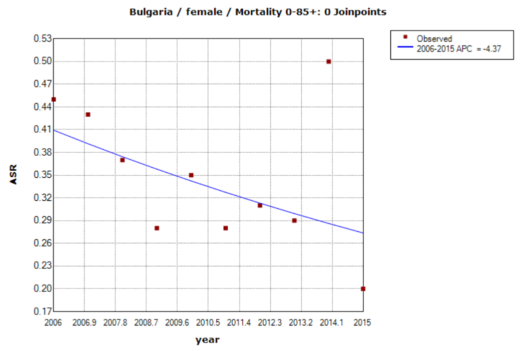

* Indicates that the Annual Percent Change (APC) is significantly different from zero at the alpha = 0.05 level.
Final Selected Model: 0 Joinpoints.

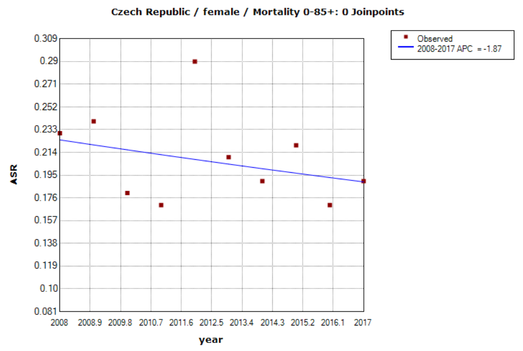

* Indicates that the Annual Percent Change (APC) is significantly different from zero at the alpha = 0.05 level.
Final Selected Model: 0 Joinpoints.

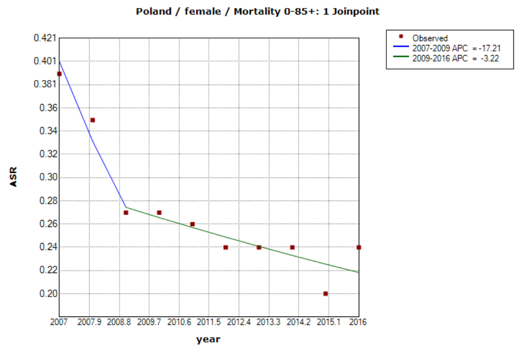

* Indicates that the Annual Percent Change (APC) is significantly different from zero at the alpha = 0.05 level.
Final Selected Model: 1 Joinpoint.

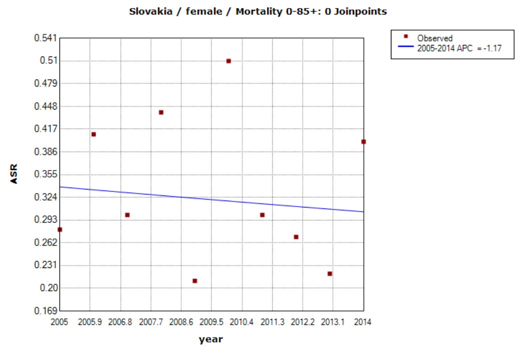

* Indicates that the Annual Percent Change (APC) is significantly different from zero at the alpha = 0.05 level.
Final Selected Model: 0 Joinpoints.

Supplementary Figure 5. AAPC of incidence of Hodgkin lymphoma aged 50 years old or above

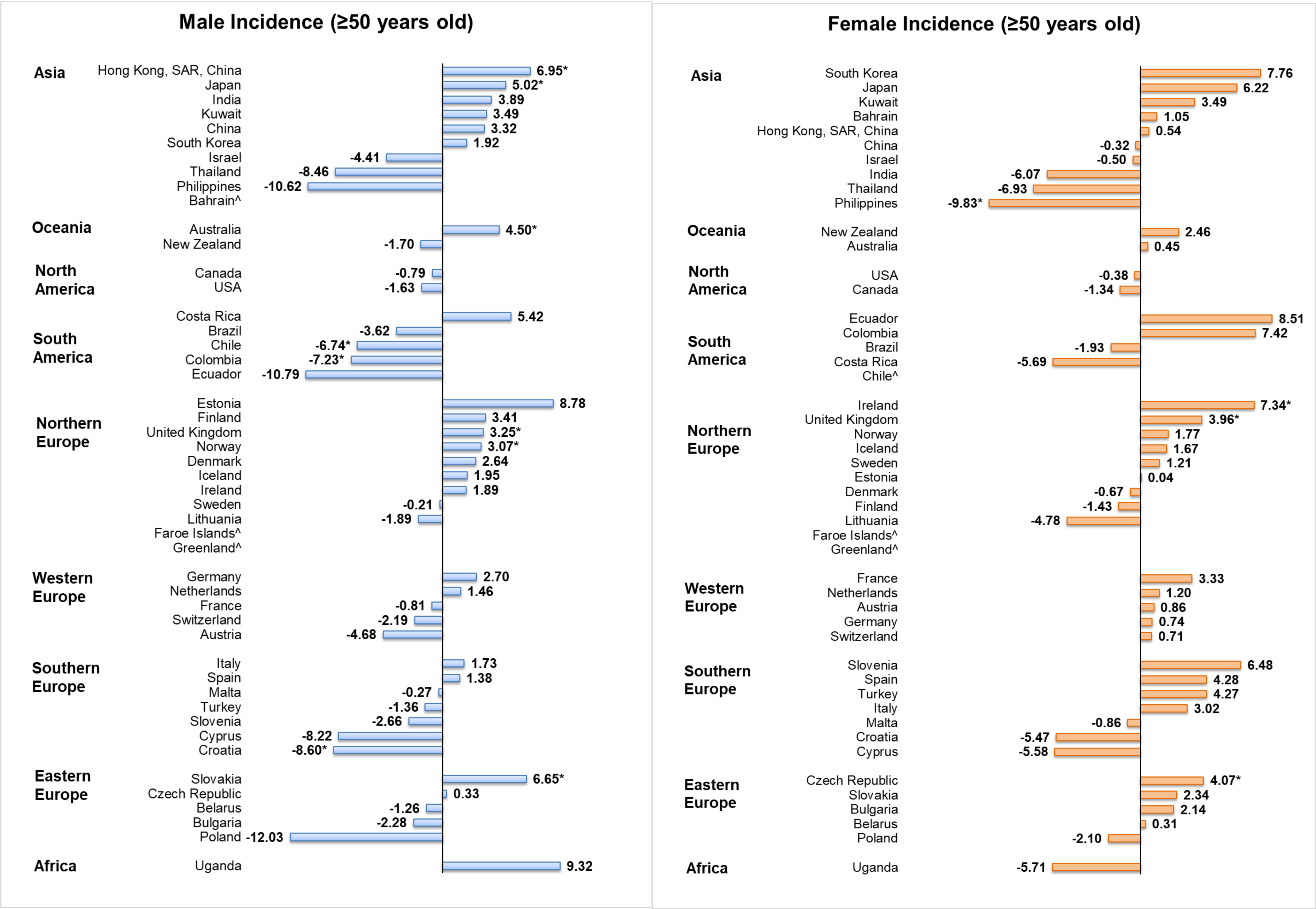

AAPC, annual percentage change; *p values less than 0.05.

Supplementary Figure 6. AAPC of incidence of Hodgkin lymphoma aged < 50 years old

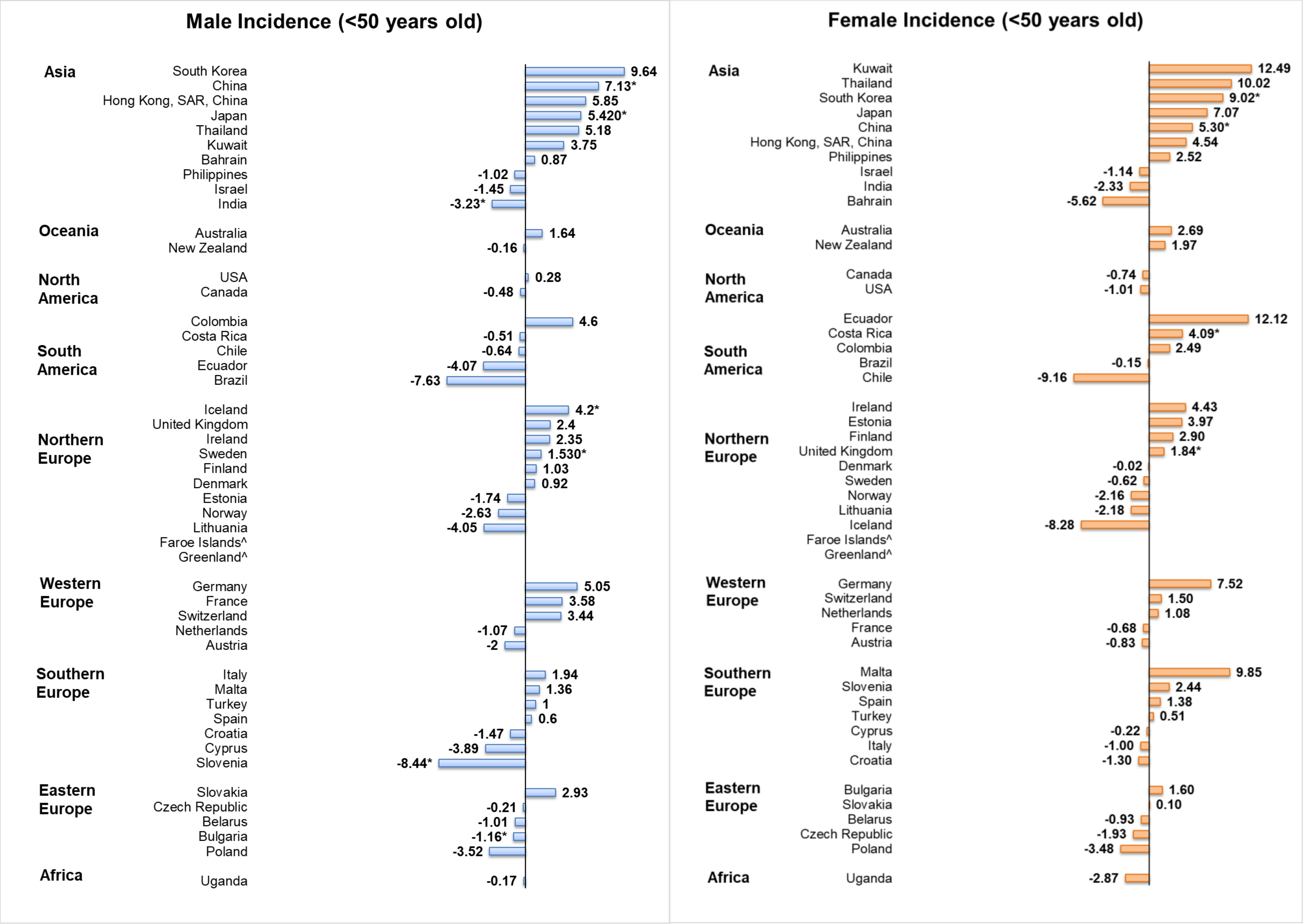

AAPC, annual percentage change; *p values less than 0.05.

Supplementary Figure 7. AAPC of incidence of Hodgkin lymphoma aged <40 years old

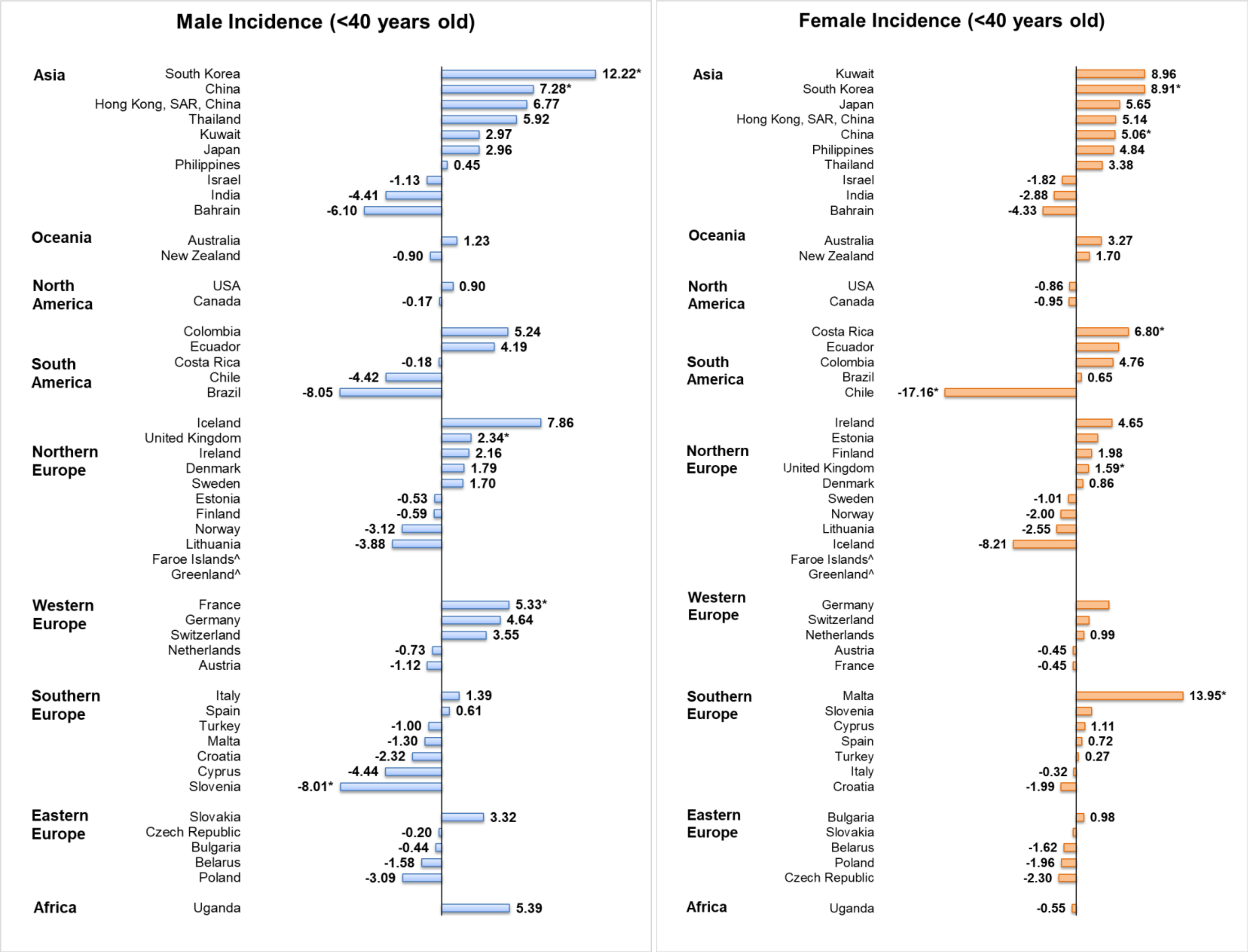

AAPC, annual percentage change; **p* values less than 0.05.
