# Supplementary material for: Incidence, mortality, risk factors, and trends for Hodgkin lymphoma: a global data analysis
Source: J Hematol Oncol. 2022 May 11;15:57. doi: 10.1186/s13045-022-01281-9 (PMC9097358; doi:10.1186/s13045-022-01281-9)
Supplement: Supplementary file 2 — Additional file 2. Table S1: Data sources for trend analysis. Table S2: Global incidence and mortality of Hodgkin lymphoma in 2020 by region, sex, and HDI. Table S3: Sensitivity analysis for mortality trend in Chile and Ireland. [file 13045_2022_1281_MOESM2_ESM.pdf]

Supplementary Tables Legends

Supplementary Table 1. Data sources for trend analysis

Supplementary Table 2. Global incidence and mortality of Hodgkin lymphoma in 2020 by region, sex and HDI

Supplementary Table 3. Sensitivity analysis for mortality trend in Chile and Ireland

Supplementary Table 1. Data sources for trend analysis

	Incidence	Mortality
Australia	CI5 (2003-2012)	WHO (2007-2016)
Austria	CI5 (2003-2012)	WHO (2008-2017)
Bahrain	CI5 (2003-2012)	n/a
Belarus	CI5 (2003-2012)	n/a
Belgium	n/a	WHO(2007-2016)
Brazil	CI5 (2003-2012)[1]	WHO (2007-2016)
Bulgaria	CI5 (2003-2012)	WHO (2006-2015)
Canada	CI5 (2003-2012)[2]	WHO (2006-2015)
Chile	CI5 (2003-2012)[3]	WHO (2007-2016)
China	CI5 (2003-2012)[4]	n/a
Colombia	CI5 (2003-2012)[5]	WHO (2006-2015)
Costa Rica	CI5 (2002-2011)	WHO (2005-2014)
Croatia	CI5 (2003-2012)	WHO (2007-2016)
Cyprus	CI5 (2003-2012)	WHO (2007-2016)
Czech Republic	CI5 (2003-2012)	WHO (2008-2017)

Denmark	NORDCAN (2010-2019)	NORDCAN (2010-2019)
Ecuador	CI5 (2003-2012)[6]	WHO (2007-2016)
Estonia	CI5 (2003-2012)	WHO (2007-2016)
Faroe Islands	n/a	n/a
Finland	NORDCAN (2010-2019)	NORDCAN (2010-2019)
France	CI5 (2002-2011)[7]	WHO (2006-2015)
Germany	CI5 (2003-2012)[8]	WHO (2007-2016)
Greenland	n/a	n/a
Hong Kong, SAR, China	CI5 (2003-2012)	WHO (2007-2016)
Iceland	NORDCAN (2010-2019)	n/a
India	CI5(2003-2012)[9]	n/a
Ireland	CI5 (2003-2012)	WHO (2006-2015)
Israel	CI5 (2003-2012)	WHO (2007-2016)
Italy	CI5 (2001-2010) [10]	WHO (2006-2015)
Japan	CI5 (2001-2010)[11]	WHO (2007-2016)
Korea (South)	CI5 (2003-2012)[12]	WHO (2007-2016)[13]

Kuwait	CI5 (2003-2012)	WHO (2005-2014)
Latvia	n/a	WHO (2006-2015)
Lithuania	CI5 (2003-2012)	WHO (2008-2017)
Malta	CI5 (2003-2012)	WHO (2006-2015)
Netherlands	CI5 (2003-2012)	WHO (2007-2016)
New Zealand	CI5 (2003-2012)	WHO (2005-2014)
Norway	NORDCAN (2010-2019)	NORDCAN (2010-2019)
Philippines	CI5 (2003-2012)[14]	WHO (2003-2012)
Poland	CI5 (2003-2012)[15]	WHO (2007-2016)
Portugal	n/a	WHO (2007-2016)
Russian Federation	n/a	n/a
Singapore	n/a	WHO (2006-2015)
Slovakia	CI5 (2001-2010)	WHO (2005-2014)
Slovenia	CI5 (2003-2012)	WHO (2006-2015)
Spain	CI5 (2001-2010)[16]	WHO (2007-2016)
Sweden	NORDCAN (2010-2019)	NORDCAN (2010-2019)

Switzerland	CI5 (2003-2012)[17]	WHO (2007-2016)
Thailand	CI5 (2003-2012)[18]	n/a
Turkey	CI5 (2003-2012)[19]	n/a
Uganda	CI5 (2003-2012)[20]	n/a
United Kingdom	CI5 (2003-2012)[21]	WHO (2007-2016)
USA	SEER(2003-2012) [22]	SEER(2007-2016) [22]
USA Black	SEER(2003-2012) [22]	SEER(2007-2016) [22]
USA White	SEER(2003-2012) [22]	SEER(2007-2016) [22]

n/a" not available; CI5: Cancer Incidence in Five Continents V; NORDCAN: Nordic Cancer Registries' SEER: USA: National Institutes of Health (NIH); WHO: World Health Organization

1. Brazil, Goiania
2. Canada (excl. Nunavut, Quebec and Yukon)
3. Chile, Valdivia
4. China (5 registries)
5. Colombia, Cali
6. Ecuador, Quito
7. France (9 registries)
8. Germany (2 registries)
9. India, Chennai
10. Italy (8 registries)

11. Japan (4 registries)
12. Korea (5 registries)
13. Republic of Korea
14. Philippines, Manila
15. Poland, Kielce
16. Spain (9 registries)
17. Switzerland (6 registries)
18. Thailand (4 registries)
19. Turkey (2 registries)
20. Uganda, Kampala
21. UK, England
22. USA, (9 registries)

Reference:

a. CI5: http://ci5.iarc.fr/CI5plus/Pages/table1_sel.aspx

b. NORDCAN: http://www-dep.iarc.fr/NORDCAN/english/frame.asp

c. SEER: http://seer.cancer.gov/data/seerstat/

d. WHO: http://apps.who.int/healthinfo/statistics/mortality/whodpms/

Supplementary Table 2. Global incidence and mortality of Hodgkin lymphoma in 2020 by region, sex and HDI

Region	Incidence						Mortality					
	Both sexes		Men		Women		Both sexes		Men		Women	
	New cases	ASR	New cases	ASR	New cases	ASR	Deaths	ASR	Deaths	ASR	Deaths	ASR
Eastern Asia	9,172	0.44	6,174	0.57	2,998	0.31	3,195	0.13	2,115	0.17	1,080	0.08
South-Eastern Asia	3,197	0.45	1,990	0.59	1,207	0.33	1,016	0.14	656	0.20	360	0.10
South-Central Asia	14,158	0.71	8,523	0.84	5,635	0.57	5,315	0.13	3,320	0.34	1,995	0.21
Western Asia	5,215	1.9	3,007	2.1	2,208	1.6	1,553	0.59	923	0.69	630	0.47
Australia and New Zealand	896	2.6	484	2.7	412	2.5	110	0.18	67	0.23	43	0.13
Melanesia	65	0.59	41	0.78	24	0.40	20	0.20	14	0.3	6	0.1
Polynesia	n/a	n/a	n/a	n/a	n/a	n/a	n/a	n/a	n/a	n/a	n/a	n/a
Micronesia	n/a	n/a	n/a	n/a	n/a	n/a	n/a	n/a	n/a	n/a	n/a	n/a
North America	9,077	2.1	5,144	2.4	3,933	1.9	1,064	0.14	633	0.20	431	0.1
Caribbean	655	1.3	371	1.6	284	1.1	235	0.41	139	0.53	96	0.31
Central America	2,888	1.5	1,686	1.9	1,202	1.2	798	0.42	476	0.54	322	0.31
South America	7,091	1.5	4,173	1.8	2,918	1.2	1,802	0.35	1,056	0.44	746	0.26
Northern Europe	3,288	2.6	1,872	3.0	1,416	2.3	511	0.23	305	0.30	206	0.16
Central and Eastern Europe	6,299	2.0	3,162	2.1	3,137	1.9	1,724	0.40	951	0.50	773	0.31
Southern Europe	4,681	2.8	2,617	3.1	2,064	2.5	973	0.31	556	0.39	417	0.23
Western Europe	5,590	2.5	3,351	3.0	2,239	2.1	745	0.17	467	0.24	278	0 1
Northern Africa	3,719	1.6	2,118	1.8	1,601	1.3	1,204	0.53	716	0.66	488	0.39
Eastern Africa	2,930	0.77	1,768	0.94	1,162	0.61	1,334	0.39	811	0.17	523	0.3
Southern Africa	712	1.0	403	1.2	309	0.86	175	0.27	108	0.36	67	0.19
Western Africa	2,815	0.86	1,705	1.1	1,110	0.67	1,299	0.45	789	0.57	510	0.34
Middle Africa	639	0.46	392	0.57	247	0.36	303	0.24	186	0.31	117	0.19
Low HDI	6,812	0.83	4,139	0.83	2,673	0.64	3,135	0.43	1,931	0.55	1,204	0.32
Medium HDI	15,752	0.69	9,323	0.78	6,429	0.57	5,974	0.27	3,646	0.33	2,328	0.21
High HDI	25,408	0.79	15,249	1.0	10,159	0.63	8,144	0.23	5,101	0.29	3,043	0.16
Very High HDI	35,098	2.0	20,260	2.2	14,838	1.70	6,123	0.23	3,610	0.29	2,513	0.16
World	83,087	0.98	48,981	1.2	34,106	0.80	23,376	0.26	14,288	0.33	9,088	0.19

HDI, human development index; ASR, age-standardized rate; a/n, data not available in the database. Data source: GLOBOCAN 2020 (https://gco.iarc.fr/)

Supplementary Table 3. Sensitivity analysis for mortality trend in Chile and Ireland

	Chile-male mortality				Ireland-male mortality			
**Year removed**	**AAPC**	**95%CI Low**	**95%CI High**	**p**	**AAPC**	**95%CI Low**	**95%CI High**	**p**
2007	8.28	2.56	14.32	0.011*	3.86	-2.41	10.54	0.193
2008	7.36	1.57	13.48	0.019*	7.33	1.09	13.96	0.027*
2009	7.83	1.87	14.13	0.016*	5.18	-1.25	12.02	0.100
2010	8.52	4.48	12.71	<0.001*	6.98	0.78	13.57	0.032*
2011	8.32	4.12	12.68	0.002*	6.59	-0.24	13.88	0.057
2012	8.32	2.28	14.72	0.013*	6.59	-0.26	13.91	0.057
2013	8.19	1.99	14.78	0.016*	6.82	0.37	13.68	0.041*
2014	7.54	1.20	14.27	0.025*	4.89	-1.12	11.27	0.097
2015	7.25	0.96	13.92	0.029*	6.63	-0.14	13.86	0.054
2016	5.74	0.24	11.54	0.043*	5.38	-1.51	12.74	0.109

AAPC, average annual percentage change; CI, confidence interval; * statistically significant
